# Plasma SOMAmer proteomics of postoperative delirium

**DOI:** 10.1002/brb3.3422

**Published:** 2024-02-12

**Authors:** Jacqueline M. Leung, Julio C. Rojas, Laura P. Sands, Brandon Chan, Binita Rajbanshi, Zhiyuan Du, Pang Du, the Perioperative Medicine Research group

**Affiliations:** ^1^ Department of Anesthesia and Perioperative Care University of California San Francisco San Francisco California USA; ^2^ Memory and Aging Center, Department of Neurology, Weill Institute for Neurosciences University of California San Francisco San Francisco California USA; ^3^ Virginia Tech, Center for Gerontology Blacksburg Virginia USA; ^4^ Virginia Tech, Department of Statistics Blacksburg Virginia USA

**Keywords:** biomarkers, postoperative delirium, proteomics

## Abstract

**Background:**

Postoperative delirium is prevalent in older adults and has been shown to increase the risk of long‐term cognitive decline. Plasma biomarkers to identify the risk for postoperative delirium and the risk of Alzheimer's disease and related dementias are needed.

**Methods:**

This biomarker discovery case–control study aimed to identify plasma biomarkers associated with postoperative delirium. Patients aged ≥65 years undergoing major elective noncardiac surgery were recruited. The preoperative plasma proteome was interrogated with SOMAmer‐based technology targeting 1433 biomarkers.

**Results:**

In 40 patients (20 with vs. 20 without postoperative delirium), a preoperative panel of 12 biomarkers discriminated patients with postoperative delirium with an accuracy of 97.5%. The final model of five biomarkers delivered a leave‐one‐out cross‐validation accuracy of 80%. Represented biological pathways included lysosomal and immune response functions.

**Conclusion:**

In older patients who have undergone major surgery, plasma SOMAmer proteomics may provide a relatively non‐invasive benchmark to identify biomarkers associated with postoperative delirium.

## Graphical abstract

This biomarker discovery case‐control study aimed to identify plasma biomarkers associated with postoperative delirium. The preoperative plasma proteome was interrogated with SOMAmer‐based technology targeting 1,433 biomarkers. In 40 patients (20 with *vs*. 20 without postoperative delirium), a preoperative panel of 12 biomarkers discriminated patients with postoperative delirium with an accuracy of 97.5%.

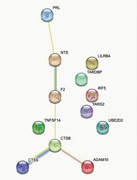

## 1 INTRODUCTION

Delirium is a common yet serious cognitive condition that affects 10%–60% of patients after major surgery (Lipowski, 1989). Delirium is an acute confusional state defined by alterations in attention, changes in consciousness, and disorganized thinking (Lipowski, 1989) and has been associated with decreased long‐term physical and cognitive functioning (Davis et al., 2017). The development of delirium is thought to be a multifactorial process in which there is a complex interrelationship between baseline patient vulnerability and precipitating factors. Perioperatively, there is a concern that exposure to anesthetics and other drugs precipitates delirium which triggers an irreversible neurodegenerative process.

Despite this concern, studies that have hypothesized this potentially causal relationship between anesthetic exposure and subsequent cognitive changes including postoperative delirium did not provide mechanistic evidence for this hypothesis. Importantly, most studies did not include the baseline vulnerability of patients as an important contributing factor to the occurrence of postoperative cognitive changes. Our proof‐of‐concept project fills the gap from previous studies to identify evidence of the critical importance of preoperative brain vulnerability including the role of molecular pathways driving perioperative cognitive changes.

Emerging evidence suggests that plasma biomarkers may reflect brain pathology of Alzheimer's disease and related dementias (ADRD) (Guzman‐Martinez et al., 2019). However, the relationship between plasma biomarkers and postoperative delirium is much less definitive. Prior work that examined the association between preoperative biomarkers and postoperative delirium revealed conflicting results because of the small number of biomarkers being examined and heterogeneous methods of statistical analysis (Casey et al., 2020; Fong et al., 2020; McKay et al., 2022). Accordingly, we undertook a case–control study to identify preoperative plasma biomarkers associated with postoperative delirium by utilizing a customized panel of 1433 biomarkers proteins quantified with SOMAmer‐based technology. SOMAmers are short, single‐stranded deoxyoligonucleotides that bind to proteins with high specificity. Here, we explore the potential of SOMAmer‐based proteomics as a discovery assay for a high‐throughput, rapid, efficient, and seamless identification of plasma biomarkers of postoperative delirium (Kraemer et al., 2011).

We hypothesized that postoperative delirium would be associated with processes that may be potentially upstream of the neurodegeneration cascade.

## 2 METHODS

### 2.1 Participants and plasma samples

The study was approved by the Institutional Review Board for Human Research, and written informed consent was obtained preoperatively from each study patient. The study was conducted at the University of California, San Francisco Medical Center between January 2002 and December 2010. Data for this study were collected from two separate studies. One study assessed the effects of nitrous oxide on postoperative delirium which showed that there was no effect of the intervention on postoperative delirium (Leung et al., 2006). The other study was an observational study of risk factors associated with postoperative delirium (Leung et al., 2013). Inclusion criteria were consecutive men or women who were ≥65 years of age, undergoing major noncardiac surgery requiring general anesthesia, who were expected to remain in the hospital postoperatively for ≥48 h, and also had plasma banked preoperatively for the biomarkers assay. Exclusion criteria were patients whose primary language was not English. Our study cohort was largely cognitively unimpaired. The patients selected for this study were based on matching scheme to ensure there was a sufficient number of individuals with delirium and low versus high preoperative cognitive status in order to ensure a sample with sufficient variability. The sample size was based primarily on the allowable budget for the biomarker assay.

Preoperatively on the day of surgery, blood was collected by phlebotomy in EDTA tubes, and centrifuged at 2500 *g* for 10 min at room temperature. Plasma was then aliquoted in 1.5‐mL cryogenic tubes and stored at −80°C until analyses.

### 2.2 SOMAmer proteomics

The SomaScan assay was performed under the control of the SomaLogic Quality System (QS) in a laboratory that is CLIA accredited (Somalogic Operating Co., Inc.). The assay used 55 μL of plasma, and multiplex assays were run in parallel in 96‐well plates. Assay qualification studies show that the assay performs with a median sensitivity of 100 fM (lower limit of detection) and median coefficient of variation of ∼5% across all analytes measured repeatedly in replicate runs of plasma samples. The overall detection range of the platform spans 10 logs in plasma or serum. The SOMAscan proteomics platform version 4.0 was used to quantify a customized panel of 1433 biomarkers. The biomarkers were selected based on previous literature supporting markers involved in aging, neurodegeneration, mitochondrial markers linked to bioenergetic deficits, neuroinflammation, cytokine, and innate immunity markers, pathways potentially upstream to the neurodegeneration cascade. A full list of all the targets is provided in Table S1.

### 2.3 Single analyte validation

To validate the SOMAscan methods of biomarkers assay, we performed ultrasensitive single‐analyte plasma as orthogonal validation as a proof‐of‐concept that markers identified with the SOMAscan platform can be followed up for clinical deployment. One of the top biomarkers identified, prolactin, was measured with electrochemiluminescence, using commercially available kits for the MESO QuickPlex SQ 120 platform (Meso Scale Diagnostics). Plasma samples were measured in duplicate, following kit instructions, by investigators blinded to participant identity or group allocation. The lowest limit of quantification for the assay was 2.1 pg/mL, and the average coefficient of variation was 8.15%.

### 2.4 Cognitive and delirium measurements

Baseline cognitive status was measured preoperatively using the Telephone Interview of Cognitive Status instrument (Brandt et al., 1988) which was adapted from the Mini Mental State Examination. For the occurrence of delirium, we used the Confusion Assessment Method Rating Scale (CAM) (Inouye et al., 1990) which was developed as a screening instrument based on the operationalization of DSM‐III‐R criteria for use by nonpsychiatric clinicians in high‐risk settings. The CAM assessments were conducted by research associates using the CAM Training Manual and Coding Guide. All assessments were validated by either Drs. Leung or Sands. The CAM was administered before surgery and daily after surgery for up to 3 days. No patients met CAM criteria for delirium prior to surgery. The primary outcome was incident delirium on any of the first three postoperative inpatient days.

### 2.5 Statistical analysis

We undertook an age‐ and sex‐matched case–control study of 20 patients who developed postoperative delirium, and 20 who did not. All the analyses were performed on log2‐transformed and standardized biomarker values. To gain an overall insight, we created a volcano plot of the 1433 biomarkers, where the biomarker values for the two groups were compared through separate two‐sample *t*‐tests. Top targets hits were considered biologically and statistically significant if |log2(fold change)| ≥0.2 and *p*‐value < 05. Significant targets were ranked by *p*‐values and entered in the online STRING database for known and predicted physical and functional protein–protein interactions (Snel et al., 2000). Functional enrichment within the Cellular Component (Gene Ontology) and Kyoto Encyclopedia of Genes and Genomes (KEGG) pathways was tested against the whole genome. The enrichment strength was expressed as Log10(observed/expected), and significant associations were ranked by *p*‐values corrected by false discovery rate. Recognizing that such analysis does not fully address the multiple comparison issue and possible interactions between biomarkers, we performed a more elaborative three‐step selection as follows.

This pilot proteomics dataset represents a typical example of ultra‐high dimensional data in statistical analysis, where the number of predictors (*p* = 1433) is much larger than the sample size (*n* = 40) (Fan & Lv, 2008). First, we applied a sure independence screening procedure to reduce the number of candidate biomarkers to a much smaller scale comparable to the sample size. Blindly analyzing all the biomarkers without screening can lead to erroneous results due to noise accumulation, spurious correlation, and high collinearity (Fan & Fan, 2008; Fan & Lv, 2008, 2010). Such a screening procedure often involves applying a statistical test to each biomarker separately, ranking their significances from the highest to the lowest (or the *p*‐values from the lowest to the highest), and keeping only the *d*_n_ most significant ones. Common choices of the statistical test are the two‐sample *t*‐test, the Kolmogorov–Smirnov test (Mai & Zou, 2013), and the Mann–Whitney test (Sun et al., 2020) with a recommended *d*_n_ of n/ln(n) = 40/ln(40) = 11. We carried out the screening procedure with each of these tests using a more conservative choice of *d*_n_ = 50. The biomarkers commonly appearing in these three 50‐biomarker sets were identified and used for all the ensuing analyses.

Next, we fitted a logistic regression model with delirium status against these screened biomarkers and applied a regularized variable selection procedure to further pinpoint the significant biomarkers. Commonly used regularized variable selection procedures are the lasso procedure (Tibshirani, 1996) and the smoothly clipped absolute deviation procedure (Fan & Li, 2001). Here, we used the lasso procedure (R package glmnet). To assess the individual predictive power of each of these biomarkers, we conducted a separate receiver operating curve (ROC) analysis and recorded the corresponding area under the ROC curve (AUC). To avoid over‐fitting, we added a further selection step by the Akaike information criterion using the R function stepAIC.

To validate the final model, we conducted a leave‐one‐out cross‐validation (Hastie et al., 2016). A logistic regression model with the final biomarkers was fitted using data from all but one subject to predict the delirium status of the left‐out subject. We plugged the biomarker measurements of the left‐out subject into the fitted logistic regression model to obtain a predicted probability of having delirium for the subject. The probability was then compared with 0.5 to determine the delirium status of the subject: the subject was classified to have delirium if the predicted probability ≥0.5 and had no delirium otherwise. We repeated this process with each of the 40 subjects left out of the training data exactly once. Finally, the average classification accuracy was recorded as the proportion of correct leave‐one‐out delirium status predictions.

## 3 RESULTS

The demographic data of the patients are shown in Table 1. The mean age of the patients was 70.28 ± 5.85 years. Note that 62.5% of the patients were female.

**TABLE 1:** Clinical characteristics of the patients.

Characteristic	Delirium *n* = 20 (50%)	No delirium N = 20 (50%)	*p*‐value
Age, mean ± SD	71.6 ± 5.9	69.0 ± 5.6	.15
Female, n/N (%)	15/20 (75)	10/20 (50)	.19
Living independently, n/N (%)	5/19 (26.3)	2/18 (11.1)	.24
Low educational level, n/N (%) (1 vs. 2, 3, 4, 5)	2/19 (10.5)	1/20 (5)	.99
Low educational level, n/N (%) (1, 2 vs. 3, 4, 5)	6/19 (31.6)	4/20 (10)	.72
American Society of Anesthesiologists group, n/N (%)			.33
I	1/20 (5)	0/20	
II	6/20 (30)	10/20 (50)	
III	13/20 (65)	10/20 (50)	
Number of comorbid diseases, median (IQR)	2 (2)	1 (1)	.18
TICS State Examination score, median (IQR)	32 (6.78)	33.5 (8.73)	.31

*Note*: The clinical characteristics of the patients with and without postoperative delirium are shown in Table 1. Educational level: 1 = not completed high school, 2 = high school completion, 3 = some college, 4 = college completion, 5 = postgraduate training or degree. *p*‐Values are either calculated by the two‐sample *t*‐test for continuous data and chi‐square test or Fisher exact test for the categorical data.

Abbreviation: TIC, telephone interview of cognitive status.

**FIGURE 1:**
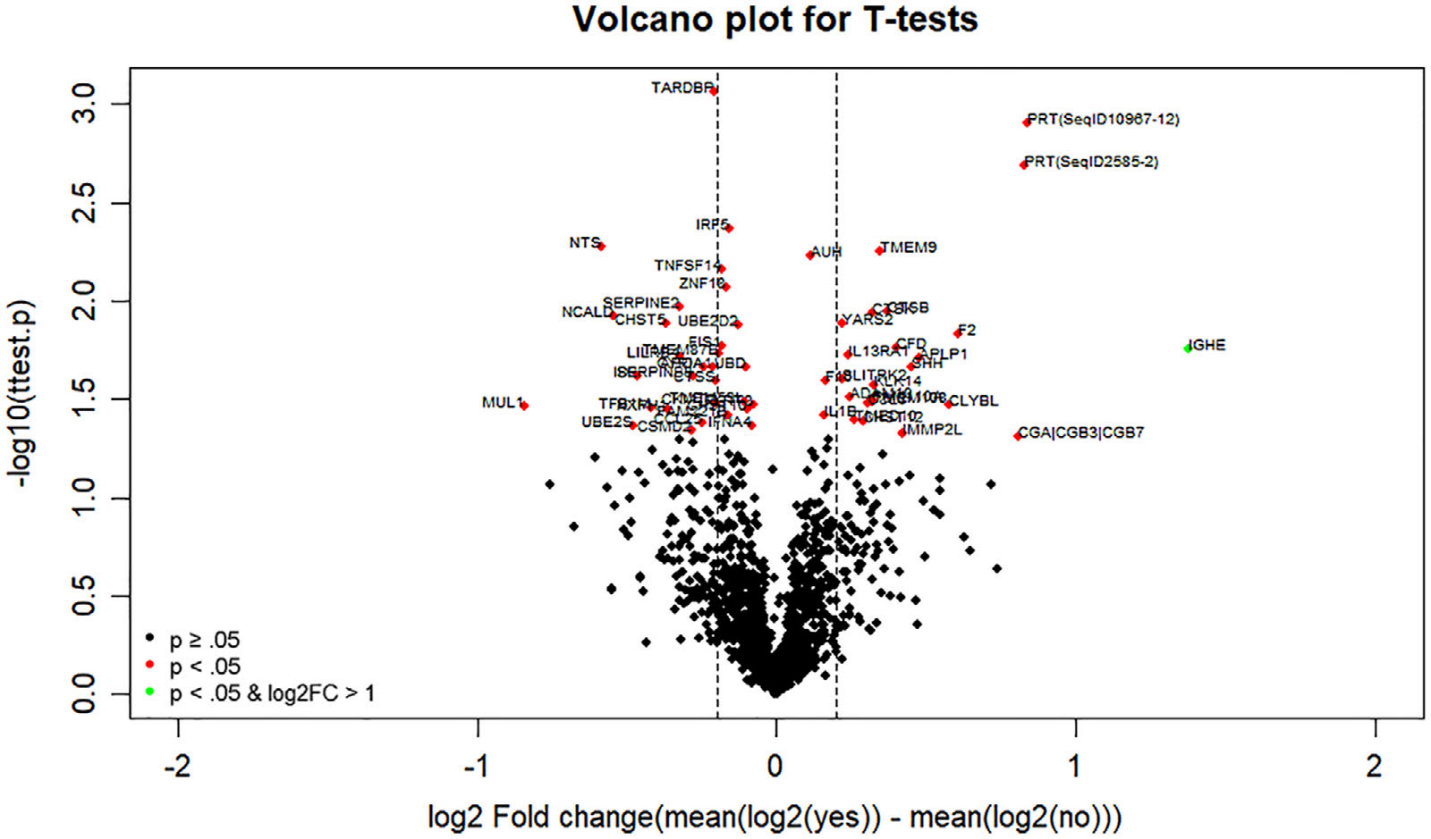
This figure shows the volcano plot, where the biomarkers with low *p*‐values (highly significant, in red) appear toward the top of the plot. The *x*‐axis is the logarithm of the fold change between the two conditions.

Of the 1433 plasma markers, 56 (4.0%) were differentially expressed between groups: 26 were upregulated, 30 were downregulated, and 1377 showed no between groups differences (Figure 1). Figure 1 shows the volcano plot, where the biomarkers with low *p*‐values (highly significant, in red) appear toward the top of the plot. The *x*‐axis is the logarithm of the fold change between the two conditions. Differentially expressed markers were related to the toll‐like receptor signaling pathway (strength = 1.27, *p* = .003) and cytokine–cytokine receptor interaction (strength = 0.97, *p* = .0030) KEGG pathways and endolysosome, endosome and extracellular matrix Gene Ontology cellular components.

**FIGURE 2:**
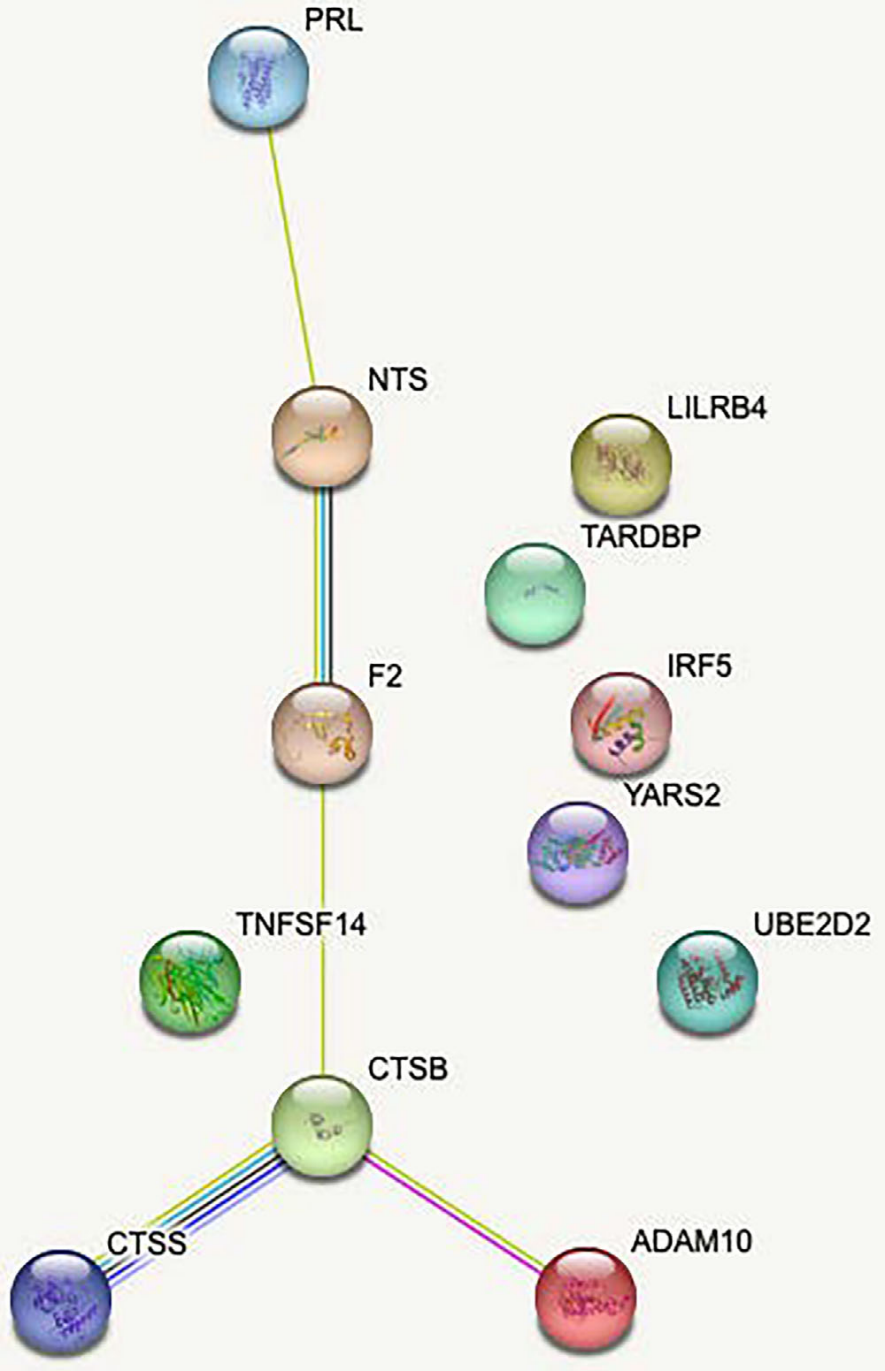
The final model contained 12 biomarkers which were entered into the STRING database for biological systems enrichment effect analyses for visualization. ADAM 10, ADAM 10 metallopeptidase domain 10; CTSB, cathepsin B; CTSS, cathepsin S; F2, coagulation factor II, thrombin; IRF5, interferon regulatory factor 5; LILRB4, leukocyte immunoglobulin‐like receptor B4; NTS, neurotensin; PRL, prolactin; TARDP, TAR DNA binding protein; TNFSF4, TNF superfamily 4; UBE202, ubiquitin conjugating enzyme E2 D2; YARS2, tyrosyl‐tRNA synthetase 2.

Comparing the 56 targets obtained from separate *t*‐tests, the screening procedure reduced the 1433 biomarkers to 15 biomarkers that consistently discriminated between groups. We then fitted a logistic regression model with postoperative delirium status as the response and the 15 biomarkers as the predictors. In the model, the biomarkers were further selected by the lasso procedure (Friedman et al., 2010; Tibshirani, 1996). The resulting model contained 12 biomarkers that could deliver an in‐sample classification accuracy of 97.5% (specific = 0.95 and sensitivity = 1); that is, the misclassification of delirium status occurred for only one of 40 patients who had no delirium but was misclassified as having delirium. These 12 biomarkers were entered into the STRING database (Szklarczyk et al., 2017) for biological systems enrichment effect analyses for visualization (Figure 2). No KEGG pathways were represented, but they redemonstrated enrichment for endolysosome lumen (strength = 2.81, *p* = .0061), endosome lumen (strength = 2.15, *p* = .0025), and extracellular space (strength = 0.61, *p* = .049) cellular components and immune response (strength = 0.91, *p* = .0091) biological processes in Gene Ontology. To assess the individual prediction power for each of the 12 biomarkers, we also conducted a separate ROC curve analysis for each biomarker, and their respective areas under the ROC curve (AUCs) are summarized in Table 2. The 12‐biomarker model can discriminate the 40 patients with an almost perfect in‐sample accuracy of 97.5%. The top three AUCs for individual biomarkers are from TARDBP (0.80), YARS2 (0.79), and prolactin (PRL) (0.78). The thresholds optimizing the Youden index (Zhou et al., 2011) for the three biomarkers are TARDBP (delirium if ≤0.33 with the max Youden index = 0.55), YARS2 (delirium if ≥−0.22 with the max Youden index = 0.5), and PRL (delirium if ≥0.39 with the max Youden index = 0.5). The sensitivities and specificities delivered by these optimal thresholds for the three biomarkers are TARDBP (sensitivity = 0.95, specificity = 0.6), YARS2 (sensitivity = 0.7, specificity = 0.8), and PRL (sensitivity = 0.7, specificity = 0.8). From the LASSO coefficients (Table 2), several of these biomarkers were confirmed to have between‐groups discriminating power. In addition, although biomarker TNFSF14 is not excluded from the model by the lasso procedure, its effect is almost negligible with a coefficient very close to 0.

**TABLE 2:** Properties of the 12 biomarkers.

Target	Target full name	AUC	Coefficients
TARDBP	TAR DNA‐binding protein 43	0.80	−0.595
YARS2	Tyrosyl‐tRNA synthetase, mitochondrial	0.79	0.351
PRL	Prolactin	0.78	0.679
IRF5	Interferon regulatory factor 5	0.78	−0.105
TNFSF14	Tumor necrosis factor ligand superfamily member 14	0.77	−0.002
ADAM10	Disintegrin and metalloproteinase domain‐containing protein	0.75	0.534
CTSB	Cathepsin B	0.74	0.121
LILRB4	Leukocyte immunoglobulin‐like receptor subfamily B member 4	0.74	−0.025
F2	Thrombin	0.74	0.562
UBE2D2	Ubiquitin‐conjugating enzyme E2 D2	0.73	−0.079
CTSS	Cathepsin S	0.73	−0.608
NTS	Neurotensin/neuromedin N	0.73	−0.326

*Note*: A logistic regression model was fitted with postoperative delirium status as the response and the 15 biomarkers as the predictors that were further selected by the lasso procedure. The final model contained 12 biomarkers that could deliver an in‐sample classification accuracy of 97.5%. AUC is calculated as the empirical AUC where the Mann–Whitney test is used. The coefficients were computed from the LASSO model, see text for details.

Abbreviation: AUC, area under the curve.

From the LASSO coefficients (Table 2), the vascular wall biology targets (i.e., F2, ADAM10, and CTSB) all show positive effects, meaning that patients with higher values of these biomarkers are more likely to have delirium. Specifically, a one‐unit increase in the value of biomarker F2 would result in an increase of the odds ratio (delirium vs. non‐delirium) by a factor of exp(0.562) = 1.75, a one‐unit increase in the value of biomarker ADAM10 would result in an increase in the odds ratio (delirium vs. non‐delirium) by a factor of exp(0.534) = 1.71, and a one‐unit increase in the value of biomarker CTSB would result in an increase in the odds ratio (delirium vs. non‐delirium) by a factor of exp(0.121) = 1.13. In addition, a one‐unit increase in the values of biomarkers YARS2 and PRL would result in odds ratio increases by factors of exp(0.351) = 1.42 and exp(0.679) = 1.97. On the other hand, several biomarkers showed negative effects, meaning that patients with lower values of these biomarkers are more likely to have delirium. In particular, a one‐unit increase in the values of biomarkers TARDBP, IRF5, LILRB4, UBE2D2, CTSS, and NTS would result in odds ratio decreases by factors of exp(−0.595) = 0.55, exp(−0.105) = 0.9, exp(−0.025) = 0.98, exp(−0.079) = 0.92, exp(−0.608) = 0.54, and exp(−0.326) = 0.72. Lastly, although biomarker TNFSF14 is not excluded from the model by the lasso procedure, its effect is almost negligible with a coefficient (= −0.002) very close to 0.

**FIGURE 3:**
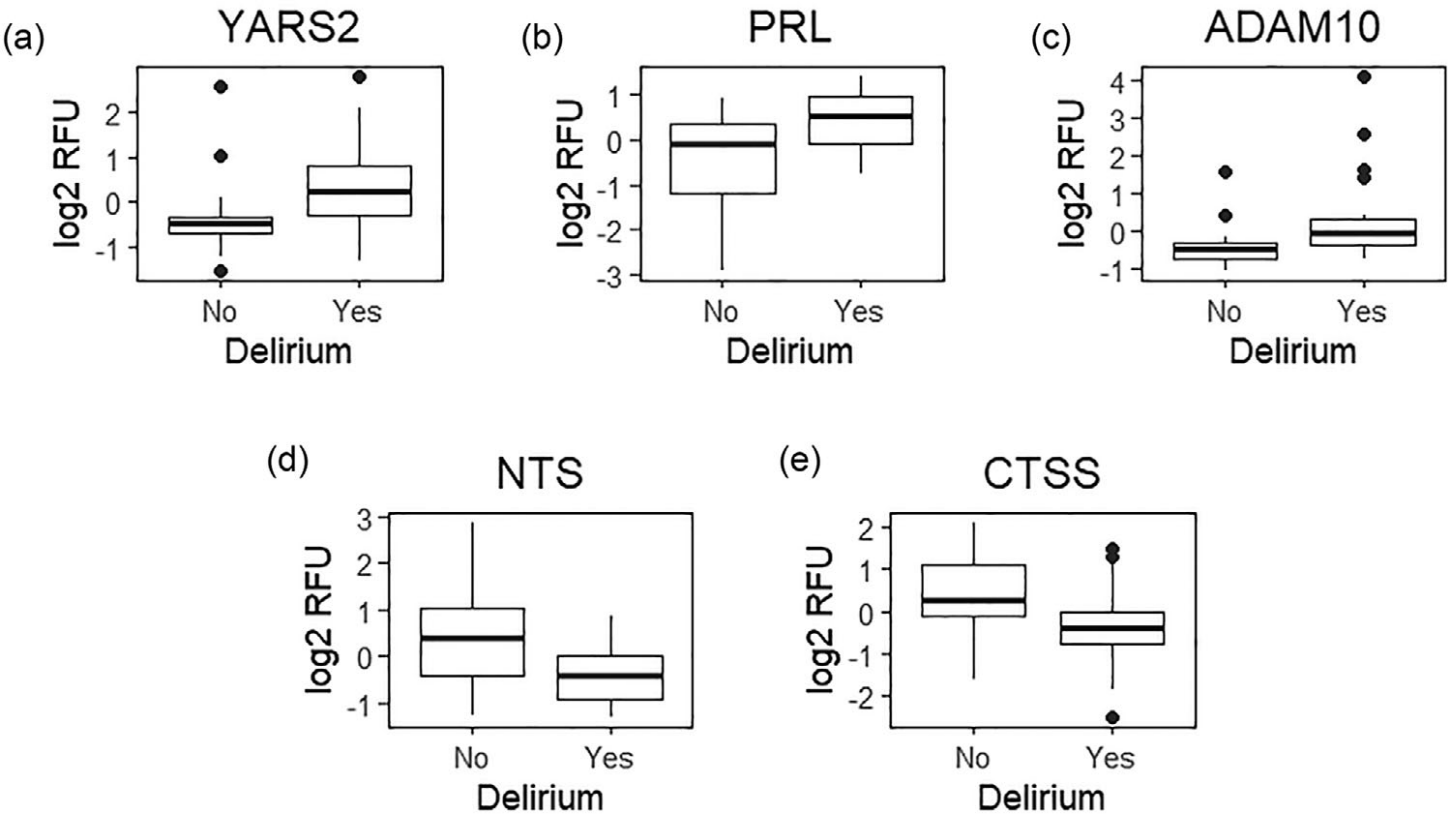
The figure contains the boxplots for the five biomarkers selected by the stepAIC, where the *x*‐axis is the group of delirium status, and the *y*‐axis is the log2 of relative fluorescent units.

The stepAIC further narrowed down the model to five biomarkers: “YARS2,” “PRL,” “ADAM10,” “NTS,” and “CTSS.” The leave‐one‐out cross‐validation yielded an average delirium status prediction accuracy of 0.80 (sensitivity = 0.75, specificity = 0.85), which confirms the excellent generalizability of the prediction power for this small set of five biomarkers. Figure 3 shows the boxplots for the five biomarkers selected by the stepAIC, where the *x*‐axis is the group of delirium status, and the *y*‐axis is the log2 of relative fluorescent units.

We performed a preliminary validation of the SOMA scan using electrochemiluminescence of one of the top biomarkers, prolactin. The AUC was 0.76 (95% confidence interval 0.59–0.87) in the prediction of postoperative delirium, which was comparable to an AUC of 0.78 from the SOMA scan.

## 4 DISCUSSION

In this case–control study, a number of proteins involving immune, endosome, and endolysosome function were differentially expressed in the plasma of cognitively healthy individuals with postoperative delirium and have potential as prognostic biomarkers of postoperative delirium. The evidence supports that high‐throughput screening with SOMAmer‐based proteomics may identify proteins of clinical value to identify individuals at risk of postoperative delirium. These preliminary data support that delirium may be mediated by cellular pathways acting upstream or parallel to established neurodegeneration cascades associated with age‐related cognitive impairment.

### 4.1 Comparison with previous studies

Prior studies of delirium biomarkers have primarily focused on the changes in biomarker levels after surgery. Studies that investigated preoperative biomarkers showed conflicting results and evaluated only a few biomarkers (Ballweg et al., 2021; Casey et al., 2020; Cunningham et al., 2019; Fong et al., 2020; Halaas et al., 2018; Idland et al., 2017; Liang et al., 2022; McKay et al., 2022; Witlox et al., 2011; Xie et al., 2014). Several studies that used the SOMAscan did not report on whether there was any difference in preoperative levels in the biomarkers between patients with versus without postoperative delirium (Dillon et al., 2023; Fong et al., 2021; Rhee et al., 2021; Vasunilashorn et al., 2022); hence, a direct comparison to our present results was not possible.

The top differentially expressed plasma proteins in delirium implicate diverse biological pathways related to neurodegeneration. TAR DNA‐binding protein 43 is an RNA/DNA‐binding protein involved in RNA regulation and diseases. It is the pathological hallmark in both amyotrophic lateral sclerosis and frontotemporal lobar dementia (Lye & Chen, 2022). It is also present in a large portion of patients with Alzheimer's disease (AD), where its accumulation is related to worse memory function (Tremblay et al., 2011). Tyrosyl‐tRNA synthetase, mitochondrial (YARS2) is inhibited by tyrosine, its levels increase during aging, neurocognitive, metabolic, and cardiovascular disorders (Jhanji et al., 2022). It is hypothesized that the decreased protein synthesis in AD brains is due to decreased activity of YARS2 (Jhanji et al., 2022). Interferon regulatory factor 5 (IRF5) is a member of the interferon regulatory (IRF) family, a group of transcription factors with diverse roles, including virus‐mediated activation of interferon, and modulation of cell growth, differentiation, apoptosis, and immune system activity (Al Mamun et al., 2018). Finally, PRL is one of the most pleiotropic pituitary hormones and is known to modulate normal neuronal function and neurodegenerative conditions. Many studies have described the influence that PRL has on the central nervous system and addressed its contribution to neurodegeneration. A previous study indicated that high and low levels of PRL were related to cognitive impairment in tasks involving processing speed and verbal recall in older men and that higher PRL levels were related to poorer working memory and verbal ability (Castanho et al., 2014). Therefore, our current results provide a new paradigm to study the pathophysiology of postoperative delirium, demonstrating that multiple biological pathways should be investigated on the molecular basis and subsequent related cognitive changes, particularly as it relates to ADRD. A recent systematic review of proteomics and delirium concurred with our present results that a system‐biology view of the mechanism of delirium needs to be pursued (Wiredu et al., 2023).

There are some potential clinical implications for our results. First, the identification of biologic pathways manifested by biofluid protein markers preoperatively suggests there is a pre‐existent vulnerability. Second, postoperative delirium has always been described as having sequelae of further cognitive and physical functional decline. However, studies that have hypothesized this potentially causal relationship did not provide mechanistic evidence for this hypothesis. The present results support the value of discovery proteomic tools to identify objective biological measures of brain vulnerability and understand the biological mechanism underlying delirium.

There are several potential limitations of our study. First, the sample size is small, and there was no replication cohort. Second, a customized panel of targets was selected from the available catalog of about 7000 targets due to budgetary constraints. However, we chose targets involved in pathways that were potentially upstream of the neurodegeneration cascade. Third, SOMAscan needs orthogonal validation of results with single analyte or mass spectrometry measures, analysis of neuropathological specimens, or pursuit of proteogenomic association studies, which was attempted here only as proof of concept. Our study focused on plasma proteomics, since this can yield easily deployable clinical biomarkers, but improved biological signatures may be obtained by analysis of cerebrospinal fluid, which may reflect the brain neurochemistry more accurately. The SOMAscan platform does not detect aberrant proteins such as β‐amyloid or phosphorylated tau, which have been strongly implicated as markers of neurodegeneration, and functional proteomics studies are needed in delirium. Lastly, this is an exploratory study with a small sample size; hence, we were not able to include potential effects of other co‐variates such as age or gender. A future larger study will need to include these potential co‐variates of postoperative delirium.

## 5 CONCLUSION

The plasma proteome of cognitively healthy individuals who undergo surgery and develop postoperative delirium is distinctive, compared to those who do not develop delirium. In older patients awaiting surgery, plasma biomarkers may provide a relatively non‐invasive benchmark for molecular pathways associated with postoperative delirium.

## AUTHOR CONTRIBUTIONS

Jacqueline M. Leung: Study design; data analysis; data collection; written the first draft of the paper; review of final version of paper. Julio C. Rojas: Data analysis; written the first draft of the paper; review of final version of paper. Laura P. Sands: Study design; data analysis; written of the first draft of the paper; review of final version of paper. Brandon Chan: Data analysis; written the first draft of the paper; review of final version of paper. Binita Rajbanshi: Data analysis; written the first draft of the paper; review of final version of paper. Zhiyuan Du: Data analysis; written the first draft of the paper; review of final version of paper. Pang Du: Data analysis; written the first draft of the paper; review of final version of paper.

## CONFLICT OF INTEREST STATEMENT

The authors declare no conflict of interest.

### PEER REVIEW

The peer review history for this article is available at https://publons.com/publon/10.1002/brb3.3422.

## Supporting information

Supplemental table 1 SomaScan 7k Custom Panel Selection (file attached separately)

## Data Availability

Data may be available for sharing pending on approval from authors and institutional review committee's from the requester's home institution and that of the authors.
