# Supplementary material for: Plasma SOMAmer proteomics of postoperative delirium
Source: Brain Behav. 2024 Feb 12;14(2):e3422. doi: 10.1002/brb3.3422 (PMC10861352; doi:10.1002/brb3.3422)
Supplement: Supplementary file 1 — Supplemental table 1 SomaScan 7k Custom Panel Selection (file attached separately) [file BRB3-14-e3422-s001.docx]

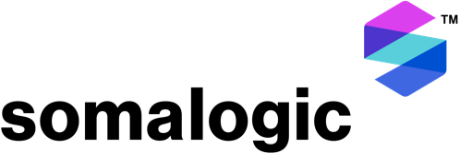


| GDF | | | | | | | | | | |
| --- | --- | --- | --- | --- | --- | --- | --- | --- | --- | --- |
|  | | | | | | | **SomaLogic Panels** | | | |
| # | **Custom Panel (X)** | **SOMAmer SeqID** | **Target Name** | **Human Target or Analyte** | **UniProt ID** | **GeneID** | **Cardiovascular Disease** | **Inflammation and Immune**  **Response** | **Metabolic Disease** | **Oncology** |
| *#* X *12345-1 Example Custom Panel Selection* | | | | | | | | | | |

| 1 | 18395-5 | ADPRH | [Protein ADP-ribosylarginine] hydrolase | P54922 | ADPRH |  |  | | |
| --- | --- | --- | --- | --- | --- | --- | --- | --- | --- |
| 2 | 5227-60 | PDK1 | [Pyruvate dehydrogenase (acetyl-transferring)] kinase isozyme 1, mitochondrial | Q15118 | PDK1 |  |
| 3 | 12651-21 | PDK2 | [Pyruvate dehydrogenase (acetyl-transferring)] kinase isozyme 2, mitochondrial | Q15119 | PDK2 | X |
| 4 | 22005-8 | PDP1 | [Pyruvate dehydrogenase [acetyl-transferring]]-phosphatase 1, mitochondrial | Q9P0J1 | PDP1 |  |  | X |  |
| 5 | 12457-10 | MTND | 1,2-dihydroxy-3-keto-5-methylthiopentene dioxygenase | Q9BV57 | ADI1 |  |  |  |  |
| 6 | 17150-8 | HSP 10 | 10 kDa heat shock protein, mitochondrial | P61604 | HSPE1 | X |  |  |  |
| 7 | 16882-27 | PHP14 | 14 kDa phosphohistidine phosphatase | Q9NRX4 | PHPT1 |  |  |  |  |
| 8 | 12414-31 | 14-3-3 protein beta/alpha | 14-3-3 protein beta/alpha | P31946 | YWHAB |  |  |  |  |
| 9 | 14156-33 | 14-3-3 protein beta/alpha | 14-3-3 protein beta/alpha | P31946 | YWHAB |  |  |  |  |
| 10 | 14157-21 | 14-3-3E | 14-3-3 protein epsilon | P62258 | YWHAE |  |  |  |  |
| 11 | 22049-24 | 14-3-3 eta | 14-3-3 protein eta | Q04917 | YWHAH | X |  | X |  |
| 12 | 4707-50 | 14-3-3 eta | 14-3-3 protein eta | Q04917 | YWHAH |  |  |  |  |
| 13 | 4179-57 | 14-3-3 protein gamma | 14-3-3 protein gamma | P61981 | YWHAG |  |  |  |  |
| 14 | 8901-40 | 14-3-3 protein gamma | 14-3-3 protein gamma | P61981 | YWHAG |  |  |  |  |
| 15 | 4829-43 | STRATIFIN | 14-3-3 protein sigma | P31947 | SFN |  |  |  | X |
| 16 | 7625-27 | 14-3-3 protein theta | 14-3-3 protein theta | P27348 | YWHAQ |  |  |  |  |
| 17 | 5858-6 | 14-3-3 protein zeta/delta | 14-3-3 protein zeta/delta | P63104 | YWHAZ |  |  |  | X |
| 18 | 20087-3 | selenoprotein15 | 15 kDa selenoprotein | O60613 | SELENOF |  |  |  |  |
| 19 | 4995-16 | HPG- | 15-hydroxyprostaglandin dehydrogenase [NAD(+)] | P15428 | HPGD |  |  |  | X |
| 20 | 13972-4 | DHB14 | 17-beta-hydroxysteroid dehydrogenase 14 | Q9BPX1 | HSD17B14 |  |  |  |  |
| 21 | 23393-56 | ABHD5 | 1-acylglycerol-3-phosphate O-acyltransferase ABHD5 | Q8WTS1 | ABHD5 |  |  | X |  |
| 22 | 11354-21 | Phospholipase C-beta-1 | 1-phosphatidylinositol 4,5-bisphosphate phosphodiesterase beta-1 | Q9NQ66 | PLCB1 |  |  |  |  |
| 23 | 25949-3 | PLCD3 | 1-phosphatidylinositol 4,5-bisphosphate phosphodiesterase delta-3 | Q8N3E9 | PLCD3 |  |  |  |  |
| 24 | 4563-61 | PLCG1 | 1-phosphatidylinositol 4,5-bisphosphate phosphodiesterase gamma-1 | P19174 | PLCG1 |  | X |  | X |
| 25 | 10070-22 | PLCG2 | 1-phosphatidylinositol 4,5-bisphosphate phosphodiesterase gamma-2 | P16885 | PLCG2 |  | X |  |  |
| 26 | 24909-40 | Phospholipase C-delta-1 | 1-phosphatidylinositol-4,5-bisphosphate phosphodiesterase delta-1 | P51178 | PLCD1 |  |  |  |  |
| 27 | 23670-11 | DPH2 | 2-(3-amino-3-carboxypropyl)histidine synthase subunit 2 | Q9BQC3 | DPH2 |  |  |  |  |
| 28 | 6609-22 | CN37 | 2',3'-cyclic-nucleotide 3'-phosphodiesterase | P09543 | CNP |  |  |  |  |
| 29 | 12687-2 | DECR | 2,4-dienoyl-CoA reductase, mitochondrial | Q16698 | DECR1 | X |  | X |  |
| 30 | 10361-25 | OAS1 | 2'-5'-oligoadenylate synthase 1 | P00973 | OAS1 |  | X |  |  |
| 31 | 25941-4 | OA synthetase | 2'-5'-oligoadenylate synthetase like protein | Q15646 | OASL |  | X |  |  |
| 32 | 23665-35 | PRS6A | 26S protease regulatory subunit 6A | P17980 | PSMC3 |  | X |  |  |
| 33 | 25456-73 | PRS8 | 26S protease regulatory subunit 8 | P62195 | PSMC5 |  | X |  |  |
| 34 | 18385-4 | PSD10 | 26S proteasome non-ATPase regulatory subunit 10 | O75832 | PSMD10 |  |  |  |  |
| 35 | 13572-43 | PSD11 | 26S proteasome non-ATPase regulatory subunit 11 | O00231 | PSMD11 |  |  |  |  |
| 36 | 13568-30 | PSMD4 | 26S proteasome non-ATPase regulatory subunit 4 | P55036 | PSMD4 |  |  |  |  |
| 37 | 10716-35 | PSMD5 | 26S proteasome non-ATPase regulatory subunit 5 | Q16401 | PSMD5 |  |  |  |  |
| 38 | 23672-10 | PSMD6 | 26S proteasome non-ATPase regulatory subunit 6 | Q15008 | PSMD6 |  | X | X |  |
| 39 | 3898-5 | PSD7 | 26S proteasome non-ATPase regulatory subunit 7 | P51665 | PSMD7 |  |  |  |  |
| 40 | 13931-22 | PSMD9 | 26S proteasome non-ATPase regulatory subunit 9 | O00233 | PSMD9 |  |  |  |  |
| 41 | 19240-265 | HAP28 | 28 kDa heat- and acid-stable phosphoprotein | Q13442 | PDAP1 |  |  |  |  |
| 42 | 22845-3 | RT14 | 28S ribosomal protein S14, mitochondrial | O60783 | MRPS14 |  |  |  |  |
| 43 | 17785-11 | RCL | 2'-deoxynucleoside 5'-phosphate N-hydrolase 1 | O43598 | DNPH1 |  |  |  |  |
| 44 | 23645-49 | HACL1 | 2-hydroxyacyl-CoA lyase 1 | Q9UJ83 | HACL1 |  |  |  |  |
| 45 | 22112-30 | COQ5 | 2-methoxy-6-polyprenyl-1,4-benzoquinol methylase, mitochondrial | Q5HYK3 | COQ5 |  |  |  |  |
| 46 | 6079-59 | ACPL2 | 2-phosphoxylose phosphatase 1 | Q8TE99 | PXYLP1 |  |  |  |  |
| 47 | 17814-8 | BPNT1 | 3'(2'),5'-bisphosphate nucleotidase 1 | O95861 | BPNT1 |  |  |  |  |
| 48 | 20433-19 | RM01 | 39S ribosomal protein L1, mitochondrial | Q9BYD6 | MRPL1 |  |  |  |  |
| 49 | 22554-101 | RM10 | 39S ribosomal protein L10, mitochondrial | Q7Z7H8 | MRPL10 |  |  |  |  |
| 50 | 23278-13 | 39S ribosomal protein L12 | 39S ribosomal protein L12, mitochondrial | P52815 | MRPL12 |  |  |  |  |
| 51 | 8021-59 | RM14 | 39S ribosomal protein L14, mitochondrial | Q6P1L8 | MRPL14 |  |  |  |  |
| 52 | 19132-1 | RM02 | 39S ribosomal protein L2, mitochondrial | Q5T653 | MRPL2 |  |  |  |  |
| 53 | 8942-2 | RM21 | 39S ribosomal protein L21, mitochondrial | Q7Z2W9 | MRPL21 |  |  |  |  |
| 54 | 21123-1 | RM28 | 39S ribosomal protein L28, mitochondrial | Q13084 | MRPL28 |  |  |  |  |
| 55 | 7982-10 | RM32 | 39S ribosomal protein L32, mitochondrial | Q9BYC8 | MRPL32 |  |  |  |  |
| 56 | 13453-2 | RM33 | 39S ribosomal protein L33, mitochondrial | O75394 | MRPL33 |  |  |  |  |
| 57 | 6933-20 | RM34 | 39S ribosomal protein L34, mitochondrial | Q9BQ48 | MRPL34 |  |  |  |  |
| 58 | 25486-38 | RM38 | 39S ribosomal protein L38, mitochondrial | Q96DV4 | MRPL38 |  |  |  |  |
| 59 | 22557-68 | RM50 | 39S ribosomal protein L50, mitochondrial | Q8N5N7 | MRPL50 |  |  |  |  |
| 60 | 7123-25 | RM52 | 39S ribosomal protein L52, mitochondrial | Q86TS9 | MRPL52 |  |  |  |  |
| 61 | 11135-5 | RM55 | 39S ribosomal protein L55, mitochondrial | Q7Z7F7 | MRPL55 |  |  |  |  |

| # | **Custom Panel (X)** | **SOMAmer SeqID** | **Target Name** | **Human Target or Analyte** | **UniProt ID** | **GeneID** | **Cardiovascular Disease** | **Inflammation and Immune**  **Response** | **Metabolic Disease** | **Oncology** |
| --- | --- | --- | --- | --- | --- | --- | --- | --- | --- | --- |

| 62 | 23271-20 | EBP | 3-beta-hydroxysteroid-Delta(8),Delta(7)-isomerase | Q15125 | EBP |  | | | |
| --- | --- | --- | --- | --- | --- | --- | --- | --- | --- |
| 63 | 5230-99 | HMGR | 3-hydroxy-3-methylglutaryl-coenzyme A reductase | P04035 | HMGCR |  | X | X |  |
| 64 | 4217-49 | ERAB | 3-hydroxyacyl-CoA dehydrogenase type-2 | Q99714 | HSD17B10 |  |  |  |  |
| 65 | 5861-78 | 3HAO | 3-hydroxyanthranilate 3,4-dioxygenase | P46952 | HAAO |  |  |  |  |
| 66 | 17329-2 | BDH2 | 3-hydroxybutyrate dehydrogenase type 2 | Q9BUT1 | BDH2 |  |  |  |  |
| 67 | 4693-72 | 3HIDH | 3-hydroxyisobutyrate dehydrogenase, mitochondrial | P31937 | HIBADH |  |  |  |  |
| 68 | 12396-19 | HIBCH | 3-hydroxyisobutyryl-CoA hydrolase, mitochondrial | Q6NVY1 | HIBCH |  |  | X |  |
| 69 | 17782-23 | THIK | 3-ketoacyl-CoA thiolase, peroxisomal | P09110 | ACAA1 |  |  |  |  |
| 70 | 19483-16 | KDSR | 3-ketodihydrosphingosine reductase | Q06136 | KDSR |  | X |  | X |
| 71 | 8272-22 | DHB7:CD | 3-keto-steroid reductase:Cytoplasmic domain | P56937 | HSD17B7 |  |  |  |  |
| 72 | 9929-16 | DHB7:ECD | 3-keto-steroid reductase:Extracellular domain (partial) | P56937 | HSD17B7 |  |  |  |  |
| 73 | 12686-15 | THTM | 3-mercaptopyruvate sulfurtransferase | P25325 | MPST |  |  |  |  |
| 74 | 18398-1 | AK1D1 | 3-oxo-5-beta-steroid 4-dehydrogenase | P51857 | AKR1D1 |  |  | X |  |
| 75 | 18271-43 | OXSM | 3-oxoacyl-[acyl-carrier-protein] synthase, mitochondrial | Q9NWU1 | OXSM |  |  |  |  |
| 76 | 4460-8 | PDPK1 | 3-phosphoinositide-dependent protein kinase 1 | O15530 | PDPK1 | X | X |  | X |
| 77 | 13011-20 | RS10 | 40S ribosomal protein S10 | P46783 | RPS10 |  |  |  |  |
| 78 | 21829-8 | RS12 | 40S ribosomal protein S12 | P25398 | RPS12 |  |  |  |  |
| 79 | 20964-13 | RS14 | 40S ribosomal protein S14 | P62263 | RPS14 |  |  |  | X |
| 80 | 19166-15 | RS19 | 40S ribosomal protein S19 | P39019 | RPS19 |  |  |  | X |
| 81 | 21643-8 | RS20 | 40S ribosomal protein S20 | P60866 | RPS20 |  |  | X | X |
| 82 | 23243-120 | RS25 | 40S ribosomal protein S25 | P62851 | RPS25 |  |  |  |  |
| 83 | 5026-66 | RS3 | 40S ribosomal protein S3 | P23396 | RPS3 |  |  |  |  |
| 84 | 5484-63 | RS3A | 40S ribosomal protein S3a | P61247 | RPS3A |  |  |  |  |
| 85 | 9758-17 | RS4X | 40S ribosomal protein S4, X isoform | P62701 | RPS4X |  |  |  | X |
| 86 | 19195-85 | RS5 | 40S ribosomal protein S5 | P46782 | RPS5 |  |  |  |  |
| 87 | 3864-5 | RS7 | 40S ribosomal protein S7 | P62081 | RPS7 |  |  |  | X |
| 88 | 19623-26 | 40S ribosomal protein SA | 40S ribosomal protein SA | P08865 | RPSA |  | X |  |  |
| 89 | 10430-31 | 4F2:CD | 4F2 cell-surface antigen heavy chain:Cytoplasmic domain | P08195 | SLC3A2 |  |  |  |  |
| 90 | 7056-16 | 4F2:ECD | 4F2 cell-surface antigen heavy chain:Extracellular domain | P08195 | SLC3A2 |  |  |  |  |
| 91 | 10024-44 | HOGA1 | 4-hydroxy-2-oxoglutarate aldolase, mitochondrial | Q86XE5 | HOGA1 |  |  | X |  |
| 92 | 17509-6 | HPPD | 4-hydroxyphenylpyruvate dioxygenase | P32754 | HPD |  |  | X |  |
| 93 | 12560-9 | NT5C | 5'(3')-deoxyribonucleotidase, cytosolic type | Q8TCD5 | NT5C |  |  |  |  |
| 94 | 17856-23 | NT5M | 5'(3')-deoxyribonucleotidase, mitochondrial | Q9NPB1 | NT5M |  |  |  |  |
| 95 | 21499-17 | COQ7 | 5-demethoxyubiquinone hydroxylase, mitochondrial | Q99807 | COQ7 |  |  |  |  |
| 96 | 14107-1 | MTHFS | 5-formyltetrahydrofolate cyclo-ligase | P49914 | MTHFS |  |  |  |  |
| 97 | 13556-28 | 5HT2A | 5-hydroxytryptamine receptor 2A | P28223 | HTR2A |  |  | X |  |
| 98 | 13561-5 | 5HT6R | 5-hydroxytryptamine receptor 6 | P50406 | HTR6 |  |  |  |  |
| 99 | 13547-5 | 5HT7R | 5-hydroxytryptamine receptor 7 | P34969 | HTR7 |  |  |  |  |
| 100 | 25880-14 | 5-Lipoxygenase | 5-Lipoxygenase | P09917 | ALOX5 | X | X |  | X |
| 101 | 15452-5 | 5'-Nucleotidase | 5'-Nucleotidase | P21589 | NT5E | X |  | X | X |
| 102 | 6993-8 | 5'-Nucleotidase | 5'-Nucleotidase | P21589 | NT5E |  |  |  |  |
| 103 | 25438-288 | NT5D1 | 5'-nucleotidase domain-containing protein 1 | Q5TFE4 | NT5DC1 |  |  |  |  |
| 104 | 9126-171 | NT5D3 | 5'-nucleotidase domain-containing protein 3 | Q86UY8 | NT5DC3 |  |  |  |  |
| 105 | 2682-68 | HSP 60 | 60 kDa heat shock protein, mitochondrial | P10809 | HSPD1 | X | X | X | X |
| 106 | 10949-59 | RLA2 | 60S acidic ribosomal protein P2 | P05387 | RPLP2 |  |  |  | X |
| 107 | 21286-29 | RL11 | 60S ribosomal protein L11 | P62913 | RPL11 |  |  |  |  |
| 108 | 19183-164 | RL12 | 60S ribosomal protein L12 | P30050 | RPL12 |  |  |  |  |
| 109 | 21583-14 | RL26L | 60S ribosomal protein L26-like 1 | Q9UNX3 | RPL26L1 |  |  |  |  |
| 110 | 12478-15 | RL30 | 60S ribosomal protein L30 | P62888 | RPL30 |  |  |  |  |
| 111 | 22553-4 | RL38 | 60S ribosomal protein L38 | P63173 | RPL38 |  |  |  |  |
| 112 | 20999-12 | RL5 | 60S ribosomal protein L5 | P46777 | RPL5 |  | X |  |  |
| 113 | 12564-9 | NIP7 | 60S ribosome subunit biogenesis protein NIP7 homolog | Q9Y221 | NIP7 |  |  |  |  |
| 114 | 22141-59 | F261 | 6-phosphofructo-2-kinase/fructose-2,6-bisphosphatase 1 | P16118 | PFKFB1 |  |  |  |  |
| 115 | 12456-5 | F263 | 6-phosphofructo-2-kinase/fructose-2,6-bisphosphatase 3 | Q16875 | PFKFB3 | X |  | X |  |
| 116 | 25041-11 | F264 | 6-phosphofructo-2-kinase/fructose-2,6-bisphosphatase 4 | Q16877 | PFKFB4 |  |  |  |  |
| 117 | 4187-49 | 6-Phosphogluconate dehydrogenase | 6-phosphogluconate dehydrogenase, decarboxylating | P52209 | PGD |  |  |  |  |
| 118 | 17799-9 | 6PGL | 6-phosphogluconolactonase | O95336 | PGLS |  |  | X |  |
| 119 | 12014-19 | PTPS | 6-pyruvoyl tetrahydrobiopterin synthase | Q03393 | PTS |  |  | X |  |
| 120 | 19206-20 | 8ODP | 7,8-dihydro-8-oxoguanine triphosphatase | P36639 | NUDT1 |  |  |  |  |
| 121 | 11370-20 | INP5E | 72 kDa inositol polyphosphate 5-phosphatase | Q9NRR6 | INPP5E |  |  | X |  |
| 122 | 4160-49 | MMP-2 | 72 kDa type IV collagenase | P08253 | MMP2 | X | X | X | X |
| 123 | 16588-10 | BiP | 78 kDa glucose-regulated protein | P11021 | HSPA5 | X | X | X | X |
| 124 | 19135-5 | Cytosolic 5'-nucleotidase III-like protein | 7-methylguanosine phosphate-specific 5'-nucleotidase | Q969T7 | NT5C3B |  |  |  |  |
| 125 | 3174-2 | ATS1 | A disintegrin and metalloproteinase with thrombospondin motifs 1 | Q9UHI8 | ADAMTS1 |  |  |  | X |
| 126 | 3175-51 | ATS13 | A disintegrin and metalloproteinase with thrombospondin motifs 13 | Q76LX8 | ADAMTS13 | X | X |  |  |
| 127 | 4533-76 | ATS15 | A disintegrin and metalloproteinase with thrombospondin motifs 15 | Q8TE58 | ADAMTS15 |  |  |  | X |
| 128 | 8845-2 | ATS3 | A disintegrin and metalloproteinase with thrombospondin motifs 3 | O15072 | ADAMTS3 |  |  |  |  |
| 129 | 2809-25 | ADAMTS-4 | A disintegrin and metalloproteinase with thrombospondin motifs 4 | O75173 | ADAMTS4 |  |  |  |  |
| 130 | 3168-8 | ADAMTS-5 | A disintegrin and metalloproteinase with thrombospondin motifs 5 | Q9UNA0 | ADAMTS5 |  |  |  |  |
| 131 | 6441-62 | ATS6 | A disintegrin and metalloproteinase with thrombospondin motifs 6 | Q9UKP5 | ADAMTS6 |  |  |  |  |
| 132 | 9794-17 | ADCK4 | AarF domain-containing protein kinase 4 | Q96D53 | COQ8B |  |  |  |  |
| 133 | 3342-76 | ABL2 | Abelson tyrosine-protein kinase 2 | P42684 | ABL2 |  |  |  |  |
| 134 | 5261-13 | ABL2 | Abelson tyrosine-protein kinase 2 | P42684 | ABL2 |  |  |  |  |
| 135 | 23553-1 | ABI3 | ABI gene family member 3 | Q9P2A4 | ABI3 |  |  |  |  |
| 136 | 25889-108 | AIM2 | Absent in melanoma 2 | O14862 | AIM2 |  |  |  |  |

| # | **Custom Panel (X)** | **SOMAmer SeqID** | **Target Name** | **Human Target or Analyte** | **UniProt ID** | **GeneID** | **Cardiovascular Disease** | **Inflammation and Immune**  **Response** | **Metabolic Disease** | **Oncology** |
| --- | --- | --- | --- | --- | --- | --- | --- | --- | --- | --- |

| 137 | 21515-61 | AACS | Acetoacetyl-CoA synthetase | Q86V21 | AACS |  | | | |
| --- | --- | --- | --- | --- | --- | --- | --- | --- | --- |
| 138 | 10980-11 | ACES | Acetylcholinesterase | P22303 | ACHE |  |  | X | X |
| 139 | 15553-22 | ACES | Acetylcholinesterase | P22303 | ACHE |  |  |  |  |
| 140 | 17341-89 | THIC | Acetyl-CoA acetyltransferase, cytosolic | Q9BWD1 | ACAT2 |  |  | X |  |
| 141 | 19197-95 | THIL | Acetyl-CoA acetyltransferase, mitochondrial | P24752 | ACAT1 |  |  | X |  |
| 142 | 25094-9 | ACSA | Acetyl-coenzyme A synthetase, cytoplasmic | Q9NR19 | ACSS2 |  |  |  |  |
| 143 | 5748-20 | Acid ceramidase | Acid ceramidase | Q13510 | ASAH1 | X | X | X |  |
| 144 | 14086-11 | ASM3A | Acid sphingomyelinase-like phosphodiesterase 3a | Q92484 | SMPDL3A |  |  |  |  |
| 145 | 4771-10 | ASM3A | Acid sphingomyelinase-like phosphodiesterase 3a | Q92484 | SMPDL3A |  |  |  |  |
| 146 | 19169-88 | FIBP | Acidic fibroblast growth factor intracellular-binding protein | O43427 | FIBP |  |  |  |  |
| 147 | 13073-14 | AN32A | Acidic leucine-rich nuclear phosphoprotein 32 family member A | P39687 | ANP32A |  |  |  |  |
| 148 | 4194-26 | AN32B | Acidic leucine-rich nuclear phosphoprotein 32 family member B | Q92688 | ANP32B |  |  |  |  |
| 149 | 22398-5 | AN32C | Acidic leucine-rich nuclear phosphoprotein 32 family member C | O43423 | ANP32C |  |  |  |  |
| 150 | 16591-71 | CHIA | Acidic mammalian chitinase | Q9BZP6 | CHIA |  |  |  |  |
| 151 | 6951-26 | ASIC4 | Acid-sensing ion channel 4 | Q96FT7 | ASIC4 |  |  |  |  |
| 152 | 10507-166 | sperm protein 10 | Acrosomal protein SP-10 | P26436 | ACRV1 |  |  |  |  |
| 153 | 15301-24 | sperm protein 10 | Acrosomal protein SP-10 | P26436 | ACRV1 |  |  |  |  |
| 154 | 9889-42 | AF1L1 | Actin filament-associated protein 1-like 1 | Q8TED9 | AFAP1L1 |  |  |  |  |
| 155 | 24445-38 | AF1L2 | Actin filament-associated protein 1-like 2 | Q8N4X5 | AFAP1L2 |  |  |  |  |
| 156 | 13578-98 | ABLM3 | Actin-binding LIM protein 3 | O94929 | ABLIM3 |  |  |  |  |
| 157 | 12689-56 | ARC1B | Actin-related protein 2/3 complex subunit 1B | O15143 | ARPC1B |  |  |  |  |
| 158 | 23376-56 | ARPC2 | Actin-related protein 2/3 complex subunit 2 | O15144 | ARPC2 |  |  |  |  |
| 159 | 13573-5 | p21-ARC | Actin-related protein 2/3 complex subunit 3 | O15145 | ARPC3 |  |  |  |  |
| 160 | 18419-20 | p16-ARC | Actin-related protein 2/3 complex subunit 5 | O15511 | ARPC5 |  |  |  |  |
| 161 | 3758-63 | Activated Protein C | Activated Protein C | P04070 | PROC |  |  | X |  |
| 162 | 3758-68 | Activated Protein C | Activated Protein C | P04070 | PROC |  |  |  |  |
| 163 | 19236-24 | TCP4 | Activated RNA polymerase II transcriptional coactivator p15 | P53999 | SUB1 |  |  |  |  |
| 164 | 10647-18 | ASCC1 | Activating signal cointegrator 1 complex subunit 1 | Q8N9N2 | ASCC1 |  |  |  |  |
| 165 | 24476-18 | ASC-1 complex subunit p100 | Activating signal cointegrator 1 complex subunit 2 | Q9H1I8 | ASCC2 |  |  |  |  |
| 166 | 11633-89 | AHSA1 | Activator of 90 kDa heat shock protein ATPase homolog 1 | O95433 | AHSA1 |  |  |  |  |
| 167 | 8008-28 | HRK | Activator of apoptosis harakiri | O00198 | HRK |  |  |  |  |
| 168 | 21958-4 | ABR | Active breakpoint cluster region-related protein | Q12979 | ABR |  |  |  |  |
| 169 | 19622-7 | Activin A | Activin A | P08476 | INHBA |  |  |  | X |
| 170 | 2748-3 | Activin A | Activin A | P08476 | INHBA |  |  |  |  |
| 171 | 16746-12 | Activin B | Activin B | P09529 | INHBB |  |  |  |  |
| 172 | 2806-49 | Activin RIB | Activin receptor type-1B | P36896 | ACVR1B |  |  |  |  |
| 173 | 19208-8 | Activin RIIA | Activin receptor type-2A | P27037 | ACVR2A |  |  |  |  |
| 174 | 9412-52 | Activin RIIB | Activin receptor type-2B | Q13705 | ACVR2B | X |  |  |  |
| 175 | 21703-31 | Activin RIA | Activin RIA | Q04771 | ACVR1 |  |  | X | X |
| 176 | 21343-3 | ARC | Activity-regulated cytoskeleton-associated protein | Q7LC44 | ARC |  |  |  |  |
| 177 | 17466-72 | ACSF2 | Acyl-CoA synthetase family member 2, mitochondrial | Q96CM8 | ACSF2 |  |  |  |  |
| 178 | 23692-19 | ACSS3 | Acyl-CoA synthetase short-chain family member 3, mitochondrial | Q9H6R3 | ACSS3 |  |  |  |  |
| 179 | 24686-19 | ACBD4 | Acyl-CoA-binding domain-containing protein 4 | Q8NC06 | ACBD4 |  |  |  |  |
| 180 | 10075-75 | ACBD6 | Acyl-CoA-binding domain-containing protein 6 | Q9BR61 | ACBD6 |  |  |  |  |
| 181 | 19341-36 | ACBD6 | Acyl-CoA-binding domain-containing protein 6 | Q9BR61 | ACBD6 |  |  |  |  |
| 182 | 13563-259 | ACBD7 | Acyl-CoA-binding domain-containing protein 7 | Q8N6N7 | ACBD7 |  |  |  |  |
| 183 | 16919-1 | ACBP | Acyl-CoA-binding protein | P07108 | DBI |  |  |  | X |
| 184 | 23601-43 | ACO12 | Acyl-coenzyme A thioesterase 12 | Q8WYK0 | ACOT12 |  |  |  |  |
| 185 | 17675-17 | ACO13 | Acyl-coenzyme A thioesterase 13 | Q9NPJ3 | ACOT13 |  |  |  |  |
| 186 | 17400-71 | ACOT8 | Acyl-coenzyme A thioesterase 8 | O14734 | ACOT8 |  |  |  |  |
| 187 | 23298-148 | THEM4 | Acyl-coenzyme A thioesterase THEM4 | Q5T1C6 | THEM4 |  |  |  |  |
| 188 | 20370-6 | ACYP1 | Acylphosphatase-1 | P07311 | ACYP1 |  |  |  |  |
| 189 | 12812-25 | ACYP2 | Acylphosphatase-2 | P14621 | ACYP2 |  |  |  |  |
| 190 | 17819-30 | FAHD1 | Acylpyruvase FAHD1, mitochondrial | Q6P587 | FAHD1 |  |  |  |  |
| 191 | 21681-10 | ADAM 28 | ADAM 28 | Q9UKQ2 | ADAM28 |  |  |  | X |
| 192 | 21440-9 | ADAM-8 | ADAM 8 | P78325 | ADAM8 |  |  |  | X |
| 193 | 9115-78 | ADEC1 | ADAM DEC1 | O15204 | ADAMDEC1 |  |  |  |  |
| 194 | 16890-37 | ATL1 | ADAMTS-like protein 1 | Q8N6G6 | ADAMTSL1 |  |  |  |  |
| 195 | 6575-79 | ATL1 | ADAMTS-like protein 1 | Q8N6G6 | ADAMTSL1 |  |  |  |  |
| 196 | 6379-62 | ATL2 | ADAMTS-like protein 2 | Q86TH1 | ADAMTSL2 | X |  | X |  |
| 197 | 4976-57 | CRK | Adapter molecule crk | P46108 | CRK | X |  |  |  |
| 198 | 20915-68 | NECP2 | Adaptin ear-binding coat-associated protein 2 | Q9NVZ3 | NECAP2 |  |  |  |  |
| 199 | 10030-8 | MutY homolog | Adenine DNA glycosylase | Q9UIF7 | MUTYH |  |  |  | X |
| 200 | 10088-37 | APT | Adenine phosphoribosyltransferase | P07741 | APRT |  |  | X | X |
| 201 | 19751-21 | ADA | Adenosine deaminase | P00813 | ADA | X | X | X |  |
| 202 | 6077-63 | CECR1 | Adenosine deaminase CECR1 | Q9NZK5 | ADA2 | X | X | X |  |
| 203 | 19207-119 | ADK | Adenosine kinase | P55263 | ADK |  |  |  |  |
| 204 | 21393-62 | SAHH | Adenosylhomocysteinase | P23526 | AHCY |  |  | X |  |
| 205 | 12652-37 | SAHH2 | Adenosylhomocysteinase 2 | O43865 | AHCYL1 |  |  |  |  |
| 206 | 11368-32 | KAD2 | Adenylate kinase 2, mitochondrial | P54819 | AK2 |  | X | X |  |
| 207 | 17432-25 | KAD4 | Adenylate kinase 4, mitochondrial | P27144 | AK4 |  |  |  |  |
| 208 | 5012-67 | Myokinase, human | Adenylate kinase isoenzyme 1 | P00568 | AK1 | X | X |  |  |
| 209 | 13613-23 | Adenylate kinase isoenzyme 5 | Adenylate kinase isoenzyme 5 | Q9Y6K8 | AK5 |  |  |  |  |
| 210 | 5023-23 | PUR8 | Adenylosuccinate lyase | P30566 | ADSL |  |  | X |  |
| 211 | 13998-26 | PURA1 | Adenylosuccinate synthetase isozyme 1 | Q8N142 | ADSS1 |  |  |  |  |

| # | **Custom Panel (X)** | **SOMAmer SeqID** | **Target Name** | **Human Target or Analyte** | **UniProt ID** | **GeneID** | **Cardiovascular Disease** | **Inflammation and Immune**  **Response** | **Metabolic Disease** | **Oncology** |
| --- | --- | --- | --- | --- | --- | --- | --- | --- | --- | --- |

| 212 | 12644-63 | PURA2 | Adenylosuccinate synthetase isozyme 2 | P30520 | ADSS2 |  | | | |
| --- | --- | --- | --- | --- | --- | --- | --- | --- | --- |
| 213 | 12034-28 | CAP 1 | Adenylyl cyclase-associated protein 1 | Q01518 | CAP1 |  |  | X |  |
| 214 | 14229-5 | MOCS3 | Adenylyltransferase and sulfurtransferase MOCS3 | O95396 | MOCS3 |  |  |  |  |
| 215 | 22588-35 | AGRB2 | Adhesion G protein-coupled receptor B2 | O60241 | ADGRB2 |  |  |  |  |
| 216 | 15365-41 | BAI3 | Adhesion G protein-coupled receptor B3 | O60242 | ADGRB3 |  |  |  |  |
| 217 | 4546-27 | EMR2 | Adhesion G protein-coupled receptor E2 | Q9UHX3 | ADGRE2 | X | X |  |  |
| 218 | 6409-57 | GP116 | Adhesion G protein-coupled receptor F5 | Q8IZF2 | ADGRF5 |  |  |  |  |
| 219 | 21546-20 | AGRD1 | Adhesion G-protein coupled receptor D1 | Q6QNK2 | ADGRD1 |  |  |  |  |
| 220 | 11243-90 | GPR110 | Adhesion G-protein coupled receptor F1 | Q5T601 | ADGRF1 |  |  |  |  |
| 221 | 21697-57 | AGRF2 | Adhesion G-protein coupled receptor F2 | Q8IZF7 | ADGRF2 |  |  |  |  |
| 222 | 18893-26 | GPR56 | Adhesion G-protein coupled receptor G1 | Q9Y653 | ADGRG1 |  | X |  |  |
| 223 | 4551-72 | GP114 | Adhesion G-protein coupled receptor G5 | Q8IZF4 | ADGRG5 |  |  |  |  |
| 224 | 10605-22 | APMAP | Adipocyte plasma membrane-associated protein | Q9HDC9 | APMAP |  |  |  |  |
| 225 | 3554-24 | Adiponectin | Adiponectin | Q15848 | ADIPOQ | X | X | X | X |
| 226 | 8100-15 | ADM2 | ADM2 | Q7Z4H4 | ADM2 | X |  |  |  |
| 227 | 6221-1 | ADPGK | ADP-dependent glucokinase | Q9BRR6 | ADPGK |  |  |  |  |
| 228 | 9482-110 | NUDT9 | ADP-ribose pyrophosphatase, mitochondrial | Q9BW91 | NUDT9 |  |  |  |  |
| 229 | 11513-92 | CD38 | ADP-ribosyl cyclase/cyclic ADP-ribose hydrolase 1 | P28907 | CD38 |  |  |  |  |
| 230 | 7174-15 | CD38 | ADP-ribosyl cyclase/cyclic ADP-ribose hydrolase 1 | P28907 | CD38 | X |  |  |  |
| 231 | 4535-50 | BST1 | ADP-ribosyl cyclase/cyclic ADP-ribose hydrolase 2 | Q10588 | BST1 |  |  |  |  |
| 232 | 19239-5 | ARF1 | ADP-ribosylation factor 1 | P84077 | ARF1 |  |  |  | X |
| 233 | 12578-13 | ARF3 | ADP-ribosylation factor 3 | P61204 | ARF3 |  |  |  |  |
| 234 | 18408-26 | ARF4 | ADP-ribosylation factor 4 | P18085 | ARF4 |  |  |  |  |
| 235 | 18409-61 | ARF5 | ADP-ribosylation factor 5 | P84085 | ARF5 |  |  |  |  |
| 236 | 12425-104 | ARF6 | ADP-ribosylation factor 6 | P62330 | ARF6 |  |  |  |  |
| 237 | 11556-19 | ARFG1 | ADP-ribosylation factor GTPase-activating protein 1 | Q8N6T3 | ARFGAP1 |  |  |  |  |
| 238 | 11664-32 | ARFG2 | ADP-ribosylation factor GTPase-activating protein 2 | Q8N6H7 | ARFGAP2 |  |  |  |  |
| 239 | 13594-158 | GGA1 | ADP-ribosylation factor-binding protein GGA1 | Q9UJY5 | GGA1 |  |  |  | X |
| 240 | 11683-19 | GGA3 | ADP-ribosylation factor-binding protein GGA3 | Q9NZ52 | GGA3 |  |  |  |  |
| 241 | 12392-30 | ARL1 | ADP-ribosylation factor-like protein 1 | P40616 | ARL1 |  |  |  |  |
| 242 | 12433-8 | ARL11 | ADP-ribosylation factor-like protein 11 | Q969Q4 | ARL11 |  | X |  |  |
| 243 | 18410-26 | ARL14 | ADP-ribosylation factor-like protein 14 | Q8N4G2 | ARL14 |  |  |  |  |
| 244 | 18411-83 | ARL15 | ADP-ribosylation factor-like protein 15 | Q9NXU5 | ARL15 |  |  |  |  |
| 245 | 12587-65 | ARL2 | ADP-ribosylation factor-like protein 2 | P36404 | ARL2 |  |  |  |  |
| 246 | 18407-36 | AR2BP | ADP-ribosylation factor-like protein 2-binding protein | Q9Y2Y0 | ARL2BP |  |  |  |  |
| 247 | 12571-14 | ARL3 | ADP-ribosylation factor-like protein 3 | P36405 | ARL3 |  |  |  |  |
| 248 | 18413-24 | ARL4D | ADP-ribosylation factor-like protein 4D | P49703 | ARL4D |  |  |  |  |
| 249 | 18414-26 | ARL5A | ADP-ribosylation factor-like protein 5A | Q9Y689 | ARL5A |  |  |  |  |
| 250 | 17404-5 | ARL5B | ADP-ribosylation factor-like protein 5B | Q96KC2 | ARL5B |  |  |  |  |
| 251 | 18415-16 | ARL6 | ADP-ribosylation factor-like protein 6 | Q9H0F7 | ARL6 |  |  | X |  |
| 252 | 24699-20 | AR6P1 | ADP-ribosylation factor-like protein 6-interacting protein 1 | Q15041 | ARL6IP1 |  |  |  |  |
| 253 | 23281-29 | ARL8A | ADP-ribosylation factor-like protein 8A | Q96BM9 | ARL8A |  |  |  |  |
| 254 | 8569-147 | ARL8B | ADP-ribosylation factor-like protein 8B | Q9NVJ2 | ARL8B |  |  |  |  |
| 255 | 18416-3 | ARL9 | ADP-ribosylation factor-like protein 9 | Q6T311 | ARL9 |  |  |  |  |
| 256 | 17761-2 | NUDT5 | ADP-sugar pyrophosphatase | Q9UKK9 | NUDT5 |  |  |  |  |
| 257 | 11231-12 | ADXL | Adrenodoxin-like protein, mitochondrial | Q6P4F2 | FDX2 |  |  | X |  |
| 258 | 14115-34 | Adrenomedullin | Adrenomedullin | P35318 | ADM |  |  |  |  |
| 259 | 7922-5 | Adrenomedullin | Adrenomedullin | P35318 | ADM | X | X |  |  |
| 260 | 12684-5 | ADSV | Adseverin | Q9Y6U3 | SCIN |  |  |  |  |
| 261 | 4125-52 | sRAGE | Advanced glycosylation end product-specific receptor, soluble | Q15109 | AGER | X | X | X |  |
| 262 | 18196-8 | Afamin | Afamin | P43652 | AFM |  |  |  |  |
| 263 | 4763-31 | Afamin | Afamin | P43652 | AFM |  |  |  |  |
| 264 | 4188-1 | Aflatoxin B1 aldehyde reductase | Aflatoxin B1 aldehyde reductase member 2 | O43488 | AKR7A2 |  |  |  |  |
| 265 | 18173-11 | ARK73 | Aflatoxin B1 aldehyde reductase member 3 | O95154 | AKR7A3 |  |  |  |  |
| 266 | 3280-49 | Aggrecan | Aggrecan core protein | P16112 | ACAN |  | X |  |  |
| 267 | 2813-11 | ART | Agouti-related protein | O00253 | AGRP | X |  | X |  |
| 268 | 5676-54 | ASIP | Agouti-signaling protein | P42127 | ASIP |  |  |  | X |
| 269 | 15483-377 | Agrin | Agrin | O00468 | AGRN |  |  |  |  |
| 270 | 3839-60 | AIP | AH receptor-interacting protein | O00170 | AIP |  |  |  | X |
| 271 | 18399-1 | AKA7A | A-kinase anchor protein 7 isoforms alpha and beta | O43687 | AKAP7 |  |  |  |  |
| 272 | 23280-9 | AKIR2 | Akirin-2 | Q53H80 | AKIRIN2 |  |  |  |  |
| 273 | 16015-19 | ALT | Alanine aminotransferase 1 | P24298 | GPT |  |  |  |  |
| 274 | 3709-4 | ALT | Alanine aminotransferase 1 | P24298 | GPT | X |  |  |  |
| 275 | 21339-19 | ALAT2 | Alanine aminotransferase 2 | Q8TD30 | GPT2 |  |  |  |  |
| 276 | 12340-17 | SYAC | Alanine--tRNA ligase, cytoplasmic | P49588 | AARS1 |  |  |  |  |
| 277 | 21535-5 | AASD1 | Alanyl-tRNA editing protein Aarsd1 | Q9BTE6 | AARSD1 |  |  |  |  |
| 278 | 4192-10 | AK1A1 | Alcohol dehydrogenase [NADP(+)] | P14550 | AKR1A1 |  |  |  |  |
| 279 | 17396-23 | ADH1A | Alcohol dehydrogenase 1A | P07327 | ADH1A |  |  |  |  |
| 280 | 9834-62 | ADH1B | Alcohol dehydrogenase 1B | P00325 | ADH1B | X |  | X | X |
| 281 | 15525-294 | ADH1G | Alcohol dehydrogenase 1C | P00326 | ADH1C |  |  |  |  |
| 282 | 8325-37 | ADH4 | Alcohol dehydrogenase 4 | P08319 | ADH4 |  |  |  |  |
| 283 | 18206-18 | ADH6 | Alcohol dehydrogenase 6 | P28332 | ADH6 |  |  |  |  |
| 284 | 11377-19 | ADH7 | Alcohol dehydrogenase class 4 mu/sigma chain | P40394 | ADH7 |  |  |  | X |
| 285 | 11369-23 | ADHX | Alcohol dehydrogenase class-3 | P11766 | ADH5 |  |  |  |  |
| 286 | 9835-16 | AL1A3 | Aldehyde dehydrogenase family 1 member A3 | P47895 | ALDH1A3 |  |  |  |  |

| # | **Custom Panel (X)** | **SOMAmer SeqID** | **Target Name** | **Human Target or Analyte** | **UniProt ID** | **GeneID** | **Cardiovascular Disease** | **Inflammation and Immune**  **Response** | **Metabolic Disease** | **Oncology** |
| --- | --- | --- | --- | --- | --- | --- | --- | --- | --- | --- |

| 287 | 12940-35 | AL3B1 | Aldehyde dehydrogenase family 3 member B1 | P43353 | ALDH3B1 |  | | | |
| --- | --- | --- | --- | --- | --- | --- | --- | --- | --- |
| 288 | 22057-9 | AL1B1 | Aldehyde dehydrogenase X, mitochondrial | P30837 | ALDH1B1 |  |  | X |  |
| 289 | 11480-1 | Aldehyde dehydrogenase, class 3 | Aldehyde dehydrogenase, dimeric NADP-preferring | P30838 | ALDH3A1 |  | X |  |  |
| 290 | 18381-16 | ALDH-E2 | Aldehyde dehydrogenase, mitochondrial | P05091 | ALDH2 | X | X |  | X |
| 291 | 16081-38 | Aldose reductase-like | Aldo-keto reductase family 1 member B10 | O60218 | AKR1B10 |  |  |  | X |
| 292 | 12618-50 | Aldo-keto reductase 1C1 | Aldo-keto reductase family 1 member C1 | Q04828 | AKR1C1 |  |  |  | X |
| 293 | 21271-53 | AK1C2 | Aldo-keto reductase family 1 member C2 | P52895 | AKR1C2 |  |  | X | X |
| 294 | 17377-1 | Aldose reductase-like C3 | Aldo-keto reductase family 1 member C3 | P42330 | AKR1C3 |  |  | X | X |
| 295 | 18397-5 | AK1C4 | Aldo-keto reductase family 1 member C4 | P17516 | AKR1C4 |  |  |  |  |
| 296 | 16606-85 | Aldose reductase | Aldose reductase | P15121 | AKR1B1 |  |  |  | X |
| 297 | 9854-36 | Aldose reductase | Aldose reductase | P15121 | AKR1B1 |  |  |  |  |
| 298 | 6284-7 | F150B | ALK and LTK ligand 2 | Q6UX46 | ALKAL2 |  |  |  |  |
| 299 | 8249-124 | ALK | ALK tyrosine kinase receptor | Q9UM73 | ALK |  | X |  | X |
| 300 | 7813-6 | Alkaline phosphatase, placental | Alkaline phosphatase, placental type | P05187 | ALPP |  |  |  |  |
| 301 | 6706-18 | PPBN | Alkaline phosphatase, placental-like | P10696 | ALPG |  |  |  |  |
| 302 | 6715-63 | PPBN | Alkaline phosphatase, placental-like | P10696 | ALPG |  |  |  |  |
| 303 | 16926-44 | Alkaline phosphatase, liver | Alkaline phosphatase, tissue-nonspecific isozyme | P05186 | ALPL |  |  | X |  |
| 304 | 11173-29 | MILR1 | Allergin-1 | Q7Z6M3 | MILR1 |  |  |  |  |
| 305 | 2849-49 | AIF1 | Allograft inflammatory factor 1 | P55008 | AIF1 |  |  |  | X |
| 306 | 18871-24 | AIF1L | Allograft inflammatory factor 1-like | Q9BQI0 | AIF1L |  |  |  |  |
| 307 | 11926-23 | P34 | Alpha- and gamma-adaptin-binding protein p34 | Q6PD74 | AAGAB |  |  |  |  |
| 308 | 21716-29 | FUT7 | Alpha-(1,3)-fucosyltransferase | Q11130 | FUT7 |  |  |  |  |
| 309 | 7156-2 | FUT10 | Alpha-(1,3)-fucosyltransferase 10 | Q6P4F1 | FUT10 |  |  |  |  |
| 310 | 21720-13 | FUT11 | Alpha-(1,3)-fucosyltransferase 11 | Q495W5 | FUT11 |  |  |  |  |
| 311 | 4549-78 | FUT5 | Alpha-(1,3)-fucosyltransferase 5 | Q11128 | FUT5 |  |  |  |  |
| 312 | 6991-24 | FUT9 | Alpha-(1,3)-fucosyltransferase 9 | Q9Y231 | FUT9 |  |  |  |  |
| 313 | 8244-16 | FUT8 | Alpha-(1,6)-fucosyltransferase | Q9BYC5 | FUT8 |  |  |  |  |
| 314 | 10571-14 | MGAT1 | Alpha-1,3-mannosyl-glycoprotein 2-beta-N-acetylglucosaminyltransferase | P26572 | MGAT1 |  |  |  |  |
| 315 | 9392-43 | MGT4A | Alpha-1,3-mannosyl-glycoprotein 4-beta-N-acetylglucosaminyltransferase A | Q9UM21 | MGAT4A |  |  |  |  |
| 316 | 7141-21 | MGT4B | Alpha-1,3-mannosyl-glycoprotein 4-beta-N-acetylglucosaminyltransferase B | Q9UQ53 | MGAT4B |  |  |  |  |
| 317 | 7208-60 | MGT4C | Alpha-1,3-mannosyl-glycoprotein 4-beta-N-acetylglucosaminyltransferase C | Q9UBM8 | MGAT4C |  |  |  |  |
| 318 | 23654-6 | ALG2 | Alpha-1,3-mannosyltransferase ALG2 | Q9H553 | ALG2 |  |  | X |  |
| 319 | 10835-25 | A4GCT | Alpha-1,4-N-acetylglucosaminyltransferase | Q9UNA3 | A4GNT |  |  |  |  |
| 320 | 6909-40 | MGAT2 | Alpha-1,6-mannosyl-glycoprotein 2-beta-N-acetylglucosaminyltransferase | Q10469 | MGAT2 |  |  | X |  |
| 321 | 21768-9 | MGT5A | Alpha-1,6-mannosylglycoprotein 6-beta-N-acetylglucosaminyltransferase A | Q09328 | MGAT5 |  | X |  |  |
| 322 | 21813-171 | MGT5A | Alpha-1,6-mannosylglycoprotein 6-beta-N-acetylglucosaminyltransferase A | Q09328 | MGAT5 |  |  |  |  |
| 323 | 2879-9 | a1-Antichymotrypsin | Alpha-1-antichymotrypsin | P01011 | SERPINA3 |  |  |  |  |
| 324 | 4153-11 | alpha-1-antichymotrypsin complex | Alpha-1-antichymotrypsin complex | P01011 | SERPINA3 |  |  | X |  |
| 325 | 3580-25 | a1-Antitrypsin | Alpha-1-antitrypsin | P01009 | SERPINA1 | X | X | X | X |
| 326 | 16561-9 | Alpha-1B-glycoprotein | Alpha-1B-glycoprotein | P04217 | A1BG |  |  |  |  |
| 327 | 15453-3 | a1-Microglobulin | Alpha-1-microglobulin | P02760 | AMBP | X |  |  |  |
| 328 | 19274-80 | Alpha-1-syntrophin | Alpha-1-syntrophin | Q13424 | SNTA1 | X |  |  |  |
| 329 | 22946-55 | Alpha-1-syntrophin | Alpha-1-syntrophin | Q13424 | SNTA1 |  |  |  |  |
| 330 | 7920-30 | SIA8B | Alpha-2,8-sialyltransferase 8B | Q92186 | ST8SIA2 |  |  |  |  |
| 331 | 6930-95 | SIA8F | Alpha-2,8-sialyltransferase 8F | P61647 | ST8SIA6 |  |  |  |  |
| 332 | 3024-18 | a2-Antiplasmin | Alpha-2-antiplasmin | P08697 | SERPINF2 |  |  |  |  |
| 333 | 10966-1 | a2-HS-Glycoprotein | Alpha-2-HS-glycoprotein | P02765 | AHSG |  |  | X |  |
| 334 | 3581-53 | a2-HS-Glycoprotein | Alpha-2-HS-glycoprotein | P02765 | AHSG |  |  |  |  |
| 335 | 3708-62 | a2-Macroglobulin | Alpha-2-macroglobulin | P01023 | A2M |  |  | X | X |
| 336 | 3640-14 | RAP | alpha-2-macroglobulin receptor-associated protein | P30533 | LRPAP1 |  |  |  |  |
| 337 | 15482-12 | A2ML1 | Alpha-2-macroglobulin-like protein 1 | A8K2U0 | A2ML1 | X |  |  |  |
| 338 | 9843-5 | ACTN1 | Alpha-actinin-1 | P12814 | ACTN1 |  |  |  |  |
| 339 | 9844-138 | ACTN2 | Alpha-actinin-2 | P35609 | ACTN2 | X |  |  |  |
| 340 | 25057-2 | ACTN4 | Alpha-actinin-4 | O43707 | ACTN4 |  |  |  | X |
| 341 | 21498-3 | AL7A1 | Alpha-aminoadipic semialdehyde dehydrogenase | P49419 | ALDH7A1 |  |  |  |  |
| 342 | 7918-114 | Amylase, alpha 1A | Alpha-amylase 1 | P04745 | AMY1A |  |  |  |  |
| 343 | 10439-57 | Alpha-amylase 2B | Alpha-amylase 2B | P19961 | AMY2B |  |  |  |  |
| 344 | 15556-49 | Alpha-amylase 2B | Alpha-amylase 2B | P19961 | AMY2B |  |  |  |  |
| 345 | 10087-10 | Alpha crystallin A chain | Alpha-crystallin A chain | P02489 | CRYAA |  | X |  |  |
| 346 | 17734-13 | a-endosulfine | Alpha-endosulfine | O43768 | ENSA | X |  |  |  |
| 347 | 11105-171 | Alpha enolase | Alpha-enolase | P06733 | ENO1 | X | X | X | X |
| 348 | 5792-8 | AFP | alpha-Fetoprotein | P02771 | AFP |  |  | X | X |
| 349 | 15588-17 | galactosidase, alpha | Alpha-galactosidase A | P06280 | GLA | X |  | X |  |
| 350 | 9025-5 | AHSP | Alpha-hemoglobin-stabilizing protein | Q9NZD4 | AHSP |  |  |  |  |
| 351 | 11436-6 | AINX | Alpha-internexin | Q16352 | INA |  |  | X |  |
| 352 | 18401-18 | ALKB3 | Alpha-ketoglutarate-dependent dioxygenase alkB homolog 3 | Q96Q83 | ALKBH3 |  |  |  |  |
| 353 | 21384-2 | Alpha-L-fucosidase I | Alpha-L-fucoside fucohydrolase | P04066 | FUCA1 |  |  | X |  |
| 354 | 3169-70 | IDUA | Alpha-L-iduronidase | P35475 | IDUA | X |  | X |  |
| 355 | 7867-154 | SIA7A | Alpha-N-acetylgalactosaminide alpha-2,6-sialyltransferase 1 | Q9NSC7 | ST6GALNAC1 |  |  |  |  |
| 356 | 7823-22 | SIA7B | Alpha-N-acetylgalactosaminide alpha-2,6-sialyltransferase 2 | Q9UJ37 | ST6GALNAC2 |  | X |  |  |
| 357 | 10626-116 | SIA7C | Alpha-N-acetylgalactosaminide alpha-2,6-sialyltransferase 3 | Q8NDV1 | ST6GALNAC3 |  |  |  |  |
| 358 | 10705-14 | SIA7C | Alpha-N-acetylgalactosaminide alpha-2,6-sialyltransferase 3 | Q8NDV1 | ST6GALNAC3 |  |  |  |  |
| 359 | 7927-16 | SIA7E | Alpha-N-acetylgalactosaminide alpha-2,6-sialyltransferase 5 | Q9BVH7 | ST6GALNAC5 |  | X |  |  |
| 360 | 7228-2 | SIA7F | Alpha-N-acetylgalactosaminide alpha-2,6-sialyltransferase 6 | Q969X2 | ST6GALNAC6 |  |  |  |  |
| 361 | 15509-2 | NAG | Alpha-N-acetylglucosaminidase | P54802 | NAGLU |  |  | X |  |

| # | **Custom Panel (X)** | **SOMAmer SeqID** | **Target Name** | **Human Target or Analyte** | **UniProt ID** | **GeneID** | **Cardiovascular Disease** | **Inflammation and Immune**  **Response** | **Metabolic Disease** | **Oncology** |
| --- | --- | --- | --- | --- | --- | --- | --- | --- | --- | --- |

| 362 | 21508-7 | SIA8A | Alpha-N-acetylneuraminide alpha-2,8-sialyltransferase | Q92185 | ST8SIA1 |  |  |  |  |
| --- | --- | --- | --- | --- | --- | --- | --- | --- | --- |
| 363 | 21663-149 | SIA8A | Alpha-N-acetylneuraminide alpha-2,8-sialyltransferase | Q92185 | ST8SIA1 |  |  |  |
| 364 | 21726-73 | SIA7D | Alpha-N-acetyl-neuraminyl-2,3-beta-galactosyl-1,3-N-acetyl-galactosaminide alpha-2,6- | Q9H4F1 | ST6GALNAC4 |  |  |  |
| 365 | 13434-172 | PARVA | Alpha-parvin | Q9NVD7 | PARVA |  |  |  |
| 366 | 4292-5 | SNAA | Alpha-soluble NSF attachment protein | P54920 | NAPA |  |  |  |
| 367 | 8458-111 | a-Synuclein | Alpha-synuclein | P37840 | SNCA |  |  |  |
| 368 | 8458-16 | a-Synuclein | Alpha-synuclein | P37840 | SNCA |  |  | X |
| 369 | 15698-6 | TXLNA | Alpha-taxilin | P40222 | TXLNA |  |  |  |
| 370 | 9065-28 | TXLNA | Alpha-taxilin | P40222 | TXLNA |  |  |  |
| 371 | 24687-18 | TTPAL | Alpha-tocopherol transfer protein-like | Q9BTX7 | TTPAL |  |  |  |
| 372 | 15486-126 | ABP1 | Amiloride-sensitive amine oxidase [copper-containing] | P19801 | AOC1 | X |  |  |
| 373 | 6209-2 | ABP1 | Amiloride-sensitive amine oxidase [copper-containing] | P19801 | AOC1 |  |  |  |
| 374 | 3343-1 | Aminoacylase-1 | Aminoacylase-1 | Q03154 | ACY1 |  |  | X | X |
| 375 | 21240-6 | GCST | Aminomethyltransferase, mitochondrial | P48728 | AMT |  |  | X |  |
| 376 | 12386-11 | AMPB | Aminopeptidase B | Q9H4A4 | RNPEP |  |  |  |  |
| 377 | 15457-14 | Aminopeptidase N | Aminopeptidase N | P15144 | ANPEP | X |  |  |  |
| 378 | 18396-10 | AES | Amino-terminal enhancer of split | Q08117 | TLE5 | X |  |  |  |
| 379 | 18402-1 | AMERL | AMMECR1-like protein | Q6DCA0 | AMMECR1L |  |  |  |  |
| 380 | 18403-25 | AMPD2 | AMP deaminase 2 | Q01433 | AMPD2 |  |  |  |  |
| 381 | 5183-53 | AMPK a1b1g1 | AMP Kinase (alpha1beta1gamma1) | Q13131|Q9Y478|P54619 | PRKAA1|PRKAB1|PRKAG1 |  | X | X | X |
| 382 | 5245-40 | AMPK a2b2g1 | AMP Kinase (alpha2beta2gamma1) | P54646|O43741|P54619 | PRKAA2|PRKAB2|PRKAG1 | X |  | X |  |
| 383 | 24908-19 | AMPH | Amphiphysin | P49418 | AMPH |  |  |  |  |
| 384 | 2970-60 | AREG | Amphiregulin | P15514 | AREG | X | X | X | X |
| 385 | 9979-13 | AMGO1:CD | Amphoterin-induced protein 1:Cytoplasmic domain | Q86WK6 | AMIGO1 |  |  |  |  |
| 386 | 20120-101 | AMGO1:ECD | Amphoterin-induced protein 1:Extracellular domain | Q86WK6 | AMIGO1 |  |  |  |  |
| 387 | 14134-49 | AMGO2 | Amphoterin-induced protein 2 | Q86SJ2 | AMIGO2 |  |  |  |  |
| 388 | 6914-15 | AMGO2 | Amphoterin-induced protein 2 | Q86SJ2 | AMIGO2 |  |  |  |  |
| 389 | 12401-3 | STALP | AMSH-like protease | Q96FJ0 | STAMBPL1 |  |  |  |  |
| 390 | 12822-34 | APBB1:PID 1 | Amyloid beta A4 precursor protein-binding family B member 1:Phosphotyrosine Interaction | O00213 | APBB1 |  |  |  |  |
| 391 | 14206-28 | APBB1:PID 2 | Amyloid beta A4 precursor protein-binding family B member 1:Phosphotyrosine Interaction | O00213 | APBB1 |  |  |  |  |
| 392 | 21949-4 | AB1IP | Amyloid beta A4 precursor protein-binding family B member 1-interacting protein | Q7Z5R6 | APBB1IP |  |  |  |  |
| 393 | 12753-6 | APBB2:PID 1 | Amyloid beta A4 precursor protein-binding family B member 2:Phosphotyrosine Interaction | Q92870 | APBB2 |  |  |  |  |
| 394 | 12761-12 | APBB2:PID 2 | Amyloid beta A4 precursor protein-binding family B member 2:Phosphotyrosine Interaction | Q92870 | APBB2 |  |  |  |  |
| 395 | 12784-10 | APBB3:PID 1 | Amyloid beta A4 precursor protein-binding family B member 3:Phosphotyrosine Interaction | O95704 | APBB3 |  |  |  |  |
| 396 | 13589-10 | APBB3:PID 2 | Amyloid beta A4 precursor protein-binding family B member 3:Phosphotyrosine Interaction | O95704 | APBB3 |  |  |  |  |
| 397 | 3171-57 | amyloid precursor protein | Amyloid beta A4 protein | P05067 | APP | X |  | X |  |
| 398 | 7210-25 | Amyloid-like protein 1 | Amyloid-like protein 1 | P51693 | APLP1 |  |  |  |  |
| 399 | 10627-87 | APLP2 | Amyloid-like protein 2 | Q06481 | APLP2 | X |  |  |  |
| 400 | 7915-31 | APLP2 | Amyloid-like protein 2 | Q06481 | APLP2 |  |  |  |  |
| 401 | 19173-5 | ZFAN1 | AN1-type zinc finger protein 1 | Q8TCF1 | ZFAND1 |  |  |  |  |
| 402 | 23319-6 | ZFN2B | AN1-type zinc finger protein 2B | Q8WV99 | ZFAND2B |  |  |  |  |
| 403 | 21875-31 | ZFAN3 | AN1-type zinc finger protein 3 | Q9H8U3 | ZFAND3 |  | X | X |  |
| 404 | 18317-111 | ZFAN5 | AN1-type zinc finger protein 5 | O76080 | ZFAND5 |  |  |  |  |
| 405 | 12345-4 | APC10 | Anaphase-promoting complex subunit 10 | Q9UM13 | ANAPC10 |  |  |  |  |
| 406 | 11690-47 | APC7 | Anaphase-promoting complex subunit 7 | Q9UJX3 | ANAPC7 |  |  |  |  |
| 407 | 8051-10 | AGGF1 | Angiogenic factor with G patch and FHA domains 1 | Q8N302 | AGGF1 | X |  |  |  |
| 408 | 4874-3 | Angiogenin | Angiogenin | P03950 | ANG | X |  | X |  |
| 409 | 25296-3 | AMOT | Angiomotin | Q4VCS5 | AMOT |  |  |  | X |
| 410 | 2811-27 | Angiopoietin-1 | Angiopoietin-1 | Q15389 | ANGPT1 | X | X |  |  |
| 411 | 3773-15 | sTie-2 | Angiopoietin-1 receptor, soluble | Q02763 | TEK | X | X |  | X |
| 412 | 13660-76 | Angiopoietin-2 | Angiopoietin-2 | O15123 | ANGPT2 |  |  |  |  |
| 413 | 2602-2 | Angiopoietin-2 | Angiopoietin-2 | O15123 | ANGPT2 |  | X |  |  |
| 414 | 2500-2 | Angiopoietin-4 | Angiopoietin-4 | Q9Y264 | ANGPT4 |  |  |  |  |
| 415 | 7183-102 | TD26 | Angiopoietin-like protein 8 | Q6UXH0 | ANGPTL8 |  |  |  |  |
| 416 | 11142-11 | ANGL1:C-term | Angiopoietin-related protein 1:C-Term, Fibrinogen domian | O95841 | ANGPTL1 |  |  |  |  |
| 417 | 9092-33 | ANGL1:N-term | Angiopoietin-related protein 1:N-term | O95841 | ANGPTL1 |  |  |  |  |
| 418 | 10382-1 | ANGL3 | Angiopoietin-related protein 3 | Q9Y5C1 | ANGPTL3 |  |  |  |  |
| 419 | 10391-1 | ANGL3 | Angiopoietin-related protein 3 | Q9Y5C1 | ANGPTL3 | X |  | X |  |
| 420 | 3796-79 | ANGL4 | Angiopoietin-related protein 4 | Q9BY76 | ANGPTL4 | X |  | X | X |
| 421 | 6371-50 | ANGL7 | Angiopoietin-related protein 7 | O43827 | ANGPTL7 |  |  |  |  |
| 422 | 3710-49 | Angiostatin | Angiostatin | P00747 | PLG | X |  |  |  |
| 423 | 10714-7 | ACE | Angiotensin-converting enzyme | P12821 | ACE | X | X | X | X |
| 424 | 2805-6 | ACE2 | Angiotensin-converting enzyme 2 | Q9BYF1 | ACE2 | X |  |  |  |
| 425 | 3484-60 | Angiotensinogen | Angiotensinogen | P01019 | AGT | X | X | X |  |
| 426 | 13979-3 | S26A7 | Anion exchange transporter | Q8TE54 | SLC26A7 |  |  |  |  |
| 427 | 25437-18 | ANKY2 | Ankyrin repeat and MYND domain-containing protein 2 | Q8IV38 | ANKMY2 |  |  |  |  |
| 428 | 25261-42 | ANKS3 | Ankyrin repeat and SAM domain-containing protein 3 | Q6ZW76 | ANKS3 |  |  |  |  |
| 429 | 21234-7 | ASB-13 | Ankyrin repeat and SOCS box protein 13 | Q8WXK3 | ASB13 |  |  |  |  |
| 430 | 22071-38 | ASB8 | Ankyrin repeat and SOCS box protein 8 | Q9H765 | ASB8 |  |  |  |  |
| 431 | 19601-15 | ASB9 | Ankyrin repeat and SOCS box protein 9 | Q96DX5 | ASB9 |  |  |  |  |
| 432 | 10398-110 | ANKR1 | Ankyrin repeat domain-containing protein 1 | Q15327 | ANKRD1 |  |  |  |  |
| 433 | 15361-37 | ANKR1 | Ankyrin repeat domain-containing protein 1 | Q15327 | ANKRD1 | X |  |  |  |
| 434 | 24720-10 | ANR16 | Ankyrin repeat domain-containing protein 16 | Q6P6B7 | ANKRD16 |  |  |  |  |
| 435 | 25274-2 | ANKR2 | Ankyrin repeat domain-containing protein 2 | Q9GZV1 | ANKRD2 |  |  |  |  |
| 436 | 12445-50 | ANR27 | Ankyrin repeat domain-containing protein 27 | Q96NW4 | ANKRD27 |  |  |  |  |

| # | **Custom Panel (X)** | **SOMAmer SeqID** | **Target Name** | **Human Target or Analyte** | **UniProt ID** | **GeneID** | **Cardiovascular Disease** | **Inflammation and Immune**  **Response** | **Metabolic Disease** | **Oncology** |
| --- | --- | --- | --- | --- | --- | --- | --- | --- | --- | --- |

| 437 | 23367-8 | ANR40 | Ankyrin repeat domain-containing protein 40 | Q6AI12 | ANKRD40 |  |  | | |
| --- | --- | --- | --- | --- | --- | --- | --- | --- | --- |
| 438 | 23413-38 | ANR45 | Ankyrin repeat domain-containing protein 45 | Q5TZF3 | ANKRD45 |  |
| 439 | 7851-30 | ANR46 | Ankyrin repeat domain-containing protein 46 | Q86W74 | ANKRD46 |  |
| 440 | 18404-22 | ANR54 | Ankyrin repeat domain-containing protein 54 | Q6NXT1 | ANKRD54 |  |
| 441 | 23424-4 | ANR63 | Ankyrin repeat domain-containing protein 63 | C9JTQ0 | None |  |
| 442 | 21590-9 | ANRA2 | Ankyrin repeat family A protein 2 | Q9H9E1 | ANKRA2 |  |
| 443 | 7624-19 | ANK2 | Ankyrin-2 | Q01484 | ANK2 | X |
| 444 | 4960-72 | annexin I | Annexin A1 | P04083 | ANXA1 | X |  |  | X |
| 445 | 13605-16 | ANX10 | Annexin A10 | Q9UJ72 | ANXA10 |  |  |  |  |
| 446 | 17513-11 | ANX11 | Annexin A11 | P50995 | ANXA11 |  |  | X |  |
| 447 | 17835-28 | ANX13 | Annexin A13 | P27216 | ANXA13 |  |  |  |  |
| 448 | 13700-10 | annexin II | Annexin A2 | P07355 | ANXA2 |  |  | X | X |
| 449 | 4961-17 | annexin II | Annexin A2 | P07355 | ANXA2 |  |  |  |  |
| 450 | 17163-117 | Annexin III | Annexin A3 | P12429 | ANXA3 |  | X |  |  |
| 451 | 17164-15 | annexin IV | Annexin A4 | P09525 | ANXA4 |  |  |  | X |
| 452 | 14158-17 | Annexin V | Annexin A5 | P08758 | ANXA5 |  |  | X | X |
| 453 | 5335-73 | annexin VI | Annexin A6 | P08133 | ANXA6 |  |  |  |  |
| 454 | 14203-3 | ANXA7 | Annexin A7 | P20073 | ANXA7 |  |  |  |  |
| 455 | 18244-1 | ANXA7 | Annexin A7 | P20073 | ANXA7 |  |  |  |  |
| 456 | 18290-6 | Annexin A8 | Annexin A8 | P13928 | ANXA8 |  |  |  |  |
| 457 | 13588-11 | ANXA9 | Annexin A9 | O76027 | ANXA9 |  |  |  |  |
| 458 | 6603-18 | KALM | Anosmin-1 | P23352 | ANOS1 |  |  |  |  |
| 459 | 4959-2 | AGR2 | Anterior gradient protein 2 homolog | O95994 | AGR2 |  |  |  | X |
| 460 | 17342-13 | AGR3 | Anterior gradient protein 3 | Q8TD06 | AGR3 |  |  |  |  |
| 461 | 5668-49 | AGR3 | Anterior gradient protein 3 | Q8TD06 | AGR3 |  |  |  |  |
| 462 | 10464-6 | ANTR1 | Anthrax toxin receptor 1 | Q9H6X2 | ANTXR1 |  |  |  |  |
| 463 | 15559-5 | ANTR2 | Anthrax toxin receptor 2 | P58335 | ANTXR2 |  |  | X | X |
| 464 | 15481-45 | LL-37 | Antibacterial protein LL-37 | P49913 | CAMP |  |  |  |  |
| 465 | 8749-194 | CD1D | Antigen-presenting glycoprotein CD1d | P15813 | CD1D |  |  |  |  |
| 466 | 4413-3 | SLPI | Antileukoproteinase | P03973 | SLPI | X |  |  |  |
| 467 | 3344-60 | Antithrombin III | Antithrombin-III | P01008 | SERPINC1 | X |  |  |  |
| 468 | 25282-6 | AZIN1 | Antizyme inhibitor 1 | O14977 | AZIN1 |  |  |  |  |
| 469 | 24954-83 | AP1B1 | AP-1 complex subunit beta-1 | Q10567 | AP1B1 |  |  |  |  |
| 470 | 12714-38 | AP1G2 | AP-1 complex subunit gamma-like 2 | O75843 | AP1G2 |  |  |  |  |
| 471 | 18405-117 | AP1S2 | AP-1 complex subunit sigma-2 | P56377 | AP1S2 |  |  |  |  |
| 472 | 20445-29 | AP1AR | AP-1 complex-associated regulatory protein | Q63HQ0 | AP1AR |  |  |  |  |
| 473 | 13621-31 | AP2A2 | AP-2 complex subunit alpha-2 | O94973 | AP2A2 |  |  |  |  |
| 474 | 24938-7 | AP2B1 | AP-2 complex subunit beta-1 | P63010 | AP2B1 |  |  |  |  |
| 475 | 7947-19 | AP4AT | AP-4 complex accessory subunit tepsin | Q96N21 | TEPSIN |  |  |  |  |
| 476 | 10076-1 | AP4M1 | AP-4 complex subunit mu-1 | O00189 | AP4M1 |  |  |  |  |
| 477 | 6622-90 | APEL | Apelin | Q9ULZ1 | APLN | X |  |  |  |
| 478 | 12423-38 | A1CF | APOBEC1 complementation factor | Q9NQ94 | A1CF |  |  |  |  |
| 479 | 2750-3 | Apo A-I | Apolipoprotein A-I | P02647 | APOA1 | X | X | X | X |
| 480 | 7127-3 | Apo A-II | Apolipoprotein A-II | P02652 | APOA2 |  |  | X |  |
| 481 | 17685-9 | Apo A-IV | Apolipoprotein A-IV | P06727 | APOA4 | X |  | X |  |
| 482 | 11318-20 | Apo A-V | Apolipoprotein A-V | Q6Q788 | APOA5 |  |  |  |  |
| 483 | 15363-32 | Apo A-V | Apolipoprotein A-V | Q6Q788 | APOA5 | X |  | X |  |
| 484 | 2797-56 | Apo B | Apolipoprotein B | P04114 | APOB | X |  | X |  |
| 485 | 15364-101 | Apo C-I | Apolipoprotein C-I | P02654 | APOC1 | X |  |  |  |
| 486 | 6350-43 | Apo C-II | Apolipoprotein C-II | P02655 | APOC2 |  | X | X |  |
| 487 | 6461-54 | Apo C-III | Apolipoprotein C-III | P02656 | APOC3 | X | X | X |  |
| 488 | 4712-28 | Apo D | Apolipoprotein D | P05090 | APOD |  |  |  |  |
| 489 | 8262-20 | Apo D | Apolipoprotein D | P05090 | APOD |  |  |  |  |
| 490 | 2418-55 | Apo E | Apolipoprotein E | P02649 | APOE | X | X | X | X |
| 491 | 5312-49 | Apo E2 | Apolipoprotein E (isoform E2) | P02649 | APOE | X | X |  | X |
| 492 | 2937-10 | Apo E3 | Apolipoprotein E (isoform E3) | P02649 | APOE |  |  |  |  |
| 493 | 2938-55 | Apo E4 | Apolipoprotein E (isoform E4) | P02649 | APOE |  |  |  |  |
| 494 | 12370-30 | Apo F | Apolipoprotein F | Q13790 | APOF |  |  |  |  |
| 495 | 11510-31 | Apo L1 | Apolipoprotein L1 | O14791 | APOL1 |  |  |  |  |
| 496 | 11510-51 | Apo L1 | Apolipoprotein L1 | O14791 | APOL1 |  |  |  |  |
| 497 | 9506-10 | Apo L1 | Apolipoprotein L1 | O14791 | APOL1 |  | X |  |  |
| 498 | 16823-75 | APOL3 | Apolipoprotein L3 | O95236 | APOL3 |  |  |  |  |
| 499 | 10445-20 | ApoM | Apolipoprotein M | O95445 | APOM |  |  |  |  |
| 500 | 14125-5 | ApoM | Apolipoprotein M | O95445 | APOM |  |  |  |  |
| 501 | 23566-6 | ApoL-II | Apolipoprotein-L2 | Q9BQE5 | APOL2 |  |  |  |  |
| 502 | 25238-6 | B2L14 | Apoptosis facilitator Bcl-2-like protein 14 | Q9BZR8 | BCL2L14 |  |  |  |  |
| 503 | 3412-7 | Bcl-2 | Apoptosis regulator Bcl-2 | P10415 | BCL2 | X | X | X | X |
| 504 | 24671-15 | ASC | Apoptosis-associated speck-like protein containing a CARD | Q9ULZ3 | PYCARD | X |  | X | X |
| 505 | 9522-3 | AIF | Apoptosis-inducing factor 1, mitochondrial | O95831 | AIFM1 |  | X | X |  |
| 506 | 13583-19 | Apaf-1 | Apoptotic protease-activating factor 1 | O14727 | APAF1 |  |  |  | X |
| 507 | 6518-85 | ghrelin | Appetite-regulating hormone | Q9UBU3 | GHRL | X |  | X | X |
| 508 | 8447-11 | ghrelin | Appetite-regulating hormone | Q9UBU3 | GHRL |  |  |  |  |
| 509 | 17736-105 | APTX | Aprataxin | Q7Z2E3 | APTX |  |  |  |  |
| 510 | 11363-58 | AQP4 | Aquaporin-4 | P55087 | AQP4 |  | X |  |  |
| 511 | 12422-143 | LX15B | Arachidonate 15-lipoxygenase B | O15296 | ALOX15B |  |  |  |  |

| # | **Custom Panel (X)** | **SOMAmer SeqID** | **Target Name** | **Human Target or Analyte** | **UniProt ID** | **GeneID** | **Cardiovascular Disease** | **Inflammation and Immune**  **Response** | **Metabolic Disease** | **Oncology** |
| --- | --- | --- | --- | --- | --- | --- | --- | --- | --- | --- |

| 512 | 13488-3 | ARFP1 | Arfaptin-1 | P53367 | ARFIP1 |  |  |  |  |
| --- | --- | --- | --- | --- | --- | --- | --- | --- | --- |
| 513 | 12630-8 | ARFP2 | Arfaptin-2 | P53365 | ARFIP2 |  |  |  |
| 514 | 11681-8 | NUPL | Arf-GAP domain and FG repeat-containing protein 1 | P52594 | AGFG1 |  |  |  |
| 515 | 23597-11 | AGFG2 | Arf-GAP domain and FG repeat-containing protein 2 | O95081 | AGFG2 |  |  |  |
| 516 | 12343-14 | CENB2 | Arf-GAP with coiled-coil, ANK repeat and PH domain-containing protein 2 | Q15057 | ACAP2 |  |  |  |
| 517 | 14291-53 | AGAP2 | Arf-GAP with GTPase, ANK repeat and PH domain-containing protein 2 | Q99490 | AGAP2 |  |  |  |
| 518 | 13960-15 | AGAP3 | Arf-GAP with GTPase, ANK repeat and PH domain-containing protein 3 | Q96P47 | AGAP3 |  |  |  |
| 519 | 13518-5 | ASAP2 | Arf-GAP with SH3 domain, ANK repeat and PH domain-containing protein 2 | O43150 | ASAP2 |  |  |  |
| 520 | 24455-2 | ASAP3 | Arf-GAP with SH3 domain, ANK repeat and PH domain-containing protein 3 | Q8TDY4 | ASAP3 |  |  |  |
| 521 | 5867-60 | ARGI1 | Arginase-1 | P05089 | ARG1 |  | X | X |
| 522 | 17752-24 | Arginase | Arginase-2, mitochondrial | P78540 | ARG2 |  | X |  |
| 523 | 9050-170 | CA063 | Arginine/serine-rich protein 1 | Q9BUV0 | RSRP1 |  |  |  |
| 524 | 22509-9 | NDUF5 | Arginine-hydroxylase NDUFAF5, mitochondrial | Q5TEU4 | NDUFAF5 |  |  | X |
| 525 | 11241-8 | ARLY | Argininosuccinate lyase | P04424 | ASL |  |  | X |
| 526 | 25108-6 | ATE1 | Arginyl-tRNA--protein transferase 1 | O95260 | ATE1 |  |  |  |
| 527 | 9107-59 | ARM10 | Armadillo repeat-containing protein 10 | Q8N2F6 | ARMC10 |  |  |  |
| 528 | 24963-1 | ARMC3 | Armadillo repeat-containing protein 3 | Q5W041 | ARMC3 |  |  |  |
| 529 | 9945-8 | ARMC5:ARM 2 | Armadillo repeat-containing protein 5:Armadillo Repeat Domain 2 | Q96C12 | ARMC5 |  |  |  |
| 530 | 8785-1 | ARMC5:ARM 5 | Armadillo repeat-containing protein 5:Armadillo Repeat Domain 5 | Q96C12 | ARMC5 |  |  |  |
| 531 | 25247-12 | ARMC8 | Armadillo repeat-containing protein 8 | Q8IUR7 | ARMC8 |  |  |  |
| 532 | 3538-26 | dopa decarboxylase | Aromatic-L-amino-acid decarboxylase | P20711 | DDC |  |  | X |
| 533 | 12352-70 | ARRD3 | Arrestin domain-containing protein 3 | Q96B67 | ARRDC3 |  |  |  | X |
| 534 | 23342-4 | ARRD5 | Arrestin domain-containing protein 5 | A6NEK1 | ARRDC5 |  |  |  |  |
| 535 | 18417-3 | AS3MT | Arsenite methyltransferase | Q9HBK9 | AS3MT |  |  |  | X |
| 536 | 2939-10 | Artemin | Artemin | Q5T4W7 | ARTN |  | X |  | X |
| 537 | 21951-32 | ARNT | Aryl hydrocarbon receptor nuclear translocator | P27540 | ARNT |  | X |  | X |
| 538 | 12632-14 | ARY1 | Arylamine N-acetyltransferase 1 | P18440 | NAT1 |  |  |  | X |
| 539 | 23646-5 | AIPL1 | Aryl-hydrocarbon-interacting protein-like 1 | Q9NZN9 | AIPL1 |  |  |  |  |
| 540 | 3583-54 | Arylsulfatase A | Arylsulfatase A | P15289 | ARSA |  |  |  |  |
| 541 | 8790-6 | Arylsulfatase A | Arylsulfatase A | P15289 | ARSA |  |  | X |  |
| 542 | 3172-28 | ARSB | Arylsulfatase B | P15848 | ARSB |  |  | X |  |
| 543 | 8269-327 | ARSK | Arylsulfatase K | Q6UWY0 | ARSK |  |  |  |  |
| 544 | 5452-71 | ASGR1 | Asialoglycoprotein receptor 1 | P07306 | ASGR1 |  |  |  |  |
| 545 | 9474-22 | ASGR2 | Asialoglycoprotein receptor 2 | P07307 | ASGR2 |  |  |  |  |
| 546 | 23694-3 | ASNS | Asparagine synthetase | P08243 | ASNS |  |  | X |  |
| 547 | 21383-37 | SYNC | Asparaginyl-tRNA synthetase, cytoplasmic | O43776 | NARS1 |  |  |  |  |
| 548 | 4912-17 | GOT1 | Aspartate aminotransferase, cytoplasmic | P17174 | GOT1 |  |  |  |  |
| 549 | 18233-10 | GOT2 | Aspartate aminotransferase, mitochondrial | P00505 | GOT2 |  |  |  |  |
| 550 | 23903-3 | GOT2 | Aspartate aminotransferase, mitochondrial | P00505 | GOT2 |  |  | X |  |
| 551 | 12675-14 | SYDC | Aspartate--tRNA ligase, cytoplasmic | P14868 | DARS1 |  |  |  |  |
| 552 | 12395-86 | SYDM | Aspartate--tRNA ligase, mitochondrial | Q6PI48 | DARS2 |  |  | X |  |
| 553 | 18187-16 | ACY2 | Aspartoacylase | P45381 | ASPA |  |  | X |  |
| 554 | 21781-9 | ACY3 | Aspartoacylase-2 | Q96HD9 | ACY3 |  |  |  |  |
| 555 | 25037-4 | DNPEP | Aspartyl aminopeptidase | Q9ULA0 | DNPEP |  |  |  |  |
| 556 | 6998-106 | HAAH | Aspartyl/asparaginyl beta-hydroxylase | Q12797 | ASPH |  |  |  |  |
| 557 | 6451-64 | ASPN | Asporin | Q9BXN1 | ASPN |  |  |  |  |
| 558 | 7993-23 | ASTL | Astacin-like metalloendopeptidase | Q6HA08 | ASTL |  |  |  |  |
| 559 | 18833-76 | PEA15 | Astrocytic phosphoprotein PEA-15 | Q15121 | PEA15 |  |  |  |  |
| 560 | 25481-66 | ATX10 | Ataxin-10 | Q9UBB4 | ATXN10 |  |  |  |  |
| 561 | 22074-35 | Ataxin-2-binding protein 1 | Ataxin-2-binding protein 1 | Q9NWB1 | RBFOX1 |  |  |  |  |
| 562 | 16825-20 | ATX3 | Ataxin-3 | P54252 | ATXN3 |  |  |  |  |
| 563 | 21544-4 | ATLA3 | Atlastin-3 | Q6DD88 | ATL3 |  |  |  |  |
| 564 | 22073-47 | AT5F1 | ATP synthase B chain, mitochondrial | P24539 | ATP5PB |  |  |  |  |
| 565 | 23226-42 | AT5F1 | ATP synthase B chain, mitochondrial | P24539 | ATP5PB |  |  |  |  |
| 566 | 4965-27 | ATP synthase beta chain | ATP synthase subunit beta, mitochondrial | P06576 | ATP5F1B |  |  |  |  |
| 567 | 11539-4 | ATPK | ATP synthase subunit f, mitochondrial | P56134 | ATP5MF |  |  |  |  |
| 568 | 9202-309 | ATPO | ATP synthase subunit O, mitochondrial | P48047 | ATP5PO |  |  |  |  |
| 569 | 7788-1 | CF6 | ATP synthase-coupling factor 6, mitochondrial | P18859 | ATP5PF | X |  |  |  |
| 570 | 13620-10 | ASNA | ATPase ASNA1 | O43681 | GET3 |  |  |  |  |
| 571 | 6625-31 | ATAD1 | ATPase family AAA domain-containing protein 1 | Q8NBU5 | ATAD1 |  |  |  |  |
| 572 | 13043-157 | ATAD2 | ATPase family AAA domain-containing protein 2 | Q6PL18 | ATAD2 |  |  |  |  |
| 573 | 19755-38 | ATIF1 | ATPase inhibitor, mitochondrial | Q9UII2 | ATP5IF1 |  |  |  |  |
| 574 | 12528-40 | WRIP1 | ATPase WRNIP1 | Q96S55 | WRNIP1 |  |  |  |  |
| 575 | 25886-11 | ABCD4 | ATP-binding cassette sub-family D member 4 | O14678 | ABCD4 |  |  | X |  |
| 576 | 23700-42 | ABCF3 | ATP-binding cassette sub-family F member 3 | Q9NUQ8 | ABCF3 |  |  |  |  |
| 577 | 12700-9 | ACLY | ATP-citrate synthase | P53396 | ACLY | X |  | X |  |
| 578 | 17384-110 | K6PF | ATP-dependent 6-phosphofructokinase, muscle type | P08237 | PFKM | X |  | X |  |
| 579 | 17196-5 | ClpP endopeptidase | ATP-dependent Clp protease proteolytic subunit, mitochondrial | Q16740 | CLPP |  |  |  |  |
| 580 | 11431-235 | RECQ1 | ATP-dependent DNA helicase Q1 | P46063 | RECQL |  |  |  | X |
| 581 | 10527-22 | DHX9 | ATP-dependent RNA helicase A | Q08211 | DHX9 |  |  |  |  |
| 582 | 25092-32 | DDX1 | ATP-dependent RNA helicase DDX1 | Q92499 | DDX1 |  |  |  |  |
| 583 | 18813-15 | DD19A | ATP-dependent RNA helicase DDX19A | Q9NUU7 | DDX19A |  |  |  |  |
| 584 | 5460-60 | DEAD-box protein 19B | ATP-dependent RNA helicase DDX19B | Q9UMR2 | DDX19B |  |  |  |  |
| 585 | 13984-23 | DDX25 | ATP-dependent RNA helicase DDX25 | Q9UHL0 | DDX25 |  |  |  |  |
| 586 | 11601-26 | DHX8 | ATP-dependent RNA helicase DHX8 | Q14562 | DHX8 |  |  |  |  |

| # | **Custom Panel (X)** | **SOMAmer SeqID** | **Target Name** | **Human Target or Analyte** | **UniProt ID** | **GeneID** | **Cardiovascular Disease** | **Inflammation and Immune**  **Response** | **Metabolic Disease** | **Oncology** |
| --- | --- | --- | --- | --- | --- | --- | --- | --- | --- | --- |

| 587 | 11190-129 | YMEL1 | ATP-dependent zinc metalloprotease YME1L1 | Q96TA2 | YME1L1 |  | | | |
| --- | --- | --- | --- | --- | --- | --- | --- | --- | --- |
| 588 | 5443-62 | ANP | Atrial natriuretic factor | P01160 | NPPA | X |  |  | X |
| 589 | 9898-161 | ARI1A | AT-rich interactive domain-containing protein 1A | O14497 | ARID1A |  | X |  | X |
| 590 | 3875-62 | ARI3A | AT-rich interactive domain-containing protein 3A | Q99856 | ARID3A |  |  |  |  |
| 591 | 25484-120 | ARI3C | AT-rich interactive domain-containing protein 3C | A6NKF2 | ARID3C |  |  |  |  |
| 592 | 15499-11 | Attractin | Attractin | O75882 | ATRN |  |  |  |  |
| 593 | 6362-6 | AUGN | Augurin | Q9H1Z8 | ECRG4 |  |  |  |  |
| 594 | 3091-70 | Aurora kinase A | Aurora kinase A | O14965 | AURKA |  |  |  | X |
| 595 | 3346-72 | AURKB | Aurora kinase B | Q96GD4 | AURKB |  | X |  |  |
| 596 | 12597-68 | Autophagy protein 5 | Autophagy protein 5 | Q9H1Y0 | ATG5 |  | X |  |  |
| 597 | 17347-80 | CA080 | Axin interactor, dorsalization-associated protein | Q96BJ3 | AIDA |  |  |  |  |
| 598 | 12925-105 | AXIN2 | Axin-2 | Q9Y2T1 | AXIN2 |  |  |  |  |
| 599 | 8429-16 | AXIN2 | Axin-2 | Q9Y2T1 | AXIN2 | X |  |  | X |
| 600 | 20437-9 | IDLC | Axonemal dynein light intermediate polypeptide 1 | O14645 | DNALI1 |  |  |  |  |
| 601 | 14713-46 | Azurocidin | Azurocidin | P20160 | AZU1 |  |  |  |  |
| 602 | 2751-16 | Azurocidin | Azurocidin | P20160 | AZU1 |  |  |  |  |
| 603 | 16308-14 | BTLA | B- and T-lymphocyte attenuator | Q7Z6A9 | BTLA |  |  |  |  |
| 604 | 6294-11 | BAGE2 | B melanoma antigen 2 | Q86Y30 | BAGE2 |  |  |  |  |
| 605 | 6442-6 | BAGE3 | B melanoma antigen 3 | Q86Y29 | BAGE3 |  |  |  |  |
| 606 | 21356-6 | B9D2 | B9 domain-containing protein 2 | Q9BPU9 | B9D2 |  |  |  |  |
| 607 | 4126-22 | BPI | Bactericidal permeability-increasing protein | P17213 | BPI |  |  |  |  |
| 608 | 10046-55 | cIAP-1 | Baculoviral IAP repeat-containing protein 2 | Q13490 | BIRC2 |  | X |  | X |
| 609 | 21464-2 | cIAP-1 | Baculoviral IAP repeat-containing protein 2 | Q13490 | BIRC2 |  |  |  |  |
| 610 | 4973-18 | cIAP-2 | Baculoviral IAP repeat-containing protein 3 | Q13489 | BIRC3 | X | X |  | X |
| 611 | 3472-40 | Survivin | Baculoviral IAP repeat-containing protein 5 | O15392 | BIRC5 |  | X |  | X |
| 612 | 15412-40 | KIAP | Baculoviral IAP repeat-containing protein 7 | Q96CA5 | BIRC7 |  |  |  | X |
| 613 | 15504-39 | Livin B | Baculoviral IAP repeat-containing protein 7 Isoform beta | Q96CA5 | BIRC7 |  |  |  |  |
| 614 | 17195-43 | BAG-1 | BAG family molecular chaperone regulator 1 | Q99933 | BAG1 |  |  |  | X |
| 615 | 19228-11 | BAG-2 | BAG family molecular chaperone regulator 2 | O95816 | BAG2 |  |  |  |  |
| 616 | 10078-5 | BAG3 | BAG family molecular chaperone regulator 3 | O95817 | BAG3 | X | X |  |  |
| 617 | 12844-10 | BAG4 | BAG family molecular chaperone regulator 4 | O95429 | BAG4 |  |  |  |  |
| 618 | 12743-18 | BAG5 | BAG family molecular chaperone regulator 5 | Q9UL15 | BAG5 |  |  |  |  |
| 619 | 13993-20 | E41L1 | Band 4.1-like protein 1 | Q9H4G0 | EPB41L1 |  |  |  |  |
| 620 | 2816-50 | BCAM | Basal Cell Adhesion Molecule | P50895 | BCAM |  |  |  |  |
| 621 | 15626-223 | Perlecan | Basement membrane-specific heparan sulfate proteoglycan core protein | P98160 | HSPG2 | X |  |  |  |
| 622 | 23659-2 | BZW2 | Basic leucine zipper and W2 domain-containing protein 2 | Q9Y6E2 | BZW2 |  |  |  |  |
| 623 | 19227-18 | BATF | Basic leucine zipper transcriptional factor ATF-like | Q16520 | BATF |  |  |  |  |
| 624 | 8858-21 | BATF3 | Basic leucine zipper transcriptional factor ATF-like 3 | Q9NR55 | BATF3 |  | X |  |  |
| 625 | 3585-54 | BASI | Basigin | P35613 | BSG |  |  | X |  |
| 626 | 15674-3 | CD79A | B-cell antigen receptor complex-associated protein alpha chain | P11912 | CD79A |  | X |  |  |
| 627 | 7796-10 | CD79A | B-cell antigen receptor complex-associated protein alpha chain | P11912 | CD79A |  |  |  |  |
| 628 | 6351-55 | CD79B | B-cell antigen receptor complex-associated protein beta chain | P40259 | CD79B |  | X |  |  |
| 629 | 21331-19 | BCL7A | B-cell CLL/lymphoma 7 protein family member A | Q4VC05 | BCL7A |  |  |  |  |
| 630 | 7009-6 | CD72 | B-cell differentiation antigen CD72 | P21854 | CD72 |  |  |  |  |
| 631 | 7009-8 | CD72 | B-cell differentiation antigen CD72 | P21854 | CD72 |  |  |  |  |
| 632 | 19225-11 | BLNK | B-cell linker protein | Q8WV28 | BLNK |  | X |  |  |
| 633 | 13111-79 | BCL6 | B-cell lymphoma 6 protein | P41182 | BCL6 |  | X |  | X |
| 634 | 13640-5 | BCL6 | B-cell lymphoma 6 protein | P41182 | BCL6 |  |  |  |  |
| 635 | 8768-4 | Bcl-10 | B-cell lymphoma/leukemia 10 | O95999 | BCL10 |  | X |  | X |
| 636 | 24486-1 | B-cell CLL 11A | B-cell lymphoma/leukemia 11A | Q9H165 | BCL11A |  |  |  | X |
| 637 | 2891-1 | CD22 | B-cell receptor CD22 | P20273 | CD22 |  |  |  |  |
| 638 | 11570-94 | BAP29 | B-cell receptor-associated protein 29 | Q9UHQ4 | BCAP29 |  |  |  |  |
| 639 | 24485-32 | BAP31 | B-cell receptor-associated protein 31 | P51572 | BCAP31 |  |  |  |  |
| 640 | 7045-4 | BNIP3 | BCL2/adenovirus E1B 19 kDa protein-interacting protein 3 | Q12983 | BNIP3 | X | X |  | X |
| 641 | 7835-2 | BNI3L | BCL2/adenovirus E1B 19 kDa protein-interacting protein 3-like | O60238 | BNIP3L |  |  |  |  |
| 642 | 5870-23 | BAD | Bcl2-associated agonist of cell death | Q92934 | BAD | X |  |  |  |
| 643 | 4423-77 | BCL2-like 1 protein | Bcl-2-like protein 1 | Q07817 | BCL2L1 | X | X | X | X |
| 644 | 7249-307 | B2L10 | Bcl-2-like protein 10 | Q9HD36 | BCL2L10 |  |  |  |  |
| 645 | 7249-37 | B2L10 | Bcl-2-like protein 10 | Q9HD36 | BCL2L10 |  |  |  |  |
| 646 | 17451-13 | BIM | Bcl-2-like protein 11 | O43521 | BCL2L11 |  |  | X |  |
| 647 | 13097-11 | Apoptosis regulator Bcl-W | Bcl-2-like protein 2 | Q92843 | BCL2L2 |  |  |  |  |
| 648 | 7134-14 | Apoptosis regulator Bcl-W | Bcl-2-like protein 2 | Q92843 | BCL2L2 |  |  |  |  |
| 649 | 21528-12 | BMF | Bcl-2-modifying factor | Q96LC9 | BMF |  | X |  |  |
| 650 | 3413-50 | BFL1 | Bcl-2-related protein A1 | Q16548 | BCL2A1 |  | X |  | X |
| 651 | 4866-59 | TrkB | BDNF/NT-3 growth factors receptor | Q16620 | NTRK2 |  |  | X | X |
| 652 | 10010-10 | BECN1 | Beclin-1 | Q14457 | BECN1 |  |  |  |  |
| 653 | 13032-1 | BECN1 | Beclin-1 | Q14457 | BECN1 | X |  |  |  |
| 654 | 20993-13 | BEND6 | BEN domain-containing protein 6 | Q5SZJ8 | BEND6 |  |  |  |  |
| 655 | 21232-39 | BPSA | Benign Prostate specific Antigen | P07288 | KLK3 |  |  |  |  |
| 656 | 10959-125 | BET1L | BET1-like protein | Q9NYM9 | BET1L |  |  |  |  |
| 657 | 7016-12 | GCNT1 | Beta-1,3-galactosyl-O-glycosyl-glycoprotein beta-1,6-N-acetylglucosaminyltransferase | Q02742 | GCNT1 |  |  |  |  |
| 658 | 10842-7 | GCNT4 | Beta-1,3-galactosyl-O-glycosyl-glycoprotein beta-1,6-N-acetylglucosaminyltransferase 4 | Q9P109 | GCNT4 |  |  |  |  |
| 659 | 10854-15 | GCNT4 | Beta-1,3-galactosyl-O-glycosyl-glycoprotein beta-1,6-N-acetylglucosaminyltransferase 4 | Q9P109 | GCNT4 |  |  |  |  |
| 660 | 9541-15 | B3GT1 | Beta-1,3-galactosyltransferase 1 | Q9Y5Z6 | B3GALT1 |  |  |  |  |
| 661 | 11638-42 | B3GT2 | Beta-1,3-galactosyltransferase 2 | O43825 | B3GALT2 |  |  |  |  |

| # | **Custom Panel (X)** | **SOMAmer SeqID** | **Target Name** | **Human Target or Analyte** | **UniProt ID** | **GeneID** | **Cardiovascular Disease** | **Inflammation and Immune**  **Response** | **Metabolic Disease** | **Oncology** |
| --- | --- | --- | --- | --- | --- | --- | --- | --- | --- | --- |

| 662 | 20593-10 | B3GT5 | Beta-1,3-galactosyltransferase 5 | Q9Y2C3 | B3GALT5 |  |  | | |
| --- | --- | --- | --- | --- | --- | --- | --- | --- | --- |
| 663 | 7981-230 | B3GT6 | Beta-1,3-galactosyltransferase 6 | Q96L58 | B3GALT6 | X |
| 664 | 5727-35 | B3GLT | Beta-1,3-glucosyltransferase | Q6Y288 | B3GLCT |  |
| 665 | 5605-77 | MFNG | Beta-1,3-N-acetylglucosaminyltransferase manic fringe | O00587 | MFNG |  |
| 666 | 7203-125 | RFNG | Beta-1,3-N-acetylglucosaminyltransferase radical fringe | Q9Y644 | RFNG |  |  |  | X |
| 667 | 8976-13 | B4GN1 | Beta-1,4 N-acetylgalactosaminyltransferase 1 | Q00973 | B4GALNT1 |  |  |  |  |
| 668 | 13381-49 | B4GT1 | Beta-1,4-galactosyltransferase 1 | P15291 | B4GALT1 |  |  | X |  |
| 669 | 9595-11 | B4GT2 | Beta-1,4-galactosyltransferase 2 | O60909 | B4GALT2 |  |  |  |  |
| 670 | 6921-24 | B4GT3 | Beta-1,4-galactosyltransferase 3 | O60512 | B4GALT3 |  |  |  |  |
| 671 | 10425-3 | B4GT5 | Beta-1,4-galactosyltransferase 5 | O43286 | B4GALT5 |  | X |  |  |
| 672 | 10832-24 | B4GT6 | Beta-1,4-galactosyltransferase 6 | Q9UBX8 | B4GALT6 |  |  |  |  |
| 673 | 7806-33 | B4GT7 | Beta-1,4-galactosyltransferase 7 | Q9UBV7 | B4GALT7 | X |  |  |  |
| 674 | 21771-47 | MGAT3 | Beta-1,4-mannosyl-glycoprotein 4-beta-N-acetylglucosaminyltransferase | Q09327 | MGAT3 |  |  |  |  |
| 675 | 9078-207 | SNTB1 | Beta-1-syntrophin | Q13884 | SNTB1 |  |  |  |  |
| 676 | 8288-27 | b2-Glycoprotein I | Beta-2-glycoprotein 1 | P02749 | APOH |  | X |  |  |
| 677 | 10574-10 | b2-Microglobulin | Beta-2-microglobulin | P61769 | B2M | X | X | X | X |
| 678 | 3485-28 | b2-Microglobulin | Beta-2-microglobulin | P61769 | B2M |  |  |  |  |
| 679 | 24468-36 | ADDB | Beta-adducin | P35612 | ADD2 |  |  |  |  |
| 680 | 3347-9 | BARK1 | beta-adrenergic receptor kinase 1 | P25098 | GRK2 | X | X |  |  |
| 681 | 5456-59 | CNDP1 | Beta-Ala-His dipeptidase | Q96KN2 | CNDP1 |  |  |  |  |
| 682 | 7870-8 | CNDP1 | Beta-Ala-His dipeptidase | Q96KN2 | CNDP1 | X |  | X |  |
| 683 | 12643-4 | ARRB1 | Beta-arrestin-1 | P49407 | ARRB1 |  |  |  |  |
| 684 | 16587-1 | Beta-casein | Beta-casein | P05814 | CSN2 |  |  |  | X |
| 685 | 21600-10 | CNBP1 | Beta-catenin-interacting protein 1 | Q9NSA3 | CTNNBIP1 |  |  |  |  |
| 686 | 13088-397 | BTC | Betacellulin | P35070 | BTC |  |  | X |  |
| 687 | 9242-11 | BTC | Betacellulin | P35070 | BTC |  |  |  |  |
| 688 | 23354-12 | CRBA2 | Beta-crystallin A2 | P53672 | CRYBA2 |  |  |  |  |
| 689 | 17751-68 | CRBB1 | Beta-crystallin B1 | P53674 | CRYBB1 |  |  |  |  |
| 690 | 10000-28 | CRBB2 | Beta-crystallin B2 | P43320 | CRYBB2 |  | X |  |  |
| 691 | 23262-11 | CRBB3 | Beta-crystallin B3 | P26998 | CRYBB3 |  |  |  |  |
| 692 | 19311-15 | CRBS | Beta-crystallin S | P22914 | CRYGS |  |  |  |  |
| 693 | 6629-3 | HBD-1 | Beta-defensin 1 | P60022 | DEFB1 |  |  |  |  |
| 694 | 5679-16 | HBD-3 | Beta-defensin 103 | P81534 | DEFB103A |  |  |  |  |
| 695 | 5763-67 | HBD-4 | Beta-defensin 104 | Q8WTQ1 | DEFB104A |  |  |  |  |
| 696 | 5664-57 | D106A | Beta-defensin 106 | Q8N104 | DEFB106A |  |  |  |  |
| 697 | 6399-52 | D107A | Beta-defensin 107 | Q8IZN7 | DEFB107A |  |  |  |  |
| 698 | 5611-56 | D108B | Beta-defensin 108B | Q8NET1 | DEFB108B |  |  |  |  |
| 699 | 8340-9 | DB110 | Beta-defensin 110 | Q30KQ9 | DEFB110 |  |  |  |  |
| 700 | 5689-1 | DB112 | Beta-defensin 112 | Q30KQ8 | DEFB112 |  |  |  |  |
| 701 | 13374-4 | DB113 | Beta-defensin 113 | Q30KQ7 | DEFB113 |  |  |  |  |
| 702 | 8391-12 | DB115 | Beta-defensin 115 | Q30KQ5 | DEFB115 |  |  |  |  |
| 703 | 11144-10 | DB116 | Beta-defensin 116 | Q30KQ4 | DEFB116 |  |  |  |  |
| 704 | 9306-7 | DB118 | Beta-defensin 118 | Q96PH6 | DEFB118 |  |  |  |  |
| 705 | 8315-5 | DB119 | Beta-defensin 119 | Q8N690 | DEFB119 |  |  |  |  |
| 706 | 5765-53 | DB121 | Beta-defensin 121 | Q5J5C9 | DEFB121 |  |  |  |  |
| 707 | 9486-13 | DB125 | Beta-defensin 125 | Q8N687 | DEFB125 |  |  |  |  |
| 708 | 20543-19 | DB127 | Beta-defensin 127 | Q9H1M4 | DEFB127 |  |  |  |  |
| 709 | 6360-7 | DB128 | Beta-defensin 128 | Q7Z7B8 | DEFB128 |  |  |  |  |
| 710 | 8347-222 | DB129 | Beta-defensin 129 | Q9H1M3 | DEFB129 |  |  |  |  |
| 711 | 9022-49 | DB132 | Beta-defensin 132 | Q7Z7B7 | DEFB132 |  |  |  |  |
| 712 | 6411-58 | DB135 | Beta-defensin 135 | Q30KP9 | DEFB135 |  |  |  |  |
| 713 | 9332-6 | DB136 | Beta-defensin 136 | Q30KP8 | DEFB136 |  |  |  |  |
| 714 | 13397-88 | HBD-2 | Beta-defensin 4A | O15263 | DEFB4A |  | X |  |  |
| 715 | 23176-17 | Beta-dystroglycan | Beta-dystroglycan | Q14118 | DAG1 |  | X |  |  |
| 716 | 2558-51 | b-Endorphin | Beta-endorphin | P01189 | POMC |  |  |  |  |
| 717 | 16616-137 | ENOB | Beta-enolase | P13929 | ENO3 |  |  | X |  |
| 718 | 10554-23 | BGAL | Beta-galactosidase | P16278 | GLB1 |  | X | X |  |
| 719 | 6035-2 | SIAT1 | Beta-galactoside alpha-2,6-sialyltransferase 1 | P15907 | ST6GAL1 | X | X | X |  |
| 720 | 21737-20 | SIAT2 | Beta-galactoside alpha-2,6-sialyltransferase 2 | Q96JF0 | ST6GAL2 |  |  |  |  |
| 721 | 15562-24 | BGLR | Beta-glucuronidase | P08236 | GUSB |  | X | X |  |
| 722 | 25917-12 | Hexosaminidase A | Beta-hexosaminidase alpha chain | P06865 | HEXA |  |  | X | X |
| 723 | 15470-11 | Hexosaminidase B | Beta-hexosaminidase subunit beta | P07686 | HEXB |  |  | X |  |
| 724 | 6075-61 | Hexosaminidase B | Beta-hexosaminidase subunit beta | P07686 | HEXB |  |  |  |  |
| 725 | 19557-3 | KLOTB | Beta-klotho | Q86Z14 | KLB |  |  |  |  |
| 726 | 23520-60 | YE006 | Beta-lactamase-like protein FLJ75971 | Q68D91 | MBLAC2 |  |  |  |  |
| 727 | 6382-17 | MANBA | Beta-mannosidase | O00462 | MANBA |  |  | X |  |
| 728 | 10620-21 | PSP-94 | Beta-microseminoprotein | P08118 | MSMB |  |  |  |  |
| 729 | 5801-72 | b-NGF | beta-nerve growth factor | P01138 | NGF | X | X |  |  |
| 730 | 12655-30 | SNAB | Beta-soluble NSF attachment protein | Q9H115 | NAPB |  |  |  |  |
| 731 | 21549-144 | TECTB | Beta-tectorin | Q96PL2 | TECTB |  |  |  |  |
| 732 | 17165-1 | BTG | Beta-thromboglobulin | P02775 | PPBP |  |  |  |  |
| 733 | 22091-14 | BUP1 | Beta-ureidopropionase | Q9UBR1 | UPB1 |  |  | X |  |
| 734 | 5798-3 | BID | BH3-interacting domain death agonist | P55957 | BID |  |  |  |  |
| 735 | 11158-40 | BICR1 | Bicaudal D-related protein 1 | Q6ZP65 | BICDL1 |  |  |  |  |
| 736 | 14007-22 | PAPS1 | Bifunctional 3'-phosphoadenosine 5'-phosphosulfate synthase 1 | O43252 | PAPSS1 |  |  |  |  |

| # | **Custom Panel (X)** | **SOMAmer SeqID** | **Target Name** | **Human Target or Analyte** | **UniProt ID** | **GeneID** | **Cardiovascular Disease** | **Inflammation and Immune**  **Response** | **Metabolic Disease** | **Oncology** |
| --- | --- | --- | --- | --- | --- | --- | --- | --- | --- | --- |

| 737 | 18322-15 | JMJD6 | Bifunctional arginine demethylase and lysyl-hydroxylase JMJD6 | Q6NYC1 | JMJD6 |  |  |  | X |
| --- | --- | --- | --- | --- | --- | --- | --- | --- | --- |
| 738 | 21207-1 | COASY | Bifunctional coenzyme A synthase | Q13057 | COASY |  |  | X |  |
| 739 | 6927-7 | NDST1 | Bifunctional heparan sulfate N-deacetylase/N-sulfotransferase 1 | P52848 | NDST1 |  |  |  |  |
| 740 | 21371-12 | MINA | Bifunctional lysine-specific demethylase and histidyl-hydroxylase MINA | Q8IUF8 | RIOX2 |  |  |  |  |
| 741 | 18321-38 | MTDC | Bifunctional methylenetetrahydrofolate dehydrogenase/cyclohydrolase, mitochondrial | P13995 | MTHFD2 |  | X |  | X |
| 742 | 13657-2 | PNKP | Bifunctional polynucleotide phosphatase/kinase | Q96T60 | PNKP |  |  | X |  |
| 743 | 23666-35 | PUR9 | Bifunctional purine biosynthesis protein PURH | P31939 | ATIC |  | X | X |  |
| 744 | 21739-7 | GLCNE | Bifunctional UDP-N-acetylglucosamine 2-epimerase/N-acetylmannosamine kinase | Q9Y223 | GNE |  |  | X |  |
| 745 | 13690-26 | BGN | Biglycan | P21810 | BGN |  | X |  |  |
| 746 | 3284-75 | BGN | Biglycan | P21810 | BGN |  |  |  |  |
| 747 | 23153-14 | BIK | BIK | Q13323 | BIK |  |  |  |  |
| 748 | 8946-38 | NR1H4 | Bile acid receptor | Q96RI1 | NR1H4 | X | X |  | X |
| 749 | 9829-91 | SULT 2A1 | Bile salt sulfotransferase | Q06520 | SULT2A1 |  |  |  |  |
| 750 | 9796-4 | CEL | Bile salt-activated lipase | P19835 | CEL |  |  | X |  |
| 751 | 11382-5 | BIEA | Biliverdin reductase A | P53004 | BLVRA |  |  |  |  |
| 752 | 23246-67 | BL1S1 | Biogenesis of lysosome-related organelles complex 1 subunit 1 | P78537 | BLOC1S1 |  |  |  |  |
| 753 | 21184-1 | BL1S2 | Biogenesis of lysosome-related organelles complex 1 subunit 2 | Q6QNY1 | BLOC1S2 |  |  |  |  |
| 754 | 24487-95 | BL1S3 | Biogenesis of lysosome-related organelles complex 1 subunit 3 | Q6QNY0 | BLOC1S3 |  |  | X |  |
| 755 | 19116-1 | MUTED | Biogenesis of lysosome-related organelles complex 1 subunit 5 | Q8TDH9 | BLOC1S5 |  |  |  |  |
| 756 | 21487-20 | BL1S6 | Biogenesis of lysosome-related organelles complex 1 subunit 6 | Q9UL45 | BLOC1S6 |  |  | X |  |
| 757 | 15644-1 | Biotinidase | Biotinidase | P43251 | BTD |  |  |  |  |
| 758 | 9269-7 | Biotinidase | Biotinidase | P43251 | BTD |  |  | X |  |
| 759 | 9826-135 | Fragile histidine triad protein | Bis(5'-adenosyl)-triphosphatase | P49789 | FHIT |  |  |  | X |
| 760 | 16583-8 | AP4A | Bis(5'-nucleosyl)-tetraphosphatase [asymmetrical] | P50583 | NUDT2 |  |  |  | X |
| 761 | 12020-39 | PMGE | Bisphosphoglycerate mutase | P07738 | BPGM |  |  | X |  |
| 762 | 14250-115 | BLMH | Bleomycin hydrolase | Q13867 | BLMH |  |  |  |  |
| 763 | 23327-1 | BORC5 | BLOC-1-related complex subunit 5 | Q969J3 | BORCS5 |  |  |  |  |
| 764 | 6935-123 | BAMBI:CD | BMP and activin membrane-bound inhibitor homolog:Cytoplasmic domain | Q13145 | BAMBI | X |  |  |  |
| 765 | 20514-8 | BAMBI:ECD | BMP and activin membrane-bound inhibitor homolog:Extracellular domain | Q13145 | BAMBI |  |  |  |  |
| 766 | 8811-24 | BAMBI:ECD | BMP and activin membrane-bound inhibitor homolog:Extracellular domain | Q13145 | BAMBI | X |  |  |  |
| 767 | 15368-3 | BMPER | BMP-binding endothelial regulator protein | Q8N8U9 | BMPER |  |  |  |  |
| 768 | 3654-27 | BMPER | BMP-binding endothelial regulator protein | Q8N8U9 | BMPER |  |  |  |  |
| 769 | 15370-5 | BOLA1 | BolA-like protein 1 | Q9Y3E2 | BOLA1 |  |  |  |  |
| 770 | 8404-102 | BolA-like protein 2 | BolA-like protein 2 | Q9H3K6 | BOLA2 |  |  |  |  |
| 771 | 5980-55 | BOLA3 | BolA-like protein 3 | Q53S33 | BOLA3 |  |  | X |  |
| 772 | 10885-36 | CF089 | Bombesin receptor-activated protein C6orf89 | Q6UWU4 | C6orf89 |  |  |  |  |
| 773 | 7258-5 | EMBP | Bone marrow proteoglycan | P13727 | PRG2 |  |  |  |  |
| 774 | 8832-55 | BST-2 | Bone marrow stromal antigen 2 | Q10589 | BST2 |  |  |  |  |
| 775 | 3348-49 | BMP-1 | Bone morphogenetic protein 1 | P13497 | BMP1 |  |  |  |  |
| 776 | 3587-53 | BMP10 | Bone morphogenetic protein 10 | O95393 | BMP10 |  |  |  |  |
| 777 | 11129-66 | BMP15 | Bone morphogenetic protein 15 | O95972 | BMP15 |  |  |  |  |
| 778 | 15666-21 | BMP-2 | Bone morphogenetic protein 2 | P12643 | BMP2 | X | X | X | X |
| 779 | 16748-1 | BMP-3 | Bone morphogenetic protein 3 | P12645 | BMP3 |  |  |  |  |
| 780 | 22948-13 | BMP-3 | Bone morphogenetic protein 3 | P12645 | BMP3 |  |  |  |  |
| 781 | 15667-39 | BMP-4 | Bone morphogenetic protein 4 | P12644 | BMP4 |  | X | X | X |
| 782 | 6326-20 | BMP-4 | Bone morphogenetic protein 4 | P12644 | BMP4 |  |  |  |  |
| 783 | 21883-17 | BMP-5 | Bone morphogenetic protein 5 | P22003 | BMP5 |  |  |  |  |
| 784 | 3736-60 | BMP-6 | Bone morphogenetic protein 6 | P22004 | BMP6 | X | X | X |  |
| 785 | 8459-10 | BMP-6 | Bone morphogenetic protein 6 | P22004 | BMP6 |  |  |  |  |
| 786 | 2972-57 | BMP-7 | Bone morphogenetic protein 7 | P18075 | BMP7 |  |  |  | X |
| 787 | 15668-19 | BMP-8 | Bone morphogenetic protein 8B | P34820 | BMP8B |  |  |  |  |
| 788 | 4859-6 | BMPR1A | Bone morphogenetic protein receptor type-1A | P36894 | BMPR1A |  |  | X | X |
| 789 | 10550-37 | BMP RIB | Bone morphogenetic protein receptor type-1B | O00238 | BMPR1B | X |  |  |  |
| 790 | 4862-63 | BMP RII | Bone morphogenetic protein receptor type-2 | Q13873 | BMPR2 | X |  |  | X |
| 791 | 14023-84 | BSP | Bone sialoprotein 2 | P21815 | IBSP |  |  |  |  |
| 792 | 3415-61 | BSP | Bone sialoprotein 2 | P21815 | IBSP |  |  |  | X |
| 793 | 6473-55 | PLUNC | BPI fold-containing family A member 1 | Q9NP55 | BPIFA1 |  |  |  |  |
| 794 | 16302-11 | SPLC2 | BPI fold-containing family A member 2 | Q96DR5 | BPIFA2 |  |  |  |  |
| 795 | 5695-5 | SPLC2 | BPI fold-containing family A member 2 | Q96DR5 | BPIFA2 |  |  |  |  |
| 796 | 11246-3 | LPLC1 | BPI fold-containing family B member 1 | Q8TDL5 | BPIFB1 |  |  |  |  |
| 797 | 15367-38 | LPLC1 | BPI fold-containing family B member 1 | Q8TDL5 | BPIFB1 |  |  |  |  |
| 798 | 20111-5 | BASP | Brain acid soluble protein 1 | P80723 | BASP1 |  |  |  | X |
| 799 | 3723-1 | BNP-32 | Brain natriuretic peptide 32 | P16860 | NPPB |  |  |  |  |
| 800 | 14047-78 | BDNF | Brain-derived neurotrophic factor | P23560 | BDNF | X |  | X |  |
| 801 | 2421-7 | BDNF | Brain-derived neurotrophic factor | P23560 | BDNF |  |  |  |  |
| 802 | 25472-12 | BEGIN | Brain-enriched guanylate kinase-associated protein | Q9BUH8 | BEGAIN |  |  |  |  |
| 803 | 21711-86 | BAI1 | Brain-specific angiogenesis inhibitor 1 | O14514 | ADGRB1 |  |  |  |  |
| 804 | 19331-18 | BAIP2 | Brain-specific angiogenesis inhibitor 1-associated protein 2 | Q9UQB8 | BAIAP2 |  |  |  |  |
| 805 | 4534-10 | BSSP4 | Brain-specific serine protease 4 | Q9GZN4 | PRSS22 |  |  |  |  |
| 806 | 19365-11 | BCAT2 | Branched-chain-amino-acid aminotransferase, mitochondrial | O15382 | BCAT2 |  |  | X |  |
| 807 | 8784-7 | F175A | BRCA1-A complex subunit Abraxas | Q6UWZ7 | ABRAXAS1 |  |  |  |  |
| 808 | 10876-300 | BRAT1 | BRCA1-associated ATM activator 1 | Q6PJG6 | BRAT1 |  |  |  |  |
| 809 | 13977-28 | BARD1 | BRCA1-associated RING domain protein 1 | Q99728 | BARD1 |  |  | X | X |
| 810 | 18304-19 | BCCIP | BRCA2 and CDKN1A-interacting protein | Q9P287 | BCCIP |  |  |  |  |
| 811 | 12634-79 | BCAR3:Ras-GEF | Breast cancer anti-estrogen resistance protein 3:Guanine Nucleotide Exchange Factor | O75815 | BCAR3 |  |  |  | X |

| # | **Custom Panel (X)** | **SOMAmer SeqID** | **Target Name** | **Human Target or Analyte** | **UniProt ID** | **GeneID** | **Cardiovascular Disease** | **Inflammation and Immune**  **Response** | **Metabolic Disease** | **Oncology** |
| --- | --- | --- | --- | --- | --- | --- | --- | --- | --- | --- |

| 812 | 5262-57 | BCAR3:SH2 | Breast cancer anti-estrogen resistance protein 3:Src Homology domain | O75815 | BCAR3 |  |  |  | X |
| --- | --- | --- | --- | --- | --- | --- | --- | --- | --- |
| 813 | 22086-2 | BRM1L | Breast cancer metastasis-suppressor 1-like protein | Q5PSV4 | BRMS1L |  |  |  |  |
| 814 | 3461-58 | PGCB | Brevican core protein | Q96GW7 | BCAN |  |  |  |  |
| 815 | 6612-90 | CP079 | BRICHOS domain-containing protein 5 | Q6PL45 | BRICD5 |  |  |  |  |
| 816 | 21540-73 | BIN2 | Bridging integrator 2 | Q9UBW5 | BIN2 |  |  |  |  |
| 817 | 24645-2 | BIN3 | Bridging integrator 3 | Q9NQY0 | BIN3 |  |  |  |  |
| 818 | 8830-29 | F175B | BRISC complex subunit Abro1 | Q15018 | ABRAXAS2 |  |  |  |  |
| 819 | 8945-7 | F175B | BRISC complex subunit Abro1 | Q15018 | ABRAXAS2 |  |  |  |  |
| 820 | 24323-43 | BROX | BRO1 domain-containing protein BROX | Q5VW32 | BROX |  |  |  |  |
| 821 | 12748-6 | BRDT | Bromodomain testis-specific protein | Q58F21 | BRDT |  |  |  |  |
| 822 | 11607-15 | BRD1 | Bromodomain-containing protein 1 | O95696 | BRD1 |  |  |  |  |
| 823 | 10074-128 | BRD2 | Bromodomain-containing protein 2 | P25440 | BRD2 |  |  |  | X |
| 824 | 15372-43 | BRD2 | Bromodomain-containing protein 2 | P25440 | BRD2 |  |  |  |  |
| 825 | 10043-31 | BRD4 | Bromodomain-containing protein 4 | O60885 | BRD4 | X |  |  | X |
| 826 | 11121-56 | VWC2 | Brorin | Q2TAL6 | VWC2 |  |  |  |  |
| 827 | 15308-108 | VWC2 | Brorin | Q2TAL6 | VWC2 |  |  |  |  |
| 828 | 4328-2 | BOC | Brother of CDO | Q9BWV1 | BOC | X |  |  | X |
| 829 | 22080-22 | BACD1 | BTB/POZ domain-containing adapter for CUL3-mediated RhoA degradation protein 1 | Q8WZ19 | KCTD13 |  |  |  |  |
| 830 | 23152-49 | BACD2 | BTB/POZ domain-containing adapter for CUL3-mediated RhoA degradation protein 2 | Q13829 | TNFAIP1 |  |  |  |  |
| 831 | 23337-54 | BACD3 | BTB/POZ domain-containing adapter for CUL3-mediated RhoA degradation protein 3 | Q9H3F6 | KCTD10 |  |  |  |  |
| 832 | 23308-31 | KCTD1 | BTB/POZ domain-containing protein KCTD1 | Q719H9 | KCTD1 |  |  |  |  |
| 833 | 23263-9 | KCD15 | BTB/POZ domain-containing protein KCTD15 | Q96SI1 | KCTD15 |  |  | X |  |
| 834 | 23384-19 | KCD17 | BTB/POZ domain-containing protein KCTD17 | Q8N5Z5 | KCTD17 |  |  |  |  |
| 835 | 23285-51 | KCTD2 | BTB/POZ domain-containing protein KCTD2 | Q14681 | KCTD2 |  |  |  |  |
| 836 | 24440-11 | KCTD3 | BTB/POZ domain-containing protein KCTD3 | Q9Y597 | KCTD3 |  |  |  |  |
| 837 | 21588-4 | KCTD4 | BTB/POZ domain-containing protein KCTD4 | Q8WVF5 | KCTD4 |  |  |  |  |
| 838 | 12473-48 | KCTD5 | BTB/POZ domain-containing protein KCTD5 | Q9NXV2 | KCTD5 |  |  |  |  |
| 839 | 23161-3 | KCTD6 | BTB/POZ domain-containing protein KCTD6 | Q8NC69 | KCTD6 |  |  |  |  |
| 840 | 23267-5 | KCTD6 | BTB/POZ domain-containing protein KCTD6 | Q8NC69 | KCTD6 |  |  |  |  |
| 841 | 22485-1 | KCTD7 | BTB/POZ domain-containing protein KCTD7 | Q96MP8 | KCTD7 |  |  | X |  |
| 842 | 22857-5 | KCTD7 | BTB/POZ domain-containing protein KCTD7 | Q96MP8 | KCTD7 |  |  |  |  |
| 843 | 21674-132 | Butyrophilin 1A1 | Butyrophilin subfamily 1 member A1 | Q13410 | BTN1A1 |  |  |  |  |
| 844 | 8869-5 | BT2A1 | Butyrophilin subfamily 2 member A1 | Q7KYR7 | BTN2A1 |  |  |  |  |
| 845 | 22586-24 | BT2A2 | Butyrophilin subfamily 2 member A2 | Q8WVV5 | BTN2A2 |  |  |  |  |
| 846 | 7081-2 | BT3A1 | Butyrophilin subfamily 3 member A1 | O00481 | BTN3A1 |  |  |  |  |
| 847 | 21433-3 | BT3A2 | Butyrophilin subfamily 3 member A2 | P78410 | BTN3A2 |  | X |  | X |
| 848 | 17692-2 | BT3A3 | Butyrophilin subfamily 3 member A3 | O00478 | BTN3A3 |  |  |  |  |
| 849 | 22950-6 | BT3A3 | Butyrophilin subfamily 3 member A3 | O00478 | BTN3A3 |  |  |  |  |
| 850 | 10848-137 | BTNL3 | Butyrophilin-like protein 3 | Q6UXE8 | BTNL3 |  |  |  |  |
| 851 | 9026-40 | BTNL8 | Butyrophilin-like protein 8 | Q6UX41 | BTNL8 |  |  |  |  |
| 852 | 7950-142 | BTNL9 | Butyrophilin-like protein 9 | Q6UXG8 | BTNL9 |  |  |  |  |
| 853 | 24647-3 | ABEC2 | C->U-editing enzyme APOBEC-2 | Q9Y235 | APOBEC2 |  |  |  |  |
| 854 | 5735-54 | C1GLC | C1GALT1-specific chaperone 1 | Q96EU7 | C1GALT1C1 |  | X |  |  |
| 855 | 21734-36 | C1GLT/C1GLC Complex | C1GLT/C1GLC Complex | Q9NS00|Q96EU7 | C1GALT1|C1GALT1C1 |  |  |  |  |
| 856 | 6404-20 | C1QRF | C1q-related factor | O75973 | C1QL1 |  |  |  |  |
| 857 | 12575-30 | C1TC | C-1-tetrahydrofolate synthase, cytoplasmic | P11586 | MTHFD1 |  |  |  |  |
| 858 | 4900-8 | C3a | C3a anaphylatoxin | P01024 | C3 |  |  |  |  |
| 859 | 2755-8 | C3adesArg | C3a anaphylatoxin des Arginine | P01024 | C3 |  |  |  |  |
| 860 | 18821-9 | C4a | C4a anaphylatoxin | P0C0L4|P0C0L5 | C4A|C4B |  |  |  |  |
| 861 | 9449-150 | C4b-binding protein alpha chain | C4b-binding protein alpha chain | P04003 | C4BPA |  |  |  |  |
| 862 | 7155-46 | C4b-binding protein beta chain | C4b-binding protein beta chain | P20851 | C4BPB |  |  |  |  |
| 863 | 2851-63 | C5a | C5a anaphylatoxin | P01031 | C5 |  |  |  |  |
| 864 | 14759-149 | Cadherin E | Cadherin-1 | P12830 | CDH1 |  |  |  |  |
| 865 | 18429-10 | Cadherin E | Cadherin-1 | P12830 | CDH1 | X | X | X | X |
| 866 | 2501-51 | Cadherin E | Cadherin-1 | P12830 | CDH1 |  |  |  |  |
| 867 | 20529-7 | CAD10 | Cadherin-10 | Q9Y6N8 | CDH10 |  |  |  |  |
| 868 | 7763-25 | Cadherin-11:CD | Cadherin-11:Cytoplasmic domain | P55287 | CDH11 |  |  |  |  |
| 869 | 16305-10 | Cadherin-11:ECD | Cadherin-11:Extracellular domain | P55287 | CDH11 |  |  |  |  |
| 870 | 9604-14 | Cadherin-11:ECD | Cadherin-11:Extracellular domain | P55287 | CDH11 |  |  |  |  |
| 871 | 8329-166 | Cadherin-12:CD | Cadherin-12:Cytoplasmic domain | P55289 | CDH12 |  |  |  |  |
| 872 | 19388-2 | Cadherin-12:ECD | Cadherin-12:Extracellular domain | P55289 | CDH12 |  |  |  |  |
| 873 | 21693-14 | CAD13 | Cadherin-13 | P55290 | CDH13 |  |  |  | X |
| 874 | 11215-6 | CAD15:CD | Cadherin-15:Cytoplasmic domain | P55291 | CDH15 |  |  |  |  |
| 875 | 5410-53 | CAD15:ECD | Cadherin-15:Extracellular domain | P55291 | CDH15 |  |  |  |  |
| 876 | 16613-3 | CAD17 | Cadherin-17 | Q12864 | CDH17 |  | X |  |  |
| 877 | 3797-1 | Cadherin-2 | Cadherin-2 | P19022 | CDH2 | X |  |  | X |
| 878 | 21679-16 | CAD20 | Cadherin-20 | Q9HBT6 | CDH20 |  |  |  |  |
| 879 | 25272-17 | CAD23 | Cadherin-23 | Q9H251 | CDH23 |  |  |  |  |
| 880 | 2643-57 | P-Cadherin | Cadherin-3 | P22223 | CDH3 |  |  |  |  |
| 881 | 20589-5 | CADH4 | Cadherin-4 | P55283 | CDH4 |  |  |  |  |
| 882 | 2819-23 | Cadherin-5 | Cadherin-5 | P33151 | CDH5 | X |  |  | X |
| 883 | 16312-45 | Cadherin-6 | Cadherin-6 | P55285 | CDH6 |  |  |  |  |
| 884 | 3591-51 | Cadherin-6 | Cadherin-6 | P55285 | CDH6 |  |  |  |  |
| 885 | 7959-34 | CADH7 | Cadherin-7 | Q9ULB5 | CDH7 |  |  |  |  |
| 886 | 16021-30 | Cadherin-8 | Cadherin-8 | P55286 | CDH8 |  |  |  |  |

| # | **Custom Panel (X)** | **SOMAmer SeqID** | **Target Name** | **Human Target or Analyte** | **UniProt ID** | **GeneID** | **Cardiovascular Disease** | **Inflammation and Immune**  **Response** | **Metabolic Disease** | **Oncology** |
| --- | --- | --- | --- | --- | --- | --- | --- | --- | --- | --- |

| 887 | 8372-29 | CDHR1 | Cadherin-related family member 1 | Q96JP9 | CDHR1 |  |  |  |  |
| --- | --- | --- | --- | --- | --- | --- | --- | --- | --- |
| 888 | 8222-49 | CDHR3 | Cadherin-related family member 3 | Q6ZTQ4 | CDHR3 |  | X |  |
| 889 | 9962-1 | MUCDL | Cadherin-related family member 5 | Q9HBB8 | CDHR5 |  |  |  |
| 890 | 9918-23 | Calbindin D28 | Calbindin | P05937 | CALB1 |  |  |  |
| 891 | 4903-72 | Calcineurin | Calcineurin | Q08209|P63098 | PPP3CA|PPP3R1 | X |  | X |
| 892 | 12458-79 | CHP1 | Calcineurin B homologous protein 1 | Q99653 | CHP1 |  |  |  |
| 893 | 12831-21 | TESC | Calcineurin B homologous protein 3 | Q96BS2 | TESC |  |  |  |
| 894 | 15545-13 | Calcineurin B a | Calcineurin subunit B type 1 | P63098 | PPP3R1 | X |  |  |
| 895 | 18866-8 | CANB2 | Calcineurin subunit B type 2 | Q96LZ3 | PPP3R2 |  |  |  |
| 896 | 13465-5 | CCP1 | Calcipressin-1 | P53805 | RCAN1 | X | X |  |
| 897 | 20975-2 | RCAN2 | Calcipressin-2 | Q14206 | RCAN2 |  |  |  |
| 898 | 20442-12 | RCAN3 | Calcipressin-3 | Q9UKA8 | RCAN3 |  |  |  |
| 899 | 17170-15 | CALCB | Calcitonin gene-related peptide 2 | P10092 | CALCB |  |  |  |
| 900 | 20542-47 | CALCR | Calcitonin receptor | P30988 | CALCR |  |  | X |
| 901 | 20423-40 | CIB1 | Calcium and integrin-binding protein 1 | Q99828 | CIB1 |  | X |  |
| 902 | 21140-19 | CAMLG | Calcium signal-modulating cyclophilin ligand | P49069 | CAMLG |  |  |  |
| 903 | 6226-69 | EFHA1 | Calcium uptake protein 2, mitochondrial | Q8IYU8 | MICU2 |  |  |  |
| 904 | 6912-6 | EFHA2 | Calcium uptake protein 3, mitochondrial | Q86XE3 | MICU3 |  |  |  |
| 905 | 5253-1 | PDE1A | Calcium/calmodulin-dependent 3',5'-cyclic nucleotide phosphodiesterase 1A | P54750 | PDE1A |  |  |  |
| 906 | 21667-57 | PDE1B | Calcium/calmodulin-dependent 3',5'-cyclic nucleotide phosphodiesterase 1B | Q01064 | PDE1B |  |  |  |
| 907 | 21281-13 | CK2N2 | Calcium/calmodulin-dependent protein kinase II inhibitor 2 | Q96S95 | CAMK2N2 |  |  |  |
| 908 | 18860-2 | CaMKK alpha | Calcium/calmodulin-dependent protein kinase kinase 1 | Q8N5S9 | CAMKK1 |  |  |  |
| 909 | 3592-4 | CAMK1 | Calcium/calmodulin-dependent protein kinase type 1 | Q14012 | CAMK1 |  |  |  |
| 910 | 3418-12 | CAMK1D | Calcium/calmodulin-dependent protein kinase type 1D | Q8IU85 | CAMK1D |  |  |  |
| 911 | 3350-53 | CAMK2A | Calcium/calmodulin-dependent protein kinase type II subunit alpha | Q9UQM7 | CAMK2A |  |  | X |
| 912 | 3351-1 | CAMK2B | Calcium/calmodulin-dependent protein kinase type II subunit beta | Q13554 | CAMK2B |  |  |  |
| 913 | 3419-49 | CAMK2D | Calcium/calmodulin-dependent protein kinase type II subunit delta | Q13557 | CAMK2D | X |  |  |
| 914 | 18904-23 | KCC4 | Calcium/calmodulin-dependent protein kinase type IV | Q16566 | CAMK4 |  |  |  |
| 915 | 10496-11 | CaCC | Calcium-activated chloride channel regulator 1 | A8K7I4 | CLCA1 |  |  |  |
| 916 | 8950-4 | CLCA2 | Calcium-activated chloride channel regulator 2 | Q9UQC9 | CLCA2 |  |  |  |
| 917 | 8905-20 | KCMB3 | Calcium-activated potassium channel subunit beta-3 | Q9NPA1 | KCNMB3 |  |  |  |
| 918 | 12534-10 | CACO2 | Calcium-binding and coiled-coil domain-containing protein 2 | Q13137 | CALCOCO2 |  |  |  |
| 919 | 24690-1 | CABP2 | Calcium-binding protein 2 | Q9NPB3 | CABP2 |  |  |  |
| 920 | 17757-86 | CAB39 | Calcium-binding protein 39 | Q9Y376 | CAB39 |  |  |  |
| 921 | 21154-8 | CB39L | Calcium-binding protein 39-like | Q9H9S4 | CAB39L |  |  |  |
| 922 | 22445-2 | CABP5 | Calcium-binding protein 5 | Q9NP86 | CABP5 |  |  |  |
| 923 | 7104-71 | CABP7 | Calcium-binding protein 7 | Q86V35 | CABP7 |  |  |  |
| 924 | 10933-107 | CABP8 | Calcium-binding protein 8 | Q9BXU9 | CALN1 |  |  |  |
| 925 | 2449-1 | GV | Calcium-dependent phospholipase A2 | P39877 | PLA2G5 |  |  |  |
| 926 | 25099-4 | CAPS2 | Calcium-dependent secretion activator 2 | Q86UW7 | CADPS2 |  |  |  |
| 927 | 12808-103 | CHSP1 | Calcium-regulated heat-stable protein 1 | Q9Y2V2 | CARHSP1 |  |  |  |
| 928 | 12432-23 | CYBP | Calcyclin-binding protein | Q9HB71 | CACYBP |  |  |  |
| 929 | 21143-10 | Calcyphosin | Calcyphosin | Q13938 | CAPS |  |  |  |
| 930 | 13688-2 | CAPSL | Calcyphosin-like protein | Q8WWF8 | CAPSL |  |  |  |
| 931 | 25273-80 | Caldesmon | Caldesmon | Q05682 | CALD1 |  | X |  |
| 932 | 24217-2 | Calgranulin A | Calgranulin A | P05109 | S100A8 |  | X |  | X |
| 933 | 25216-8 | Calgranulin A | Calgranulin A | P05109 | S100A8 |  |  |  |  |
| 934 | 10600-24 | CLGN | Calmegin | O14967 | CLGN |  |  |  |  |
| 935 | 8834-58 | Calnexin | Calnexin | P27824 | CANX | X |  |  |  |
| 936 | 2668-70 | Calpain I | Calpain I | P07384|P04632 | CAPN1|CAPNS1 |  |  |  | X |
| 937 | 18299-13 | CPNS1 | Calpain small subunit 1 | P04632 | CAPNS1 |  |  |  |  |
| 938 | 22063-20 | CPNS2 | Calpain small subunit 2 | Q96L46 | CAPNS2 |  |  |  |  |
| 939 | 24915-123 | CAN13 | Calpain-13 | Q6MZZ7 | CAPN13 |  |  |  |  |
| 940 | 14684-17 | CAN2 | Calpain-2 catalytic subunit | P17655 | CAPN2 |  |  |  |  |
| 941 | 12385-4 | CAN3 | Calpain-3 | P20807 | CAPN3 |  |  |  |  |
| 942 | 20173-39 | CAN9 | Calpain-9 | O14815 | CAPN9 |  |  |  |  |
| 943 | 3026-5 | Calpastatin | Calpastatin | P20810 | CAST |  |  |  |  |
| 944 | 15566-10 | Calponin-1 | Calponin-1 | P51911 | CNN1 |  |  |  |  |
| 945 | 18877-15 | CNN2 | Calponin-2 | Q99439 | CNN2 |  |  |  | X |
| 946 | 5264-65 | calreticulin | Calreticulin | P27797 | CALR | X |  |  | X |
| 947 | 16803-4 | CALB2 | Calretinin | P22676 | CALB2 |  |  | X |  |
| 948 | 10513-13 | CSEN | Calsenilin | Q9Y2W7 | KCNIP3 |  |  |  |  |
| 949 | 11263-57 | CASQ1 | Calsequestrin-1 | P31415 | CASQ1 |  |  |  |  |
| 950 | 19291-2 | CASQ2 | Calsequestrin-2 | O14958 | CASQ2 | X |  |  |  |
| 951 | 15521-4 | Alcadein alpha-1 | Calsyntenin-1 | O94985 | CLSTN1 |  |  |  |  |
| 952 | 18882-7 | CSTN2 | Calsyntenin-2 | Q9H4D0 | CLSTN2 |  |  |  |  |
| 953 | 6291-55 | Alcadein-beta | Calsyntenin-3 | Q9BQT9 | CLSTN3 |  |  |  |  |
| 954 | 20128-1 | CREB1 | cAMP response element-binding protein | P16220 | CREB1 | X |  |  |  |
| 955 | 3466-8 | PRKACA | cAMP-dependent protein kinase catalytic subunit alpha | P17612 | PRKACA |  |  |  | X |
| 956 | 21483-155 | KAP0 | cAMP-dependent protein kinase type I-alpha regulatory subunit | P10644 | PRKAR1A | X |  | X | X |
| 957 | 12479-50 | KAP1 | cAMP-dependent protein kinase type I-beta regulatory subunit | P31321 | PRKAR1B |  |  |  |  |
| 958 | 19125-26 | KAP2 | cAMP-dependent protein kinase type II-alpha regulatory subunit | P13861 | PRKAR2A |  |  |  |  |
| 959 | 25463-3 | KAP3 | cAMP-dependent protein kinase type II-beta regulatory subunit | P31323 | PRKAR2B | X |  | X |  |
| 960 | 4963-19 | ARP19 | cAMP-regulated phosphoprotein 19 | P56211 | ARPP19 |  |  |  |  |
| 961 | 12860-7 | cAMP-regulated phosphoprotein 21 | cAMP-regulated phosphoprotein 21 | Q9UBL0 | ARPP21 |  |  |  |  |

| # | **Custom Panel (X)** | **SOMAmer SeqID** | **Target Name** | **Human Target or Analyte** | **UniProt ID** | **GeneID** | **Cardiovascular Disease** | **Inflammation and Immune**  **Response** | **Metabolic Disease** | **Oncology** |
| --- | --- | --- | --- | --- | --- | --- | --- | --- | --- | --- |

| 962 | 22116-9 | CREM | cAMP-responsive element modulator | Q03060 | CREM | X | X |  | |
| --- | --- | --- | --- | --- | --- | --- | --- | --- | --- |
| 963 | 22115-2 | CRBL2 | cAMP-responsive element-binding protein-like 2 | O60519 | CREBL2 |  |  |
| 964 | 18918-86 | PDE4A | cAMP-specific 3',5'-cyclic phosphodiesterase 4A | P27815 | PDE4A |  |  |
| 965 | 20221-26 | PDE4C | cAMP-specific 3',5'-cyclic phosphodiesterase 4C | Q08493 | PDE4C |  |  |
| 966 | 5255-22 | PDE4D | cAMP-specific 3',5'-cyclic phosphodiesterase 4D | Q08499 | PDE4D |  | X |
| 967 | 6931-10 | CTAG1A | Cancer/testis antigen 1 | P78358 | CTAG1A |  |  |
| 968 | 23586-32 | Cancer/testis antigen 1B | Cancer/testis antigen 1B | P78358 | CTAG1A |  |  |
| 969 | 9363-11 | CX048 | Cancer/testis antigen 55 | Q8WUE5 | CT55 |  |  |
| 970 | 24667-27 | CT451 | Cancer/testis antigen family 45 member A1 | Q5HYN5 | CT45A1 |  |  |
| 971 | 24473-11 | CT453 | Cancer/testis antigen family 45 member A3 | Q8NHU0 | CT45A3 |  |  |
| 972 | 24431-32 | CMTR1 | Cap-specific mRNA (nucleoside-2'-O-)-methyltransferase 1 | Q8N1G2 | CMTR1 |  |  |
| 973 | 23620-16 | CPZIP | CapZ-interacting protein | Q6JBY9 | RCSD1 |  |  |
| 974 | 7803-4 | CHST1 | Carbohydrate sulfotransferase 1 | O43916 | CHST1 |  |  |
| 975 | 21721-6 | CHSTA | Carbohydrate sulfotransferase 10 | O43529 | CHST10 |  |  |
| 976 | 7779-86 | CHSTB | Carbohydrate sulfotransferase 11 | Q9NPF2 | CHST11 |  |  |
| 977 | 6626-81 | CHSTC | Carbohydrate sulfotransferase 12 | Q9NRB3 | CHST12 |  |  |
| 978 | 7262-191 | CHSTE | Carbohydrate sulfotransferase 14 | Q8NCH0 | CHST14 | X |  |
| 979 | 14097-86 | ST4S6 | Carbohydrate sulfotransferase 15 | Q7LFX5 | CHST15 |  |  |
| 980 | 4469-78 | ST4S6 | Carbohydrate sulfotransferase 15 | Q7LFX5 | CHST15 |  |  |
| 981 | 18863-176 | CHST2 | Carbohydrate sulfotransferase 2 | Q9Y4C5 | CHST2 |  |  |
| 982 | 4428-1 | CHST2 | Carbohydrate sulfotransferase 2 | Q9Y4C5 | CHST2 |  |  |
| 983 | 7189-55 | CHST3 | Carbohydrate sulfotransferase 3 | Q7LGC8 | CHST3 |  | X |
| 984 | 18876-77 | CHST4 | Carbohydrate sulfotransferase 4 | Q8NCG5 | CHST4 |  |  |
| 985 | 7020-13 | CHST5 | Carbohydrate sulfotransferase 5 | Q9GZS9 | CHST5 |  |  |
| 986 | 4429-51 | CHST6 | Carbohydrate sulfotransferase 6 | Q9GZX3 | CHST6 |  |  |
| 987 | 11646-4 | Carbohydrate sulfotransferase 9 | Carbohydrate sulfotransferase 9 | Q7L1S5 | CHST9 |  |  |
| 988 | 4969-2 | Carbonic anhydrase I | Carbonic anhydrase 1 | P00915 | CA1 | X |  | X | X |
| 989 | 19347-37 | Carbonic Anhydrase XII | Carbonic anhydrase 12 | O43570 | CA12 |  |  |  |  |
| 990 | 3420-21 | Carbonic anhydrase XIII | Carbonic anhydrase 13 | Q8N1Q1 | CA13 |  |  |  |  |
| 991 | 4970-55 | carbonic anhydrase II | Carbonic anhydrase 2 | P00918 | CA2 |  |  | X | X |
| 992 | 3799-11 | Carbonic anhydrase III | Carbonic anhydrase 3 | P07451 | CA3 | X |  | X |  |
| 993 | 14069-61 | Carbonic Anhydrase IV | Carbonic anhydrase 4 | P22748 | CA4 | X |  | X |  |
| 994 | 3177-49 | Carbonic Anhydrase IV | Carbonic anhydrase 4 | P22748 | CA4 |  |  |  |  |
| 995 | 8791-151 | Carbonic Anhydrase VA | Carbonic anhydrase 5A, mitochondrial | P35218 | CA5A |  |  |  |  |
| 996 | 17673-34 | Carbonic Anhydrase VB | Carbonic anhydrase 5B, mitochondrial | Q9Y2D0 | CA5B |  |  |  |  |
| 997 | 13747-9 | Carbonic anhydrase 6 | Carbonic anhydrase 6 | P23280 | CA6 |  |  |  |  |
| 998 | 3352-80 | Carbonic anhydrase 6 | Carbonic anhydrase 6 | P23280 | CA6 |  |  |  |  |
| 999 | 3356-50 | Carbonic anhydrase VII | Carbonic anhydrase 7 | P43166 | CA7 |  |  |  |  |
| 1000 | 3798-71 | Carbonic anhydrase 9 | Carbonic anhydrase 9 | Q16790 | CA9 |  | X |  | X |
| 1001 | 11390-24 | Carbonic Anhydrase VIII | Carbonic anhydrase-related protein | P35219 | CA8 |  |  |  |  |
| 1002 | 13666-222 | Carbonic Anhydrase X | Carbonic anhydrase-related protein 10 | Q9NS85 | CA10 |  |  |  |  |
| 1003 | 3289-19 | Carbonic Anhydrase X | Carbonic anhydrase-related protein 10 | Q9NS85 | CA10 |  |  |  |  |
| 1004 | 8859-51 | CAH11 | Carbonic anhydrase-related protein 11 | O75493 | CA11 |  |  |  |  |
| 1005 | 12381-26 | CBR1 | Carbonyl reductase [NADPH] 1 | P16152 | CBR1 | X |  |  | X |
| 1006 | 14091-42 | Carbonyl reductase 3 | Carbonyl reductase [NADPH] 3 | O75828 | CBR3 |  |  |  |  |
| 1007 | 21119-1 | CBR4 | Carbonyl reductase family member 4 | Q8N4T8 | CBR4 |  |  |  |  |
| 1008 | 25292-4 | EST3 | Carboxylesterase 3 | Q6UWW8 | CES3 | X |  |  |  |
| 1009 | 25292-6 | EST3 | Carboxylesterase 3 | Q6UWW8 | CES3 |  |  |  |  |
| 1010 | 19115-13 | CMBL | Carboxymethylenebutenolidase homolog | Q96DG6 | CMBL |  |  |  |  |
| 1011 | 19760-26 | CBPA1 | Carboxypeptidase A1 | P15085 | CPA1 |  |  |  |  |
| 1012 | 23981-172 | CBPA1 | Carboxypeptidase A1 | P15085 | CPA1 | X |  |  |  |
| 1013 | 9276-7 | CBPA2 | Carboxypeptidase A2 | P48052 | CPA2 |  |  |  |  |
| 1014 | 9267-2 | CBPA4 | Carboxypeptidase A4 | Q9UI42 | CPA4 |  |  |  |  |
| 1015 | 15375-49 | Carboxypeptidase B1 | Carboxypeptidase B | P15086 | CPB1 |  |  |  |  |
| 1016 | 6356-3 | Carboxypeptidase B1 | Carboxypeptidase B | P15086 | CPB1 |  |  |  |  |
| 1017 | 3518-54 | TAFI | Carboxypeptidase B2 | Q96IY4 | CPB2 |  |  | X |  |
| 1018 | 7917-17 | CBPD | Carboxypeptidase D | O75976 | CPD |  |  |  |  |
| 1019 | 5343-74 | CBPE | Carboxypeptidase E | P16870 | CPE |  |  |  |  |
| 1020 | 6024-68 | CBPE | Carboxypeptidase E | P16870 | CPE |  |  | X | X |
| 1021 | 7768-10 | CBPM | Carboxypeptidase M | P14384 | CPM |  |  |  |  |
| 1022 | 9416-77 | CBPM | Carboxypeptidase M | P14384 | CPM |  |  |  |  |
| 1023 | 7142-5 | CBPN | Carboxypeptidase N catalytic chain | P15169 | CPN1 |  |  | X |  |
| 1024 | 6415-90 | CPN2 | Carboxypeptidase N subunit 2 | P22792 | CPN2 |  |  |  |  |
| 1025 | 9394-19 | Aminopeptidase | Carboxypeptidase Q | Q9Y646 | CPQ |  |  |  | X |
| 1026 | 6493-9 | CBPZ | Carboxypeptidase Z | Q66K79 | CPZ |  |  |  |  |
| 1027 | 21166-1 | CTDS1 | Carboxy-terminal domain RNA polymerase II polypeptide A small phosphatase 1 | Q9GZU7 | CTDSP1 |  |  |  |  |
| 1028 | 24299-1 | CEA16 | Carcinoembryonic antigen-related cell adhesion molecule 16 | Q2WEN9 | CEACAM16 |  |  |  |  |
| 1029 | 7048-4 | CEA19 | Carcinoembryonic antigen-related cell adhesion molecule 19 | Q7Z692 | CEACAM19 |  |  |  |  |
| 1030 | 10453-7 | CEA20 | Carcinoembryonic antigen-related cell adhesion molecule 20 | Q6UY09 | CEACAM20 |  |  |  |  |
| 1031 | 7204-1 | CEA21 | Carcinoembryonic antigen-related cell adhesion molecule 21 | Q3KPI0 | CEACAM21 |  |  |  |  |
| 1032 | 8999-19 | CEAM3 | Carcinoembryonic antigen-related cell adhesion molecule 3 | P40198 | CEACAM3 |  |  |  |  |
| 1033 | 10910-6 | CEAM4 | Carcinoembryonic antigen-related cell adhesion molecule 4 | O75871 | CEACAM4 |  |  |  |  |
| 1034 | 7184-13 | CEAM7 | Carcinoembryonic antigen-related cell adhesion molecule 7 | Q14002 | CEACAM7 |  |  |  |  |
| 1035 | 18873-8 | CEAM8 | Carcinoembryonic antigen-related cell adhesion molecule 8 | P31997 | CEACAM8 |  |  |  |  |
| 1036 | 13732-79 | Cardiotrophin-1 | Cardiotrophin-1 | Q16619 | CTF1 | X |  | X |  |

| # | **Custom Panel (X)** | **SOMAmer SeqID** | **Target Name** | **Human Target or Analyte** | **UniProt ID** | **GeneID** | **Cardiovascular Disease** | **Inflammation and Immune**  **Response** | **Metabolic Disease** | **Oncology** |
| --- | --- | --- | --- | --- | --- | --- | --- | --- | --- | --- |

| 1037 | 2889-37 | Cardiotrophin-1 | Cardiotrophin-1 | Q16619 | CTF1 |  | | | |
| --- | --- | --- | --- | --- | --- | --- | --- | --- | --- |
| 1038 | 12637-7 | CACP | Carnitine O-acetyltransferase | P43155 | CRAT |  |  |  |  |
| 1039 | 11709-29 | CPT1B | Carnitine O-palmitoyltransferase 1, muscle isoform | Q92523 | CPT1B | X |  | X |  |
| 1040 | 23638-3 | CARME | Carnosine N-methyltransferase | Q8N4J0 | CARNMT1 |  |  |  |  |
| 1041 | 5632-6 | CRAC1 | Cartilage acidic protein 1 | Q9NQ79 | CRTAC1 |  |  |  |  |
| 1042 | 5717-2 | CILP | Cartilage intermediate layer protein 1 | O75339 | CILP |  |  |  |  |
| 1043 | 8841-65 | CILP2 | Cartilage intermediate layer protein 2 | Q8IUL8 | CILP2 |  |  |  |  |
| 1044 | 8043-153 | COMP | Cartilage oligomeric matrix protein | P49747 | COMP | X |  | X |  |
| 1045 | 12855-16 | CT032 | Cas scaffolding protein family member 4 | Q9NQ75 | CASS4 |  |  |  |  |
| 1046 | 22484-17 | KC1AL | Casein kinase I isoform alpha-like | Q8N752 | CSNK1A1L |  |  |  |  |
| 1047 | 11289-31 | KC1D | Casein kinase I isoform delta | P48730 | CSNK1D |  |  |  |  |
| 1048 | 20130-144 | KC1G1 | Casein kinase I isoform gamma-1 | Q9HCP0 | CSNK1G1 |  |  |  |  |
| 1049 | 12653-13 | CSNK1G2 | Casein kinase I isoform gamma-2 | P78368 | CSNK1G2 |  |  |  |  |
| 1050 | 17436-193 | CSNK1G2 | Casein kinase I isoform gamma-2 | P78368 | CSNK1G2 |  |  |  |  |
| 1051 | 5225-50 | CK2-A1:B | Casein kinase II 2-alpha:2-beta heterotetramer | P68400|P67870 | CSNK2A1|CSNK2B |  | X |  |  |
| 1052 | 5226-36 | CK2-A2:B | Casein kinase II 2-alpha':2-beta heterotetramer | P19784|P67870 | CSNK2A2|CSNK2B |  | X |  |  |
| 1053 | 3427-63 | CSK21 | Casein kinase II subunit alpha | P68400 | CSNK2A1 |  | X |  |  |
| 1054 | 5224-20 | CSK21 | Casein kinase II subunit alpha | P68400 | CSNK2A1 |  |  |  |  |
| 1055 | 13681-173 | CSK22 | Casein kinase II subunit alpha' | P19784 | CSNK2A2 |  |  |  |  |
| 1056 | 11330-15 | CSK2B | Casein kinase II subunit beta | P67870 | CSNK2B |  |  |  |  |
| 1057 | 16837-20 | FLIP | CASP8 and FADD-like apoptosis regulator | O15519 | CFLAR |  | X |  | X |
| 1058 | 18267-74 | CAR17 | Caspase recruitment domain-containing protein 17 | Q5XLA6 | CARD17 |  |  |  |  |
| 1059 | 18190-15 | ICBR | Caspase recruitment domain-containing protein 18 | P57730 | CARD18 |  |  |  |  |
| 1060 | 7778-104 | BINCA | Caspase recruitment domain-containing protein 19 | Q96LW7 | CARD19 |  |  |  |  |
| 1061 | 11405-150 | CARD9 | Caspase recruitment domain-containing protein 9 | Q9H257 | CARD9 |  | X |  |  |
| 1062 | 5340-24 | Caspase-10:region 1 | Caspase-10:region 1 | Q92851 | CASP10 |  | X |  | X |
| 1063 | 21450-333 | Caspase-10:region 2 | Caspase-10:region 2 | Q92851 | CASP10 |  | X |  | X |
| 1064 | 23156-146 | Caspase-10:region 2 | Caspase-10:region 2 | Q92851 | CASP10 |  |  |  |  |
| 1065 | 16617-14 | Caspase-14 | Caspase-14 | P31944 | CASP14 |  |  |  |  |
| 1066 | 4904-7 | Caspase-2 | Caspase-2 | P42575 | CASP2 |  |  |  | X |
| 1067 | 3593-72 | Caspase-3 | Caspase-3 | P42574 | CASP3 | X | X | X |  |
| 1068 | 22092-43 | Caspase-4 | Caspase-4 | P49662 | CASP4 |  |  |  |  |
| 1069 | 25895-29 | Caspase-5 | Caspase-5 | P51878 | CASP5 |  |  |  |  |
| 1070 | 21513-1 | Caspase-7 | Caspase-7 | P55210 | CASP7 | X |  |  | X |
| 1071 | 18158-45 | Caspase-8 | Caspase-8 | Q14790 | CASP8 | X | X | X | X |
| 1072 | 3488-64 | Catalase | Catalase | P04040 | CAT | X | X | X | X |
| 1073 | 18382-109 | Catechol O-methyltransferase | Catechol O-methyltransferase | P21964 | COMT | X |  |  | X |
| 1074 | 23253-5 | CMTD1 | Catechol O-methyltransferase domain-containing protein 1 | Q86VU5 | COMTD1 |  |  |  |  |
| 1075 | 24426-15 | CTNA1 | Catenin alpha-1 | P35221 | CTNNA1 |  |  | X | X |
| 1076 | 24426-191 | CTNA1 | Catenin alpha-1 | P35221 | CTNNA1 |  |  |  |  |
| 1077 | 9872-23 | CTNA2 | Catenin alpha-2 | P26232 | CTNNA2 |  |  |  |  |
| 1078 | 25283-2 | CTNA3 | Catenin alpha-3 | Q9UI47 | CTNNA3 | X | X |  |  |
| 1079 | 8418-30 | b-Catenin | Catenin beta-1 | P35222 | CTNNB1 |  |  |  |  |
| 1080 | 8424-269 | b-Catenin | Catenin beta-1 | P35222 | CTNNB1 |  |  |  |  |
| 1081 | 9842-2 | b-Catenin | Catenin beta-1 | P35222 | CTNNB1 | X | X | X | X |
| 1082 | 9384-17 | Cathelicidin peptide | Cathelicidin antimicrobial peptide | P49913 | CAMP |  |  |  |  |
| 1083 | 3061-61 | Cathepsin B | Cathepsin B | P07858 | CTSB |  |  |  |  |
| 1084 | 8007-19 | Cathepsin B | Cathepsin B | P07858 | CTSB |  |  |  | X |
| 1085 | 5508-62 | Cathepsin D | Cathepsin D | P07339 | CTSD | X | X | X | X |
| 1086 | 15376-134 | CATE | Cathepsin E | P14091 | CTSE |  |  |  |  |
| 1087 | 8393-121 | CATF | Cathepsin F | Q9UBX1 | CTSF |  |  |  |  |
| 1088 | 9212-22 | CATF | Cathepsin F | Q9UBX1 | CTSF |  |  |  |  |
| 1089 | 2431-17 | Cathepsin G | Cathepsin G | P08311 | CTSG |  |  |  |  |
| 1090 | 8465-52 | Cathepsin H | Cathepsin H | P09668 | CTSH |  |  |  |  |
| 1091 | 8644-46 | Cathepsin H | Cathepsin H | P09668 | CTSH |  | X | X |  |
| 1092 | 9443-137 | cathepsin K | Cathepsin K | P43235 | CTSK |  |  | X |  |
| 1093 | 3364-76 | Cathepsin V | Cathepsin L2 | O60911 | CTSV |  |  |  |  |
| 1094 | 9264-11 | CATO | Cathepsin O | P43234 | CTSO |  |  |  |  |
| 1095 | 3181-50 | Cathepsin S | Cathepsin S | P25774 | CTSS |  |  | X |  |
| 1096 | 4971-1 | CATZ | Cathepsin Z | Q9UBR2 | CTSZ | X |  |  |  |
| 1097 | 16057-6 | IGF-II receptor | Cation-independent mannose-6-phosphate receptor | P11717 | IGF2R | X |  |  |  |
| 1098 | 3676-15 | IGF-II receptor | Cation-independent mannose-6-phosphate receptor | P11717 | IGF2R |  |  |  |  |
| 1099 | 9457-3 | CAV2 | Caveolin-2 | P51636 | CAV2 | X | X |  |  |
| 1100 | 8690-25 | CAV3 | Caveolin-3 | P56539 | CAV3 | X |  | X |  |
| 1101 | 18218-48 | CB032 | CB1 cannabinoid receptor-interacting protein 1 | Q96F85 | CNRIP1 |  |  |  |  |
| 1102 | 13687-5 | I-309 | C-C motif chemokine 1 | P22362 | CCL1 |  |  |  |  |
| 1103 | 2770-51 | I-309 | C-C motif chemokine 1 | P22362 | CCL1 |  |  |  |  |
| 1104 | 4144-13 | MCP-4 | C-C motif chemokine 13 | Q99616 | CCL13 |  |  |  |  |
| 1105 | 2900-53 | HCC-1 | C-C motif chemokine 14 | Q16627 | CCL14 | X |  | X |  |
| 1106 | 14109-15 | MIP-5 | C-C motif chemokine 15 | Q16663 | CCL15 |  |  |  |  |
| 1107 | 18289-16 | MIP-5 | C-C motif chemokine 15 | Q16663 | CCL15 |  |  |  |  |
| 1108 | 3509-1 | MIP-5 | C-C motif chemokine 15 | Q16663 | CCL15 |  |  |  |  |
| 1109 | 4913-78 | HCC-4 | C-C motif chemokine 16 | O15467 | CCL16 |  |  |  |  |
| 1110 | 3519-3 | TARC | C-C motif chemokine 17 | Q92583 | CCL17 | X | X |  |  |
| 1111 | 3044-3 | PARC | C-C motif chemokine 18 | P55774 | CCL18 | X |  | X |  |

| # | **Custom Panel (X)** | **SOMAmer SeqID** | **Target Name** | **Human Target or Analyte** | **UniProt ID** | **GeneID** | **Cardiovascular Disease** | **Inflammation and Immune**  **Response** | **Metabolic Disease** | **Oncology** |
| --- | --- | --- | --- | --- | --- | --- | --- | --- | --- | --- |

| 1112 | 4922-13 | MIP-3b | C-C motif chemokine 19 | Q99731 | CCL19 |  | X |  | |
| --- | --- | --- | --- | --- | --- | --- | --- | --- | --- |
| 1113 | 2578-67 | MCP-1 | C-C motif chemokine 2 | P13500 | CCL2 | X | X | X | X |
| 1114 | 2468-62 | MIP-3a | C-C motif chemokine 20 | P78556 | CCL20 |  | X | X | X |
| 1115 | 2516-57 | 6Ckine | C-C motif chemokine 21 | O00585 | CCL21 |  | X |  |  |
| 1116 | 22993-9 | MDC | C-C motif chemokine 22 | O00626 | CCL22 |  | X |  |  |
| 1117 | 3508-78 | MDC | C-C motif chemokine 22 | O00626 | CCL22 |  |  |  |  |
| 1118 | 2913-1 | MPIF-1 | C-C motif chemokine 23 | P55773 | CCL23 |  |  |  |  |
| 1119 | 4128-27 | Eotaxin-2 | C-C motif chemokine 24 | O00175 | CCL24 | X | X |  |  |
| 1120 | 14068-29 | TECK | C-C motif chemokine 25 | O15444 | CCL25 |  |  |  |  |
| 1121 | 2705-5 | TECK | C-C motif chemokine 25 | O15444 | CCL25 |  |  |  |  |
| 1122 | 9168-31 | Eotaxin-3 | C-C motif chemokine 26 | Q9Y258 | CCL26 |  |  |  |  |
| 1123 | 2192-63 | CTACK | C-C motif chemokine 27 | Q9Y4X3 | CCL27 |  |  |  |  |
| 1124 | 2890-59 | CCL28 | C-C motif chemokine 28 | Q9NRJ3 | CCL28 |  |  |  |  |
| 1125 | 3040-59 | MIP-1a | C-C motif chemokine 3 | P10147 | CCL3 | X | X |  |  |
| 1126 | 2783-18 | LD78-beta | C-C motif chemokine 3-like 1 | P16619 | CCL3L1 |  | X |  |  |
| 1127 | 2781-63 | LAG-1 | C-C motif chemokine 4-like | Q8NHW4 | CCL4L1 |  |  |  |  |
| 1128 | 2523-31 | RANTES | C-C motif chemokine 5 | P13501 | CCL5 |  |  |  |  |
| 1129 | 5480-49 | RANTES | C-C motif chemokine 5 | P13501 | CCL5 | X | X | X |  |
| 1130 | 22969-12 | MCP-3 | C-C motif chemokine 7 | P80098 | CCL7 | X | X |  |  |
| 1131 | 4886-3 | MCP-3 | C-C motif chemokine 7 | P80098 | CCL7 |  |  |  |  |
| 1132 | 13748-4 | MCP-2 | C-C motif chemokine 8 | P80075 | CCL8 |  | X |  |  |
| 1133 | 2785-15 | MCP-2 | C-C motif chemokine 8 | P80075 | CCL8 |  |  |  |  |
| 1134 | 18874-66 | CEBPA | CCAAT/enhancer-binding protein alpha | P49715 | CEBPA |  | X | X | X |
| 1135 | 15675-3 | CEBPB | CCAAT/enhancer-binding protein beta | P17676 | CEBPB |  |  |  |  |
| 1136 | 22953-85 | CEBPB | CCAAT/enhancer-binding protein beta | P17676 | CEBPB | X |  |  |  |
| 1137 | 21208-163 | CEBPE | CCAAT/enhancer-binding protein epsilon | Q15744 | CEBPE |  | X |  |  |
| 1138 | 19572-10 | CEBPG | CCAAT/enhancer-binding protein gamma | P53567 | CEBPG |  |  |  |  |
| 1139 | 13482-14 | CNOT1 | CCR4-NOT transcription complex subunit 1 | A5YKK6 | CNOT1 |  |  |  |  |
| 1140 | 3290-50 | CD109 | CD109 antigen | Q6YHK3 | CD109 |  |  |  | X |
| 1141 | 21276-11 | CD133 | CD133 antigen | O43490 | PROM1 |  |  |  | X |
| 1142 | 17677-47 | BY55 | CD160 antigen | O95971 | CD160 |  |  |  |  |
| 1143 | 6915-2 | C16L2 | CD164 sialomucin-like 2 protein | Q6UWJ8 | CD164L2 |  |  |  |  |
| 1144 | 5451-1 | ALCAM | CD166 antigen | Q13740 | ALCAM | X |  |  | X |
| 1145 | 13116-25 | CD177 | CD177 antigen | Q8N6Q3 | CD177 |  |  |  |  |
| 1146 | 22576-1 | DC-SIGN | CD209 antigen | Q9NNX6 | CD209 |  |  |  |  |
| 1147 | 22576-2 | DC-SIGN | CD209 antigen | Q9NNX6 | CD209 |  |  |  |  |
| 1148 | 3029-52 | DC-SIGN | CD209 antigen | Q9NNX6 | CD209 |  | X |  |  |
| 1149 | 5062-60 | CD226 | CD226 antigen | Q15762 | CD226 |  |  |  |  |
| 1150 | 5721-1 | CD226 | CD226 antigen | Q15762 | CD226 |  | X |  |  |
| 1151 | 5412-53 | CD27 | CD27 antigen | P26842 | CD27 |  | X | X | X |
| 1152 | 20521-83 | CD28 | CD28 | P10747 | CD28 |  | X |  | X |
| 1153 | 23949-12 | CD29 | CD29 | P05556 | ITGB1 |  |  |  |  |
| 1154 | 21961-14 | CD2AP | CD2-associated protein | Q9Y5K6 | CD2AP |  |  |  |  |
| 1155 | 16320-139 | CD320 | CD320 antigen | Q9NPF0 | CD320 |  |  | X |  |
| 1156 | 3534-14 | CD40 ligand, soluble | CD40 ligand | P29965 | CD40LG | X | X | X |  |
| 1157 | 9283-8 | CD44 | CD44 antigen | P16070 | CD44 |  | X | X | X |
| 1158 | 3292-75 | CD48 | CD48 antigen | P09326 | CD48 |  |  |  | X |
| 1159 | 3293-2 | CD5L | CD5 antigen-like | O43866 | CD5L |  |  |  |  |
| 1160 | 10480-33 | CD59 | CD59 glycoprotein | P13987 | CD59 | X | X | X |  |
| 1161 | 11514-196 | CD59 | CD59 glycoprotein | P13987 | CD59 |  |  |  |  |
| 1162 | 20519-7 | CD6 | CD6 | P30203 | CD6 |  | X |  |  |
| 1163 | 9190-7 | CD63 | CD63 antigen | P08962 | CD63 |  |  |  |  |
| 1164 | 5807-77 | CD70 | CD70 antigen | P32970 | CD70 |  | X | X | X |
| 1165 | 8762-38 | CD70 | CD70 antigen | P32970 | CD70 |  |  |  |  |
| 1166 | 19612-3 | CD81 | CD81 antigen | P60033 | CD81 |  | X |  |  |
| 1167 | 20574-8 | CD81 | CD81 antigen | P60033 | CD81 |  |  |  |  |
| 1168 | 21480-2 | CD82 antigen | CD82 antigen | P27701 | CD82 |  |  |  |  |
| 1169 | 5065-8 | CD83 | CD83 antigen | Q01151 | CD83 |  | X |  |  |
| 1170 | 21692-12 | CD8A/CD8B Complex | CD8A/CD8B Complex | P01732|P10966 | CD8A|CD8B |  |  |  |  |
| 1171 | 17449-23 | CD9 | CD9 antigen | P21926 | CD9 |  |  |  | X |
| 1172 | 18881-7 | CD97 | CD97 antigen | P48960 | ADGRE5 |  |  |  |  |
| 1173 | 2822-56 | CD97 | CD97 antigen | P48960 | ADGRE5 |  |  |  |  |
| 1174 | 10539-30 | C99L2 | CD99 antigen-like protein 2 | Q8TCZ2 | CD99L2 |  |  |  |  |
| 1175 | 23403-64 | BORG4 | Cdc42 effector protein 4 | Q9H3Q1 | CDC42EP4 |  |  |  |  |
| 1176 | 17676-13 | CIP4 | Cdc42-interacting protein 4 | Q15642 | TRIP10 |  |  |  |  |
| 1177 | 7745-3 | ZCD1 | CDGSH iron-sulfur domain-containing protein 1 | Q9NZ45 | CISD1 |  |  |  |  |
| 1178 | 8094-20 | CISD2 | CDGSH iron-sulfur domain-containing protein 2 | Q8N5K1 | CISD2 |  |  | X |  |
| 1179 | 21128-2 | CDK2 | CDK2 | P24941 | CDK2 |  | X |  | X |
| 1180 | 7105-7 | CABL2 | CDK5 and ABL1 enzyme substrate 2 | Q9BTV7 | CABLES2 |  |  |  |  |
| 1181 | 21238-40 | CK5P3 | CDK5 regulatory subunit-associated protein 3 | Q96JB5 | CDK5RAP3 |  |  |  |  |
| 1182 | 23540-3 | MAT1 | CDK-activating kinase assembly factor MAT1 | P51948 | MNAT1 |  |  |  |  |
| 1183 | 3326-58 | Nectin-like protein 2 | Cell adhesion molecule 1 | Q9BY67 | CADM1 |  |  |  | X |
| 1184 | 16907-3 | Nectin-like protein 3 | Cell adhesion molecule 2 | Q8N3J6 | CADM2 |  |  |  |  |
| 1185 | 3630-27 | Nectin-like protein 1 | Cell adhesion molecule 3 | Q8N126 | CADM3 |  |  |  |  |
| 1186 | 4541-49 | CDON | Cell adhesion molecule-related/down-regulated by oncogenes | Q4KMG0 | CDON |  |  |  |  |

| # | **Custom Panel (X)** | **SOMAmer SeqID** | **Target Name** | **Human Target or Analyte** | **UniProt ID** | **GeneID** | **Cardiovascular Disease** | **Inflammation and Immune**  **Response** | **Metabolic Disease** | **Oncology** |
| --- | --- | --- | --- | --- | --- | --- | --- | --- | --- | --- |

| 1187 | 12670-15 | RAD1 | Cell cycle checkpoint protein RAD1 | O60671 | RAD1 |  | | | |
| --- | --- | --- | --- | --- | --- | --- | --- | --- | --- |
| 1188 | 8889-5 | CCPG1 | Cell cycle progression protein 1 | Q9ULG6 | CCPG1 |
| 1189 | 24678-10 | AVEN | Cell death regulator Aven | Q9NQS1 | AVEN |
| 1190 | 8975-26 | RCD1 | Cell differentiation protein RCD1 homolog | Q92600 | CNOT9 |
| 1191 | 9840-2 | Cdc42Hs | Cell division control protein 42 homolog | P60953 | CDC42 |  | X |  | X |
| 1192 | 6257-56 | CGRE1 | Cell growth regulator with EF hand domain protein 1 | Q99674 | CGREF1 |  |  |  |  |
| 1193 | 10636-1 | MO2R1:CD | Cell surface glycoprotein CD200 receptor 1:Isoform 4, Cytoplasmic Domain | Q8TD46 | CD200R1 |  |  |  |  |
| 1194 | 5103-30 | MO2R1:ECD | Cell surface glycoprotein CD200 receptor 1:Isoform 4, Extracellular Domain | Q8TD46 | CD200R1 |  |  |  |  |
| 1195 | 8980-19 | MO2R2 | Cell surface glycoprotein CD200 receptor 2 | Q6Q8B3 | CD200R1L |  |  |  |  |
| 1196 | 11967-23 | RABP1 | Cellular retinoic acid-binding protein 1 | P29762 | CRABP1 |  |  |  | X |
| 1197 | 11696-7 | RABP2 | Cellular retinoic acid-binding protein 2 | P29373 | CRABP2 |  |  | X |  |
| 1198 | 6123-69 | p53 | Cellular tumor antigen p53 | P04637 | TP53 | X | X | X | X |
| 1199 | 6168-11 | p53 R175H | Cellular tumor antigen p53 R175H mutant | P04637 | TP53 |  |  |  |  |
| 1200 | 21106-206 | CETN1 | Centrin-1 | Q12798 | CETN1 |  |  |  |  |
| 1201 | 13078-3 | CETN2 | Centrin-2 | P41208 | CETN2 |  |  |  |  |
| 1202 | 17410-5 | CETN3 | Centrin-3 | O15182 | CETN3 |  |  |  |  |
| 1203 | 8817-29 | CENPV | Centromere protein V | Q7Z7K6 | CENPV |  |  |  |  |
| 1204 | 8864-59 | CENPW | Centromere protein W | Q5EE01 | CENPW |  |  |  |  |
| 1205 | 25262-6 | ZW10 | Centromere/kinetochore protein zw10 homolog | O43264 | ZW10 |  |  |  |  |
| 1206 | 23292-1 | CE112 | Centrosomal protein of 112 kDa | Q8N8E3 | CEP112 |  |  |  |  |
| 1207 | 23560-154 | CEP41 | Centrosomal protein of 41 kDa | Q9BYV8 | CEP41 |  |  |  |  |
| 1208 | 23675-16 | CEP76 | Centrosomal protein of 76 kDa | Q8TAP6 | CEP76 |  |  |  |  |
| 1209 | 13494-6 | CERS5 | Ceramide synthase 5 | Q8N5B7 | CERS5 |  |  |  |  |
| 1210 | 20385-21 | CPTP | Ceramide-1-phosphate transfer protein | Q5TA50 | CPTP |  |  |  |  |
| 1211 | 21426-88 | CPTP | Ceramide-1-phosphate transfer protein | Q5TA50 | CPTP |  |  |  |  |
| 1212 | 6230-56 | CER1 | Cerberus | O95813 | CER1 |  |  |  |  |
| 1213 | 9313-27 | CBLN1 | Cerebellin-1 | P23435 | CBLN1 |  |  |  |  |
| 1214 | 21887-2 | CBLN2 | Cerebellin-2 | Q8IUK8 | CBLN2 |  |  |  |  |
| 1215 | 5688-65 | CBLN4 | Cerebellin-4 | Q9NTU7 | CBLN4 |  |  |  |  |
| 1216 | 12347-29 | CCM2 | Cerebral cavernous malformations 2 protein | Q9BSQ5 | CCM2 | X |  |  | X |
| 1217 | 13693-5 | ARMEL | Cerebral dopamine neurotrophic factor | Q49AH0 | CDNF |  |  |  |  |
| 1218 | 4962-52 | ARMEL | Cerebral dopamine neurotrophic factor | Q49AH0 | CDNF |  |  |  |  |
| 1219 | 9795-9 | CLN5:CD | Ceroid-lipofuscinosis neuronal protein 5:Cytoplasmic domain | O75503 | CLN5 |  |  |  |  |
| 1220 | 8874-53 | CLN5:LD | Ceroid-lipofuscinosis neuronal protein 5:Lumenal domain | O75503 | CLN5 |  |  | X |  |
| 1221 | 17453-34 | Ceruloplasmin | Ceruloplasmin | P00450 | CP |  | X | X | X |
| 1222 | 23378-29 | CGBP1 | CGG triplet repeat-binding protein 1 | Q9UFW8 | CGGBP1 |  |  |  |  |
| 1223 | 5246-64 | cGMP-stimulated PDE | cGMP-dependent 3',5'-cyclic phosphodiesterase | O00408 | PDE2A |  |  |  | X |
| 1224 | 13067-5 | KGP1B | cGMP-dependent protein kinase 1, beta isozyme | Q13976 | PRKG1 |  | X | X |  |
| 1225 | 5254-69 | PDE3A | cGMP-inhibited 3',5'-cyclic phosphodiesterase A | Q14432 | PDE3A | X |  |  |  |
| 1226 | 16805-5 | PDE5A | cGMP-specific 3',5'-cyclic phosphodiesterase | O76074 | PDE5A |  |  |  |  |
| 1227 | 5256-86 | PDE5A | cGMP-specific 3',5'-cyclic phosphodiesterase | O76074 | PDE5A |  |  |  |  |
| 1228 | 18224-11 | CHM1A | Charged multivesicular body protein 1a | Q9HD42 | CHMP1A |  |  |  |  |
| 1229 | 18207-6 | CHM1B | Charged multivesicular body protein 1b | Q7LBR1 | CHMP1B |  |  |  |  |
| 1230 | 17728-61 | CHM2A | Charged multivesicular body protein 2a | O43633 | CHMP2A |  |  |  |  |
| 1231 | 17350-13 | CHM2B | Charged multivesicular body protein 2b | Q9UQN3 | CHMP2B |  |  | X |  |
| 1232 | 12508-9 | CHMP3 | Charged multivesicular body protein 3 | Q9Y3E7 | CHMP3 |  |  |  |  |
| 1233 | 21341-19 | CHM4A | Charged multivesicular body protein 4a | Q9BY43 | CHMP4A |  |  |  |  |
| 1234 | 21160-4 | CHMP6 | Charged multivesicular body protein 6 | Q96FZ7 | CHMP6 |  |  |  |  |
| 1235 | 21290-66 | HUS1 | Checkpoint protein HUS1 | O60921 | HUS1 |  |  |  |  |
| 1236 | 25898-4 | CHID1 | Chitinase domain-containing protein 1 | Q9BWS9 | CHID1 |  |  |  |  |
| 1237 | 11104-13 | YKL-40 | Chitinase-3-like protein 1 | P36222 | CHI3L1 | X |  |  | X |
| 1238 | 9383-24 | CH3L2 | Chitinase-3-like protein 2 | Q15782 | CHI3L2 |  |  |  |  |
| 1239 | 10460-1 | Chitotriosidase-1 | Chitotriosidase-1 | Q13231 | CHIT1 |  |  |  |  |
| 1240 | 3600-2 | Chitotriosidase-1 | Chitotriosidase-1 | Q13231 | CHIT1 | X |  | X |  |
| 1241 | 13683-18 | CLCKB | Chloride channel protein ClC-Kb | P51801 | CLCNKB |  |  |  |  |
| 1242 | 5013-2 | NCC27 | Chloride intracellular channel protein 1 | O00299 | CLIC1 |  | X |  | X |
| 1243 | 17837-5 | CLIC2 | Chloride intracellular channel protein 2 | O15247 | CLIC2 |  |  |  |  |
| 1244 | 22954-10 | CLIC2 | Chloride intracellular channel protein 2 | O15247 | CLIC2 |  |  |  |  |
| 1245 | 24693-5 | CLIC3 | Chloride intracellular channel protein 3 | O95833 | CLIC3 |  |  |  |  |
| 1246 | 12491-23 | CLIC4 | Chloride intracellular channel protein 4 | Q9Y696 | CLIC4 |  |  |  |  |
| 1247 | 15314-49 | CLIC4 | Chloride intracellular channel protein 4 | Q9Y696 | CLIC4 |  |  |  |  |
| 1248 | 12475-48 | CLIC5 | Chloride intracellular channel protein 5 | Q9NZA1 | CLIC5 |  |  |  |  |
| 1249 | 6918-183 | CCKN | Cholecystokinin | P06307 | CCK |  |  |  |  |
| 1250 | 7131-8 | CETP | Cholesteryl ester transfer protein | P11597 | CETP | X |  | X |  |
| 1251 | 13117-232 | CHKB | Choline/ethanolamine kinase | Q9Y259 | CHKB |  |  |  |  |
| 1252 | 7761-125 | CHKB | Choline/ethanolamine kinase | Q9Y259 | CHKB |  |  | X |  |
| 1253 | 23349-5 | PCY1A | Choline-phosphate cytidylyltransferase A | P49585 | PCYT1A |  |  |  |  |
| 1254 | 15514-26 | Pseudocholinesterase | Cholinesterase | P06276 | BCHE | X | X | X | X |
| 1255 | 8239-223 | Pseudocholinesterase | Cholinesterase | P06276 | BCHE |  |  |  |  |
| 1256 | 13460-4 | CHAD | Chondroadherin | O15335 | CHAD |  |  |  |  |
| 1257 | 18875-125 | Chondrocalcin | Chondrocalcin | P02458 | COL2A1 |  |  |  |  |
| 1258 | 10772-21 | CGAT2 | Chondroitin sulfate N-acetylgalactosaminyltransferase 2 | Q8N6G5 | CSGALNACT2 |  |  |  |  |
| 1259 | 8951-162 | CSPG4 | Chondroitin sulfate proteoglycan 4 | Q6UVK1 | CSPG4 |  |  |  |  |
| 1260 | 22118-7 | CSAG2 | Chondrosarcoma-associated gene 2/3 protein | Q9Y5P2 | CSAG2 |  |  |  |  |
| 1261 | 13438-115 | CHRD | Chordin | Q9H2X0 | CHRD |  |  |  |  |

| # | **Custom Panel (X)** | **SOMAmer SeqID** | **Target Name** | **Human Target or Analyte** | **UniProt ID** | **GeneID** | **Cardiovascular Disease** | **Inflammation and Immune**  **Response** | **Metabolic Disease** | **Oncology** |
| --- | --- | --- | --- | --- | --- | --- | --- | --- | --- | --- |

| 1262 | 3362-61 | CRDL1 | Chordin-like protein 1 | Q9BU40 | CHRDL1 |  |  |  | |
| --- | --- | --- | --- | --- | --- | --- | --- | --- | --- |
| 1263 | 6086-15 | CRDL2 | Chordin-like protein 2 | Q6WN34 | CHRDL2 |  |  |
| 1264 | 21391-17 | b-CF | Choriogonadotropin subunit beta 3 | P0DN86 | CGB3 |  |  |
| 1265 | 6213-10 | CGB2 | Choriogonadotropin subunit beta variant 2 | Q6NT52 | CGB2 |  |  |
| 1266 | 13103-125 | CSH | Chorionic somatomammotropin hormone | P0DML2|P0DML3 | CSH1|CSH2 |  |  |
| 1267 | 6229-13 | CSH | Chorionic somatomammotropin hormone | P0DML2|P0DML3 | CSH1|CSH2 |  |  |
| 1268 | 21153-5 | BAP18 | Chromatin complexes subunit BAP18 | Q8IXM2 | BAP18 |  |  |
| 1269 | 18817-50 | CBX1 | Chromobox protein homolog 1 | P83916 | CBX1 |  |  |
| 1270 | 22095-29 | CBX2 | Chromobox protein homolog 2 | Q14781 | CBX2 |  |  |
| 1271 | 18868-7 | CBX3 | Chromobox protein homolog 3 | Q13185 | CBX3 |  |  |
| 1272 | 4540-11 | CBX5 | Chromobox protein homolog 5 | P45973 | CBX5 |  |  |
| 1273 | 13027-20 | CBX7 | Chromobox protein homolog 7 | O95931 | CBX7 | X | X |
| 1274 | 10752-8 | CDYL2 | Chromodomain Y-like protein 2 | Q8N8U2 | CDYL2 |  |  |  |  |
| 1275 | 25072-11 | CHD1L | Chromodomain-helicase-DNA-binding protein 1-like | Q86WJ1 | CHD1L |  |  |  |  |
| 1276 | 14005-2 | CHD7 | Chromodomain-helicase-DNA-binding protein 7 | Q9P2D1 | CHD7 |  | X | X |  |
| 1277 | 11184-51 | CgA | Chromogranin-A | P10645 | CHGA |  |  |  |  |
| 1278 | 8476-11 | CgA | Chromogranin-A | P10645 | CHGA |  |  |  |  |
| 1279 | 24294-13 | CTF8 | Chromosome transmission fidelity protein 8 homolog | P0CG13 | CHTF8 |  |  |  |  |
| 1280 | 3423-59 | Chymase | Chymase | P23946 | CMA1 | X |  |  |  |
| 1281 | 5626-20 | CTRC | Chymotrypsin-C | Q99895 | CTRC | X |  | X |  |
| 1282 | 6107-3 | ELA1 | Chymotrypsin-like elastase family member 1 | Q9UNI1 | CELA1 |  |  |  |  |
| 1283 | 7140-1 | ELA2A | Chymotrypsin-like elastase family member 2A | P08217 | CELA2A | X |  | X |  |
| 1284 | 6357-83 | ELA3B | Chymotrypsin-like elastase family member 3B | P08861 | CELA3B |  |  |  |  |
| 1285 | 9229-9 | CTRL | Chymotrypsin-like protease CTRL-1 | P40313 | CTRL |  |  |  |  |
| 1286 | 5671-1 | Chymotrypsin | Chymotrypsinogen B | P17538 | CTRB1 |  |  |  |  |
| 1287 | 5648-28 | CTRB2 | Chymotrypsinogen B2 | Q6GPI1 | CTRB2 |  |  |  |  |
| 1288 | 21109-1 | CFA36 | Cilia- and flagella-associated protein 36 | Q96G28 | CFAP36 |  |  |  |  |
| 1289 | 6553-68 | CCD19 | Cilia- and flagella-associated protein 45 | Q9UL16 | CFAP45 |  |  |  |  |
| 1290 | 3489-9 | CNTF | Ciliary neurotrophic factor | P26441 | CNTF | X |  | X |  |
| 1291 | 14101-2 | CNTFR alpha | Ciliary neurotrophic factor receptor subunit alpha | P26992 | CNTFR |  |  |  |  |
| 1292 | 2711-6 | CNTFR alpha | Ciliary neurotrophic factor receptor subunit alpha | P26992 | CNTFR |  |  |  |  |
| 1293 | 25242-12 | CATIP | Ciliogenesis-associated TTC17-interacting protein | Q7Z7H3 | CATIP |  |  |  |  |
| 1294 | 23541-61 | CLYBL | Citrate lyase subunit beta-like protein, mitochondrial | Q8N0X4 | CLYBL |  |  |  |  |
| 1295 | 18309-18 | CISY | Citrate synthase, mitochondrial | O75390 | CS | X |  | X |  |
| 1296 | 3028-36 | Ck-b-8-1 | Ck-beta-8-1 | P55773 | CCL23 |  | X |  |  |
| 1297 | 23269-40 | CKLF4 | CKLF-like MARVEL transmembrane domain-containing protein 4 | Q8IZR5 | CMTM4 |  |  |  |  |
| 1298 | 13711-10 | CLH1 | Clathrin heavy chain 1 | Q00610 | CLTC |  | X |  |  |
| 1299 | 11659-31 | EPN4 | Clathrin interactor 1 | Q14677 | CLINT1 |  |  |  |  |
| 1300 | 19257-11 | CLCA | Clathrin light chain A | P09496 | CLTA |  |  |  |  |
| 1301 | 24641-10 | CLD1 | Claudin-1 | O95832 | CLDN1 |  |  |  | X |
| 1302 | 20972-37 | CPSF5 | Cleavage and polyadenylation specificity factor subunit 5 | O43809 | NUDT21 |  |  |  |  |
| 1303 | 24055-44 | CSTF1 | Cleavage stimulation factor 50 kDa subunit | Q05048 | CSTF1 |  |  |  |  |
| 1304 | 21853-3 | CLC1A | CLEC-1 | Q8NC01 | CLEC1A |  | X |  |  |
| 1305 | 24941-14 | CLU | Clustered mitochondria protein homolog | O75153 | CLUH |  |  |  |  |
| 1306 | 4542-24 | Clusterin | Clusterin | P10909 | CLU |  | X | X |  |
| 1307 | 23307-7 | CLUA1 | Clusterin-associated protein 1 | Q96AJ1 | CLUAP1 |  |  |  |  |
| 1308 | 21492-19 | CLUL1 | Clusterin-like protein 1 | Q15846 | CLUL1 |  |  |  | X |
| 1309 | 25100-11 | CMIP | C-Maf-inducing protein | Q8IY22 | CMIP |  |  | X |  |
| 1310 | 5657-28 | SIA4A | CMP-N-acetylneuraminate-beta-galactosamide-alpha-2,3-sialyltransferase 1 | Q11201 | ST3GAL1 |  | X |  |  |
| 1311 | 6281-51 | SIA4B | CMP-N-acetylneuraminate-beta-galactosamide-alpha-2,3-sialyltransferase 2 | Q16842 | ST3GAL2 |  |  |  |  |
| 1312 | 7038-45 | SIA8D | CMP-N-acetylneuraminate-poly-alpha-2,8-sialyltransferase | Q92187 | ST8SIA4 |  |  |  |  |
| 1313 | 5623-11 | CLM1 | CMRF35-like molecule 1 | Q8TDQ1 | CD300LF |  |  |  |  |
| 1314 | 10798-4 | CLM2 | CMRF35-like molecule 2 | Q496F6 | CD300E |  |  |  |  |
| 1315 | 8287-17 | CLM2 | CMRF35-like molecule 2 | Q496F6 | CD300E |  |  |  | X |
| 1316 | 13406-161 | CLM6 | CMRF35-like molecule 6 | Q08708 | CD300C |  |  |  |  |
| 1317 | 5066-134 | CLM6 | CMRF35-like molecule 6 | Q08708 | CD300C |  |  |  |  |
| 1318 | 10713-151 | CLM7 | CMRF35-like molecule 7 | A8K4G0 | CD300LB |  |  |  |  |
| 1319 | 5630-48 | CM35H | CMRF35-like molecule 8 | Q9UGN4 | CD300A |  |  |  |  |
| 1320 | 20585-5 | CLM9 | CMRF35-like molecule 9 | Q6UXG3 | CD300LG |  |  |  |  |
| 1321 | 20585-9 | CLM9 | CMRF35-like molecule 9 | Q6UXG3 | CD300LG |  |  |  |  |
| 1322 | 19250-50 | MYCBP | C-Myc-binding protein | Q99417 | MYCBP |  |  |  |  |
| 1323 | 4905-63 | Coactosin-like protein | Coactosin-like protein | Q14019 | COTL1 |  |  |  | X |
| 1324 | 4876-32 | Coagulation Factor IX | Coagulation factor IX | P00740 | F9 | X |  | X |  |
| 1325 | 5307-12 | Coagulation Factor IXab | Coagulation factor IXab | P00740 | F9 |  |  |  |  |
| 1326 | 4906-35 | Coagulation Factor V | Coagulation Factor V | P12259 | F5 | X |  |  | X |
| 1327 | 3184-25 | Coagulation Factor VII | Coagulation factor VII | P08709 | F7 | X | X | X |  |
| 1328 | 13499-30 | Coagulation Factor VIII | Coagulation Factor VIII | P00451 | F8 | X | X |  |  |
| 1329 | 4878-3 | Coagulation Factor X | Coagulation Factor X | P00742 | F10 |  |  |  |  |
| 1330 | 3077-66 | Coagulation Factor Xa | Coagulation factor Xa | P00742 | F10 | X |  |  |  |
| 1331 | 2190-55 | Coagulation Factor XI | Coagulation Factor XI | P03951 | F11 | X |  | X |  |
| 1332 | 16927-9 | Coagulation factor XIII | Coagulation factor XIII | P00488|P05160 | F13A1|F13B | X | X |  |  |
| 1333 | 5658-64 | coagulation factor XIII B | Coagulation factor XIII B chain | P05160 | F13B | X |  |  |  |
| 1334 | 24459-15 | COPB2 | Coatomer subunit beta' | P35606 | COPB2 |  |  | X |  |
| 1335 | 19310-81 | COPE | Coatomer subunit epsilon | O14579 | COPE |  |  |  |  |
| 1336 | 17783-9 | MMAB | Cob(I)yrinic acid a,c-diamide adenosyltransferase, mitochondrial | Q96EY8 | MMAB |  |  | X |  |

| # | **Custom Panel (X)** | **SOMAmer SeqID** | **Target Name** | **Human Target or Analyte** | **UniProt ID** | **GeneID** | **Cardiovascular Disease** | **Inflammation and Immune**  **Response** | **Metabolic Disease** | **Oncology** |
| --- | --- | --- | --- | --- | --- | --- | --- | --- | --- | --- |

| 1337 | 5743-82 | CART | Cocaine- and amphetamine-regulated transcript protein | Q16568 | CARTPT |  |  | X |  |
| --- | --- | --- | --- | --- | --- | --- | --- | --- | --- |
| 1338 | 7227-75 | COCH | Cochlin | O43405 | COCH |  |  |  |
| 1339 | 21457-141 | CQ10A | Coenzyme Q-binding protein COQ10 homolog A, mitochondrial | Q96MF6 | COQ10A |  |  |  |
| 1340 | 4203-50 | Cofilin-1 | Cofilin-1 | P23528 | CFL1 |  |  |  | X |
| 1341 | 15339-32 | COF2 | Cofilin-2 | Q9Y281 | CFL2 | X |  |  |  |
| 1342 | 23314-46 | CC103 | Coiled-coil domain-containing protein 103 | Q8IW40 | CCDC103 | X |  |  |  |
| 1343 | 23390-6 | CC107 | Coiled-coil domain-containing protein 107 | Q8WV48 | CCDC107 |  |  |  |  |
| 1344 | 24658-98 | CC115 | Coiled-coil domain-containing protein 115 | Q96NT0 | CCDC115 |  |  |  |  |
| 1345 | 6388-21 | CC126 | Coiled-coil domain-containing protein 126 | Q96EE4 | CCDC126 |  |  |  |  |
| 1346 | 15300-66 | CC134 | Coiled-coil domain-containing protein 134 | Q9H6E4 | CCDC134 |  |  |  |  |
| 1347 | 5587-3 | CC134 | Coiled-coil domain-containing protein 134 | Q9H6E4 | CCDC134 |  |  |  |  |
| 1348 | 25409-5 | CC140 | Coiled-coil domain-containing protein 140 | Q96MF4 | CCDC140 |  |  |  |  |
| 1349 | 24702-31 | CC149 | Coiled-coil domain-containing protein 149 | Q6ZUS6 | CCDC149 |  |  |  |  |
| 1350 | 7797-11 | CC167 | Coiled-coil domain-containing protein 167 | Q9P0B6 | CCDC167 |  |  |  |  |
| 1351 | 23356-32 | CCD24 | Coiled-coil domain-containing protein 24 | Q8N4L8 | CCDC24 |  |  |  |  |
| 1352 | 18264-12 | CCD25 | Coiled-coil domain-containing protein 25 | Q86WR0 | CCDC25 |  |  |  |  |
| 1353 | 21595-8 | CCD43 | Coiled-coil domain-containing protein 43 | Q96MW1 | CCDC43 |  |  |  |  |
| 1354 | 12399-194 | CCD50 | Coiled-coil domain-containing protein 50 | Q8IVM0 | CCDC50 |  |  |  |  |
| 1355 | 12790-10 | CCD51 | Coiled-coil domain-containing protein 51 | Q96ER9 | CCDC51 |  |  |  |  |
| 1356 | 20932-10 | CCD69 | Coiled-coil domain-containing protein 69 | A6NI79 | CCDC69 |  |  |  |  |
| 1357 | 3234-23 | URB | Coiled-coil domain-containing protein 80 | Q76M96 | CCDC80 |  | X |  |  |
| 1358 | 23596-17 | CCD89 | Coiled-coil domain-containing protein 89 | Q8N998 | CCDC89 |  |  |  |  |
| 1359 | 25116-11 | CCDC9 | Coiled-coil domain-containing protein 9 | Q9Y3X0 | CCDC9 |  |  |  |  |
| 1360 | 7792-58 | CC90B | Coiled-coil domain-containing protein 90B, mitochondrial | Q9GZT6 | CCDC90B |  |  |  |  |
| 1361 | 23418-66 | CCD92 | Coiled-coil domain-containing protein 92 | Q53HC0 | CCDC92 | X |  | X |  |
| 1362 | 23571-93 | CCD94 | Coiled-coil domain-containing protein 94 | Q9BW85 | YJU2 |  |  |  |  |
| 1363 | 23321-42 | CCD95 | Coiled-coil domain-containing protein 95 | Q8NBZ0 | INO80E |  |  |  |  |
| 1364 | 23561-4 | CCD97 | Coiled-coil domain-containing protein 97 | Q96F63 | CCDC97 |  |  |  |  |
| 1365 | 24289-1 | CG053 | Coiled-coil domain-containing transmembrane protein C7orf53 | Q8N8F7 | LSMEM1 |  |  |  |  |
| 1366 | 11270-17 | CHC10 | Coiled-coil-helix-coiled-coil-helix domain-containing protein 10, mitochondrial | Q8WYQ3 | CHCHD10 |  |  | X |  |
| 1367 | 20387-277 | CHCH7 | Coiled-coil-helix-coiled-coil-helix domain-containing protein 7 | Q9BUK0 | CHCHD7 |  |  |  |  |
| 1368 | 12754-14 | CSDC2 | Cold shock domain-containing protein C2 | Q9Y534 | CSDC2 |  |  |  |  |
| 1369 | 12735-39 | CSDE1 | Cold shock domain-containing protein E1 | O75534 | CSDE1 |  |  |  |  |
| 1370 | 12724-81 | CIRBP | Cold-inducible RNA-binding protein | Q14011 | CIRBP | X |  |  |  |
| 1371 | 5749-53 | COL | Colipase | P04118 | CLPS |  |  |  |  |
| 1372 | 9526-3 | COLL1 | Colipase-like protein 1 | A2RUU4 | CLPSL1 |  |  |  |  |
| 1373 | 7767-1 | CF126 | Colipase-like protein 2 | Q6UWE3 | CLPSL2 |  |  |  |  |
| 1374 | 11140-56 | CO1A1:C-term propeptide | Collagen alpha-1(I) chain:C-term propeptide | P02452 | COL1A1 | X | X |  |  |
| 1375 | 13484-69 | CO1A1:N-term propeptide | Collagen alpha-1(I) chain:N-term propeptide | P02452 | COL1A1 | X | X | X |  |
| 1376 | 15466-30 | CO9A1 | Collagen alpha-1(IX) chain | P20849 | COL9A1 |  |  |  |  |
| 1377 | 6631-17 | CO9A1 | Collagen alpha-1(IX) chain | P20849 | COL9A1 |  |  |  | X |
| 1378 | 22047-46 | CO5A1 | Collagen alpha-1(V) chain | P20908 | COL5A1 | X | X |  | X |
| 1379 | 11150-3 | Collagen a1(VI) | Collagen alpha-1(VI) chain | P12109 | COL6A1 |  |  |  |  |
| 1380 | 16828-8 | Collagen a1(VI) | Collagen alpha-1(VI) chain | P12109 | COL6A1 | X |  |  |  |
| 1381 | 4807-13 | CO8A1 | Collagen alpha-1(VIII) chain | P27658 | COL8A1 | X |  |  |  |
| 1382 | 15653-9 | COAA1 | Collagen alpha-1(X) chain | Q03692 | COL10A1 |  |  |  |  |
| 1383 | 6570-1 | CODA1 | Collagen alpha-1(XIII) chain | Q5TAT6 | COL13A1 |  |  |  |  |
| 1384 | 8974-172 | COFA1 | Collagen alpha-1(XV) chain | P39059 | COL15A1 |  |  |  |  |
| 1385 | 8804-39 | COKA1 | Collagen alpha-1(XX) chain | Q9P218 | COL20A1 |  |  |  |  |
| 1386 | 4543-65 | CONA1 | Collagen alpha-1(XXIII) chain | Q86Y22 | COL23A1 |  |  |  |  |
| 1387 | 7006-4 | COPA1 | Collagen alpha-1(XXV) chain | Q9BXS0 | COL25A1 |  |  |  |  |
| 1388 | 10702-1 | COSA1 | Collagen alpha-1(XXVIII) chain | Q2UY09 | COL28A1 |  |  |  |  |
| 1389 | 16753-46 | CO6A2 | Collagen alpha-2(VI) chain | P12110 | COL6A2 |  |  |  |  |
| 1390 | 11278-4 | COL11A2 | Collagen alpha-2(XI) chain | P13942 | COL11A2 |  |  |  |  |
| 1391 | 20175-17 | CO9A3 | Collagen alpha-3(IX) chain | Q14050 | COL9A3 |  |  |  |  |
| 1392 | 11196-31 | Collagen alpha-3(VI):BPTI/Kunitz inhibitor | Collagen alpha-3(VI) chain:Bovine pancreatic trypsin inhibitor/Kunitz inhibitor domain, isoform | P12111 | COL6A3 |  |  |  |  |
| 1393 | 10511-10 | Collagen alpha-3(VI):isoform 3 | Collagen alpha-3(VI) chain:isoform 3 | P12111 | COL6A3 |  |  |  |  |
| 1394 | 11155-16 | CO6A5 | Collagen alpha-5(VI) chain | A8TX70 | COL6A5 |  |  |  |  |
| 1395 | 15467-10 | CTHR1 | Collagen triple helix repeat-containing protein 1 | Q96CG8 | CTHRC1 |  |  |  |  |
| 1396 | 6236-51 | CTHR1 | Collagen triple helix repeat-containing protein 1 | Q96CG8 | CTHRC1 |  |  |  |  |
| 1397 | 15569-15 | Collagen II | Collagen Type II | P02458 | COL2A1 |  | X |  |  |
| 1398 | 18880-81 | Collagen Type III | Collagen Type III | P02461 | COL3A1 | X | X | X |  |
| 1399 | 13535-2 | C43BP:PH domain | Collagen type IV alpha-3-binding protein:PH domain | Q9Y5P4 | CERT1 |  |  |  |  |
| 1400 | 13950-9 | C43BP:START domain | Collagen type IV alpha-3-binding protein:StAR-related lipid-transfer domain, isoform 2 | Q9Y5P4 | CERT1 |  |  |  |  |
| 1401 | 4925-54 | MMP-13 | Collagenase 3 | P45452 | MMP13 |  |  |  |  |
| 1402 | 6558-5 | COL10 | Collectin-10 | Q9Y6Z7 | COLEC10 | X |  |  |  |
| 1403 | 4430-44 | Collectin Kidney 1 | Collectin-11 | Q9BWP8 | COLEC11 | X |  |  |  |
| 1404 | 5457-5 | COLEC12 | Collectin-12 | Q5KU26 | COLEC12 |  |  |  |  |
| 1405 | 9787-23 | COLEC12 | Collectin-12 | Q5KU26 | COLEC12 |  |  |  |  |
| 1406 | 12509-115 | COMD1 | COMM domain-containing protein 1 | Q8N668 | COMMD1 |  | X | X |  |
| 1407 | 23257-14 | COMDA | COMM domain-containing protein 10 | Q9Y6G5 | COMMD10 |  |  |  |  |
| 1408 | 24704-38 | COMD5 | COMM domain-containing protein 5 | Q9GZQ3 | COMMD5 |  |  |  |  |
| 1409 | 20396-70 | COMD6 | COMM domain-containing protein 6 | Q7Z4G1 | COMMD6 |  |  |  |  |
| 1410 | 2823-7 | COMMD7 | COMM domain-containing protein 7 | Q86VX2 | COMMD7 |  |  |  |  |
| 1411 | 21507-48 | COMD8 | COMM domain-containing protein 8 | Q9NX08 | COMMD8 |  |  |  |  |

| # | **Custom Panel (X)** | **SOMAmer SeqID** | **Target Name** | **Human Target or Analyte** | **UniProt ID** | **GeneID** | **Cardiovascular Disease** | **Inflammation and Immune**  **Response** | **Metabolic Disease** | **Oncology** |
| --- | --- | --- | --- | --- | --- | --- | --- | --- | --- | --- |

| 1412 | 17683-2 | COMD9 | COMM domain-containing protein 9 | Q9P000 | COMMD9 |  | | | |
| --- | --- | --- | --- | --- | --- | --- | --- | --- | --- |
| 1413 | 16605-2 | C1T9A | Complement C1q and tumor necrosis factor-related protein 9A | P0C862 | C1QTNF9 |
| 1414 | 6019-12 | C1T9A | Complement C1q and tumor necrosis factor-related protein 9A | P0C862 | C1QTNF9 |
| 1415 | 2753-2 | C1q | Complement C1q subcomponent | P02745|P02746|P02747 | C1QA|C1QB|C1QC | X | X |  | X |
| 1416 | 14100-63 | C1QC | Complement C1q subcomponent subunit C | P02747 | C1QC |  | X |  |  |
| 1417 | 6304-8 | C1QT1 | Complement C1q tumor necrosis factor-related protein 1 | Q9BXJ1 | C1QTNF1 |  |  |  |  |
| 1418 | 7251-64 | C1QT3 | Complement C1q tumor necrosis factor-related protein 3 | Q9BXJ4 | C1QTNF3 |  |  |  |  |
| 1419 | 21567-214 | C1QT4 | Complement C1q tumor necrosis factor-related protein 4 | Q9BXJ3 | C1QTNF4 |  |  |  |  |
| 1420 | 23200-25 | C1QT4 | Complement C1q tumor necrosis factor-related protein 4 | Q9BXJ3 | C1QTNF4 |  |  |  |  |
| 1421 | 25964-12 | C1QT4 | Complement C1q tumor necrosis factor-related protein 4 | Q9BXJ3 | C1QTNF4 |  |  |  |  |
| 1422 | 7810-20 | C1QTNF5 | Complement C1q tumor necrosis factor-related protein 5 | Q9BXJ0 | C1QTNF5 |  |  |  |  |
| 1423 | 15670-15 | C1QL2 | Complement C1q-like protein 2 | Q7Z5L3 | C1QL2 |  |  |  |  |
| 1424 | 6423-66 | C1QL2 | Complement C1q-like protein 2 | Q7Z5L3 | C1QL2 |  |  |  |  |
| 1425 | 21707-15 | C1QL3 | Complement C1q-like protein 3 | Q5VWW1 | C1QL3 |  |  |  |  |
| 1426 | 7132-55 | C1QL4 | Complement C1q-like protein 4 | Q86Z23 | C1QL4 |  |  |  |  |
| 1427 | 3285-23 | C1r | Complement C1r subcomponent | P00736 | C1R | X | X |  |  |
| 1428 | 9348-1 | C1RL1 | Complement C1r subcomponent-like protein | Q9NZP8 | C1RL |  |  |  |  |
| 1429 | 20203-45 | C1s | Complement C1s subcomponent | P09871 | C1S | X | X |  |  |
| 1430 | 8840-61 | C1s | Complement C1s subcomponent | P09871 | C1S |  |  |  |  |
| 1431 | 3186-2 | C2 | Complement C2 | P06681 | C2 | X | X | X |  |
| 1432 | 2754-50 | C3 | Complement C3 | P01024 | C3 | X | X |  |  |
| 1433 | 4480-59 | C3b | Complement C3b | P01024 | C3 |  |  |  |  |
| 1434 | 2683-1 | iC3b | Complement C3b, inactivated | P01024 | C3 |  |  |  |  |
| 1435 | 5803-24 | C3d | Complement C3d fragment | P01024 | C3 |  |  |  |  |
| 1436 | 4481-34 | C4 | Complement C4 | P0C0L4|P0C0L5 | C4A|C4B | X | X |  |  |
| 1437 | 2182-54 | C4b | Complement C4b | P0C0L4|P0C0L5 | C4A|C4B |  |  |  |  |
| 1438 | 2381-52 | C5 | Complement C5 | P01031 | C5 |  | X |  |  |
| 1439 | 4482-66 | C5b, 6 Complex | Complement C5b-C6 complex | P01031|P13671 | C5|C6 |  | X | X |  |
| 1440 | 4967-1 | C1QBP | Complement component 1 Q subcomponent-binding protein, mitochondrial | Q07021 | C1QBP |  |  |  | X |
| 1441 | 11200-52 | C1QR1 | Complement component C1q receptor | Q9NPY3 | CD93 |  |  |  |  |
| 1442 | 14136-234 | C1QR1 | Complement component C1q receptor | Q9NPY3 | CD93 | X |  |  |  |
| 1443 | 4127-75 | C6 | Complement component C6 | P13671 | C6 | X | X |  |  |
| 1444 | 13731-14 | C7 | Complement component C7 | P10643 | C7 |  | X |  |  |
| 1445 | 2888-49 | C7 | Complement component C7 | P10643 | C7 |  |  |  |  |
| 1446 | 2429-27 | C8 | Complement component C8 | P07357|P07358|P07360 | C8A|C8B|C8G |  | X |  |  |
| 1447 | 14708-59 | CO8G | Complement component C8 gamma chain | P07360 | C8G |  | X |  |  |
| 1448 | 13722-105 | C9 | Complement component C9 | P02748 | C9 |  | X |  |  |
| 1449 | 3060-43 | C9 | Complement component C9 | P02748 | C9 |  |  |  |  |
| 1450 | 5069-9 | DAF | Complement decay-accelerating factor | P08174 | CD55 |  | X |  |  |
| 1451 | 4129-72 | Factor B | Complement factor B | P00751 | CFB |  | X |  |  |
| 1452 | 13678-169 | Factor D | Complement factor D | P00746 | CFD | X | X |  |  |
| 1453 | 2946-52 | Factor D | Complement factor D | P00746 | CFD |  |  |  |  |
| 1454 | 4159-130 | Factor H | Complement factor H | P08603 | CFH |  | X |  |  |
| 1455 | 15468-14 | FHR1 | Complement factor H-related protein 1 | Q03591 | CFHR1 |  | X |  |  |
| 1456 | 5982-50 | FHR1 | Complement factor H-related protein 1 | Q03591 | CFHR1 |  |  |  |  |
| 1457 | 15584-9 | FHR2 | Complement factor H-related protein 2 | P36980 | CFHR2 |  | X |  |  |
| 1458 | 16836-1 | FHR3 | Complement factor H-related protein 3 | Q02985 | CFHR3 |  | X |  |  |
| 1459 | 6471-53 | FHR4 | Complement factor H-related protein 4 | Q92496 | CFHR4 |  | X |  |  |
| 1460 | 16055-3 | complement factor H-related 5 | Complement factor H-related protein 5 | Q9BXR6 | CFHR5 |  |  |  |  |
| 1461 | 3666-17 | complement factor H-related 5 | Complement factor H-related protein 5 | Q9BXR6 | CFHR5 | X | X | X |  |
| 1462 | 7885-17 | complement factor H-related 5 | Complement factor H-related protein 5 | Q9BXR6 | CFHR5 |  |  |  |  |
| 1463 | 2567-5 | Factor I | Complement factor I | P05156 | CFI |  | X |  |  |
| 1464 | 19556-12 | Complement receptor type 1 | Complement receptor type 1 | P17927 | CR1 |  | X |  |  |
| 1465 | 15570-99 | Complement receptor type 2 | Complement receptor type 2 | P20023 | CR2 | X | X | X |  |
| 1466 | 21107-5 | CIA30 | Complex I intermediate-associated protein 30, mitochondrial | Q9Y375 | NDUFAF1 |  |  | X |  |
| 1467 | 18332-17 | CPLX1 | Complexin-1 | O14810 | CPLX1 |  |  |  |  |
| 1468 | 15321-8 | CPLX2 | Complexin-2 | Q6PUV4 | CPLX2 |  |  |  |  |
| 1469 | 21536-65 | CPLX3 | Complexin-3 | Q8WVH0 | CPLX3 |  |  |  |  |
| 1470 | 2975-19 | CTGF | Connective tissue growth factor | P29279 | CCN2 | X | X | X | X |
| 1471 | 4544-4 | CTAP-III | Connective tissue-activating peptide III | P02775 | PPBP |  |  |  |  |
| 1472 | 9543-131 | COG8 | Conserved oligomeric Golgi complex subunit 8 | Q96MW5 | COG8 |  |  | X |  |
| 1473 | 2974-61 | contactin-1 | Contactin-1 | Q12860 | CNTN1 | X |  |  |  |
| 1474 | 3296-92 | CNTN2 | Contactin-2 | Q02246 | CNTN2 |  |  |  |  |
| 1475 | 20586-18 | CNTN3 | Contactin-3 | Q9P232 | CNTN3 |  | X |  |  |
| 1476 | 3298-52 | Contactin-4 | Contactin-4 | Q8IWV2 | CNTN4 |  |  |  |  |
| 1477 | 3299-29 | Contactin-5 | Contactin-5 | O94779 | CNTN5 |  |  |  |  |
| 1478 | 20561-15 | CNTN6 | Contactin-6 | Q9UQ52 | CNTN6 |  |  |  |  |
| 1479 | 6965-19 | CNTP2 | Contactin-associated protein-like 2 | Q9UHC6 | CNTNAP2 |  | X |  |  |
| 1480 | 9044-1 | CNTP5 | Contactin-associated protein-like 5 | Q8WYK1 | CNTNAP5 |  |  |  |  |
| 1481 | 14029-42 | CSN2 | COP9 signalosome complex subunit 2 | P61201 | COPS2 |  |  |  |  |
| 1482 | 24725-4 | CSN5 | COP9 signalosome complex subunit 5 | Q92905 | COPS5 |  | X |  |  |
| 1483 | 12384-92 | CSN7B | COP9 signalosome complex subunit 7b | Q9H9Q2 | COPS7B |  |  |  |  |
| 1484 | 22119-18 | CSN8 | COP9 signalosome complex subunit 8 | Q99627 | COPS8 |  |  |  |  |
| 1485 | 5346-24 | CPNE1:C2, 1 and 2 | Copine-1:Ca2+-dependent membrane-targeting module domains 1 and 2 | Q99829 | CPNE1 |  |  |  |  |
| 1486 | 11602-12 | CPNE1:VWFA | Copine-1:Von Willebrand factor type A domain | Q99829 | CPNE1 |  |  |  |  |

| # | **Custom Panel (X)** | **SOMAmer SeqID** | **Target Name** | **Human Target or Analyte** | **UniProt ID** | **GeneID** | **Cardiovascular Disease** | **Inflammation and Immune**  **Response** | **Metabolic Disease** | **Oncology** |
| --- | --- | --- | --- | --- | --- | --- | --- | --- | --- | --- |

| 1487 | 23689-52 | CPNE6 | Copine-6 | O95741 | CPNE6 |  |  | | |
| --- | --- | --- | --- | --- | --- | --- | --- | --- | --- |
| 1488 | 24277-22 | CPNE7 | Copine-7 | Q9UBL6 | CPNE7 |  |
| 1489 | 13068-139 | CCS | Copper chaperone for superoxide dismutase | O14618 | CCS |  |
| 1490 | 15315-64 | CUTC | Copper homeostasis protein cutC homolog | Q9NTM9 | CUTC |  |
| 1491 | 19233-75 | ATOX1 | Copper transport protein ATOX1 | O00244 | ATOX1 | X |
| 1492 | 21664-6 | H2AY | Core histone macro-H2A.1 | O75367 | MACROH2A1 |  |
| 1493 | 10048-7 | PEBB | Core-binding factor subunit beta | Q13951 | CBFB | X |  |  | X |
| 1494 | 7085-81 | CDSN | Corneodesmosin | Q15517 | CDSN |  | X |  |  |
| 1495 | 17738-7 | CRNN | Cornulin | Q9UBG3 | CRNN |  |  |  | X |
| 1496 | 19637-9 | CRH | Corticoliberin | P06850 | CRH | X |  |  |  |
| 1497 | 5614-44 | CRH | Corticoliberin | P06850 | CRH |  |  |  |  |
| 1498 | 21967-20 | DHI1 | Corticosteroid 11-beta-dehydrogenase isozyme 1 | P28845 | HSD11B1 | X | X | X |  |
| 1499 | 4785-30 | CBG | Corticosteroid-binding globulin | P08185 | SERPINA6 |  |  |  |  |
| 1500 | 4890-10 | ACTH | Corticotropin | P01189 | POMC | X | X |  | X |
| 1501 | 6039-24 | CRHBP | Corticotropin-releasing factor-binding protein | P24387 | CRHBP |  |  |  |  |
| 1502 | 11204-80 | CXAR | Coxsackievirus and adenovirus receptor | P78310 | CXADR |  | X |  |  |
| 1503 | 24706-73 | CFDP1 | Craniofacial development protein 1 | Q9UEE9 | CFDP1 |  |  |  |  |
| 1504 | 4337-49 | CRP | C-reactive protein | P02741 | CRP | X | X | X |  |
| 1505 | 3800-71 | CK-BB | Creatine kinase B-type | P12277 | CKB | X |  |  | X |
| 1506 | 2670-67 | CK-MM | Creatine kinase M-type | P06732 | CKM | X |  |  |  |
| 1507 | 3714-49 | CK-MB | Creatine kinase M-type:Creatine kinase B-type heterodimer | P12277|P06732 | CKB|CKM | X |  |  | X |
| 1508 | 18197-97 | KCRS | Creatine kinase S-type, mitochondrial | P17540 | CKMT2 |  |  |  |  |
| 1509 | 15542-19 | KCRU | Creatine kinase U-type, mitochondrial | P12532 | CKMT1A |  |  |  |  |
| 1510 | 21134-9 | ZHANG | CREB/ATF bZIP transcription factor | Q9NS37 | CREBZF |  |  |  |  |
| 1511 | 13614-6 | CREB-binding protein | CREB-binding protein | Q92793 | CREBBP |  | X | X | X |
| 1512 | 25901-3 | CRTC3 | CREB-regulated transcription coactivator 3 | Q6UUV7 | CRTC3 |  |  |  |  |
| 1513 | 9877-28 | CRKL | Crk-like protein | P46109 | CRKL | X |  |  |  |
| 1514 | 3294-55 | CFC1 | Cryptic protein | P0CG37 | CFC1 | X |  |  |  |
| 1515 | 20443-37 | TEN1L | CST complex subunit TEN1 | Q86WV5 | TEN1 |  |  |  |  |
| 1516 | 20929-4 | CTDSL | CTD small phosphatase-like protein | O15194 | CTDSPL |  |  |  |  |
| 1517 | 11217-16 | CTBP1 | C-terminal-binding protein 1 | Q13363 | CTBP1 |  |  |  |  |
| 1518 | 23705-42 | PYRG1 | CTP synthase 1 | P17812 | CTPS1 |  |  |  |  |
| 1519 | 4332-6 | CLC1B | C-type lectin domain family 1 member B | Q9P126 | CLEC1B |  |  |  |  |
| 1520 | 10955-4 | CLC10 | C-type lectin domain family 10 member A | Q8IUN9 | CLEC10A | X |  |  |  |
| 1521 | 11187-11 | CL12A | C-type lectin domain family 12 member A | Q5QGZ9 | CLEC12A |  | X |  |  |
| 1522 | 10953-14 | CLC2A | C-type lectin domain family 2 member A | Q6UVW9 | CLEC2A |  |  |  |  |
| 1523 | 7786-83 | CLC2B | C-type lectin domain family 2 member B | Q92478 | CLEC2B |  |  |  |  |
| 1524 | 7054-87 | CLC2D | C-type lectin domain family 2 member D | Q9UHP7 | CLEC2D |  |  |  |  |
| 1525 | 8242-9 | CLC2L | C-type lectin domain family 2 member L | P0C7M8 | CLEC2L |  |  |  |  |
| 1526 | 8853-2 | CLC4A | C-type lectin domain family 4 member A | Q9UMR7 | CLEC4A |  | X |  |  |
| 1527 | 9094-5 | CLC4C | C-type lectin domain family 4 member C | Q8WTT0 | CLEC4C |  |  |  |  |
| 1528 | 7752-31 | CLC4D | C-type lectin domain family 4 member D | Q8WXI8 | CLEC4D |  | X |  |  |
| 1529 | 10781-19 | CLC4G | C-type lectin domain family 4 member G | Q6UXB4 | CLEC4G |  | X |  |  |
| 1530 | 19765-17 | CLC4G | C-type lectin domain family 4 member G | Q6UXB4 | CLEC4G |  |  |  |  |
| 1531 | 3361-26 | CLC4K | C-type lectin domain family 4 member K | Q9UJ71 | CD207 |  |  |  |  |
| 1532 | 3030-3 | DC-SIGNR | C-type lectin domain family 4 member M | Q9H2X3 | CLEC4M |  |  |  |  |
| 1533 | 6911-103 | CLC6A | C-type lectin domain family 6 member A | Q6EIG7 | CLEC6A |  |  |  |  |
| 1534 | 3603-60 | CLC7A | C-type lectin domain family 7 member A | Q9BXN2 | CLEC7A |  | X |  |  |
| 1535 | 20575-82 | CLC9A | C-type lectin domain family 9 member A | Q6UXN8 | CLEC9A |  |  |  |  |
| 1536 | 3041-55 | MRC2 | C-type mannose receptor 2 | Q9UBG0 | MRC2 |  |  |  | X |
| 1537 | 20430-8 | Natriuretic Peptide C-Type | C-type natriuretic peptide | P23582 | NPPC |  |  |  |  |
| 1538 | 9598-23 | CSMD1 | CUB and sushi domain-containing protein 1 | Q96PZ7 | CSMD1 |  |  |  |  |
| 1539 | 9971-5 | CSMD2 | CUB and sushi domain-containing protein 2 | Q7Z408 | CSMD2 |  |  |  |  |
| 1540 | 7943-16 | CUZD1 | CUB and zona pellucida-like domain-containing protein 1 | Q86UP6 | CUZD1 |  |  |  |  |
| 1541 | 16818-200 | CDCP1 | CUB domain-containing protein 1 | Q9H5V8 | CDCP1 |  |  |  |  |
| 1542 | 6565-68 | CDCP1 | CUB domain-containing protein 1 | Q9H5V8 | CDCP1 |  | X |  |  |
| 1543 | 8589-13 | CDCP1 | CUB domain-containing protein 1 | Q9H5V8 | CDCP1 |  |  |  |  |
| 1544 | 12904-180 | cubilin | cubilin | O60494 | CUBN |  |  | X |  |
| 1545 | 21149-27 | CUED1 | CUE domain-containing protein 1 | Q9NWM3 | CUEDC1 |  |  |  |  |
| 1546 | 7245-2 | CELF2 | CUGBP Elav-like family member 2 | O95319 | CELF2 |  | X |  |  |
| 1547 | 17797-1 | CUL1 | Cullin-1 | Q13616 | CUL1 |  | X |  |  |
| 1548 | 10045-47 | CUL3 | Cullin-3 | Q13618 | CUL3 |  |  |  |  |
| 1549 | 13743-56 | CUL4B | Cullin-4B | Q13620 | CUL4B |  |  |  |  |
| 1550 | 12991-49 | CUL9 | Cullin-9 | Q8IWT3 | CUL9 |  |  |  |  |
| 1551 | 13937-75 | CAND1 | Cullin-associated NEDD8-dissociated protein 1 | Q86VP6 | CAND1 |  |  |  |  |
| 1552 | 23686-44 | C19L1 | CWF19-like protein 1 | Q69YN2 | CWF19L1 |  |  |  |  |
| 1553 | 21133-33 | CMC4 | Cx9C motif-containing protein 4 | P56277 | CMC4 |  |  |  |  |
| 1554 | 9585-80 | ACAM:CD | CXADR-like membrane protein:Cytoplasmic domain | Q9H6B4 | CLMP |  |  |  |  |
| 1555 | 10440-26 | ACAM:ECD | CXADR-like membrane protein:Extracellular domain | Q9H6B4 | CLMP |  |  |  |  |
| 1556 | 4141-79 | IP-10 | C-X-C motif chemokine 10 | P02778 | CXCL10 | X | X | X | X |
| 1557 | 18171-25 | I-TAC | C-X-C motif chemokine 11 | O14625 | CXCL11 |  |  |  |  |
| 1558 | 3038-9 | I-TAC | C-X-C motif chemokine 11 | O14625 | CXCL11 |  | X | X |  |
| 1559 | 13701-2 | BLC | C-X-C motif chemokine 13 | O43927 | CXCL13 |  | X |  | X |
| 1560 | 3487-32 | BLC | C-X-C motif chemokine 13 | O43927 | CXCL13 |  |  |  |  |
| 1561 | 5730-60 | BRAK | C-X-C motif chemokine 14 | O95715 | CXCL14 |  | X |  | X |

| # | **Custom Panel (X)** | **SOMAmer SeqID** | **Target Name** | **Human Target or Analyte** | **UniProt ID** | **GeneID** | **Cardiovascular Disease** | **Inflammation and Immune**  **Response** | **Metabolic Disease** | **Oncology** |
| --- | --- | --- | --- | --- | --- | --- | --- | --- | --- | --- |

| 1562 | 2436-49 | CXCL16, soluble | C-X-C motif chemokine 16 | Q9H2A7 | CXCL16 | X |  |  | |
| --- | --- | --- | --- | --- | --- | --- | --- | --- | --- |
| 1563 | 9495-10 | VCC1 | C-X-C motif chemokine 17 | Q6UXB2 | CXCL17 |  |  |
| 1564 | 2979-8 | ENA-78 | C-X-C motif chemokine 5 | P42830 | CXCL5 |  | X |
| 1565 | 3495-15 | GCP-2 | C-X-C motif chemokine 6 | P80162 | CXCL6 |  | X |
| 1566 | 11593-21 | MIG | C-X-C motif chemokine 9 | Q07325 | CXCL9 |  |  |
| 1567 | 9188-119 | MIG | C-X-C motif chemokine 9 | Q07325 | CXCL9 | X | X | X | X |
| 1568 | 20926-31 | ATF1 | Cyclic AMP-dependent transcription factor ATF-1 | P18846 | ATF1 |  |  |  |  |
| 1569 | 22075-16 | ATF3 | Cyclic AMP-dependent transcription factor ATF-3 | P18847 | ATF3 | X | X | X | X |
| 1570 | 22365-52 | ATF5 | Cyclic AMP-dependent transcription factor ATF-5 | Q9Y2D1 | ATF5 |  |  |  |  |
| 1571 | 23771-17 | ATF5 | Cyclic AMP-dependent transcription factor ATF-5 | Q9Y2D1 | ATF5 |  |  |  |  |
| 1572 | 11277-23 | ATF6A | Cyclic AMP-dependent transcription factor ATF-6 alpha | P18850 | ATF6 |  | X |  |  |
| 1573 | 11387-3 | ATF6B | Cyclic AMP-dependent transcription factor ATF-6 beta | Q99941 | ATF6B |  |  |  |  |
| 1574 | 11198-37 | CR3L1 | Cyclic AMP-responsive element-binding protein 3-like protein 1 | Q96BA8 | CREB3L1 |  |  |  |  |
| 1575 | 17336-54 | CR3L2 | Cyclic AMP-responsive element-binding protein 3-like protein 2 | Q70SY1 | CREB3L2 |  |  |  |  |
| 1576 | 11308-8 | CR3L4 | Cyclic AMP-responsive element-binding protein 3-like protein 4 | Q8TEY5 | CREB3L4 |  |  |  |  |
| 1577 | 24462-4 | CNGB1 | Cyclic nucleotide-gated cation channel beta-1 | Q14028 | CNGB1 |  |  |  |  |
| 1578 | 12872-35 | CNGA2 | Cyclic nucleotide-gated olfactory channel | Q16280 | CNGA2 |  |  |  |  |
| 1579 | 15319-226 | CCNA1 | Cyclin-A1 | P78396 | CCNA1 |  | X |  |  |
| 1580 | 15574-37 | Cyclin A | Cyclin-A2 | P20248 | CCNA2 |  |  |  |  |
| 1581 | 3422-4 | CDK1/cyclin B | Cyclin-dependent kinase 1:G2/mitotic-specific cyclin-B1 complex | P06493|P14635 | CDK1|CCNB1 |  | X |  | X |
| 1582 | 21581-87 | CDK15 | Cyclin-dependent kinase 15; EC=2.7.11.22 | Q96Q40 | CDK15 |  |  |  |  |
| 1583 | 16867-76 | PCTK1 | Cyclin-dependent kinase 16 | Q00536 | CDK16 |  |  |  |  |
| 1584 | 3357-67 | CDK2/cyclin A | Cyclin-dependent kinase 2:Cyclin-A2 complex | P24941|P20248 | CDK2|CCNA2 |  |  |  | X |
| 1585 | 22103-25 | CDK20 | Cyclin-dependent kinase 20 | Q8IZL9 | CDK20 |  |  |  |  |
| 1586 | 9450-18 | CDKA1 | Cyclin-dependent kinase 2-associated protein 1 | O14519 | CDK2AP1 |  |  |  |  |
| 1587 | 18227-3 | CDKA2 | Cyclin-dependent kinase 2-associated protein 2 | O75956 | CDK2AP2 |  |  |  |  |
| 1588 | 22952-28 | CDKA2 | Cyclin-dependent kinase 2-associated protein 2 | O75956 | CDK2AP2 |  |  |  |  |
| 1589 | 19383-131 | CINP | Cyclin-dependent kinase 2-interacting protein | Q9BW66 | CINP |  |  |  |  |
| 1590 | 9874-28 | p15-INK4b | Cyclin-dependent kinase 4 inhibitor B | P42772 | CDKN2B | X | X |  | X |
| 1591 | 12521-3 | CDN2C | Cyclin-dependent kinase 4 inhibitor C | P42773 | CDKN2C | X | X |  | X |
| 1592 | 12376-85 | p19-INK4d | Cyclin-dependent kinase 4 inhibitor D | P55273 | CDKN2D |  |  |  |  |
| 1593 | 3358-51 | CDK5/p35 | Cyclin-dependent kinase 5:Cyclin-dependent kinase 5 activator 1 complex | Q00535|Q15078 | CDK5|CDK5R1 |  |  |  |  |
| 1594 | 3359-11 | CDK8/cyclin C | Cyclin-dependent kinase 8:Cyclin-C complex | P49336|P24863 | CDK8|CCNC | X |  |  |  |
| 1595 | 18291-8 | p21 | Cyclin-dependent kinase inhibitor 1 | P38936 | CDKN1A | X | X |  | X |
| 1596 | 3719-2 | p27Kip1 | Cyclin-dependent kinase inhibitor 1B | P46527 | CDKN1B |  | X | X | X |
| 1597 | 14178-18 | CDKN3 | Cyclin-dependent kinase inhibitor 3 | Q16667 | CDKN3 |  |  |  |  |
| 1598 | 21786-25 | CDKL2 | Cyclin-dependent kinase-like 2 | Q92772 | CDKL2 |  |  |  |  |
| 1599 | 12530-14 | CKS-1 | Cyclin-dependent kinases regulatory subunit 1 | P61024 | CKS1B |  |  |  |  |
| 1600 | 18913-3 | Cyclin H | Cyclin-H | P51946 | CCNH |  |  |  |  |
| 1601 | 9848-22 | Cyclin H | Cyclin-H | P51946 | CCNH |  |  |  | X |
| 1602 | 22098-10 | CCYL1 | Cyclin-Y-like protein 1 | Q8N7R7 | CCNYL1 |  |  |  |  |
| 1603 | 10086-39 | CBS | Cystathionine beta-synthase | P35520 | CBS | X | X | X | X |
| 1604 | 21599-6 | CGL | Cystathionine gamma-lyase | P32929 | CTH |  |  | X |  |
| 1605 | 19768-13 | Cystatin B | Cystatin B | P04080 | CSTB | X |  |  | X |
| 1606 | 10572-65 | CST8 | Cystatin-8 | O60676 | CST8 |  |  |  |  |
| 1607 | 2609-59 | Cystatin C | Cystatin-C | P01034 | CST3 | X |  | X | X |
| 1608 | 13661-193 | CYTD | Cystatin-D | P28325 | CST5 |  |  |  |  |
| 1609 | 3803-10 | CYTD | Cystatin-D | P28325 | CST5 |  |  |  |  |
| 1610 | 14038-130 | CYTF | Cystatin-F | O76096 | CST7 |  |  |  |  |
| 1611 | 3302-58 | CYTF | Cystatin-F | O76096 | CST7 |  |  |  |  |
| 1612 | 6368-9 | CST1L | Cystatin-like 1 | Q9H114 | CSTL1 |  |  |  |  |
| 1613 | 14711-27 | Cystatin M | Cystatin-M | Q15828 | CST6 |  |  |  |  |
| 1614 | 3303-23 | Cystatin M | Cystatin-M | Q15828 | CST6 |  |  |  | X |
| 1615 | 14076-74 | Cystatin-S | Cystatin-S | P01036 | CST4 |  |  |  |  |
| 1616 | 4324-33 | CYTT | Cystatin-SA | P09228 | CST2 |  |  |  |  |
| 1617 | 5459-33 | CYTN | Cystatin-SN | P01037 | CST1 |  | X |  | X |
| 1618 | 15529-33 | Cysteine-rich protein 1 | Cysteine and glycine-rich protein 1 | P21291 | CSRP1 |  |  |  |  |
| 1619 | 12968-2 | CSRP2 | Cysteine and glycine-rich protein 2 | Q16527 | CSRP2 |  |  |  |  |
| 1620 | 9171-11 | CSRP3 | Cysteine and glycine-rich protein 3 | P50461 | CSRP3 | X |  |  |  |
| 1621 | 21436-56 | ATG4A | Cysteine protease ATG4A | Q8WYN0 | ATG4A |  |  |  |  |
| 1622 | 13629-25 | ATG4B | Cysteine protease ATG4B | Q9Y4P1 | ATG4B |  |  |  |  |
| 1623 | 25078-13 | ATG4C | Cysteine protease ATG4C | Q96DT6 | ATG4C |  |  |  |  |
| 1624 | 8699-43 | CRIM1:CD | Cysteine-rich motor neuron 1 protein:Cytoplasmic domain | Q9NZV1 | CRIM1 |  |  |  |  |
| 1625 | 15492-1 | CRIM1:ECD | Cysteine-rich motor neuron 1 protein:Extracellular domain | Q9NZV1 | CRIM1 |  |  |  |  |
| 1626 | 6502-50 | CRIM1:ECD | Cysteine-rich motor neuron 1 protein:Extracellular domain | Q9NZV1 | CRIM1 |  |  |  |  |
| 1627 | 19294-26 | CRIPT | Cysteine-rich PDZ-binding protein | Q9P021 | CRIPT |  |  |  |  |
| 1628 | 18275-5 | CRIP1 | Cysteine-rich protein 1 | P50238 | CRIP1 |  |  |  |  |
| 1629 | 9053-16 | CRIP2 | Cysteine-rich protein 2 | P52943 | CRIP2 |  |  |  |  |
| 1630 | 9282-12 | CRIS2 | Cysteine-rich secretory protein 2 | P16562 | CRISP2 |  |  |  |  |
| 1631 | 3187-52 | CRIS3 | Cysteine-rich secretory protein 3 | P54108 | CRISP3 |  |  |  |  |
| 1632 | 5691-2 | CRLD2 | Cysteine-rich secretory protein LCCL domain-containing 2 | Q9H0B8 | CRISPLD2 |  |  |  |  |
| 1633 | 25220-8 | CRTP1 | Cysteine-rich tail protein 1 | A8MQ03 | CYSRT1 |  |  |  |  |
| 1634 | 7628-40 | CREL1 | Cysteine-rich with EGF-like domain protein 1 | Q96HD1 | CRELD1 | X |  |  |  |
| 1635 | 14098-28 | SYCC | Cysteine--tRNA ligase, cytoplasmic | P49589 | CARS1 |  |  |  |  |
| 1636 | 7717-95 | CFTR | Cystic fibrosis transmembrane conductance regulator | P13569 | CFTR |  | X |  | X |

| # | **Custom Panel (X)** | **SOMAmer SeqID** | **Target Name** | **Human Target or Analyte** | **UniProt ID** | **GeneID** | **Cardiovascular Disease** | **Inflammation and Immune**  **Response** | **Metabolic Disease** | **Oncology** |
| --- | --- | --- | --- | --- | --- | --- | --- | --- | --- | --- |

| 1637 | 18336-31 | CDA | Cytidine deaminase | P32320 | CDA |  |  |  | X |
| --- | --- | --- | --- | --- | --- | --- | --- | --- | --- |
| 1638 | 24682-35 | CYBR1 | Cytochrome b reductase 1 | Q53TN4 | CYBRD1 |  |  | X |  |
| 1639 | 11287-14 | Cytochrome b5 | Cytochrome b5 | P00167 | CYB5A |  |  | X |  |
| 1640 | 24959-20 | NB5R4 | Cytochrome b5 reductase 4 | Q7L1T6 | CYB5R4 |  |  |  |  |
| 1641 | 13377-3 | C56D1 | Cytochrome b561 domain-containing protein 1 | Q8N8Q1 | CYB561D1 |  |  |  |  |
| 1642 | 12957-62 | UCR6 | Cytochrome b-c1 complex subunit 7 | P14927 | UQCRB |  |  | X |  |
| 1643 | 2942-50 | Cytochrome c | Cytochrome c | P99999 | CYCS | X |  | X |  |
| 1644 | 7888-58 | CCD56 | Cytochrome c oxidase assembly factor 3 homolog, mitochondrial | Q9Y2R0 | COA3 |  |  | X |  |
| 1645 | 20457-13 | COA4 | Cytochrome c oxidase assembly factor 4 homolog, mitochondrial | Q9NYJ1 | COA4 |  |  |  |  |
| 1646 | 23258-56 | COA7 | Cytochrome c oxidase assembly factor 7 | Q96BR5 | COA7 |  |  |  |  |
| 1647 | 23575-58 | COX19 | Cytochrome c oxidase assembly protein COX19 | Q49B96 | COX19 |  |  |  |  |
| 1648 | 7850-1 | COX42 | Cytochrome c oxidase subunit 4 isoform 2, mitochondrial | Q96KJ9 | COX4I2 |  |  | X |  |
| 1649 | 18226-148 | COX5A | Cytochrome c oxidase subunit 5A, mitochondrial | P20674 | COX5A |  |  | X |  |
| 1650 | 7887-57 | COX5B | Cytochrome c oxidase subunit 5B, mitochondrial | P10606 | COX5B | X |  |  |  |
| 1651 | 10535-25 | CX6A2 | Cytochrome c oxidase subunit 6A2, mitochondrial | Q02221 | COX6A2 |  |  |  |  |
| 1652 | 8903-1 | COX6C | Cytochrome c oxidase subunit 6C | P09669 | COX6C |  |  |  |  |
| 1653 | 8390-25 | CX7A1 | Cytochrome c oxidase subunit 7A1, mitochondrial | P24310 | COX7A1 |  |  |  |  |
| 1654 | 9590-10 | COX7R | Cytochrome c oxidase subunit 7A-related protein, mitochondrial | O14548 | COX7A2L |  |  |  |  |
| 1655 | 24453-75 | CCHL | Cytochrome c-type heme lyase | P53701 | HCCS |  |  |  |  |
| 1656 | 25464-1 | CP2CJ | Cytochrome P450 2C19 | P33261 | CYP2C19 | X |  | X |  |
| 1657 | 2943-5 | Cytochrome P450 3A4 | Cytochrome P450 3A4 | P08684 | CYP3A4 | X | X |  | X |
| 1658 | 7879-12 | Cytochrome P450 3A4 | Cytochrome P450 3A4 | P08684 | CYP3A4 |  |  |  |  |
| 1659 | 11546-7 | CYGB | Cytoglobin | Q8WWM9 | CYGB |  |  |  |  |
| 1660 | 21239-31 | CYH1 | Cytohesin-1 | Q15438 | CYTH1 | X |  |  |  |
| 1661 | 12533-135 | CYH2 | Cytohesin-2 | Q99418 | CYTH2 |  |  |  |  |
| 1662 | 21365-5 | CYH3 | Cytohesin-3 | O43739 | CYTH3 |  |  |  |  |
| 1663 | 12746-4 | CYH4 | Cytohesin-4 | Q9UIA0 | CYTH4 |  |  |  |  |
| 1664 | 13612-7 | CYTIP | Cytohesin-interacting protein | O60759 | CYTIP |  |  |  |  |
| 1665 | 11137-43 | IL3RB:CD | Cytokine receptor common subunit beta:Cytoplasmic domain | P32927 | CSF2RB |  |  |  |  |
| 1666 | 10512-13 | IL3RB:ECD | Cytokine receptor common subunit beta:Extracellular domain | P32927 | CSF2RB |  |  |  |  |
| 1667 | 2634-2 | IL-2 sRg | Cytokine receptor common subunit gamma | P31785 | IL2RG |  | X | X |  |
| 1668 | 14747-9 | CRLF1 | Cytokine receptor-like factor 1 | O75462 | CRLF1 |  |  |  |  |
| 1669 | 2607-54 | CLF-1/CLC Complex | Cytokine receptor-like factor 1:Cardiotrophin-like cytokine factor 1 Complex | O75462|Q9UBD9 | CRLF1|CLCF1 |  |  |  |  |
| 1670 | 13694-24 | TSLP R | Cytokine receptor-like factor 2 | Q9HC73 | CRLF2 |  | X |  | X |
| 1671 | 2746-56 | TSLP R | Cytokine receptor-like factor 2 | Q9HC73 | CRLF2 |  |  |  |  |
| 1672 | 25052-3 | CRLF3 | Cytokine receptor-like factor 3 | Q8IUI8 | CRLF3 |  |  |  |  |
| 1673 | 15617-8 | lymphotactin beta | Cytokine SCM-1 beta | Q9UBD3 | XCL2 |  |  |  |  |
| 1674 | 25501-128 | CLNK | Cytokine-dependent hematopoietic cell linker | Q7Z7G1 | CLNK |  |  |  |  |
| 1675 | 8402-22 | CYTL1 | Cytokine-like protein 1 | Q9NRR1 | CYTL1 |  |  |  |  |
| 1676 | 20054-28 | Aconitase 1 | Cytoplasmic aconitate hydratase | P21399 | ACO1 |  |  |  |  |
| 1677 | 24981-8 | DC1L1 | Cytoplasmic dynein 1 light intermediate chain 1 | Q9Y6G9 | DYNC1LI1 |  |  |  |  |
| 1678 | 25053-1 | DC1L2 | Cytoplasmic dynein 1 light intermediate chain 2 | O43237 | DYNC1LI2 |  |  |  |  |
| 1679 | 5014-49 | NCK1 | Cytoplasmic protein NCK1 | P16333 | NCK1 |  |  |  |  |
| 1680 | 13615-60 | NCK2 | Cytoplasmic protein NCK2 | O43639 | NCK2 |  |  |  |  |
| 1681 | 3414-40 | BMX | Cytoplasmic tyrosine-protein kinase BMX | P51813 | BMX |  |  |  |  |
| 1682 | 5345-51 | CKAP2 | Cytoskeleton-associated protein 2 | Q8WWK9 | CKAP2 |  |  |  |  |
| 1683 | 7221-56 | CKAP4 | Cytoskeleton-associated protein 4 | Q07065 | CKAP4 |  |  |  |  |
| 1684 | 13959-7 | LAP | Cytosol aminopeptidase | P28838 | LAP3 |  |  |  |  |
| 1685 | 15610-72 | LAP | Cytosol aminopeptidase | P28838 | LAP3 |  |  |  | X |
| 1686 | 23362-26 | 5NT1A | Cytosolic 5'-nucleotidase 1A | Q9BXI3 | NT5C1A |  |  |  |  |
| 1687 | 19615-213 | 5NT3 | Cytosolic 5'-nucleotidase 3A | Q9H0P0 | NT5C3A |  |  |  | X |
| 1688 | 23385-18 | CAST1 | Cytosolic arginine sensor for mTORC1 subunit 1 | Q8WTX7 | CASTOR1 |  |  |  |  |
| 1689 | 21592-8 | GBA3 | Cytosolic beta-glucosidase | Q9H227 | GBA3 |  |  |  |  |
| 1690 | 16781-2 | ENASE | Cytosolic endo-beta-N-acetylglucosaminidase | Q8NFI3 | ENGASE |  |  |  |  |
| 1691 | 21863-11 | NUBP2 | Cytosolic Fe-S cluster assembly factor NUBP2 | Q9Y5Y2 | NUBP2 |  |  |  |  |
| 1692 | 3192-3 | Glutamate carboxypeptidase | Cytosolic non-specific dipeptidase | Q96KP4 | CNDP2 |  |  |  |  |
| 1693 | 21430-4 | cPLA2-alpha | Cytosolic phospholipase A2 alpha | P47712 | PLA2G4A |  | X | X | X |
| 1694 | 11288-26 | 5NTC | Cytosolic purine 5'-nucleotidase | P49902 | NT5C2 |  | X |  |  |
| 1695 | 7968-15 | CRTAM | Cytotoxic and regulatory T-cell molecule | O95727 | CRTAM |  |  |  |  |
| 1696 | 3022-4 | CTLA-4 | Cytotoxic T-lymphocyte protein 4 | P16410 | CTLA4 | X | X | X | X |
| 1697 | 15548-35 | SERA | D-3-phosphoglycerate dehydrogenase | O43175 | PHGDH |  |  |  |  |
| 1698 | 8986-2 | SERA | D-3-phosphoglycerate dehydrogenase | O43175 | PHGDH |  |  | X | X |
| 1699 | 19120-33 | OXDA | D-amino-acid oxidase | P14920 | DAO |  |  | X |  |
| 1700 | 25306-51 | DAXX | DAXX | Q9UER7 | DAXX |  |  |  | X |
| 1701 | 23401-3 | DAZP1 | DAZ-associated protein 1 | Q96EP5 | DAZAP1 |  |  |  |  |
| 1702 | 21685-29 | DCC | DCC | P43146 | DCC |  |  | X | X |
| 1703 | 12825-18 | DP13A | DCC-interacting protein 13-alpha | Q9UKG1 | APPL1 |  |  | X |  |
| 1704 | 17366-6 | DCNL1 | DCN1-like protein 1 | Q96GG9 | DCUN1D1 |  |  |  |  |
| 1705 | 19511-8 | DCNL2 | DCN1-like protein 2 | Q6PH85 | DCUN1D2 |  |  |  |  |
| 1706 | 13553-4 | DCNL3 | DCN1-like protein 3 | Q8IWE4 | DCUN1D3 |  |  |  |  |
| 1707 | 8760-10 | DCNL5 | DCN1-like protein 5 | Q9BTE7 | DCUN1D5 |  |  |  |  |
| 1708 | 4314-12 | XTP3A | dCTP pyrophosphatase 1 | Q9H773 | DCTPP1 |  |  |  |  |
| 1709 | 22120-4 | DCA11 | DDB1- and CUL4-associated factor 11 | Q8TEB1 | DCAF11 |  |  |  |  |
| 1710 | 22121-43 | DCA12 | DDB1- and CUL4-associated factor 12 | Q5T6F0 | DCAF12 |  |  |  |  |
| 1711 | 11283-13 | DCAF5 | DDB1- and CUL4-associated factor 5 | Q96JK2 | DCAF5 |  |  |  |  |

| # | **Custom Panel (X)** | **SOMAmer SeqID** | **Target Name** | **Human Target or Analyte** | **UniProt ID** | **GeneID** | **Cardiovascular Disease** | **Inflammation and Immune**  **Response** | **Metabolic Disease** | **Oncology** |
| --- | --- | --- | --- | --- | --- | --- | --- | --- | --- | --- |

| 1712 | 4907-56 | D-dimer | D-dimer | P02671|P02675|P02679 | FGA|FGB|FGG |  | | | |
| --- | --- | --- | --- | --- | --- | --- | --- | --- | --- |
| 1713 | 19237-17 | D-dopachrome decarboxylase | D-dopachrome decarboxylase | P30046 | DDT |
| 1714 | 23559-10 | DDRGK | DDRGK domain-containing protein 1 | Q96HY6 | DDRGK1 |
| 1715 | 15322-35 | CRADD | Death domain-containing protein CRADD | P78560 | CRADD |  |  |  | X |
| 1716 | 19141-22 | DAP1 | Death-associated protein 1 | P51397 | DAP |  |  |  |  |
| 1717 | 13955-33 | DAPK1 | Death-associated protein kinase 1 | P53355 | DAPK1 |  | X |  | X |
| 1718 | 4355-13 | DAPK2 | Death-associated protein kinase 2 | Q9UIK4 | DAPK2 |  |  |  |  |
| 1719 | 24261-202 | DAPK3 | Death-associated protein kinase 3 | O43293 | DAPK3 |  |  |  |  |
| 1720 | 14329-4 | DIDO1 | Death-inducer obliterator 1 | Q9BTC0 | DIDO1 |  |  |  |  |
| 1721 | 2666-53 | Bone proteoglycan II | Decorin | P07585 | DCN | X | X |  |  |
| 1722 | 25904-17 | DOCK2 | Dedicator of cytokinesis protein 2 | Q92608 | DOCK2 |  |  |  |  |
| 1723 | 14002-18 | DOCK9 | Dedicator of cytokinesis protein 9 | Q9BZ29 | DOCK9 |  |  |  |  |
| 1724 | 16785-45 | HD-5 | Defensin-5 | Q01523 | DEFA5 |  | X |  |  |
| 1725 | 6387-61 | HD-5 | Defensin-5 | Q01523 | DEFA5 |  |  |  |  |
| 1726 | 6369-82 | DEAF1 | Deformed epidermal autoregulatory factor 1 homolog | O75398 | DEAF1 |  |  |  |  |
| 1727 | 23317-10 | DHR11 | Dehydrogenase/reductase SDR family member 11 | Q6UWP2 | DHRS11 |  |  |  |  |
| 1728 | 21308-44 | DHRS4 | Dehydrogenase/reductase SDR family member 4 | Q9BTZ2 | DHRS4 |  |  |  |  |
| 1729 | 17467-1 | DHRS9 | Dehydrogenase/reductase SDR family member 9 | Q9BPW9 | DHRS9 |  |  |  |  |
| 1730 | 19212-4 | DMBT1 | Deleted in malignant brain tumors 1 protein | Q9UGM3 | DMBT1 |  |  |  |  |
| 1731 | 11442-1 | DNER:CD | Delta and Notch-like epidermal growth factor-related receptor:Cytoplasmic domain | Q8NFT8 | DNER |  |  |  |  |
| 1732 | 9769-48 | DNER:ECD | Delta and Notch-like epidermal growth factor-related receptor:Extracellular domain | Q8NFT8 | DNER |  |  |  |  |
| 1733 | 12662-82 | ECH1 | Delta(3,5)-Delta(2,4)-dienoyl-CoA isomerase, mitochondrial | Q13011 | ECH1 |  |  |  |  |
| 1734 | 15523-9 | HEM2 | Delta-aminolevulinic acid dehydratase | P13716 | ALAD | X |  | X | X |
| 1735 | 5349-69 | DLL1 | Delta-like protein 1 | O00548 | DLL1 |  |  |  | X |
| 1736 | 8264-43 | DLL1 | Delta-like protein 1 | O00548 | DLL1 |  |  |  |  |
| 1737 | 9974-8 | DLL3 | Delta-like protein 3 | Q9NYJ7 | DLL3 |  |  |  | X |
| 1738 | 3305-6 | DLL4 | Delta-like protein 4 | Q9NR61 | DLL4 |  |  |  | X |
| 1739 | 23650-2 | Dematin | Dematin | Q08495 | DMTN |  |  |  |  |
| 1740 | 19287-59 | DENR | Density-regulated protein | O43583 | DENR |  |  |  |  |
| 1741 | 21547-6 | DMP4 | Dentin matrix protein 4 | Q8IXL6 | FAM20C |  |  |  |  |
| 1742 | 21661-82 | DMP4 | Dentin matrix protein 4 | Q8IXL6 | FAM20C |  |  |  |  |
| 1743 | 9836-20 | DCK | Deoxycytidine kinase | P27707 | DCK |  |  |  |  |
| 1744 | 17756-69 | DCTD | Deoxycytidylate deaminase | P32321 | DCTD |  |  |  |  |
| 1745 | 24420-57 | DOHH | Deoxyhypusine hydroxylase | Q9BU89 | DOHH |  |  |  |  |
| 1746 | 9856-22 | DHYS | Deoxyhypusine synthase | P49366 | DHPS |  |  |  |  |
| 1747 | 11303-7 | SAMH1 | Deoxynucleoside triphosphate triphosphohydrolase SAMHD1 | Q9Y3Z3 | SAMHD1 | X | X | X |  |
| 1748 | 6324-11 | DNSL2 | Deoxyribonuclease-1-like 2 | Q92874 | DNASE1L2 |  |  |  |  |
| 1749 | 6533-20 | DNS2B | Deoxyribonuclease-2-beta | Q8WZ79 | DNASE2B |  |  |  |  |
| 1750 | 9995-6 | DUT | Deoxyuridine 5'-triphosphate nucleotidohydrolase, mitochondrial | P33316 | DUT |  |  |  |  |
| 1751 | 13393-46 | DERL1 | Derlin-1 | Q9BUN8 | DERL1 |  | X |  |  |
| 1752 | 4979-34 | DERM | Dermatopontin | Q07507 | DPT | X |  |  |  |
| 1753 | 5963-9 | Dermokine | Dermokine | Q6E0U4 | DMKN |  |  |  |  |
| 1754 | 8535-102 | Dermokine | Dermokine | Q6E0U4 | DMKN |  |  |  |  |
| 1755 | 4389-2 | DHH | Desert hedgehog protein N-product | O43323 | DHH |  |  |  |  |
| 1756 | 12030-82 | Desmin | Desmin | P17661 | DES | X |  | X | X |
| 1757 | 10882-12 | Desmocollin-1 | Desmocollin-1 | Q08554 | DSC1 |  |  |  |  |
| 1758 | 13126-52 | DSC2 | Desmocollin-2 | Q02487 | DSC2 | X |  |  |  |
| 1759 | 4981-6 | DSC3 | Desmocollin-3 | Q14574 | DSC3 |  |  |  | X |
| 1760 | 2976-58 | Desmoglein-1 | Desmoglein-1 | Q02413 | DSG1 |  | X | X |  |
| 1761 | 20517-1 | Desmoglein-2 | Desmoglein-2 | Q14126 | DSG2 |  |  |  |  |
| 1762 | 9484-75 | Desmoglein-2 | Desmoglein-2 | Q14126 | DSG2 | X |  |  |  |
| 1763 | 11310-8 | Desmoglein-3 | Desmoglein-3 | P32926 | DSG3 |  |  |  |  |
| 1764 | 16317-20 | Desmoglein-3 | Desmoglein-3 | P32926 | DSG3 |  |  |  |  |
| 1765 | 22568-7 | DSG4 | Desmoglein-4 | Q86SJ6 | DSG4 |  |  |  |  |
| 1766 | 18883-4 | DEST | Destrin | P60981 | DSTN |  |  |  |  |
| 1767 | 22958-6 | DEST | Destrin | P60981 | DSTN | X |  |  |  |
| 1768 | 21204-70 | DESI1 | Desumoylating isopeptidase 1 | Q6ICB0 | DESI1 |  |  |  |  |
| 1769 | 23187-9 | VCIP1 | Deubiquitinating protein VCIP135 | Q96JH7 | VCPIP1 |  |  |  |  |
| 1770 | 21589-80 | DPPA4 | Developmental pluripotency-associated protein 4 | Q7L190 | DPPA4 |  |  |  |  |
| 1771 | 17849-6 | DRG1 | Developmentally-regulated GTP-binding protein 1 | Q9Y295 | DRG1 |  |  |  |  |
| 1772 | 7808-5 | GLCE | D-glucuronyl C5-epimerase | O94923 | GLCE |  |  |  |  |
| 1773 | 3122-6 | SMAC | Diablo homolog, mitochondrial | Q9NR28 | DIABLO | X |  |  |  |
| 1774 | 12895-28 | DGKB | Diacylglycerol kinase beta | Q9Y6T7 | DGKB | X |  |  |  |
| 1775 | 9859-180 | SSAT-1 | Diamine acetyltransferase 1 | P21673 | SAT1 |  | X | X |  |
| 1776 | 12524-18 | SAT2 | Diamine acetyltransferase 2 | Q96F10 | SAT2 |  |  |  |  |
| 1777 | 16309-30 | Soggy-1 | Dickkopf-like protein 1 | Q9UK85 | DKKL1 |  |  |  |  |
| 1778 | 3644-5 | Soggy-1 | Dickkopf-like protein 1 | Q9UK85 | DKKL1 |  |  |  |  |
| 1779 | 3535-84 | DKK1 | Dickkopf-related protein 1 | O94907 | DKK1 | X |  | X | X |
| 1780 | 15678-71 | DKK2 | Dickkopf-related protein 2 | Q9UBU2 | DKK2 |  |  |  |  |
| 1781 | 6546-41 | DKK2 | Dickkopf-related protein 2 | Q9UBU2 | DKK2 |  |  |  |  |
| 1782 | 10746-24 | DKK3 | Dickkopf-related protein 3 | Q9UBP4 | DKK3 |  |  |  |  |
| 1783 | 3607-71 | DKK3 | Dickkopf-related protein 3 | Q9UBP4 | DKK3 |  |  |  |  |
| 1784 | 22959-32 | Dkk-4 | Dickkopf-related protein 4 | Q9UBT3 | DKK4 |  |  |  |  |
| 1785 | 3365-7 | Dkk-4 | Dickkopf-related protein 4 | Q9UBT3 | DKK4 |  |  |  |  |
| 1786 | 14090-23 | DEFI6 | Differentially expressed in FDCP 6 homolog | Q9H4E7 | DEF6 |  | X |  |  |

| # | **Custom Panel (X)** | **SOMAmer SeqID** | **Target Name** | **Human Target or Analyte** | **UniProt ID** | **GeneID** | **Cardiovascular Disease** | **Inflammation and Immune**  **Response** | **Metabolic Disease** | **Oncology** |
| --- | --- | --- | --- | --- | --- | --- | --- | --- | --- | --- |

| 1787 | 9823-2 | DYR | Dihydrofolate reductase | P00374 | DHFR |  | X | X | X |
| --- | --- | --- | --- | --- | --- | --- | --- | --- | --- |
| 1788 | 10025-1 | DLDH | Dihydrolipoyl dehydrogenase, mitochondrial | P09622 | DLD |  |  |  |  |
| 1789 | 15527-90 | DLDH | Dihydrolipoyl dehydrogenase, mitochondrial | P09622 | DLD |  |  | X |  |
| 1790 | 20185-44 | ODP2 | Dihydrolipoyllysine-residue acetyltransferase component of pyruvate dehydrogenase | P10515 | DLAT | X |  | X |  |
| 1791 | 23699-61 | ODO2 | Dihydrolipoyllysine-residue succinyltransferase component of 2-oxoglutarate dehydrogenase | P36957 | DLST |  |  |  |  |
| 1792 | 21346-71 | PYRD | Dihydroorotate dehydrogenase (quinone), mitochondrial | Q02127 | DHODH |  |  |  |  |
| 1793 | 11257-1 | DHPR | Dihydropteridine reductase | P09417 | QDPR |  |  | X |  |
| 1794 | 20165-4 | DPYS | Dihydropyrimidinase | Q14117 | DPYS |  |  | X |  |
| 1795 | 18323-39 | DRP-1 | Dihydropyrimidinase-related protein 1 | Q14194 | CRMP1 |  |  |  |  |
| 1796 | 13567-1 | DPYL2 | Dihydropyrimidinase-related protein 2 | Q16555 | DPYSL2 |  |  |  |  |
| 1797 | 12707-26 | DPYL3 | Dihydropyrimidinase-related protein 3 | Q14195 | DPYSL3 |  |  |  |  |
| 1798 | 20247-17 | DPYL4 | Dihydropyrimidinase-related protein 4 | O14531 | DPYSL4 |  |  |  |  |
| 1799 | 12683-156 | DPYL5 | Dihydropyrimidinase-related protein 5 | Q9BPU6 | DPYSL5 |  |  |  |  |
| 1800 | 23644-19 | DAK | Dihydroxyacetone kinase | Q3LXA3 | TKFC |  |  |  |  |
| 1801 | 21344-31 | TFB1M | Dimethyladenosine transferase 1, mitochondrial | Q8WVM0 | TFB1M |  |  |  |  |
| 1802 | 21193-1 | TFB2M | Dimethyladenosine transferase 2, mitochondrial | Q9H5Q4 | TFB2M |  |  |  |  |
| 1803 | 25288-16 | FMO3 | Dimethylaniline monooxygenase [N-oxide-forming] 3 | P31513 | FMO3 |  |  | X |  |
| 1804 | 10528-2 | DPEP1 | Dipeptidase 1 | P16444 | DPEP1 |  |  |  |  |
| 1805 | 8794-13 | DPEP1 | Dipeptidase 1 | P16444 | DPEP1 |  |  |  |  |
| 1806 | 8327-26 | DPEP2 | Dipeptidase 2 | Q9H4A9 | DPEP2 |  |  |  |  |
| 1807 | 21969-5 | DPP6 | Dipeptidyl aminopeptidase-like protein 6 | P42658 | DPP6 | X |  | X |  |
| 1808 | 13730-18 | CATC | Dipeptidyl peptidase 1 | P53634 | CTSC | X | X |  |  |
| 1809 | 3178-5 | CATC | Dipeptidyl peptidase 1 | P53634 | CTSC |  |  |  |  |
| 1810 | 8346-9 | DPP2 | Dipeptidyl peptidase 2 | Q9UHL4 | DPP7 |  |  |  |  |
| 1811 | 15460-9 | CD26 | Dipeptidyl peptidase 4 | P27487 | DPP4 | X |  | X |  |
| 1812 | 25281-50 | DPP8 | Dipeptidyl peptidase 8 | Q6V1X1 | DPP8 |  |  |  |  |
| 1813 | 12796-44 | NUDT3 | Diphosphoinositol polyphosphate phosphohydrolase 1 | O95989 | NUDT3 |  |  |  |  |
| 1814 | 20957-57 | NUD10 | Diphosphoinositol polyphosphate phosphohydrolase 3-alpha | Q8NFP7 | NUDT10 |  |  |  |  |
| 1815 | 23623-48 | NUD11 | Diphosphoinositol polyphosphate phosphohydrolase 3-beta | Q96G61 | NUDT11 |  |  |  |  |
| 1816 | 18422-41 | ERG19 | Diphosphomevalonate decarboxylase | P53602 | MVD |  |  |  |  |
| 1817 | 19748-3 | ERG19 | Diphosphomevalonate decarboxylase | P53602 | MVD |  |  |  |  |
| 1818 | 23373-77 | DPH5 | Diphthine methyl ester synthase | Q9H2P9 | DPH5 |  |  |  |  |
| 1819 | 25235-2 | DPH7 | Diphthine methyltransferase | Q9BTV6 | DPH7 |  |  |  |  |
| 1820 | 21963-48 | DAB2 | Disabled homolog 2 | P98082 | DAB2 |  |  |  |  |
| 1821 | 15381-45 | Discoidin domain receptor 2 | Discoidin domain-containing receptor 2 | Q16832 | DDR2 |  |  |  |  |
| 1822 | 7884-15 | Discoidin domain receptor 2 | Discoidin domain-containing receptor 2 | Q16832 | DDR2 |  |  |  |  |
| 1823 | 9402-18 | DCBD1 | Discoidin, CUB and LCCL domain-containing protein 1 | Q8N8Z6 | DCBLD1 |  |  |  |  |
| 1824 | 8654-13 | ADAM 10:CD | Disintegrin and metalloproteinase domain-containing protein 10:Cytoplasmic domain | O14672 | ADAM10 |  |  |  | X |
| 1825 | 8405-108 | ADAM 10:ECD | Disintegrin and metalloproteinase domain-containing protein 10:Extracellular domain | O14672 | ADAM10 |  |  |  | X |
| 1826 | 6586-19 | ADA11 | Disintegrin and metalloproteinase domain-containing protein 11 | O75078 | ADAM11 |  |  |  |  |
| 1827 | 4420-7 | ADAM12 | Disintegrin and metalloproteinase domain-containing protein 12 | O43184 | ADAM12 |  |  |  |  |
| 1828 | 9956-7 | ADA15:CD | Disintegrin and metalloproteinase domain-containing protein 15:Cytoplasmic domain | Q13444 | ADAM15 |  |  |  |  |
| 1829 | 15455-40 | ADA15:ECD | Disintegrin and metalloproteinase domain-containing protein 15:Extracellular domain | Q13444 | ADAM15 |  |  |  |  |
| 1830 | 8882-1 | ADAM 17:CD | Disintegrin and metalloproteinase domain-containing protein 17:Cytoplasmic domain | P78536 | ADAM17 |  | X |  |  |
| 1831 | 8959-61 | ADAM 17:ECD | Disintegrin and metalloproteinase domain-containing protein 17:Extracellular domain | P78536 | ADAM17 |  | X |  |  |
| 1832 | 8948-13 | ADA19 | Disintegrin and metalloproteinase domain-containing protein 19 | Q9H013 | ADAM19 |  |  |  |  |
| 1833 | 7933-75 | ADA22 | Disintegrin and metalloproteinase domain-containing protein 22 | Q9P0K1 | ADAM22 |  |  |  |  |
| 1834 | 7049-2 | ADAM 23 | Disintegrin and metalloproteinase domain-containing protein 23 | O75077 | ADAM23 |  |  |  |  |
| 1835 | 13549-15 | ADA29 | Disintegrin and metalloproteinase domain-containing protein 29 | Q9UKF5 | ADAM29 |  |  |  |  |
| 1836 | 8520-8 | ADA30 | Disintegrin and metalloproteinase domain-containing protein 30 | Q9UKF2 | ADAM30 |  |  |  |  |
| 1837 | 7827-20 | ADA32 | Disintegrin and metalloproteinase domain-containing protein 32 | Q8TC27 | ADAM32 |  |  |  |  |
| 1838 | 10753-31 | ADAM7 | Disintegrin and metalloproteinase domain-containing protein 7 | Q9H2U9 | ADAM7 |  |  |  |  |
| 1839 | 3795-6 | ADAM 9 | Disintegrin and metalloproteinase domain-containing protein 9 | Q13443 | ADAM9 |  |  |  |  |
| 1840 | 19620-16 | DLG2 | Disks large homolog 2 | Q15700 | DLG2 |  |  |  |  |
| 1841 | 7897-75 | DLG3 | Disks large homolog 3 | Q92796 | DLG3 |  |  |  |  |
| 1842 | 8070-88 | DLG4 | Disks large homolog 4 | P78352 | DLG4 | X |  |  |  |
| 1843 | 24436-23 | DLGP4 | Disks large-associated protein 4 | Q9Y2H0 | DLGAP4 |  |  |  |  |
| 1844 | 13441-30 | DIXC1 | Dixin | Q155Q3 | DIXDC1 |  |  |  |  |
| 1845 | 19142-39 | DNM3L | DNA (cytosine-5)-methyltransferase 3-like | Q9UJW3 | DNMT3L |  |  |  |  |
| 1846 | 19119-10 | DDIT3 | DNA damage-inducible transcript 3 protein | P35638 | DDIT3 | X | X | X | X |
| 1847 | 17817-22 | DDIT4 | DNA damage-inducible transcript 4 protein | Q9NX09 | DDIT4 |  | X |  |  |
| 1848 | 13930-3 | ABC3G | DNA dC->dU-editing enzyme APOBEC-3G | Q9HC16 | APOBEC3G |  |  |  |  |
| 1849 | 12585-39 | ERCC1 | DNA excision repair protein ERCC-1 | P07992 | ERCC1 |  |  | X | X |
| 1850 | 21249-115 | DFFA | DNA fragmentation factor subunit alpha | O00273 | DFFA |  |  |  |  |
| 1851 | 9739-4 | MSH2 | DNA mismatch repair protein Msh2 | P43246 | MSH2 |  |  | X | X |
| 1852 | 18400-52 | ALKB2 | DNA oxidative demethylase ALKBH2 | Q6NS38 | ALKBH2 |  |  |  |  |
| 1853 | 21210-33 | DPOLB | DNA polymerase beta | P06746 | POLB |  | X |  | X |
| 1854 | 19159-9 | DNA polymerase subunit delta 4 | DNA polymerase delta subunit 4 | Q9HCU8 | POLD4 |  |  |  |  |
| 1855 | 11562-9 | DPOE2 | DNA polymerase epsilon subunit 2 | P56282 | POLE2 |  |  |  |  |
| 1856 | 19170-25 | DPOE3 | DNA polymerase epsilon subunit 3 | Q9NRF9 | POLE3 |  |  |  |  |
| 1857 | 10022-207 | POLH | DNA polymerase eta | Q9Y253 | POLH |  |  | X |  |
| 1858 | 13536-56 | POLI | DNA polymerase iota | Q9UNA4 | POLI |  |  |  |  |
| 1859 | 13591-31 | PRI1 | DNA primase small subunit | P49642 | PRIM1 |  |  |  |  |
| 1860 | 9895-77 | XPF | DNA repair endonuclease XPF | Q92889 | ERCC4 |  |  | X | X |
| 1861 | 2871-73 | RAD51 | DNA repair protein RAD51 homolog 1 | Q06609 | RAD51 |  |  | X | X |

| # | **Custom Panel (X)** | **SOMAmer SeqID** | **Target Name** | **Human Target or Analyte** | **UniProt ID** | **GeneID** | **Cardiovascular Disease** | **Inflammation and Immune**  **Response** | **Metabolic Disease** | **Oncology** |
| --- | --- | --- | --- | --- | --- | --- | --- | --- | --- | --- |

| 1862 | 22544-10 | RA51C | DNA repair protein RAD51 homolog 3 | O43502 | RAD51C |  |  | X | X |
| --- | --- | --- | --- | --- | --- | --- | --- | --- | --- |
| 1863 | 12554-10 | RA51D | DNA repair protein RAD51 homolog 4 | O75771 | RAD51D |  |  |  |  |
| 1864 | 12535-2 | XRCC1 | DNA repair protein XRCC1 | P18887 | XRCC1 |  | X |  | X |
| 1865 | 9886-28 | XRCC4 | DNA repair protein XRCC4 | Q13426 | XRCC4 |  | X | X | X |
| 1866 | 22541-46 | PSF1 | DNA replication complex GINS protein PSF1 | Q14691 | GINS1 |  |  |  |  |
| 1867 | 9897-9 | MCM6 | DNA replication licensing factor MCM6 | Q14566 | MCM6 |  |  | X |  |
| 1868 | 2876-74 | Topoisomerase I | DNA topoisomerase 1 | P11387 | TOP1 |  |  |  | X |
| 1869 | 9947-22 | TOPB1 | DNA topoisomerase 2-binding protein 1 | Q92547 | TOPBP1 |  | X |  |  |
| 1870 | 9849-13 | APEX1 | DNA-(apurinic or apyrimidinic site) lyase | P27695 | APEX1 | X |  |  | X |
| 1871 | 25123-198 | APEX2 | DNA-(apurinic or apyrimidinic site) lyase 2 | Q9UBZ4 | APEX2 |  |  |  |  |
| 1872 | 13476-16 | KIN17 | DNA/RNA-binding protein KIN17 | O60870 | KIN |  |  |  |  |
| 1873 | 12438-127 | 3MG | DNA-3-methyladenine glycosylase | P29372 | MPG |  |  |  |  |
| 1874 | 15402-2 | ID-1 | DNA-binding protein inhibitor ID-1 | P41134 | ID1 |  |  |  |  |
| 1875 | 9436-2 | ID-1 | DNA-binding protein inhibitor ID-1 | P41134 | ID1 | X |  | X |  |
| 1876 | 15403-53 | ID2 | DNA-binding protein inhibitor ID-2 | Q02363 | ID2 |  |  |  |  |
| 1877 | 9426-73 | ID2 | DNA-binding protein inhibitor ID-2 | Q02363 | ID2 |  |  |  |  |
| 1878 | 24958-3 | RFX5 | DNA-binding protein RFX5 | P48382 | RFX5 |  | X | X |  |
| 1879 | 13511-29 | SATB1 | DNA-binding protein SATB1 | Q01826 | SATB1 |  |  |  |  |
| 1880 | 10081-17 | SATB2 | DNA-binding protein SATB2 | Q9UPW6 | SATB2 |  |  |  |  |
| 1881 | 12851-5 | DPOLM | DNA-directed DNA/RNA polymerase mu | Q9NP87 | POLM |  |  |  |  |
| 1882 | 23398-1 | GRL1A | DNA-directed RNA polymerase II subunit GRINL1A | P0CAP2 | POLR2M |  |  |  |  |
| 1883 | 19199-3 | RPB11 | DNA-directed RNA polymerase II subunit RPB11-a | P52435 | POLR2J |  |  |  |  |
| 1884 | 21000-40 | RPB1B | DNA-directed RNA polymerase II subunit RPB11-b1 | Q9GZM3 | POLR2J2 |  |  |  |  |
| 1885 | 20995-47 | RPB3 | DNA-directed RNA polymerase II subunit RPB3 | P19387 | POLR2C |  |  |  |  |
| 1886 | 19809-47 | RPB9 | DNA-directed RNA polymerase II subunit RPB9 | P36954 | POLR2I |  |  |  |  |
| 1887 | 21368-46 | RPC10 | DNA-directed RNA polymerase III subunit RPC10 | Q9Y2Y1 | POLR3K |  |  |  |  |
| 1888 | 11444-49 | RPC6 | DNA-directed RNA polymerase III subunit RPC6 | Q9H1D9 | POLR3F |  |  |  |  |
| 1889 | 17771-35 | RPC9 | DNA-directed RNA polymerase III subunit RPC9 | O75575 | CRCP |  |  |  |  |
| 1890 | 12939-1 | RPAC1 | DNA-directed RNA polymerases I and III subunit RPAC1 | O15160 | POLR1C |  |  |  |  |
| 1891 | 20441-35 | RPAB1 | DNA-directed RNA polymerases I, II, and III subunit RPABC1 | P19388 | POLR2E |  |  |  |  |
| 1892 | 20448-7 | RPAB2 | DNA-directed RNA polymerases I, II, and III subunit RPABC2 | P61218 | POLR2F |  |  |  |  |
| 1893 | 19134-66 | RPAB4 | DNA-directed RNA polymerases I, II, and III subunit RPABC4 | P53803 | POLR2K |  |  |  |  |
| 1894 | 11582-63 | DNJA2 | DnaJ homolog subfamily A member 2 | O60884 | DNAJA2 |  |  |  |  |
| 1895 | 9744-139 | DNJA4 | DnaJ homolog subfamily A member 4 | Q8WW22 | DNAJA4 |  |  |  |  |
| 1896 | 3852-19 | HSP 40 | DnaJ homolog subfamily B member 1 | P25685 | DNAJB1 |  |  |  |  |
| 1897 | 7110-2 | DJB11 | DnaJ homolog subfamily B member 11 | Q9UBS4 | DNAJB11 |  |  |  |  |
| 1898 | 8006-12 | DJB12 | DnaJ homolog subfamily B member 12 | Q9NXW2 | DNAJB12 |  |  |  |  |
| 1899 | 24659-6 | DJB13 | DnaJ homolog subfamily B member 13 | P59910 | DNAJB13 | X |  |  |  |
| 1900 | 8053-16 | DJB14:CD | DnaJ homolog subfamily B member 14:Cytoplasmic domain | Q8TBM8 | DNAJB14 |  |  |  |  |
| 1901 | 8037-53 | DJB14:LD | DnaJ homolog subfamily B member 14:Lumenal domain | Q8TBM8 | DNAJB14 |  |  |  |  |
| 1902 | 11438-6 | DNJB2 | DnaJ homolog subfamily B member 2 | P25686 | DNAJB2 |  |  |  |  |
| 1903 | 23252-4 | DNJB3 | DnaJ homolog subfamily B member 3 | Q8WWF6 | DNAJB3 |  |  |  |  |
| 1904 | 18884-22 | DNJB4 | DnaJ homolog subfamily B member 4 | Q9UDY4 | DNAJB4 |  |  |  |  |
| 1905 | 11606-22 | DNJB6 | DnaJ homolog subfamily B member 6 | O75190 | DNAJB6 |  |  |  |  |
| 1906 | 20942-4 | DNJB8 | DnaJ homolog subfamily B member 8 | Q8NHS0 | DNAJB8 |  |  |  |  |
| 1907 | 11214-40 | DNJB9 | DnaJ homolog subfamily B member 9 | Q9UBS3 | DNAJB9 |  |  |  |  |
| 1908 | 24917-95 | DNJC1 | DnaJ homolog subfamily C member 1 | Q96KC8 | DNAJC1 |  |  |  |  |
| 1909 | 8297-8 | DJC10 | DnaJ homolog subfamily C member 10 | Q8IXB1 | DNAJC10 |  |  |  | X |
| 1910 | 9783-75 | DJC11 | DnaJ homolog subfamily C member 11 | Q9NVH1 | DNAJC11 |  |  |  |  |
| 1911 | 21114-18 | DJC12 | DnaJ homolog subfamily C member 12 | Q9UKB3 | DNAJC12 |  |  |  |  |
| 1912 | 7197-2 | DJC15 | DnaJ homolog subfamily C member 15 | Q9Y5T4 | DNAJC15 |  |  |  |  |
| 1913 | 10454-99 | DJC16 | DnaJ homolog subfamily C member 16 | Q9Y2G8 | DNAJC16 |  |  |  |  |
| 1914 | 14655-1 | DJC17 | DnaJ homolog subfamily C member 17 | Q9NVM6 | DNAJC17 |  |  |  |  |
| 1915 | 8033-1 | DJC18 | DnaJ homolog subfamily C member 18 | Q9H819 | DNAJC18 |  |  |  |  |
| 1916 | 12799-65 | DJC27 | DnaJ homolog subfamily C member 27 | Q9NZQ0 | DNAJC27 |  |  |  |  |
| 1917 | 7866-11 | DJC30 | DnaJ homolog subfamily C member 30 | Q96LL9 | DNAJC30 |  |  |  |  |
| 1918 | 8653-132 | DNJC4:C-term | DnaJ homolog subfamily C member 4:C-term | Q9NNZ3 | DNAJC4 |  |  |  |  |
| 1919 | 8016-19 | DNJC4:N-term | DnaJ homolog subfamily C member 4:N-term | Q9NNZ3 | DNAJC4 |  |  |  |  |
| 1920 | 24669-12 | DNJ5B | DnaJ homolog subfamily C member 5B | Q9UF47 | DNAJC5B |  |  |  |  |
| 1921 | 16831-7 | DOK1 | Docking protein 1 | Q99704 | DOK1 |  |  |  |  |
| 1922 | 14246-50 | DOK2 | Docking protein 2 | O60496 | DOK2 |  |  |  |  |
| 1923 | 19578-19 | DOK2 | Docking protein 2 | O60496 | DOK2 |  |  |  |  |
| 1924 | 17161-1 | OST48 | Dolichyl-diphosphooligosaccharide--protein glycosyltransferase 48 kDa subunit | P39656 | DDOST |  |  | X |  |
| 1925 | 10490-3 | RPN1:CD | Dolichyl-diphosphooligosaccharide--protein glycosyltransferase subunit 1:Cytoplasmic domain | P04843 | RPN1 |  |  |  |  |
| 1926 | 6458-6 | RPN1:LD | Dolichyl-diphosphooligosaccharide--protein glycosyltransferase subunit 1:Lumenal domain | P04843 | RPN1 |  |  |  |  |
| 1927 | 7976-19 | FRS1L | DOMON domain-containing protein FRRS1L | Q9P0K9 | FRRS1L |  |  |  |  |
| 1928 | 8034-6 | DRGX | Dorsal root ganglia homeobox protein | A6NNA5 | DRGX |  |  |  |  |
| 1929 | 7997-118 | DOC2B | Double C2-like domain-containing protein beta | Q14184 | DOC2B |  |  |  |  |
| 1930 | 24688-9 | DMRTB | Doublesex- and mab-3-related transcription factor B1 | Q96MA1 | DMRTB1 |  |  |  |  |
| 1931 | 22369-12 | DMRTD | Doublesex- and mab-3-related transcription factor C2 | Q8IXT2 | DMRTC2 |  |  |  |  |
| 1932 | 11319-106 | MRE11 | Double-strand break repair protein MRE11 | P49959 | MRE11 | X | X | X | X |
| 1933 | 12471-47 | STAU1 | Double-stranded RNA-binding protein Staufen homolog 1 | O95793 | STAU1 |  |  |  |  |
| 1934 | 12970-35 | STAU2 | Double-stranded RNA-binding protein Staufen homolog 2 | Q9NUL3 | STAU2 |  |  |  |  |
| 1935 | 9175-48 | DSCAM | Down syndrome cell adhesion molecule | O60469 | DSCAM |  |  |  |  |
| 1936 | 18886-28 | DSCL1 | Down syndrome cell adhesion molecule-like protein 1 | Q8TD84 | DSCAML1 |  |  |  |  |

| # | **Custom Panel (X)** | **SOMAmer SeqID** | **Target Name** | **Human Target or Analyte** | **UniProt ID** | **GeneID** | **Cardiovascular Disease** | **Inflammation and Immune**  **Response** | **Metabolic Disease** | **Oncology** |
| --- | --- | --- | --- | --- | --- | --- | --- | --- | --- | --- |

| 1937 | 24636-9 | Dr1-associated corepressor | Dr1-associated corepressor | Q14919 | DRAP1 |  |  |  | |
| --- | --- | --- | --- | --- | --- | --- | --- | --- | --- |
| 1938 | 7084-1 | DRAXI | Draxin | Q8NBI3 | DRAXIN |  |  |
| 1939 | 4978-54 | DBNL | Drebrin-like protein | Q9UJU6 | DBNL |  |  |
| 1940 | 9755-19 | DBNL | Drebrin-like protein | Q9UJU6 | DBNL |  |  |
| 1941 | 24319-19 | ISPD | D-ribitol-5-phosphate cytidylyltransferase | A4D126 | CRPPA |  |  |
| 1942 | 19194-9 | Histidyl-tRNA synthetase-related | D-tyrosyl-tRNA(Tyr) deacylase 1 | Q8TEA8 | DTD1 |  |  |
| 1943 | 5252-33 | PDE11 | Dual 3',5'-cyclic-AMP and -GMP phosphodiesterase 11A | Q9HCR9 | PDE11A |  |  |
| 1944 | 11615-16 | DAPP1 | Dual adapter for phosphotyrosine and 3-phosphotyrosine and 3-phosphoinositide | Q9UN19 | DAPP1 |  | X |
| 1945 | 2864-2 | MEK1 | Dual specificity mitogen-activated protein kinase kinase 1 | Q02750 | MAP2K1 | X | X |  | X |
| 1946 | 3628-3 | MP2K2 | Dual specificity mitogen-activated protein kinase kinase 2 | P36507 | MAP2K2 | X |  |  | X |
| 1947 | 6151-18 | MP2K3 | Dual specificity mitogen-activated protein kinase kinase 3 | P46734 | MAP2K3 |  |  |  |  |
| 1948 | 5242-37 | MP2K4 | Dual specificity mitogen-activated protein kinase kinase 4 | P45985 | MAP2K4 |  |  |  | X |
| 1949 | 22041-26 | MP2K5 | Dual specificity mitogen-activated protein kinase kinase 5 | Q13163 | MAP2K5 |  |  |  |  |
| 1950 | 17175-5 | MP2K6 | Dual specificity mitogen-activated protein kinase kinase 6 | P52564 | MAP2K6 |  | X |  |  |
| 1951 | 9940-35 | DUS28 | Dual specificity phosphatase 28 | Q4G0W2 | DUSP28 |  |  |  |  |
| 1952 | 23323-25 | DUPD1 | Dual specificity phosphatase DUPD1 | Q68J44 | DUPD1 |  |  |  |  |
| 1953 | 11327-56 | CLK2 | Dual specificity protein kinase CLK2 | P49760 | CLK2 |  |  |  |  |
| 1954 | 21183-1 | DUS10 | Dual specificity protein phosphatase 10 | Q9Y6W6 | DUSP10 |  |  |  |  |
| 1955 | 6525-17 | DUSP13 | Dual specificity protein phosphatase 13 isoform A | Q6B8I1 | DUSP13 |  |  |  |  |
| 1956 | 12838-28 | DUS15 | Dual specificity protein phosphatase 15 | Q9H1R2 | DUSP15 |  |  |  |  |
| 1957 | 14631-22 | DUS16 | Dual specificity protein phosphatase 16 | Q9BY84 | DUSP16 |  |  |  |  |
| 1958 | 20959-12 | DUS18 | Dual specificity protein phosphatase 18 | Q8NEJ0 | DUSP18 |  |  |  |  |
| 1959 | 20984-142 | DUS19 | Dual specificity protein phosphatase 19 | Q8WTR2 | DUSP19 |  |  |  |  |
| 1960 | 21191-24 | DUS21 | Dual specificity protein phosphatase 21 | Q9H596 | DUSP21 |  |  |  |  |
| 1961 | 17326-44 | DUS23 | Dual specificity protein phosphatase 23 | Q9BVJ7 | DUSP23 |  |  |  |  |
| 1962 | 8967-6 | DUS26 | Dual specificity protein phosphatase 26 | Q9BV47 | DUSP26 |  |  |  |  |
| 1963 | 3480-7 | DUS3 | Dual specificity protein phosphatase 3 | P51452 | DUSP3 |  |  |  |  |
| 1964 | 10035-6 | DUS4 | Dual specificity protein phosphatase 4 | Q13115 | DUSP4 |  | X |  |  |
| 1965 | 12341-8 | DUS6 | Dual specificity protein phosphatase 6 | Q16828 | DUSP6 | X |  |  | X |
| 1966 | 25301-48 | DYR1A | Dual specificity tyrosine-phosphorylation-regulated kinase 1A | Q13627 | DYRK1A |  |  |  |  |
| 1967 | 20205-55 | DYRK2 | Dual specificity tyrosine-phosphorylation-regulated kinase 2 | Q92630 | DYRK2 |  |  |  |  |
| 1968 | 21662-121 | DYRK3 | Dual specificity tyrosine-phosphorylation-regulated kinase 3 | O43781 | DYRK3 |  |  |  |  |
| 1969 | 4359-87 | DYRK3 | Dual specificity tyrosine-phosphorylation-regulated kinase 3 | O43781 | DYRK3 |  |  |  |  |
| 1970 | 5879-51 | Dynactin subunit 2 | Dynactin subunit 2 | Q13561 | DCTN2 |  |  |  |  |
| 1971 | 18311-44 | DCTN6 | Dynactin subunit 6 | O00399 | DCTN6 |  |  |  |  |
| 1972 | 24435-1 | Dynamin | Dynamin-1 | Q05193 | DNM1 |  |  |  |  |
| 1973 | 21452-3 | DNM1L | Dynamin-1-like protein | O00429 | DNM1L | X |  | X |  |
| 1974 | 11572-4 | DYN2 | Dynamin-2 | P50570 | DNM2 |  | X |  |  |
| 1975 | 11396-39 | DNAI1 | Dynein intermediate chain 1, axonemal | Q9UI46 | DNAI1 | X |  |  |  |
| 1976 | 3881-49 | DLC8 | Dynein light chain 1, cytoplasmic | P63167 | DYNLL1 | X |  |  |  |
| 1977 | 11493-169 | DYL2 | Dynein light chain 2, cytoplasmic | Q96FJ2 | DYNLL2 |  |  |  |  |
| 1978 | 3845-51 | DLRB1 | Dynein light chain roadblock-type 1 | Q9NP97 | DYNLRB1 |  |  |  |  |
| 1979 | 17827-53 | DLRB2 | Dynein light chain roadblock-type 2 | Q8TF09 | DYNLRB2 |  |  |  |  |
| 1980 | 14331-262 | DYLT1 | Dynein light chain Tctex-type 1 | P63172 | DYNLT1 |  |  |  |  |
| 1981 | 12867-40 | DYLT3 | Dynein light chain Tctex-type 3 | P51808 | DYNLT3 |  |  |  |  |
| 1982 | 19638-9 | Dynorphin A (1-17) | Dynorphin A (1-17) | P01213 | PDYN | X |  |  |  |
| 1983 | 23352-9 | DTBP1 | Dysbindin | Q96EV8 | DTNBP1 |  |  | X |  |
| 1984 | 20970-14 | DBND1 | Dysbindin domain-containing protein 1 | Q9H9R9 | DBNDD1 |  |  |  |  |
| 1985 | 21122-3 | DBND2 | Dysbindin domain-containing protein 2 | Q9BQY9 | DBNDD2 |  |  |  |  |
| 1986 | 13698-28 | K319L | Dyslexia-associated protein KIAA0319-like protein | Q8IZA0 | KIAA0319L | X | X | X |  |
| 1987 | 24701-21 | DTNA | Dystrobrevin alpha | Q9Y4J8 | DTNA | X |  |  |  |
| 1988 | 25087-11 | DTNA | Dystrobrevin alpha | Q9Y4J8 | DTNA |  |  |  |  |
| 1989 | 8369-102 | DAG1 | Dystroglycan | Q14118 | DAG1 |  |  |  |  |
| 1990 | 22124-94 | DYTN | Dystrotelin | A2CJ06 | DYTN |  |  |  |  |
| 1991 | 22125-9 | E2F5 | E2F5 | Q15329 | E2F5 |  |  |  |  |
| 1992 | 12934-1 | HERC5 | E3 ISG15--protein ligase HERC5 | Q9UII4 | HERC5 |  | X |  |  |
| 1993 | 22517-106 | NSE2 | E3 SUMO-protein ligase NSE2 | Q96MF7 | NSMCE2 |  |  |  |  |
| 1994 | 13513-174 | PIAS3 | E3 SUMO-protein ligase PIAS3 | Q9Y6X2 | PIAS3 |  |  |  |  |
| 1995 | 10342-55 | PIAS4 | E3 SUMO-protein ligase PIAS4 | Q8N2W9 | PIAS4 |  |  |  |  |
| 1996 | 12016-60 | CBL | E3 ubiquitin-protein ligase CBL | P22681 | CBL | X |  |  |  |
| 1997 | 22417-10 | CBLC | E3 ubiquitin-protein ligase CBL-C | Q9ULV8 | CBLC |  |  |  |  |
| 1998 | 9728-4 | CIP1 | E3 ubiquitin-protein ligase CCNB1IP1 | Q9NPC3 | CCNB1IP1 |  |  |  |  |
| 1999 | 11320-29 | CHFR | E3 ubiquitin-protein ligase CHFR | Q96EP1 | CHFR |  |  |  | X |
| 2000 | 10336-3 | CHIP | E3 ubiquitin-protein ligase CHIP | Q9UNE7 | STUB1 |  |  |  |  |
| 2001 | 11350-30 | CHIP | E3 ubiquitin-protein ligase CHIP | Q9UNE7 | STUB1 |  |  |  |  |
| 2002 | 11430-49 | DTX1 | E3 ubiquitin-protein ligase DTX1 | Q86Y01 | DTX1 |  |  |  | X |
| 2003 | 11643-73 | DTX3L | E3 ubiquitin-protein ligase DTX3L | Q8TDB6 | DTX3L |  |  |  |  |
| 2004 | 10063-10 | FANCL | E3 ubiquitin-protein ligase FANCL | Q9NW38 | FANCL |  |  | X |  |
| 2005 | 12669-30 | HECW1 | E3 ubiquitin-protein ligase HECW1 | Q76N89 | HECW1 |  |  |  |  |
| 2006 | 25126-19 | HECW2 | E3 ubiquitin-protein ligase HECW2 | Q9P2P5 | HECW2 |  |  |  |  |
| 2007 | 12551-3 | ITCH | E3 ubiquitin-protein ligase Itchy homolog | Q96J02 | ITCH |  | X |  |  |
| 2008 | 9936-27 | LNX1 | E3 ubiquitin-protein ligase LNX | Q8TBB1 | LNX1 |  |  |  |  |
| 2009 | 24411-144 | LRSM1 | E3 ubiquitin-protein ligase LRSAM1 | Q6UWE0 | LRSAM1 |  |  |  |  |
| 2010 | 13228-75 | MDM2 | E3 ubiquitin-protein ligase Mdm2 | Q00987 | MDM2 |  |  |  |  |
| 2011 | 4245-80 | MDM2 | E3 ubiquitin-protein ligase Mdm2 | Q00987 | MDM2 |  | X | X | X |

| # | **Custom Panel (X)** | **SOMAmer SeqID** | **Target Name** | **Human Target or Analyte** | **UniProt ID** | **GeneID** | **Cardiovascular Disease** | **Inflammation and Immune**  **Response** | **Metabolic Disease** | **Oncology** |
| --- | --- | --- | --- | --- | --- | --- | --- | --- | --- | --- |

| 2012 | 13604-27 | NEUL1 | E3 ubiquitin-protein ligase NEURL1 | O76050 | NEURL1 | X |  | | |
| --- | --- | --- | --- | --- | --- | --- | --- | --- | --- |
| 2013 | 13411-21 | RNF41 | E3 ubiquitin-protein ligase NRDP1 | Q9H4P4 | RNF41 |  |
| 2014 | 13013-41 | PRKN2 | E3 ubiquitin-protein ligase parkin | O60260 | PRKN |  |  |  | X |
| 2015 | 22421-79 | PELI1 | E3 ubiquitin-protein ligase pellino homolog 1 | Q96FA3 | PELI1 |  |  |  |  |
| 2016 | 12702-13 | PELI2 | E3 ubiquitin-protein ligase pellino homolog 2 | Q9HAT8 | PELI2 |  |  |  |  |
| 2017 | 25111-24 | RAD18 | E3 ubiquitin-protein ligase RAD18 | Q9NS91 | RAD18 |  |  |  |  |
| 2018 | 9887-40 | RBBP6 | E3 ubiquitin-protein ligase RBBP6 | Q7Z6E9 | RBBP6 |  |  |  |  |
| 2019 | 14186-13 | RFFL | E3 ubiquitin-protein ligase rififylin | Q8WZ73 | RFFL |  |  |  |  |
| 2020 | 9957-9 | RN114 | E3 ubiquitin-protein ligase RNF114 | Q9Y508 | RNF114 |  |  |  |  |
| 2021 | 6510-56 | RN128:region 1 | E3 ubiquitin-protein ligase RNF128:region 1 | Q8TEB7 | RNF128 |  |  |  |  |
| 2022 | 8633-18 | RN128:region 2 | E3 ubiquitin-protein ligase RNF128:region 2 | Q8TEB7 | RNF128 |  |  |  |  |
| 2023 | 8087-250 | RNF13 | E3 ubiquitin-protein ligase RNF13 | O43567 | RNF13 |  |  |  |  |
| 2024 | 11401-181 | RN146 | E3 ubiquitin-protein ligase RNF146 | Q9NTX7 | RNF146 |  |  |  |  |
| 2025 | 9773-15 | RN149 | E3 ubiquitin-protein ligase RNF149 | Q8NC42 | RNF149 |  |  |  |  |
| 2026 | 23637-55 | RNF25 | E3 ubiquitin-protein ligase RNF25 | Q96BH1 | RNF25 |  |  |  |  |
| 2027 | 23183-6 | RNF31 | E3 ubiquitin-protein ligase RNF31 | Q96EP0 | RNF31 |  |  |  |  |
| 2028 | 13386-248 | RNF34 | E3 ubiquitin-protein ligase RNF34 | Q969K3 | RNF34 |  |  |  |  |
| 2029 | 10505-12 | RNF43 | E3 ubiquitin-protein ligase RNF43 | Q68DV7 | RNF43 |  | X |  | X |
| 2030 | 14120-2 | RNF43 | E3 ubiquitin-protein ligase RNF43 | Q68DV7 | RNF43 |  |  |  |  |
| 2031 | 14663-44 | RNF8 | E3 ubiquitin-protein ligase RNF8 | O76064 | RNF8 |  |  |  |  |
| 2032 | 21858-25 | SIAH1 | E3 ubiquitin-protein ligase SIAH1 | Q8IUQ4 | SIAH1 |  |  |  |  |
| 2033 | 11557-3 | SMUF1 | E3 ubiquitin-protein ligase SMURF1 | Q9HCE7 | SMURF1 |  |  |  |  |
| 2034 | 13985-12 | SMUF2 | E3 ubiquitin-protein ligase SMURF2 | Q9HAU4 | SMURF2 |  |  |  |  |
| 2035 | 22015-2 | RO52 | E3 ubiquitin-protein ligase TRIM21 | P19474 | TRIM21 |  |  |  |  |
| 2036 | 23225-26 | TRI62 | E3 ubiquitin-protein ligase TRIM62 | Q9BVG3 | TRIM62 |  |  |  |  |
| 2037 | 22817-126 | TRIM9 | E3 ubiquitin-protein ligase TRIM9 | Q9C026 | TRIM9 |  |  |  |  |
| 2038 | 13651-54 | ZFP91 | E3 ubiquitin-protein ligase ZFP91 | Q96JP5 | ZFP91 |  |  |  |  |
| 2039 | 10390-21 | ZNRF3 | E3 ubiquitin-protein ligase ZNRF3 | Q9ULT6 | ZNRF3 |  |  |  |  |
| 2040 | 14122-132 | ZNRF3 | E3 ubiquitin-protein ligase ZNRF3 | Q9ULT6 | ZNRF3 |  |  |  |  |
| 2041 | 16618-7 | CD69 | Early activation antigen CD69 | Q07108 | CD69 |  | X | X |  |
| 2042 | 14043-12 | EEA1 | Early endosome antigen 1 | Q15075 | EEA1 |  |  |  |  |
| 2043 | 6410-26 | INSL4 | Early placenta insulin-like peptide | Q14641 | INSL4 |  |  |  |  |
| 2044 | 25907-47 | EMAL2 | Echinoderm microtubule-associated protein-like 2 | O95834 | EML2 |  |  |  |  |
| 2045 | 10970-3 | NAR3 | Ecto-ADP-ribosyltransferase 3 | Q13508 | ART3 |  |  |  |  |
| 2046 | 7970-315 | NAR3 | Ecto-ADP-ribosyltransferase 3 | Q13508 | ART3 |  |  |  |  |
| 2047 | 6576-1 | ART4 | Ecto-ADP-ribosyltransferase 4 | Q93070 | ART4 |  |  |  |  |
| 2048 | 23202-78 | NAR5 | Ecto-ADP-ribosyltransferase 5 | Q96L15 | ART5 |  |  |  |  |
| 2049 | 14024-196 | EDA | Ectodysplasin-A, secreted form | Q92838 | EDA |  |  |  |  |
| 2050 | 2826-53 | EDA | Ectodysplasin-A, secreted form | Q92838 | EDA |  |  |  |  |
| 2051 | 12933-17 | ENOX1 | Ecto-NOX disulfide-thiol exchanger 1 | Q8TC92 | ENOX1 |  |  |  |  |
| 2052 | 13422-66 | ENOX2 | Ecto-NOX disulfide-thiol exchanger 2 | Q16206 | ENOX2 |  |  |  |  |
| 2053 | 3182-38 | CD39 | Ectonucleoside triphosphate diphosphohydrolase 1 | P49961 | ENTPD1 |  |  |  |  |
| 2054 | 7999-23 | CD39 | Ectonucleoside triphosphate diphosphohydrolase 1 | P49961 | ENTPD1 |  |  |  |  |
| 2055 | 18887-7 | ENTP2 | Ectonucleoside triphosphate diphosphohydrolase 2 | Q9Y5L3 | ENTPD2 |  |  |  |  |
| 2056 | 4436-1 | ENTP3 | Ectonucleoside triphosphate diphosphohydrolase 3 | O75355 | ENTPD3 |  |  |  |  |
| 2057 | 4437-56 | ENTP5 | Ectonucleoside triphosphate diphosphohydrolase 5 | O75356 | ENTPD5 |  |  |  |  |
| 2058 | 8932-1 | ENTP6 | Ectonucleoside triphosphate diphosphohydrolase 6 | O75354 | ENTPD6 |  |  | X |  |
| 2059 | 16892-23 | ENPP2 | Ectonucleotide pyrophosphatase/phosphodiesterase family member 2 | Q13822 | ENPP2 |  |  |  |  |
| 2060 | 25251-6 | ENPP3 | Ectonucleotide pyrophosphatase/phosphodiesterase family member 3 | O14638 | ENPP3 |  |  |  |  |
| 2061 | 6556-5 | ENPP5 | Ectonucleotide pyrophosphatase/phosphodiesterase family member 5 | Q9UJA9 | ENPP5 |  |  |  |  |
| 2062 | 15579-26 | ENPP6 | Ectonucleotide pyrophosphatase/phosphodiesterase family member 6 | Q6UWR7 | ENPP6 |  |  |  |  |
| 2063 | 4435-66 | ENPP7 | Ectonucleotide pyrophosphatase/phosphodiesterase family member 7 | Q6UWV6 | ENPP7 |  |  |  |  |
| 2064 | 23259-23 | EFMT1 | EEF1A lysine methyltransferase 1 | Q8WVE0 | EEF1AKMT1 |  |  |  |  |
| 2065 | 23274-27 | EFCB1 | EF-hand calcium-binding domain-containing protein 1 | Q9HAE3 | EFCAB1 |  |  |  |  |
| 2066 | 9514-46 | K0494:C-term | EF-hand calcium-binding domain-containing protein 14:C-term | O75071 | EFCAB14 |  |  |  |  |
| 2067 | 10830-5 | K0494:N-term | EF-hand calcium-binding domain-containing protein 14:N-term | O75071 | EFCAB14 |  |  |  |  |
| 2068 | 24304-3 | EFC4B | EF-hand calcium-binding domain-containing protein 4B | Q9BSW2 | CRACR2A |  |  |  |  |
| 2069 | 19616-100 | EFHD1 | EF-hand domain-containing protein D1 | Q9BUP0 | EFHD1 |  |  |  |  |
| 2070 | 22037-47 | EFHD2 | EF-hand domain-containing protein D2 | Q96C19 | EFHD2 |  |  |  |  |
| 2071 | 8480-29 | FBLN3 | EGF-containing fibulin-like extracellular matrix protein 1 | Q12805 | EFEMP1 | X |  | X | X |
| 2072 | 9360-33 | EDIL3 | EGF-like repeat and discoidin I-like domain-containing protein 3 | O43854 | EDIL3 |  |  |  |  |
| 2073 | 9901-28 | EGLN1 | Egl nine homolog 1 | Q9GZT9 | EGLN1 |  | X |  |  |
| 2074 | 24674-22 | EGLN2 | Egl nine homolog 2 | Q96KS0 | EGLN2 |  |  |  |  |
| 2075 | 20986-58 | EGLN3 | Egl nine homolog 3 | Q9H6Z9 | EGLN3 |  |  |  |  |
| 2076 | 12813-18 | EHBP1 | EH domain-binding protein 1 | Q8NDI1 | EHBP1 |  |  |  |  |
| 2077 | 24235-2 | EHD1 | EH domain-containing protein 1 | Q9H4M9 | EHD1 |  |  |  |  |
| 2078 | 23640-10 | EHD2 | EH domain-containing protein 2 | Q9NZN4 | EHD2 |  |  |  |  |
| 2079 | 25232-4 | EHD3 | EH domain-containing protein 3 | Q9NZN3 | EHD3 |  |  |  |  |
| 2080 | 11421-10 | EHD4 | EH domain-containing protein 4 | Q9H223 | EHD4 |  |  |  |  |
| 2081 | 14284-23 | E2AK4 | eIF-2-alpha kinase GCN2 | Q9P2K8 | EIF2AK4 | X | X | X |  |
| 2082 | 12417-46 | TPRKB | EKC/KEOPS complex subunit TPRKB | Q9Y3C4 | TPRKB |  |  |  |  |
| 2083 | 4982-54 | Elafin | Elafin | P19957 | PI3 | X |  |  |  |
| 2084 | 11592-1 | ELAV1 | ELAV-like protein 1 | Q15717 | ELAVL1 |  |  |  |  |
| 2085 | 21173-25 | ELAV2 | ELAV-like protein 2 | Q12926 | ELAVL2 |  |  |  |  |
| 2086 | 17435-43 | ETFA | Electron transfer flavoprotein subunit alpha, mitochondrial | P13804 | ETFA |  |  | X |  |

| # | **Custom Panel (X)** | **SOMAmer SeqID** | **Target Name** | **Human Target or Analyte** | **UniProt ID** | **GeneID** | **Cardiovascular Disease** | **Inflammation and Immune**  **Response** | **Metabolic Disease** | **Oncology** |
| --- | --- | --- | --- | --- | --- | --- | --- | --- | --- | --- |

| 2087 | 12798-46 | S4A8 | Electroneutral sodium bicarbonate exchanger 1 | Q2Y0W8 | SLC4A8 |  | | | |
| --- | --- | --- | --- | --- | --- | --- | --- | --- | --- |
| 2088 | 24983-119 | RB6I2 | ELKS/RAB6-interacting/CAST family member 1 | Q8IUD2 | ERC1 |
| 2089 | 25256-153 | EF-1-alpha-1 | Elongation factor 1-alpha 1 | P68104 | EEF1A1 |
| 2090 | 25256-23 | EF-1-alpha-1 | Elongation factor 1-alpha 1 | P68104 | EEF1A1 |  |  |  | X |
| 2091 | 25906-5 | EF-1-alpha-1 | Elongation factor 1-alpha 1 | P68104 | EEF1A1 |  |  |  |  |
| 2092 | 5882-34 | EF-1-beta | Elongation factor 1-beta | P24534 | EEF1B2 |  |  |  | X |
| 2093 | 17826-341 | EF1D | Elongation factor 1-delta | P29692 | EEF1D |  |  |  |  |
| 2094 | 24111-10 | EF1G | Elongation factor 1-gamma | P26641 | EEF1G |  |  |  |  |
| 2095 | 25102-23 | ELP1 | Elongator complex protein 1 | O95163 | ELP1 |  |  |  | X |
| 2096 | 12572-236 | EFS | Embryonal Fyn-associated substrate | O43281 | EFS |  |  |  |  |
| 2097 | 9547-29 | EMID1 | EMI domain-containing protein 1 | Q96A84 | EMID1 |  |  |  |  |
| 2098 | 8773-172 | EMIL3:region 1 | EMILIN-3:region 1 | Q9NT22 | EMILIN3 |  |  |  |  |
| 2099 | 9991-112 | EMIL3:region 2 | EMILIN-3:region 2 | Q9NT22 | EMILIN3 |  |  |  |  |
| 2100 | 11656-110 | EVL | Ena/VASP-like protein | Q9UI08 | EVL |  |  |  | X |
| 2101 | 12531-5 | ERVV1 | Endogenous retrovirus group V member 1 Env polyprotein | B6SEH8 | ERVV-1 |  |  |  |  |
| 2102 | 4908-6 | Endoglin | Endoglin | P17813 | ENG | X |  | X |  |
| 2103 | 21802-53 | NEIL1 | Endonuclease 8-like 1 | Q96FI4 | NEIL1 |  |  | X |  |
| 2104 | 21476-43 | NEIL2 | Endonuclease 8-like 2 | Q969S2 | NEIL2 |  |  |  |  |
| 2105 | 12499-108 | SH3G2 | Endophilin-A1 | Q99962 | SH3GL2 |  |  |  |  |
| 2106 | 20139-57 | SH3G2 | Endophilin-A1 | Q99962 | SH3GL2 |  |  |  |  |
| 2107 | 18318-98 | SH3G3 | Endophilin-A3 | Q99963 | SH3GL3 |  |  |  |  |
| 2108 | 23029-3 | SH3G3 | Endophilin-A3 | Q99963 | SH3GL3 |  |  |  |  |
| 2109 | 16859-100 | SHLB1 | Endophilin-B1 | Q9Y371 | SH3GLB1 |  |  |  |  |
| 2110 | 18222-34 | SHLB2 | Endophilin-B2 | Q9NR46 | SH3GLB2 |  |  |  |  |
| 2111 | 4964-67 | ARTS1 | Endoplasmic reticulum aminopeptidase 1 | Q9NZ08 | ERAP1 | X |  |  |  |
| 2112 | 8960-3 | LRAP | Endoplasmic reticulum aminopeptidase 2 | Q6P179 | ERAP2 |  |  |  |  |
| 2113 | 25491-54 | LNP | Endoplasmic reticulum junction formation protein lunapark | Q9C0E8 | LNPK |  |  |  |  |
| 2114 | 8957-72 | XTP3B | Endoplasmic reticulum lectin 1 | Q96DZ1 | ERLEC1 |  |  |  |  |
| 2115 | 7071-23 | MA1B1 | Endoplasmic reticulum mannosyl-oligosaccharide 1,2-alpha-mannosidase | Q9UKM7 | MAN1B1 |  |  |  |  |
| 2116 | 9333-59 | ERP27 | Endoplasmic reticulum resident protein 27 | Q96DN0 | ERP27 |  |  |  |  |
| 2117 | 13728-19 | ERP29 | Endoplasmic reticulum resident protein 29 | P30040 | ERP29 |  |  |  | X |
| 2118 | 4983-6 | ERP29 | Endoplasmic reticulum resident protein 29 | P30040 | ERP29 |  |  |  |  |
| 2119 | 6064-4 | TXNDC4 | Endoplasmic reticulum resident protein 44 | Q9BS26 | ERP44 |  |  |  | X |
| 2120 | 23372-112 | ERGI1 | Endoplasmic reticulum-Golgi intermediate compartment protein 1 | Q969X5 | ERGIC1 |  |  |  |  |
| 2121 | 6393-63 | Endoplasmin | Endoplasmin | P14625 | HSP90B1 |  |  |  |  |
| 2122 | 22381-1 | LACB2 | Endoribonuclease LACTB2 | Q53H82 | LACTB2 |  |  |  |  |
| 2123 | 15491-20 | CD248 | Endosialin | Q9HCU0 | CD248 |  |  |  |  |
| 2124 | 2201-17 | Endostatin | Endostatin | P39060 | COL18A1 | X |  | X |  |
| 2125 | 23148-100 | Endothelial lipase | Endothelial cell-derived lipase | Q9Y5X9 | LIPG |  |  |  |  |
| 2126 | 20536-11 | ESAM | Endothelial cell-selective adhesion molecule | Q96AP7 | ESAM |  |  |  |  |
| 2127 | 2981-9 | ESAM | Endothelial cell-selective adhesion molecule | Q96AP7 | ESAM |  |  |  |  |
| 2128 | 7841-84 | ESAM | Endothelial cell-selective adhesion molecule | Q96AP7 | ESAM |  |  |  |  |
| 2129 | 3805-16 | Endocan | Endothelial cell-specific molecule 1 | Q9NQ30 | ESM1 |  |  |  | X |
| 2130 | 12415-122 | EDF1 | Endothelial differentiation-related factor 1 | O60869 | EDF1 |  |  |  |  |
| 2131 | 2714-78 | EMAP-2 | Endothelial monocyte-activating polypeptide 2 | Q12904 | AIMP1 |  | X |  | X |
| 2132 | 6495-14 | Endothelin 1 | Endothelin-1 | P05305 | EDN1 | X | X | X |  |
| 2133 | 12574-36 | Endothelin 2 | Endothelin-2 | P20800 | EDN2 | X |  |  |  |
| 2134 | 15383-200 | Endothelin 3 | Endothelin-3 | P14138 | EDN3 | X |  |  |  |
| 2135 | 6113-31 | Endothelin 3 | Endothelin-3 | P14138 | EDN3 |  |  |  |  |
| 2136 | 8767-44 | Endothelin-converting enzyme 1:CD | Endothelin-converting enzyme 1:Cytoplasmic domain | P42892 | ECE1 | X |  |  |  |
| 2137 | 3611-70 | Endothelin-converting enzyme 1:ECD | Endothelin-converting enzyme 1:Extracellular domain | P42892 | ECE1 | X |  | X |  |
| 2138 | 7076-17 | ECEL1 | Endothelin-converting enzyme-like 1 | O95672 | ECEL1 |  |  |  |  |
| 2139 | 12764-3 | ELMO1 | Engulfment and cell motility protein 1 | Q92556 | ELMO1 |  | X |  |  |
| 2140 | 25061-8 | ELMO2 | Engulfment and cell motility protein 2 | Q96JJ3 | ELMO2 | X |  |  |  |
| 2141 | 12862-14 | CASL | Enhancer of filamentation 1 | Q14511 | NEDD9 |  |  |  |  |
| 2142 | 13066-42 | EDC4 | Enhancer of mRNA-decapping protein 4 | Q6P2E9 | EDC4 |  |  |  |  |
| 2143 | 11614-29 | ERH | Enhancer of rudimentary homolog | P84090 | ERH |  |  |  |  |
| 2144 | 24914-10 | EZH2 | Enhancer of zeste homolog 2 | Q15910 | EZH2 |  | X |  | X |
| 2145 | 19367-34 | D3D2 | Enoyl-CoA delta isomerase 1, mitochondrial | P42126 | ECI1 |  |  |  |  |
| 2146 | 12859-33 | PECI | Enoyl-CoA delta isomerase 2, mitochondrial | O75521 | ECI2 |  |  |  |  |
| 2147 | 17787-1 | Enoyl-CoA hydratase | Enoyl-CoA hydratase, mitochondrial | P30084 | ECHS1 |  |  | X |  |
| 2148 | 3189-61 | Enterokinase | Enteropeptidase | P98073 | TMPRSS15 |  |  | X |  |
| 2149 | 14019-73 | EVPL | Envoplakin | Q92817 | EVPL |  |  |  |  |
| 2150 | 15576-158 | ECP | Eosinophil cationic protein | P12724 | RNASE3 |  |  |  |  |
| 2151 | 5741-55 | ECP | Eosinophil cationic protein | P12724 | RNASE3 |  | X |  |  |
| 2152 | 5301-7 | Eotaxin | Eotaxin | P51671 | CCL11 | X | X |  |  |
| 2153 | 8079-39 | EID3 | EP300-interacting inhibitor of differentiation 3 | Q8N140 | EID3 |  |  |  |  |
| 2154 | 3431-54 | EphA1 | Ephrin type-A receptor 1 | P21709 | EPHA1 |  |  |  |  |
| 2155 | 5076-53 | EPHAA | Ephrin type-A receptor 10 | Q5JZY3 | EPHA10 |  |  |  |  |
| 2156 | 6036-78 | EPHAA | Ephrin type-A receptor 10 | Q5JZY3 | EPHA10 |  |  |  |  |
| 2157 | 4834-61 | Epithelial cell kinase | Ephrin type-A receptor 2 | P29317 | EPHA2 | X |  |  | X |
| 2158 | 3432-21 | EPHA3 | Ephrin type-A receptor 3 | P29320 | EPHA3 | X |  |  | X |
| 2159 | 16288-17 | EPHA4 | Ephrin type-A receptor 4 | P54764 | EPHA4 |  |  | X |  |
| 2160 | 7190-50 | EPHA4 | Ephrin type-A receptor 4 | P54764 | EPHA4 |  |  |  |  |
| 2161 | 3806-55 | EphA5 | Ephrin type-A receptor 5 | P54756 | EPHA5 |  |  |  |  |

| # | **Custom Panel (X)** | **SOMAmer SeqID** | **Target Name** | **Human Target or Analyte** | **UniProt ID** | **GeneID** | **Cardiovascular Disease** | **Inflammation and Immune**  **Response** | **Metabolic Disease** | **Oncology** |
| --- | --- | --- | --- | --- | --- | --- | --- | --- | --- | --- |

| 2162 | 22587-37 | EPHA6 | Ephrin type-A receptor 6 | Q9UF33 | EPHA6 |  | | | |
| --- | --- | --- | --- | --- | --- | --- | --- | --- | --- |
| 2163 | 15580-2 | EPHA7 | Ephrin type-A receptor 7 | Q15375 | EPHA7 |
| 2164 | 7195-119 | EPHA7 | Ephrin type-A receptor 7 | Q15375 | EPHA7 |
| 2165 | 20558-4 | EPHA8 | Ephrin type-A receptor 8 | P29322 | EPHA8 |
| 2166 | 17680-12 | EPHB1 | Ephrin type-B receptor 1 | P54762 | EPHB1 |
| 2167 | 8225-86 | EPHB2 | Ephrin type-B receptor 2 | P29323 | EPHB2 |  |  |  | X |
| 2168 | 8348-4 | EPHB2 | Ephrin type-B receptor 2 | P29323 | EPHB2 |  |  |  |  |
| 2169 | 9220-7 | EPHB3 | Ephrin type-B receptor 3 | P54753 | EPHB3 |  |  |  |  |
| 2170 | 15530-33 | EphB4 | Ephrin type-B receptor 4 | P54760 | EPHB4 | X |  |  | X |
| 2171 | 5078-82 | EphB6 | Ephrin type-B receptor 6 | O15197 | EPHB6 |  |  |  |  |
| 2172 | 9261-14 | EphB6 | Ephrin type-B receptor 6 | O15197 | EPHB6 |  |  |  |  |
| 2173 | 20091-138 | Ephrin-A1 | Ephrin-A1 | P20827 | EFNA1 |  |  |  | X |
| 2174 | 10801-11 | Ephrin-A2 | Ephrin-A2 | O43921 | EFNA2 |  |  |  |  |
| 2175 | 14124-6 | Ephrin-A2 | Ephrin-A2 | O43921 | EFNA2 |  |  |  |  |
| 2176 | 22580-29 | Ephrin-A2 | Ephrin-A2 | O43921 | EFNA2 |  |  |  |  |
| 2177 | 14153-8 | Ephrin-A3 | Ephrin-A3 | P52797 | EFNA3 |  |  |  |  |
| 2178 | 5759-10 | Ephrin-A3 | Ephrin-A3 | P52797 | EFNA3 |  |  |  |  |
| 2179 | 14050-61 | Ephrin-A4 | Ephrin-A4 | P52798 | EFNA4 |  |  |  |  |
| 2180 | 2614-28 | Ephrin-A4 | Ephrin-A4 | P52798 | EFNA4 |  |  |  |  |
| 2181 | 2615-60 | Ephrin-A5 | Ephrin-A5 | P52803 | EFNA5 | X |  |  |  |
| 2182 | 13104-32 | EFNB1 | Ephrin-B1 | P98172 | EFNB1 |  |  |  |  |
| 2183 | 5746-37 | EFNB1 | Ephrin-B1 | P98172 | EFNB1 |  |  | X |  |
| 2184 | 8772-5 | EFNB2:CD | Ephrin-B2:Cytoplasmic domain | P52799 | EFNB2 | X |  |  | X |
| 2185 | 14131-37 | EFNB2:ECD | Ephrin-B2:Extracellular domain | P52799 | EFNB2 | X |  |  | X |
| 2186 | 7785-1 | Ephrin-B3:CD | Ephrin-B3:Cytoplasmic domain | Q15768 | EFNB3 |  |  |  |  |
| 2187 | 2514-65 | Ephrin-B3:ECD | Ephrin-B3:Extracellular domain | Q15768 | EFNB3 |  |  |  |  |
| 2188 | 2677-1 | ERBB1 | Epidermal growth factor receptor | P00533 | EGFR | X |  |  | X |
| 2189 | 24899-13 | ES8L1 | Epidermal growth factor receptor kinase substrate 8-like protein 1 | Q8TE68 | EPS8L1 |  |  |  |  |
| 2190 | 24891-54 | ES8L2 | Epidermal growth factor receptor kinase substrate 8-like protein 2 | Q9H6S3 | EPS8L2 |  |  |  |  |
| 2191 | 24931-9 | ES8L3 | Epidermal growth factor receptor kinase substrate 8-like protein 3 | Q8TE67 | EPS8L3 |  |  |  |  |
| 2192 | 4212-5 | EP15R | Epidermal growth factor receptor substrate 15-like 1 | Q9UBC2 | EPS15L1 |  |  |  |  |
| 2193 | 19567-1 | EGFRvIII | Epidermal growth factor receptor variant III | P00533 | EGFR |  |  |  |  |
| 2194 | 11949-25 | EGF:CD | Epidermal growth factor:Cytoplasmic domain | P01133 | EGF | X | X |  | X |
| 2195 | 5509-7 | EGF:ECD | Epidermal growth factor:Extracellular domain | P01133 | EGF | X | X | X | X |
| 2196 | 17454-15 | EGFL6 | Epidermal growth factor-like protein 6 | Q8IUX8 | EGFL6 |  |  |  |  |
| 2197 | 9475-22 | GPX5 | Epididymal secretory glutathione peroxidase | O75715 | GPX5 |  |  |  |  |
| 2198 | 9536-16 | EP3A | Epididymal secretory protein E3-alpha | Q14507 | EDDM3A |  |  |  |  |
| 2199 | 7782-34 | EP3B | Epididymal secretory protein E3-beta | P56851 | EDDM3B |  |  |  |  |
| 2200 | 13007-66 | LCN10 | Epididymal-specific lipocalin-10 | Q6JVE6 | LCN10 |  |  |  |  |
| 2201 | 5643-2 | LCN8 | Epididymal-specific lipocalin-8 | Q6JVE9 | LCN8 |  |  |  |  |
| 2202 | 9251-28 | MA2B2 | Epididymis-specific alpha-mannosidase | Q9Y2E5 | MAN2B2 |  |  |  |  |
| 2203 | 4956-2 | EPI | Epiregulin | O14944 | EREG |  |  | X |  |
| 2204 | 11836-144 | discoidin domain receptor 1 | Epithelial discoidin domain-containing receptor 1 | Q08345 | DDR1 |  |  |  | X |
| 2205 | 4122-12 | discoidin domain receptor 1 | Epithelial discoidin domain-containing receptor 1 | Q08345 | DDR1 |  |  |  |  |
| 2206 | 8828-21 | ESRP1 | Epithelial splicing regulatory protein 1 | Q6NXG1 | ESRP1 |  |  |  |  |
| 2207 | 21269-198 | HYES | Epoxide hydrolase 2 | P34913 | EPHX2 | X |  | X |  |
| 2208 | 24255-38 | EPN1 | Epsin-1 | Q9Y6I3 | EPN1 |  |  |  |  |
| 2209 | 11162-37 | EQTN | Equatorin | Q9NQ60 | EQTN |  |  |  |  |
| 2210 | 21512-6 | EDEM2 | ER degradation-enhancing alpha-mannosidase-like 2 | Q9BV94 | EDEM2 |  |  |  |  |
| 2211 | 11989-35 | EMC1 | ER membrane protein complex subunit 1 | Q8N766 | EMC1 |  |  |  |  |
| 2212 | 20435-41 | EMC2 | ER membrane protein complex subunit 2 | Q15006 | EMC2 |  |  |  |  |
| 2213 | 13516-46 | TMM85 | ER membrane protein complex subunit 4 | Q5J8M3 | EMC4 |  |  |  |  |
| 2214 | 17770-42 | EMC8 | ER membrane protein complex subunit 8 | O43402 | EMC8 |  |  |  |  |
| 2215 | 8776-10 | ERLN1 | Erlin-1 | O75477 | ERLIN1 |  |  |  |  |
| 2216 | 23425-19 | ERMIN | Ermin | Q8TAM6 | ERMN |  |  |  |  |
| 2217 | 7060-2 | ERO1A | ERO1-like protein alpha | Q96HE7 | ERO1A |  |  |  |  |
| 2218 | 7994-41 | ERO1B | ERO1-like protein beta | Q86YB8 | ERO1B |  |  |  |  |
| 2219 | 8261-51 | STOM | Erythrocyte band 7 integral membrane protein | P27105 | STOM |  |  |  |  |
| 2220 | 8631-13 | ERMAP | Erythroid membrane-associated protein | Q96PL5 | ERMAP |  |  |  |  |
| 2221 | 5813-58 | Epo | Erythropoietin | P01588 | EPO | X | X | X | X |
| 2222 | 19575-4 | EPO-R | Erythropoietin receptor | P19235 | EPOR |  |  |  | X |
| 2223 | 2715-25 | EPO-R | Erythropoietin receptor | P19235 | EPOR |  |  |  |  |
| 2224 | 5981-6 | ES1 | ES1 protein homolog, mitochondrial | P30042 | None |  |  |  |  |
| 2225 | 3470-1 | sE-Selectin | E-selectin | P16581 | SELE | X | X | X |  |
| 2226 | 24957-6 | ESPN | Espin | B1AK53 | None |  |  |  |  |
| 2227 | 17697-2 | OVCA2 | Esterase OVCA2 | Q8WZ82 | OVCA2 |  |  |  |  |
| 2228 | 4708-3 | 17-beta-HSD 1 | Estradiol 17-beta-dehydrogenase 1 | P14061 | HSD17B1 |  |  |  |  |
| 2229 | 17397-8 | DHB11 | Estradiol 17-beta-dehydrogenase 11 | Q8NBQ5 | HSD17B11 |  |  |  |  |
| 2230 | 19491-11 | DHB8 | Estradiol 17-beta-dehydrogenase 8 | Q92506 | HSD17B8 |  |  |  |  |
| 2231 | 16932-5 | SULT 1E | Estrogen sulfotransferase | P49888 | SULT1E1 |  |  |  |  |
| 2232 | 9878-3 | SULT 1E | Estrogen sulfotransferase | P49888 | SULT1E1 | X |  |  |  |
| 2233 | 8328-9 | EKI1 | Ethanolamine kinase 1 | Q9HBU6 | ETNK1 |  | X |  |  |
| 2234 | 21379-30 | EKI2 | Ethanolamine kinase 2 | Q9NVF9 | ETNK2 |  |  |  |  |
| 2235 | 20952-15 | ECHD1 | Ethylmalonyl-CoA decarboxylase | Q9NTX5 | ECHDC1 |  |  |  |  |
| 2236 | 10006-25 | ELK1 | ETS domain-containing protein Elk-1 | P19419 | ELK1 |  |  |  |  |

| # | **Custom Panel (X)** | **SOMAmer SeqID** | **Target Name** | **Human Target or Analyte** | **UniProt ID** | **GeneID** | **Cardiovascular Disease** | **Inflammation and Immune**  **Response** | **Metabolic Disease** | **Oncology** |
| --- | --- | --- | --- | --- | --- | --- | --- | --- | --- | --- |

| 2237 | 5707-55 | ELK3 | ETS domain-containing protein Elk-3 | P41970 | ELK3 | X |  |  | X |
| --- | --- | --- | --- | --- | --- | --- | --- | --- | --- |
| 2238 | 22128-8 | ELK4 | ETS domain-containing protein Elk-4 | P28324 | ELK4 |  |  |  |  |
| 2239 | 13387-55 | EHF | ETS homologous factor | Q9NZC4 | EHF |  |  |  |  |
| 2240 | 23210-4 | ETV2 | ETS translocation variant 2 | O00321 | ETV2 |  |  |  |  |
| 2241 | 22134-1 | ETV4 | ETS translocation variant 4 | P43268 | ETV4 |  |  |  | X |
| 2242 | 13403-5 | ETV5 | ETS translocation variant 5 | P41161 | ETV5 |  |  |  |  |
| 2243 | 13457-33 | ELF5 | ETS-related transcription factor Elf-5 | Q9UKW6 | ELF5 |  |  |  |  |
| 2244 | 12332-7 | EF2K | Eukaryotic elongation factor 2 kinase | O00418 | EEF2K |  |  |  |  |
| 2245 | 18829-4 | IF4A1 | Eukaryotic initiation factor 4A-I | P60842 | EIF4A1 |  | X |  |  |
| 2246 | 18824-7 | IF4A2 | Eukaryotic initiation factor 4A-II | Q14240 | EIF4A2 |  |  |  |  |
| 2247 | 22984-10 | IF4A2 | Eukaryotic initiation factor 4A-II | Q14240 | EIF4A2 |  |  |  |  |
| 2248 | 4997-19 | IF4A3 | Eukaryotic initiation factor 4A-III | P38919 | EIF4A3 |  |  |  |  |
| 2249 | 24253-5 | ERF3B | Eukaryotic peptide chain release factor GTP-binding subunit ERF3B | Q8IYD1 | GSPT2 |  |  |  |  |
| 2250 | 21628-6 | MCA3 | Eukaryotic translation elongation factor 1 epsilon-1 | O43324 | EEF1E1 |  |  |  |  |
| 2251 | 20913-27 | EIF1 | Eukaryotic translation initiation factor 1 | P41567 | EIF1 |  |  |  |  |
| 2252 | 9850-38 | IF1AX | Eukaryotic translation initiation factor 1A, X-chromosomal | P47813 | EIF1AX |  |  |  |  |
| 2253 | 19224-5 | IF1AY | Eukaryotic translation initiation factor 1A, Y-chromosomal | O14602 | EIF1AY |  |  |  |  |
| 2254 | 12701-1 | EIF1B | Eukaryotic translation initiation factor 1b | O60739 | EIF1B |  |  |  |  |
| 2255 | 19259-176 | IF2A | Eukaryotic translation initiation factor 2 subunit 1 | P05198 | EIF2S1 |  |  |  |  |
| 2256 | 24723-58 | eIF-2-beta | Eukaryotic translation initiation factor 2 subunit 2 | P20042 | EIF2S2 |  |  |  | X |
| 2257 | 25105-70 | EIF2A | Eukaryotic translation initiation factor 2A | Q9BY44 | EIF2A | X |  |  |  |
| 2258 | 25105-87 | EIF2A | Eukaryotic translation initiation factor 2A | Q9BY44 | EIF2A |  |  |  |  |
| 2259 | 21504-41 | I2C2 | Eukaryotic translation initiation factor 2C 2 | Q9UKV8 | AGO2 |  |  |  |  |
| 2260 | 24979-17 | EIF3B | Eukaryotic translation initiation factor 3 subunit B | P55884 | EIF3B |  | X |  |  |
| 2261 | 11454-87 | EIF3G | Eukaryotic translation initiation factor 3 subunit G | O75821 | EIF3G |  |  |  |  |
| 2262 | 13497-34 | EIF3J | Eukaryotic translation initiation factor 3 subunit J | O75822 | EIF3J |  |  |  |  |
| 2263 | 23410-46 | EIF3M | Eukaryotic translation initiation factor 3 subunit M | Q7L2H7 | EIF3M |  |  |  |  |
| 2264 | 25287-7 | IF4G1 | Eukaryotic translation initiation factor 4 gamma 1 | Q04637 | EIF4G1 |  | X |  |  |
| 2265 | 4230-1 | IF4G2 | Eukaryotic translation initiation factor 4 gamma 2 | P78344 | EIF4G2 |  |  |  |  |
| 2266 | 13991-47 | IF4G3 | Eukaryotic translation initiation factor 4 gamma 3 | O43432 | EIF4G3 |  |  |  |  |
| 2267 | 14675-20 | IF4B | Eukaryotic translation initiation factor 4B | P23588 | EIF4B |  |  |  |  |
| 2268 | 19263-147 | IF4E | Eukaryotic translation initiation factor 4E | P06730 | EIF4E |  | X |  |  |
| 2269 | 9745-20 | IF4E2 | Eukaryotic translation initiation factor 4E type 2 | O60573 | EIF4E2 |  |  |  |  |
| 2270 | 17372-5 | 4EBP1 | Eukaryotic translation initiation factor 4E-binding protein 1 | Q13541 | EIF4EBP1 |  | X |  | X |
| 2271 | 20117-5 | 4EBP2 | Eukaryotic translation initiation factor 4E-binding protein 2 | Q13542 | EIF4EBP2 |  |  |  |  |
| 2272 | 4184-43 | 4EBP2 | Eukaryotic translation initiation factor 4E-binding protein 2 | Q13542 | EIF4EBP2 |  |  |  |  |
| 2273 | 18859-7 | 4EBP3 | Eukaryotic translation initiation factor 4E-binding protein 3 | O60516 | EIF4EBP3 |  |  |  |  |
| 2274 | 5885-55 | eIF-4H | Eukaryotic translation initiation factor 4H | Q15056 | EIF4H |  |  |  |  |
| 2275 | 2612-5 | eIF-5 | Eukaryotic translation initiation factor 5 | P55010 | EIF5 |  |  |  |  |
| 2276 | 5888-29 | eIF-5A-1 | Eukaryotic translation initiation factor 5A-1 | P63241 | EIF5A |  | X |  | X |
| 2277 | 11355-10 | IF5A2 | Eukaryotic translation initiation factor 5A-2 | Q9GZV4 | EIF5A2 |  |  |  |  |
| 2278 | 25110-19 | EXOC7 | Exocyst complex component 7 | Q9UPT5 | EXOC7 |  |  |  |  |
| 2279 | 7930-3 | EXOS1 | Exosome complex component CSL4 | Q9Y3B2 | EXOSC1 |  |  |  |  |
| 2280 | 12605-1 | Exosome component 3 | Exosome complex component RRP40 | Q9NQT5 | EXOSC3 |  |  |  |  |
| 2281 | 18909-11 | EXOS8 | Exosome complex component RRP43 | Q96B26 | EXOSC8 |  |  |  |  |
| 2282 | 20968-22 | EXOS5 | Exosome complex component RRP46 | Q9NQT4 | EXOSC5 |  |  |  |  |
| 2283 | 6528-95 | EXTL2 | Exostosin-like 2 | Q9UBQ6 | EXTL2 |  |  |  |  |
| 2284 | 25963-2 | XPO5 | Exportin-5 | Q9HAV4 | XPO5 |  |  |  |  |
| 2285 | 7163-26 | lacritin | Extracellular glycoprotein lacritin | Q9GZZ8 | LACRT |  |  |  |  |
| 2286 | 3366-51 | ECM1 | Extracellular matrix protein 1 | Q16610 | ECM1 |  |  |  |  |
| 2287 | 8305-18 | SULF2 | Extracellular sulfatase Sulf-2 | Q8IWU5 | SULF2 |  |  |  |  |
| 2288 | 5660-51 | SOD3 | Extracellular superoxide dismutase [Cu-Zn] | P08294 | SOD3 | X | X |  |  |
| 2289 | 8463-2 | SOD3 | Extracellular superoxide dismutase [Cu-Zn] | P08294 | SOD3 |  |  |  |  |
| 2290 | 8664-36 | PKDCC | Extracellular tyrosine-protein kinase PKDCC | Q504Y2 | PKDCC |  |  |  |  |
| 2291 | 21152-25 | EYA2 | Eyes absent homolog 2 | O00167 | EYA2 |  |  |  |  |
| 2292 | 9753-17 | Ezrin | Ezrin | P15311 | EZR |  |  |  | X |
| 2293 | 24694-158 | NHERF | Ezrin-radixin-moesin-binding phosphoprotein 50 | O14745 | SLC9A3R1 |  |  | X |  |
| 2294 | 5032-64 | SSRP1 | FACT complex subunit SSRP1 | Q08945 | SSRP1 |  |  |  |  |
| 2295 | 11449-22 | CAZA1 | F-actin-capping protein subunit alpha-1 | P52907 | CAPZA1 |  |  |  |  |
| 2296 | 23557-110 | CAPZB | F-actin-capping protein subunit beta | P47756 | CAPZB | X |  |  | X |
| 2297 | 20067-26 | FSAP | Factor seven-activating protease | Q14520 | HABP2 |  |  |  |  |
| 2298 | 8017-23 | FXRD1 | FAD-dependent oxidoreductase domain-containing protein 1 | Q96CU9 | FOXRED1 |  |  | X |  |
| 2299 | 15398-2 | HERV1 | FAD-linked sulfhydryl oxidase ALR | P55789 | GFER |  |  | X | X |
| 2300 | 11336-9 | FANCF | Fanconi anemia group F protein | Q9NPI8 | FANCF |  |  | X |  |
| 2301 | 20960-47 | FUBP1 | Far upstream element-binding protein 1 | Q96AE4 | FUBP1 |  |  |  |  |
| 2302 | 24926-9 | FUBP2 | Far upstream element-binding protein 2 | Q92945 | KHSRP |  |  |  |  |
| 2303 | 24952-28 | FUBP3 | Far upstream element-binding protein 3 | Q96I24 | FUBP3 |  |  |  |  |
| 2304 | 21501-30 | FPPS | Farnesyl pyrophosphate synthetase | P14324 | FDPS |  |  |  |  |
| 2305 | 10420-30 | FAIM1 | Fas apoptotic inhibitory molecule 1 | Q9NVQ4 | FAIM |  |  |  |  |
| 2306 | 16594-44 | FAIM1 | Fas apoptotic inhibitory molecule 1 | Q9NVQ4 | FAIM |  |  |  |  |
| 2307 | 6574-11 | FAIM3 | Fas apoptotic inhibitory molecule 3 | O60667 | FCMR |  |  |  |  |
| 2308 | 16593-3 | FADD | FAS-associated death domain protein | Q13158 | FADD | X |  |  | X |
| 2309 | 9738-7 | FAF2 | FAS-associated factor 2 | Q96CS3 | FAF2 |  |  |  |  |
| 2310 | 24226-30 | Fascin | Fascin | Q16658 | FSCN1 |  |  |  |  |
| 2311 | 25051-104 | FTO | Fat mass and obesity-associated protein | Q9C0B1 | FTO |  |  | X | X |

| # | **Custom Panel (X)** | **SOMAmer SeqID** | **Target Name** | **Human Target or Analyte** | **UniProt ID** | **GeneID** | **Cardiovascular Disease** | **Inflammation and Immune**  **Response** | **Metabolic Disease** | **Oncology** |
| --- | --- | --- | --- | --- | --- | --- | --- | --- | --- | --- |

| 2312 | 9074-6 | CE004 | Fatty acid hydroxylase domain-containing protein 2 | Q96IV6 | FAXDC2 |  | X |  | |
| --- | --- | --- | --- | --- | --- | --- | --- | --- | --- |
| 2313 | 8403-18 | Fatty acid synthase | Fatty acid synthase | P49327 | FASN | X |  | X | X |
| 2314 | 18888-37 | FBP12 | Fatty acid-binding protein 12 | A6NFH5 | FABP12 |  |  |  |  |
| 2315 | 18194-18 | FABP9 | Fatty acid-binding protein 9 | Q0Z7S8 | FABP9 |  |  |  |  |
| 2316 | 15386-7 | FABPA | Fatty acid-binding protein, adipocyte | P15090 | FABP4 | X |  |  | X |
| 2317 | 9851-9 | FABPA | Fatty acid-binding protein, adipocyte | P15090 | FABP4 |  |  |  |  |
| 2318 | 4985-11 | FABPE | Fatty acid-binding protein, epidermal | Q01469 | FABP5 | X |  |  |  |
| 2319 | 4696-2 | FABP | Fatty acid-binding protein, heart | P05413 | FABP3 |  |  |  |  |
| 2320 | 5437-63 | FABP | Fatty acid-binding protein, heart | P05413 | FABP3 |  |  |  |  |
| 2321 | 15385-116 | FABP2 | Fatty acid-binding protein, intestinal | P12104 | FABP2 | X |  |  |  |
| 2322 | 11516-7 | FABPL | Fatty acid-binding protein, liver | P07148 | FABP1 |  |  | X |  |
| 2323 | 8396-42 | FAAH2 | Fatty-acid amide hydrolase 2 | Q6GMR7 | FAAH2 |  |  |  | X |
| 2324 | 24895-25 | FCSD1 | F-BAR and double SH3 domains protein 1 | Q86WN1 | FCHSD1 |  |  |  |  |
| 2325 | 22143-6 | FBX28 | F-box only protein 28 | Q9NVF7 | FBXO28 |  |  |  |  |
| 2326 | 14628-72 | FBX3 | F-box only protein 3 | Q9UK99 | FBXO3 |  |  |  |  |
| 2327 | 22144-65 | FBX48 | F-box only protein 48 | Q5FWF7 | FBXO48 |  |  |  |  |
| 2328 | 9951-36 | FBXL4:LRR2 and LRR3 | F-box/LRR-repeat protein 4:Leucine-rich repeats 2 and 3 | Q9UKA2 | FBXL4 |  |  |  |  |
| 2329 | 11416-23 | FBXL4:LRR4 and LRR5 | F-box/LRR-repeat protein 4:Leucine-rich repeats 4 and 5 | Q9UKA2 | FBXL4 |  |  |  |  |
| 2330 | 12846-3 | FBXL5 | F-box/LRR-repeat protein 5 | Q9UKA1 | FBXL5 |  |  |  |  |
| 2331 | 24975-3 | FCRLA | Fc receptor-like A | Q7L513 | FCRLA |  |  |  |  |
| 2332 | 15583-18 | FCRLB | Fc receptor-like B | Q6BAA4 | FCRLB |  |  |  |  |
| 2333 | 5728-60 | FCRL1 | Fc receptor-like protein 1 | Q96LA6 | FCRL1 |  |  |  |  |
| 2334 | 7951-146 | FCRL2 | Fc receptor-like protein 2 | Q96LA5 | FCRL2 |  |  |  |  |
| 2335 | 4440-15 | FCRL3 | Fc receptor-like protein 3 | Q96P31 | FCRL3 |  | X |  |  |
| 2336 | 8991-115 | FCRL4:CD | Fc receptor-like protein 4:Cytoplasmic domain | Q96PJ5 | FCRL4 |  |  |  |  |
| 2337 | 8973-23 | FCRL4:ECD | Fc receptor-like protein 4:Extracellular domain | Q96PJ5 | FCRL4 |  |  |  |  |
| 2338 | 6103-70 | FCRL5 | Fc receptor-like protein 5 | Q96RD9 | FCRL5 |  |  |  |  |
| 2339 | 6617-12 | FCRL6 | Fc receptor-like protein 6 | Q6DN72 | FCRL6 |  |  |  |  |
| 2340 | 12599-10 | URP2 | Fermitin family homolog 3 | Q86UX7 | FERMT3 |  | X |  |  |
| 2341 | 5934-1 | Ferritin | Ferritin | P02794|P02792 | FTH1|FTL |  |  | X | X |
| 2342 | 25913-17 | FRIH | Ferritin heavy chain | P02794 | FTH1 |  |  | X | X |
| 2343 | 25225-14 | FHL17 | Ferritin heavy polypeptide-like 17 | Q9BXU8 | FTHL17 |  |  |  |  |
| 2344 | 15324-58 | Ferritin light chain | Ferritin light chain | P02792 | FTL |  |  | X |  |
| 2345 | 8048-9 | FTMT | Ferritin, mitochondrial | Q8N4E7 | FTMT |  |  |  |  |
| 2346 | 7740-33 | FATE1 | Fetal and adult testis-expressed transcript protein | Q969F0 | FATE1 |  |  |  |  |
| 2347 | 3367-8 | FETUB | Fetuin-B | Q9UGM5 | FETUB | X |  | X |  |
| 2348 | 21793-4 | FR1OP | FGFR1 oncogene partner | O95684 | CEP43 |  |  |  |  |
| 2349 | 25115-21 | FHOD1 | FH1/FH2 domain-containing protein 1 | Q9Y613 | FHOD1 |  |  |  |  |
| 2350 | 2796-62 | Fibrinogen | Fibrinogen | P02671|P02675|P02679 | FGA|FGB|FGG | X |  |  | X |
| 2351 | 18890-227 | Fibrinogen B | Fibrinogen beta chain | P02675 | FGB |  |  | X |  |
| 2352 | 9378-6 | FBCD1 | Fibrinogen C domain-containing protein 1 | Q8N539 | FIBCD1 |  |  |  |  |
| 2353 | 4989-7 | Fibrinogen g-chain dimer | Fibrinogen gamma chain | P02679 | FGG |  |  |  |  |
| 2354 | 5581-28 | FGL1 | Fibrinogen-like protein 1 | Q08830 | FGL1 |  |  |  |  |
| 2355 | 3486-58 | b-ECGF | Fibroblast growth factor 1 | P05230 | FGF1 | X |  |  |  |
| 2356 | 2441-2 | FGF-10 | Fibroblast growth factor 10 | O15520 | FGF10 |  |  |  | X |
| 2357 | 4392-54 | FGF-12 | Fibroblast growth factor 12 | P61328 | FGF12 |  |  |  |  |
| 2358 | 13725-3 | FGF-16 | Fibroblast growth factor 16 | O43320 | FGF16 |  |  |  |  |
| 2359 | 4393-3 | FGF-16 | Fibroblast growth factor 16 | O43320 | FGF16 |  |  |  |  |
| 2360 | 3494-71 | FGF-17 | Fibroblast growth factor 17 | O60258 | FGF17 |  |  |  |  |
| 2361 | 2761-49 | FGF-18 | Fibroblast growth factor 18 | O76093 | FGF18 |  |  |  |  |
| 2362 | 13724-27 | FGF-19 | Fibroblast growth factor 19 | O95750 | FGF19 |  | X |  |  |
| 2363 | 2762-30 | FGF-19 | Fibroblast growth factor 19 | O95750 | FGF19 |  |  |  |  |
| 2364 | 3025-50 | bFGF | Fibroblast growth factor 2 | P09038 | FGF2 | X | X | X | X |
| 2365 | 2763-66 | FGF-20 | Fibroblast growth factor 20 | Q9NP95 | FGF20 |  |  |  |  |
| 2366 | 9513-9 | FGF22 | Fibroblast growth factor 22 | Q9HCT0 | FGF22 |  |  |  |  |
| 2367 | 3807-1 | FGF23 | Fibroblast growth factor 23 | Q9GZV9 | FGF23 | X | X | X |  |
| 2368 | 7894-155 | FGF-3 | Fibroblast growth factor 3 | P11487 | FGF3 |  |  |  | X |
| 2369 | 4123-60 | FGF-4 | Fibroblast growth factor 4 | P08620 | FGF4 |  |  |  | X |
| 2370 | 3065-65 | FGF-5 | Fibroblast growth factor 5 | P12034 | FGF5 | X | X |  |  |
| 2371 | 4130-71 | FGF-6 | Fibroblast growth factor 6 | P10767 | FGF6 |  |  | X |  |
| 2372 | 14031-18 | FGF7 | Fibroblast growth factor 7 | P21781 | FGF7 | X | X |  |  |
| 2373 | 4487-1 | FGF7 | Fibroblast growth factor 7 | P21781 | FGF7 |  |  |  |  |
| 2374 | 19570-12 | FGF-8 | Fibroblast growth factor 8 | P55075 | FGF8 | X |  |  |  |
| 2375 | 4394-71 | FGF-8A | Fibroblast growth factor 8 isoform A | P55075 | FGF8 |  |  |  |  |
| 2376 | 14757-144 | FGF-8B | Fibroblast growth factor 8 isoform B | P55075 | FGF8 |  |  |  |  |
| 2377 | 2443-10 | FGF-8B | Fibroblast growth factor 8 isoform B | P55075 | FGF8 |  |  |  |  |
| 2378 | 17166-4 | FGF-8F | Fibroblast growth factor 8 isoform F | P55075 | FGF8 |  |  |  |  |
| 2379 | 19584-33 | FGF9 | Fibroblast growth factor 9 | P31371 | FGF9 |  |  |  | X |
| 2380 | 5532-53 | bFGF-R | Fibroblast growth factor receptor 1 | P11362 | FGFR1 | X |  | X | X |
| 2381 | 3808-76 | FGFR-2 | Fibroblast growth factor receptor 2 | P21802 | FGFR2 |  |  | X | X |
| 2382 | 3809-1 | FGFR-3:CD | Fibroblast growth factor receptor 3:Cytoplasmic domain | P22607 | FGFR3 | X | X |  | X |
| 2383 | 13669-6 | FGFR-3:ECD | Fibroblast growth factor receptor 3:Extracellular domain | P22607 | FGFR3 | X | X |  | X |
| 2384 | 4988-49 | FGFR4 | Fibroblast growth factor receptor 4 | P22455 | FGFR4 |  |  |  | X |
| 2385 | 13025-4 | FRS2 | Fibroblast growth factor receptor substrate 2 | Q8WU20 | FRS2 |  |  |  |  |
| 2386 | 6104-1 | FGRL1 | Fibroblast growth factor receptor-like 1 | Q8N441 | FGFRL1 |  |  |  |  |

| # | **Custom Panel (X)** | **SOMAmer SeqID** | **Target Name** | **Human Target or Analyte** | **UniProt ID** | **GeneID** | **Cardiovascular Disease** | **Inflammation and Immune**  **Response** | **Metabolic Disease** | **Oncology** |
| --- | --- | --- | --- | --- | --- | --- | --- | --- | --- | --- |

| 2387 | 6237-11 | FGRL1 | Fibroblast growth factor receptor-like 1 | Q8N441 | FGFRL1 |  |  |  | |
| --- | --- | --- | --- | --- | --- | --- | --- | --- | --- |
| 2388 | 15494-11 | FGFP1 | Fibroblast growth factor-binding protein 1 | Q14512 | FGFBP1 |  |  |
| 2389 | 9507-55 | FGFP1 | Fibroblast growth factor-binding protein 1 | Q14512 | FGFBP1 |  |  |
| 2390 | 11219-95 | FGFP3 | Fibroblast growth factor-binding protein 3 | Q8TAT2 | FGFBP3 |  |  |
| 2391 | 19635-69 | FGL2 | Fibroleukin | Q14314 | FGL2 |  | X |
| 2392 | 6367-66 | fibromodulin | fibromodulin | Q06828 | FMOD |  |  |
| 2393 | 4131-72 | Fibronectin | Fibronectin | P02751 | FN1 | X | X |  | X |
| 2394 | 3434-34 | FN1.3 | Fibronectin Fragment 3 | P02751 | FN1 |  |  |  |  |
| 2395 | 3435-53 | FN1.4 | Fibronectin Fragment 4 | P02751 | FN1 |  |  | X |  |
| 2396 | 15581-16 | FANK1 | Fibronectin type 3 and ankyrin repeat domains protein 1 | Q8TC84 | FANK1 |  |  |  |  |
| 2397 | 23695-1 | FSD1 | Fibronectin type III and SPRY domain-containing protein 1 | Q9BTV5 | FSD1 |  |  |  |  |
| 2398 | 13451-2 | FNDC4 | Fibronectin type III domain-containing protein 4 | Q9H6D8 | FNDC4 |  |  | X |  |
| 2399 | 23365-7 | FNDC8 | Fibronectin type III domain-containing protein 8 | Q8TC99 | FNDC8 |  |  |  |  |
| 2400 | 20064-24 | FN1.2 | Fibronectin-1 Fragment 2 | P02751 | FN1 |  |  |  |  |
| 2401 | 10819-108 | fibulin 1 | Fibulin-1 | P23142 | FBLN1 |  |  |  |  |
| 2402 | 6470-19 | fibulin 1 | Fibulin-1 | P23142 | FBLN1 |  |  |  |  |
| 2403 | 10738-11 | fibulin 5 | Fibulin-5 | Q9UBX5 | FBLN5 |  |  |  |  |
| 2404 | 15585-304 | fibulin 5 | Fibulin-5 | Q9UBX5 | FBLN5 | X | X |  |  |
| 2405 | 21891-31 | FBLN7 | Fibulin-7 | Q53RD9 | FBLN7 |  |  |  |  |
| 2406 | 15582-25 | FCN1 | Ficolin-1 | O00602 | FCN1 |  |  |  |  |
| 2407 | 22961-7 | FCN1 | Ficolin-1 | O00602 | FCN1 |  |  |  |  |
| 2408 | 3613-62 | FCN1 | Ficolin-1 | O00602 | FCN1 |  |  |  |  |
| 2409 | 13717-15 | FCN2 | Ficolin-2 | Q15485 | FCN2 | X |  | X |  |
| 2410 | 3313-21 | FCN2 | Ficolin-2 | Q15485 | FCN2 |  |  |  |  |
| 2411 | 14077-6 | Ficolin-3 | Ficolin-3 | O75636 | FCN3 |  | X |  |  |
| 2412 | 5462-62 | Ficolin-3 | Ficolin-3 | O75636 | FCN3 |  |  |  |  |
| 2413 | 11245-43 | filamin A:CH1 | Filamin-A:Calponin Homology 1 | P21333 | FLNA | X |  |  | X |
| 2414 | 11171-25 | filamin A:CH2 | Filamin-A:Calponin Homology 2 | P21333 | FLNA | X |  |  | X |
| 2415 | 21357-12 | FBLI1 | Filamin-binding LIM protein 1 | Q8WUP2 | FBLIM1 |  | X |  |  |
| 2416 | 25912-131 | FK506 binding protein 1A | FK506 binding protein 1A | P62942 | FKBP1A |  |  |  |  |
| 2417 | 21577-35 | FKBP5 | FK506-binding protein 5 | Q13451 | FKBP5 |  | X |  |  |
| 2418 | 12577-100 | FLAP endonuclease-1 | Flap endonuclease 1 | P39748 | FEN1 |  |  |  |  |
| 2419 | 17148-7 | BLVRB | Flavin reductase (NADPH) | P30043 | BLVRB |  |  |  |  |
| 2420 | 14093-10 | Flt3 ligand | Fms-related tyrosine kinase 3 ligand | P49771 | FLT3LG |  |  |  |  |
| 2421 | 3053-49 | Flt3 ligand | Fms-related tyrosine kinase 3 ligand | P49771 | FLT3LG |  |  |  |  |
| 2422 | 4986-59 | FAK1 | Focal adhesion kinase 1 | Q05397 | PTK2 | X | X |  | X |
| 2423 | 17455-42 | FOLR1 | Folate receptor alpha | P15328 | FOLR1 | X |  | X | X |
| 2424 | 15587-20 | FOLR2 | Folate receptor beta | P14207 | FOLR2 |  |  |  |  |
| 2425 | 15495-9 | FOLR3 | Folate receptor gamma | P41439 | FOLR3 |  |  |  |  |
| 2426 | 5624-66 | FOLR3 | Folate receptor gamma | P41439 | FOLR3 |  |  |  |  |
| 2427 | 3032-11 | FSH | Follicle stimulating hormone | P01215|P01225 | CGA|FSHB | X |  |  |  |
| 2428 | 17346-61 | FDSCP | Follicular dendritic cell secreted peptide | Q8NFU4 | FDCSP |  |  |  |  |
| 2429 | 4132-27 | FST | Follistatin | P19883 | FST | X |  |  | X |
| 2430 | 13112-179 | FSTL1 | Follistatin-related protein 1 | Q12841 | FSTL1 | X |  |  |  |
| 2431 | 3438-10 | FSTL3 | Follistatin-related protein 3 | O95633 | FSTL3 |  |  |  |  |
| 2432 | 9350-3 | FSTL4 | Follistatin-related protein 4 | Q6MZW2 | FSTL4 |  |  |  |  |
| 2433 | 7099-33 | FSTL5 | Follistatin-related protein 5 | Q8N475 | FSTL5 |  |  |  |  |
| 2434 | 8036-75 | FSHB | Follitropin subunit beta | P01225 | FSHB | X |  |  |  |
| 2435 | 14051-54 | FOXC2 | Forkhead box protein C2 | Q99958 | FOXC2 | X | X | X |  |
| 2436 | 9896-21 | FOXGB | Forkhead box protein G1 | P55316 | FOXG1 |  |  |  |  |
| 2437 | 14204-55 | FOXJ2 | Forkhead box protein J2 | Q9P0K8 | FOXJ2 |  |  |  |  |
| 2438 | 11375-49 | FOXL2 | Forkhead box protein L2 | P58012 | FOXL2 |  |  |  | X |
| 2439 | 10056-5 | FOXM1 | Forkhead box protein M1 | Q08050 | FOXM1 |  | X |  | X |
| 2440 | 22055-31 | FOXO1A | Forkhead box protein O1A | Q12778 | FOXO1 |  | X |  | X |
| 2441 | 11540-37 | FOXO3A | Forkhead box protein O3 | O43524 | FOXO3 |  | X | X | X |
| 2442 | 22147-19 | FOXO4 | Forkhead box protein O4 | P98177 | FOXO4 |  |  |  |  |
| 2443 | 24935-62 | FOXP1 | Forkhead box protein P1 | Q9H334 | FOXP1 |  | X |  | X |
| 2444 | 22148-135 | FOXP3 | Forkhead box protein P3 | Q9BZS1 | FOXP3 |  | X | X | X |
| 2445 | 24259-24 | FOXP4 | Forkhead box protein P4 | Q8IVH2 | FOXP4 |  |  |  |  |
| 2446 | 9213-24 | FTCD | Formimidoyltransferase-cyclodeaminase | O95954 | FTCD |  | X | X |  |
| 2447 | 25265-8 | FNBP1 | Formin-binding protein 1 | Q96RU3 | FNBP1 |  |  |  |  |
| 2448 | 22146-20 | FOSL2 | Fos-related antigen 2 | P15408 | FOSL2 |  | X |  | X |
| 2449 | 16814-13 | SLIM 1 | Four and a half LIM domains protein 1 | Q13642 | FHL1 | X |  |  |  |
| 2450 | 7921-65 | FJX1 | Four-jointed box protein 1 | Q86VR8 | FJX1 |  |  |  | X |
| 2451 | 2827-23 | Fractalkine/CX3CL-1 | Fractalkine | P78423 | CX3CL1 | X | X | X |  |
| 2452 | 7713-102 | FMR1 | Fragile X mental retardation protein 1 | Q06787 | FMR1 |  |  |  |  |
| 2453 | 7713-50 | FMR1 | Fragile X mental retardation protein 1 | Q06787 | FMR1 |  |  |  |  |
| 2454 | 7721-81 | FMR1 | Fragile X mental retardation protein 1 | Q06787 | FMR1 |  |  |  |  |
| 2455 | 13076-4 | FXR1 | Fragile X mental retardation syndrome-related protein 1 | P51114 | FXR1 |  |  |  |  |
| 2456 | 20568-3 | FREM1 | FRAS1-related extracellular matrix protein 1 | Q5H8C1 | FREM1 |  |  |  |  |
| 2457 | 7246-4 | FREM2 | FRAS1-related extracellular matrix protein 2 | Q5SZK8 | FREM2 |  |  |  |  |
| 2458 | 11833-83 | FRDA | Frataxin, mitochondrial | Q16595 | FXN |  |  | X |  |
| 2459 | 15389-1 | FRDA | Frataxin, mitochondrial | Q16595 | FXN |  |  |  |  |
| 2460 | 21971-47 | FLI1 | Friend leukemia integration 1 transcription factor | Q01543 | FLI1 |  |  |  | X |
| 2461 | 22590-68 | FZD1 | Frizzled-1 | Q9UP38 | FZD1 |  |  |  |  |

| # | **Custom Panel (X)** | **SOMAmer SeqID** | **Target Name** | **Human Target or Analyte** | **UniProt ID** | **GeneID** | **Cardiovascular Disease** | **Inflammation and Immune**  **Response** | **Metabolic Disease** | **Oncology** |
| --- | --- | --- | --- | --- | --- | --- | --- | --- | --- | --- |

| 2462 | 11647-6 | Frizzled-10:CD | Frizzled-10:Cytoplasmic domain | Q9ULW2 | FZD10 |  | | | |
| --- | --- | --- | --- | --- | --- | --- | --- | --- | --- |
| 2463 | 20056-7 | Frizzled-10:FZ | Frizzled-10:Frizzled domain | Q9ULW2 | FZD10 |
| 2464 | 22589-3 | FZD2 | Frizzled-2 | Q14332 | FZD2 |
| 2465 | 22585-5 | FZD4 | Frizzled-4 | Q9ULV1 | FZD4 |
| 2466 | 20066-19 | Frizzled-5 | Frizzled-5 | Q13467 | FZD5 |
| 2467 | 20576-71 | FZD7 | Frizzled-7 | O75084 | FZD7 |
| 2468 | 20577-5 | FZD8 | Frizzled-8 | Q9H461 | FZD8 |
| 2469 | 22591-4 | FZD9 | Frizzled-9 | O00144 | FZD9 |
| 2470 | 21147-9 | Fructosamine-3-kinase | Fructosamine-3-kinase | Q9H479 | FN3K |
| 2471 | 7206-20 | F16P1 | Fructose-1,6-bisphosphatase 1 | P09467 | FBP1 |  |  | X | X |
| 2472 | 9867-23 | F16P2 | Fructose-1,6-bisphosphatase isozyme 2 | O00757 | FBP2 |  |  | X |  |
| 2473 | 12476-50 | TIGAR | Fructose-2,6-bisphosphatase TIGAR | Q9NQ88 | TIGAR |  |  |  |  |
| 2474 | 5864-10 | aldolase A | Fructose-bisphosphate aldolase A | P04075 | ALDOA | X |  | X | X |
| 2475 | 18185-118 | ALDOB | Fructose-bisphosphate aldolase B | P05062 | ALDOB |  | X | X |  |
| 2476 | 9876-20 | aldolase C | Fructose-bisphosphate aldolase C | P09972 | ALDOC |  |  |  |  |
| 2477 | 13384-110 | FUMH | Fumarate hydratase, mitochondrial | P07954 | FH |  |  | X | X |
| 2478 | 11424-4 | FAAA | Fumarylacetoacetase | P16930 | FAH |  |  | X |  |
| 2479 | 23389-28 | FAH2A | Fumarylacetoacetate hydrolase domain-containing protein 2A | Q96GK7 | FAHD2A |  |  |  |  |
| 2480 | 22149-2 | FUND1 | FUN14 domain-containing protein 1 | Q8IVP5 | FUNDC1 |  |  |  |  |
| 2481 | 6276-16 | Furin | Furin | P09958 | FURIN |  |  |  |  |
| 2482 | 25412-53 | FXYD6 | FXYD domain-containing ion transport regulator 6 | Q9H0Q3 | FXYD6 |  |  |  |  |
| 2483 | 24318-13 | FGD2 | FYVE, RhoGEF and PH domain-containing protein 2 | Q7Z6J4 | FGD2 |  |  |  |  |
| 2484 | 20068-61 | GG12F | G antigen 12F | P0CL80 | GAGE12F |  |  |  |  |
| 2485 | 25413-80 | GAGE-2 | G antigen 2 | Q13066 | GAGE2B |  |  |  |  |
| 2486 | 18268-5 | GAG2A | G antigen 2A | Q6NT46 | GAGE2A |  |  |  |  |
| 2487 | 18273-14 | GGE2D | G antigen 2D | Q9UEU5 | GAGE2D |  |  |  |  |
| 2488 | 22968-9 | GGE2D | G antigen 2D | Q9UEU5 | GAGE2D |  |  |  |  |
| 2489 | 24201-86 | GAGC1 | G antigen family C member 1 | O60829 | PAGE4 |  |  |  |  |
| 2490 | 21510-24 | GRK5 | G protein-coupled receptor kinase 5 | P34947 | GRK5 | X |  |  |  |
| 2491 | 8931-124 | G0S2 | G0/G1 switch protein 2 | P27469 | G0S2 |  |  |  | X |
| 2492 | 19767-20 | Cyclin E | G1/S-specific cyclin-E1 | P24864 | CCNE1 |  |  |  | X |
| 2493 | 5347-59 | Cyclin B1 | G2/mitotic-specific cyclin-B1 | P14635 | CCNB1 |  |  |  |  |
| 2494 | 18208-3 | CCNB2 | G2/mitotic-specific cyclin-B2 | O95067 | CCNB2 |  |  |  |  |
| 2495 | 22150-2 | GABP1 | GA-binding protein subunit beta-1 | Q06547 | GABPB1 |  |  |  |  |
| 2496 | 11448-34 | GALK1 | Galactokinase | P51570 | GALK1 |  |  | X |  |
| 2497 | 10734-339 | G3ST2 | Galactose-3-O-sulfotransferase 2 | Q9H3Q3 | GAL3ST2 |  |  |  |  |
| 2498 | 9121-28 | FUT2 | Galactoside 2-alpha-L-fucosyltransferase 2 | Q10981 | FUT2 |  | X |  |  |
| 2499 | 4548-4 | Fucosyltransferase 3 | Galactoside 3(4)-L-fucosyltransferase | P21217 | FUT3 |  |  |  |  |
| 2500 | 19147-6 | PP13 | Galactoside-binding soluble lectin 13 | Q9UHV8 | LGALS13 |  |  |  |  |
| 2501 | 8696-15 | G3ST1 | Galactosylceramide sulfotransferase | Q99999 | GAL3ST1 |  |  |  |  |
| 2502 | 21770-18 | B3GA1 | Galactosylgalactosylxylosylprotein 3-beta-glucuronosyltransferase 1 | Q9P2W7 | B3GAT1 |  |  |  |  |
| 2503 | 6897-38 | B3GA3 | Galactosylgalactosylxylosylprotein 3-beta-glucuronosyltransferase 3 | O94766 | B3GAT3 |  |  |  |  |
| 2504 | 15390-3 | Galanin | Galanin | P22466 | GAL | X | X |  |  |
| 2505 | 9398-30 | GALP | Galanin-like peptide | Q9UBC7 | GALP |  |  |  |  |
| 2506 | 8046-9 | Galectin-1 | Galectin-1 | P09382 | LGALS1 |  | X |  | X |
| 2507 | 11094-104 | LPPL | Galectin-10 | Q05315 | CLC |  |  |  |  |
| 2508 | 3033-57 | Galectin-2 | Galectin-2 | P05162 | LGALS2 | X |  |  |  |
| 2509 | 3066-12 | Galectin-3 | Galectin-3 | P17931 | LGALS3 | X |  |  | X |
| 2510 | 5000-52 | LG3BP | Galectin-3-binding protein | Q08380 | LGALS3BP |  |  |  |  |
| 2511 | 2982-82 | Galectin-4 | Galectin-4 | P56470 | LGALS4 |  |  |  |  |
| 2512 | 9196-8 | Galectin-7 | Galectin-7 | P47929 | LGALS7 |  |  |  | X |
| 2513 | 9400-40 | Galectin-7 | Galectin-7 | P47929 | LGALS7 |  |  |  |  |
| 2514 | 4909-68 | Galectin-8 | Galectin-8 | O00214 | LGALS8 |  |  |  |  |
| 2515 | 9197-4 | LEG9 | Galectin-9 | O00182 | LGALS9 | X | X |  |  |
| 2516 | 18236-3 | LEGL | Galectin-related protein | Q3ZCW2 | LGALSL |  |  |  |  |
| 2517 | 20946-41 | GTSF1 | Gametocyte-specific factor 1 | Q8WW33 | GTSF1 |  |  |  |  |
| 2518 | 17735-130 | GBRAP | Gamma-aminobutyric acid receptor-associated protein | O95166 | GABARAP |  |  |  |  |
| 2519 | 22966-20 | GBRAP | Gamma-aminobutyric acid receptor-associated protein | O95166 | GABARAP |  |  |  |  |
| 2520 | 12661-44 | GBRL1 | Gamma-aminobutyric acid receptor-associated protein-like 1 | Q9H0R8 | GABARAPL1 |  |  |  |  |
| 2521 | 12494-99 | GBRL2 | Gamma-aminobutyric acid receptor-associated protein-like 2 | P60520 | GABARAPL2 |  |  |  |  |
| 2522 | 11279-42 | GABR1 | Gamma-aminobutyric acid type B receptor subunit 1 | Q9UBS5 | GABBR1 |  |  |  |  |
| 2523 | 9930-48 | GABR2:CD | Gamma-aminobutyric acid type B receptor subunit 2:Cytoplasmic domain | O75899 | GABBR2 |  |  |  |  |
| 2524 | 13948-50 | GABR2:ECD | Gamma-aminobutyric acid type B receptor subunit 2:Extracellular domain | O75899 | GABBR2 |  |  |  |  |
| 2525 | 24449-11 | CRGA | Gamma-crystallin A | P11844 | CRYGA |  |  |  |  |
| 2526 | 18203-9 | CRGC | Gamma-crystallin C | P07315 | CRYGC |  |  |  |  |
| 2527 | 12366-16 | CRGD | Gamma-crystallin D | P07320 | CRYGD |  |  |  |  |
| 2528 | 10339-48 | NSE | Gamma-enolase | P09104 | ENO2 |  |  |  |  |
| 2529 | 11083-23 | NSE | Gamma-enolase | P09104 | ENO2 |  |  |  | X |
| 2530 | 9370-69 | GGH | Gamma-glutamyl hydrolase | Q92820 | GGH |  | X |  | X |
| 2531 | 21548-20 | GGT5 | Gamma-glutamyltransferase 5 | P36269 | GGT5 |  |  |  |  |
| 2532 | 13940-19 | IP16:HIN 1 | Gamma-interferon-inducible protein 16:Isoform 2, Hematopoietic expression, interferon- | Q16666 | IFI16 |  |  |  |  |
| 2533 | 12893-159 | IP16:HIN 2 | Gamma-interferon-inducible protein 16:Isoform 2, Hematopoietic expression, interferon- | Q16666 | IFI16 |  |  |  |  |
| 2534 | 23624-34 | PEN2 | Gamma-secretase subunit PEN-2 | Q9NZ42 | PSENEN |  | X |  |  |
| 2535 | 17773-26 | SNAG | Gamma-soluble NSF attachment protein | Q99747 | NAPG |  |  |  |  |
| 2536 | 19630-2 | g-Synuclein | Gamma-synuclein | O76070 | SNCG |  |  |  | X |

| # | **Custom Panel (X)** | **SOMAmer SeqID** | **Target Name** | **Human Target or Analyte** | **UniProt ID** | **GeneID** | **Cardiovascular Disease** | **Inflammation and Immune**  **Response** | **Metabolic Disease** | **Oncology** |
| --- | --- | --- | --- | --- | --- | --- | --- | --- | --- | --- |

| 2537 | 15441-6 | SAP3 | Ganglioside GM2 activator | P17900 | GM2A |  |  | X |  |
| --- | --- | --- | --- | --- | --- | --- | --- | --- | --- |
| 2538 | 23402-147 | GD1L1 | Ganglioside-induced differentiation-associated protein 1-like 1 | Q96MZ0 | GDAP1L1 |  |  |  |
| 2539 | 9937-7 | CXA1 | Gap junction alpha-1 protein | P17302 | GJA1 | X |  |  | X |
| 2540 | 12711-19 | CXA8 | Gap junction alpha-8 protein | P48165 | GJA8 |  |  |  |  |
| 2541 | 11678-105 | CXD2 | Gap junction delta-2 protein | Q9UKL4 | GJD2 |  |  |  |  |
| 2542 | 16292-288 | GIP | Gastric inhibitory polypeptide | P09681 | GIP |  |  |  |  |
| 2543 | 5755-29 | GIP | Gastric inhibitory polypeptide | P09681 | GIP |  |  |  |  |
| 2544 | 17672-184 | Gastric intrinsic factor | Gastric intrinsic factor | P27352 | CBLIF | X |  | X |  |
| 2545 | 5897-58 | Gastrin-releasing peptide | Gastrin-releasing peptide | P07492 | GRP |  |  |  | X |
| 2546 | 8400-74 | Gastrin-releasing peptide | Gastrin-releasing peptide | P07492 | GRP |  |  |  |  |
| 2547 | 15374-15 | CA11 protein | Gastrokine-1 | Q9NS71 | GKN1 |  |  |  | X |
| 2548 | 6416-8 | GKN2 | Gastrokine-2 | Q86XP6 | GKN2 |  |  |  |  |
| 2549 | 6450-8 | FABP6 | Gastrotropin | P51161 | FABP6 |  |  |  |  |
| 2550 | 22154-37 | GATD1 | GATA zinc finger domain-containing protein 1 | Q8WUU5 | GATAD1 | X |  |  |  |
| 2551 | 7161-25 | G6PE | GDH/6PGL endoplasmic bifunctional protein | O95479 | H6PD |  |  | X | X |
| 2552 | 3314-74 | GFRa-1 | GDNF family receptor alpha-1 | P56159 | GFRA1 |  |  |  |  |
| 2553 | 2515-14 | GFRa-2 | GDNF family receptor alpha-2 | O00451 | GFRA2 |  |  |  |  |
| 2554 | 2505-49 | GFRa-3 | GDNF family receptor alpha-3 | O60609 | GFRA3 |  |  |  |  |
| 2555 | 6920-1 | GFRAL | GDNF family receptor alpha-like | Q6UXV0 | GFRAL |  |  |  |  |
| 2556 | 5634-39 | OFUT1 | GDP-fucose protein O-fucosyltransferase 1 | Q9H488 | POFUT1 |  |  |  |  |
| 2557 | 12657-2 | FCL | GDP-L-fucose synthase | Q13630 | TSTA3 |  |  |  |  |
| 2558 | 18337-4 | GMDS | GDP-mannose 4,6 dehydratase | O60547 | GMDS |  |  |  |  |
| 2559 | 16607-78 | Gelsolin | Gelsolin | P06396 | GSN |  |  |  |  |
| 2560 | 4775-34 | Gelsolin | Gelsolin | P06396 | GSN | X | X | X |  |
| 2561 | 21614-2 | GEMI6 | Gem-associated protein 6 | Q8WXD5 | GEMIN6 |  |  |  |  |
| 2562 | 22155-44 | GEMI7 | Gem-associated protein 7 | Q9H840 | GEMIN7 |  |  |  |  |
| 2563 | 17703-40 | GEMI | Geminin | O75496 | GMNN |  |  |  |  |
| 2564 | 24322-1 | T2EA | General transcription factor IIE subunit 1 | P29083 | GTF2E1 |  |  |  |  |
| 2565 | 20927-43 | T2FB | General transcription factor IIF subunit 2 | P13984 | GTF2F2 |  |  |  |  |
| 2566 | 13609-11 | GTF2I | General transcription factor II-I | P78347 | GTF2I | X | X |  |  |
| 2567 | 13639-101 | USO1 | General vesicular transport factor p115 | O60763 | USO1 |  |  |  | X |
| 2568 | 24902-84 | GEPH | Gephyrin | Q9NQX3 | GPHN |  |  | X |  |
| 2569 | 17786-5 | GGPPS | Geranylgeranyl pyrophosphate synthase | O95749 | GGPS1 |  |  |  |  |
| 2570 | 9730-22 | GHDC | GH3 domain-containing protein | Q8N2G8 | GHDC |  |  |  |  |
| 2571 | 10054-3 | GAN | Gigaxonin | Q9H2C0 | GAN |  |  |  |  |
| 2572 | 17784-23 | GMFB | Glia maturation factor beta | P60983 | GMFB |  |  |  |  |
| 2573 | 13062-4 | GMFG | Glia maturation factor gamma | O60234 | GMFG |  |  |  |  |
| 2574 | 19154-41 | Protease nexin I | Glia-derived nexin | P07093 | SERPINE2 |  |  |  |  |
| 2575 | 3217-74 | Protease nexin I | Glia-derived nexin | P07093 | SERPINE2 |  |  |  |  |
| 2576 | 5822-22 | GDNF | Glial cell line-derived neurotrophic factor | P39905 | GDNF |  | X |  | X |
| 2577 | 20126-19 | GFAP | Glial fibrillary acidic protein | P14136 | GFAP |  |  |  |  |
| 2578 | 3034-1 | GFAP | Glial fibrillary acidic protein | P14136 | GFAP |  | X | X |  |
| 2579 | 9265-10 | GLIP1 | Glioma pathogenesis-related protein 1 | P48060 | GLIPR1 |  |  |  |  |
| 2580 | 20591-48 | GLDN | Gliomedin | Q6ZMI3 | GLDN |  |  |  |  |
| 2581 | 4891-50 | Glucagon | Glucagon | P01275 | GCG | X |  | X |  |
| 2582 | 13085-18 | GLP1R:CD | Glucagon-like peptide 1 receptor:Cytoplasmic domain | P43220 | GLP1R | X |  |  |  |
| 2583 | 20581-42 | GLP1R:ECD | Glucagon-like peptide 1 receptor:Extracellular domain | P43220 | GLP1R | X |  | X |  |
| 2584 | 12804-5 | GMEB2 | Glucocorticoid modulatory element-binding protein 2 | Q9UKD1 | GMEB2 |  |  |  |  |
| 2585 | 2857-70 | Glucocorticoid receptor | Glucocorticoid receptor | P04150 | NR3C1 | X |  | X |  |
| 2586 | 12960-9 | HXK4 | Glucokinase | P35557 | GCK |  |  | X |  |
| 2587 | 5223-59 | GCKR | Glucokinase regulatory protein | Q14397 | GCKR |  |  | X |  |
| 2588 | 13954-9 | GNA1 | Glucosamine 6-phosphate N-acetyltransferase | Q96EK6 | GNPNAT1 |  |  |  |  |
| 2589 | 8909-77 | GNPI1 | Glucosamine-6-phosphate isomerase 1 | P46926 | GNPDA1 |  |  |  |  |
| 2590 | 19297-4 | G6PD | Glucose-6-phosphate 1-dehydrogenase | P11413 | G6PD |  | X | X |  |
| 2591 | 4272-46 | PHI | Glucose-6-phosphate isomerase | P06744 | GPI |  |  |  | X |
| 2592 | 20373-141 | GID8 | Glucose-induced degradation protein 8 homolog | Q9NWU2 | GID8 |  |  |  |  |
| 2593 | 5687-5 | GLU2B | Glucosidase 2 subunit beta | P14314 | PRKCSH |  |  |  |  |
| 2594 | 8229-1 | GXLT1 | Glucoside xylosyltransferase 1 | Q4G148 | GXYLT1 |  |  |  |  |
| 2595 | 18331-3 | GLCM | Glucosylceramidase | P04062 | GBA |  |  | X |  |
| 2596 | 3218-8 | PSMA | Glutamate carboxypeptidase 2 | Q04609 | FOLH1 |  |  |  |  |
| 2597 | 5478-50 | PSMA | Glutamate carboxypeptidase 2 | Q04609 | FOLH1 |  |  |  |  |
| 2598 | 11280-6 | DCE1 | Glutamate decarboxylase 1 | Q99259 | GAD1 |  |  |  |  |
| 2599 | 25902-27 | DHE3 | Glutamate dehydrogenase 1, mitochondrial | P00367 | GLUD1 |  |  | X |  |
| 2600 | 25068-131 | DHE4 | Glutamate dehydrogenase 2, mitochondrial | P49448 | GLUD2 |  |  |  |  |
| 2601 | 10760-107 | GRIA4 | Glutamate receptor 4 | P48058 | GRIA4 |  |  |  |  |
| 2602 | 13493-5 | GRID1 | Glutamate receptor ionotropic, delta-1 | Q9ULK0 | GRID1 |  |  |  |  |
| 2603 | 12758-47 | GRID2 | Glutamate receptor ionotropic, delta-2 | O43424 | GRID2 |  |  |  |  |
| 2604 | 20544-103 | GRIK2 | Glutamate receptor ionotropic, kainate 2 | Q13002 | GRIK2 |  |  |  | X |
| 2605 | 18214-2 | GSH0 | Glutamate--cysteine ligase regulatory subunit | P48507 | GCLM | X |  |  |  |
| 2606 | 24920-6 | GLSK | Glutaminase kidney isoform, mitochondrial | O94925 | GLS |  |  |  |  |
| 2607 | 19238-12 | GLNA | Glutamine synthetase | P15104 | GLUL |  |  | X | X |
| 2608 | 21126-27 | GFPT1 | Glutamine--fructose-6-phosphate aminotransferase [isomerizing] 1 | Q06210 | GFPT1 |  |  | X |  |
| 2609 | 23669-20 | GFPT2 | Glutamine--fructose-6-phosphate aminotransferase [isomerizing] 2 | O94808 | GFPT2 |  |  |  |  |
| 2610 | 7849-3 | Glutaminyl cyclase | Glutaminyl-peptide cyclotransferase | Q16769 | QPCT | X | X | X |  |
| 2611 | 8866-53 | QPCTL | Glutaminyl-peptide cyclotransferase-like protein | Q9NXS2 | QPCTL |  |  |  |  |

| # | **Custom Panel (X)** | **SOMAmer SeqID** | **Target Name** | **Human Target or Analyte** | **UniProt ID** | **GeneID** | **Cardiovascular Disease** | **Inflammation and Immune**  **Response** | **Metabolic Disease** | **Oncology** |
| --- | --- | --- | --- | --- | --- | --- | --- | --- | --- | --- |

| 2612 | 15558-63 | AMPE | Glutamyl aminopeptidase | Q07075 | ENPEP |  |  | | |
| --- | --- | --- | --- | --- | --- | --- | --- | --- | --- |
| 2613 | 18386-36 | GLRX1 | Glutaredoxin-1 | P35754 | GLRX |  |
| 2614 | 12486-8 | GLRX2 | Glutaredoxin-2, mitochondrial | Q9NS18 | GLRX2 |  |
| 2615 | 16596-25 | GLRX3 | Glutaredoxin-3 | O76003 | GLRX3 | X |
| 2616 | 13378-80 | YD286 | Glutaredoxin-like protein C5orf63 | A6NC05 | C5orf63 |  |  |  |  |
| 2617 | 16597-11 | GLRX5 | Glutaredoxin-related protein 5, mitochondrial | Q86SX6 | GLRX5 |  |  | X |  |
| 2618 | 19258-24 | GCDH | Glutaryl-CoA dehydrogenase, mitochondrial | Q92947 | GCDH |  |  | X |  |
| 2619 | 15591-28 | Glutathione peroxidase | Glutathione peroxidase 1 | P07203 | GPX1 | X |  | X | X |
| 2620 | 18894-1 | GPX2 | Glutathione peroxidase 2 | P18283 | GPX2 |  |  |  | X |
| 2621 | 21796-43 | Glutathione peroxidase 3 | Glutathione peroxidase 3 | P22352 | GPX3 |  |  | X | X |
| 2622 | 8345-27 | GPX7 | Glutathione peroxidase 7 | Q96SL4 | GPX7 |  |  |  |  |
| 2623 | 19273-3 | Glutathione reductase | Glutathione reductase, mitochondrial | P00390 | GSR |  |  | X |  |
| 2624 | 12446-49 | GST A1-1 | Glutathione S-transferase A1 | P08263 | GSTA1 | X |  |  |  |
| 2625 | 17138-8 | GST A1-1 | Glutathione S-transferase A1 | P08263 | GSTA1 |  |  |  |  |
| 2626 | 23326-10 | GSTA2 | Glutathione S-transferase A2 | P09210 | GSTA2 |  |  |  |  |
| 2627 | 4993-16 | GSTA3 | Glutathione S-transferase A3 | Q16772 | GSTA3 |  |  |  |  |
| 2628 | 14645-253 | GSTA4 | Glutathione S-transferase A4 | O15217 | GSTA4 | X |  |  |  |
| 2629 | 13474-40 | GSTK1 | Glutathione S-transferase kappa 1 | Q9Y2Q3 | GSTK1 |  |  |  |  |
| 2630 | 15395-15 | GST M1-1 | Glutathione S-transferase Mu 1 | P09488 | GSTM1 | X | X | X | X |
| 2631 | 7239-9 | GST M1-1 | Glutathione S-transferase Mu 1 | P09488 | GSTM1 |  |  |  |  |
| 2632 | 9748-31 | GSTM3-3 | Glutathione S-transferase Mu 3 | P21266 | GSTM3 |  |  |  |  |
| 2633 | 18895-54 | GSTM4 | Glutathione S-transferase Mu 4 | Q03013 | GSTM4 |  |  |  |  |
| 2634 | 18212-43 | GSTM5-5 | Glutathione S-transferase Mu 5 | P46439 | GSTM5 |  |  |  |  |
| 2635 | 12436-84 | GST omega-1 | Glutathione S-transferase omega-1 | P78417 | GSTO1 |  |  |  | X |
| 2636 | 13576-15 | Glutathione S-transferase Pi | Glutathione S-transferase P | P09211 | GSTP1 | X | X | X | X |
| 2637 | 4911-49 | Glutathione S-transferase Pi | Glutathione S-transferase P | P09211 | GSTP1 |  |  |  |  |
| 2638 | 19230-12 | GSTT1 | Glutathione S-transferase theta-1 | P30711 | GSTT1 | X | X |  | X |
| 2639 | 24223-5 | GST2 | Glutathione S-transferase theta-2 | P0CG29 | GSTT2 | X |  |  |  |
| 2640 | 11273-176 | GSTT2 | Glutathione S-transferase theta-2B | P0CG30 | GSTT2B |  |  |  |  |
| 2641 | 15526-33 | GSHB | Glutathione synthetase | P48637 | GSS |  |  | X | X |
| 2642 | 23293-15 | CHAC1 | Glutathione-specific gamma-glutamylcyclotransferase 1 | Q9BUX1 | CHAC1 |  |  |  |  |
| 2643 | 3848-14 | GAPDH, liver | Glyceraldehyde-3-phosphate dehydrogenase | P04406 | GAPDH | X |  | X | X |
| 2644 | 8004-15 | G3PT | Glyceraldehyde-3-phosphate dehydrogenase, testis-specific | O14556 | GAPDHS |  |  |  |  |
| 2645 | 11081-1 | GPDA | Glycerol-3-phosphate dehydrogenase [NAD(+)], cytoplasmic | P21695 | GPD1 |  |  | X |  |
| 2646 | 13697-51 | GPDA | Glycerol-3-phosphate dehydrogenase [NAD(+)], cytoplasmic | P21695 | GPD1 |  |  |  |  |
| 2647 | 12420-10 | GPD1L | Glycerol-3-phosphate dehydrogenase 1-like protein | Q8N335 | GPD1L | X |  |  |  |
| 2648 | 18235-16 | PGP | Glycerol-3-phosphate phosphatase | A6NDG6 | PGP |  |  |  |  |
| 2649 | 12786-61 | GDE5 | Glycerophosphocholine phosphodiesterase GPCPD1 | Q9NPB8 | GPCPD1 |  |  |  |  |
| 2650 | 18188-12 | GATM | Glycine amidinotransferase, mitochondrial | P50440 | GATM | X |  | X | X |
| 2651 | 10956-82 | GCSH | Glycine cleavage system H protein, mitochondrial | P23434 | GCSH |  |  | X |  |
| 2652 | 19506-6 | GLYAT | Glycine N-acyltransferase | Q6IB77 | GLYAT |  |  |  |  |
| 2653 | 20069-23 | GLYL2 | Glycine N-acyltransferase-like protein 2 | Q8WU03 | GLYATL2 |  |  |  |  |
| 2654 | 14006-36 | GNMT | Glycine N-methyltransferase | Q14749 | GNMT |  |  | X | X |
| 2655 | 8867-18 | Glycodelin | Glycodelin | P09466 | PAEP |  |  |  | X |
| 2656 | 24414-3 | Glycogen phosphorylase | Glycogen phosphorylase, brain form | P11216 | PYGB | X |  |  |  |
| 2657 | 11441-11 | PYGL | Glycogen phosphorylase, liver form | P06737 | PYGL |  |  | X |  |
| 2658 | 3441-64 | GSK-3 alpha | Glycogen synthase kinase-3 alpha | P49840 | GSK3A |  |  |  |  |
| 2659 | 24050-26 | GSK-3 beta | Glycogen synthase kinase-3 beta | P49841 | GSK3B | X | X | X | X |
| 2660 | 3236-12 | GSK-3 beta | Glycogen synthase kinase-3 beta | P49841 | GSK3B |  |  |  |  |
| 2661 | 25066-32 | GLYG2 | Glycogenin-2 | O15488 | GYG2 |  |  | X |  |
| 2662 | 12513-8 | GLTP | Glycolipid transfer protein | Q9NZD2 | GLTP |  |  |  |  |
| 2663 | 7948-129 | GLTD2 | Glycolipid transfer protein domain-containing protein 2 | A6NH11 | GLTPD2 |  |  |  |  |
| 2664 | 8014-359 | MANEA | Glycoprotein endo-alpha-1,2-mannosidase | Q5SRI9 | MANEA |  |  |  |  |
| 2665 | 6395-58 | GPHA2 | Glycoprotein hormone alpha-2 | Q96T91 | GPHA2 |  |  |  |  |
| 2666 | 14056-4 | Glycoprotein hormones a-chain | Glycoprotein hormones alpha chain | P01215 | CGA |  |  |  |  |
| 2667 | 8044-90 | XG | Glycoprotein Xg | P55808 | XG |  |  |  |  |
| 2668 | 7198-197 | FA20B | Glycosaminoglycan xylosylkinase | O75063 | FAM20B |  |  |  |  |
| 2669 | 8955-60 | GL8D1 | Glycosyltransferase 8 domain-containing protein 1 | Q68CQ7 | GLT8D1 |  |  | X |  |
| 2670 | 21509-29 | GL8D2 | Glycosyltransferase 8 domain-containing protein 2 | Q9H1C3 | GLT8D2 |  |  |  |  |
| 2671 | 7935-26 | LARGE | Glycosyltransferase-like protein LARGE1 | O95461 | LARGE1 |  |  |  |  |
| 2672 | 21351-8 | Glycyl t-RNA synthetase | Glycyl t-RNA synthetase | P41250 | GARS1 |  |  |  |  |
| 2673 | 12448-246 | NMT1 | Glycylpeptide N-tetradecanoyltransferase 1 | P30419 | NMT1 |  |  |  |  |
| 2674 | 5196-7 | NMT1 | Glycylpeptide N-tetradecanoyltransferase 1 | P30419 | NMT1 |  |  |  |  |
| 2675 | 17821-20 | NMT2 | Glycylpeptide N-tetradecanoyltransferase 2 | O60551 | NMT2 |  |  |  |  |
| 2676 | 18295-102 | GRHPR | Glyoxylate reductase/hydroxypyruvate reductase | Q9UBQ7 | GRHPR |  |  | X |  |
| 2677 | 8697-38 | Glypican 1 | Glypican-1 | P35052 | GPC1 |  |  |  | X |
| 2678 | 3315-15 | GPC2 | Glypican-2 | Q8N158 | GPC2 |  |  |  |  |
| 2679 | 4842-62 | Glypican 3 | Glypican-3 | P51654 | GPC3 | X |  |  |  |
| 2680 | 18892-48 | GPC4 | Glypican-4 | O75487 | GPC4 | X |  |  |  |
| 2681 | 4991-12 | GPC5 | Glypican-5 | P78333 | GPC5 |  |  |  |  |
| 2682 | 5350-14 | GPC6 | Glypican-6 | Q9Y625 | GPC6 |  |  | X |  |
| 2683 | 19254-125 | GMPR1 | GMP reductase 1 | P36959 | GMPR |  | X |  |  |
| 2684 | 19446-1 | GMPR2 | GMP reductase 2 | Q9P2T1 | GMPR2 |  |  |  | X |
| 2685 | 18184-28 | GUAA | GMP synthase [glutamine-hydrolyzing] | P49915 | GMPS |  |  |  |  |
| 2686 | 17456-53 | GOLM1 | Golgi membrane protein 1 | Q8NBJ4 | GOLM1 |  |  |  |  |

| # | **Custom Panel (X)** | **SOMAmer SeqID** | **Target Name** | **Human Target or Analyte** | **UniProt ID** | **GeneID** | **Cardiovascular Disease** | **Inflammation and Immune**  **Response** | **Metabolic Disease** | **Oncology** |
| --- | --- | --- | --- | --- | --- | --- | --- | --- | --- | --- |

| 2687 | 8983-7 | GOLM1 | Golgi membrane protein 1 | Q8NBJ4 | GOLM1 |  |  | | |
| --- | --- | --- | --- | --- | --- | --- | --- | --- | --- |
| 2688 | 24721-17 | GOLP3 | Golgi phosphoprotein 3 | Q9H4A6 | GOLPH3 |  |
| 2689 | 23542-8 | GLP3L | Golgi phosphoprotein 3-like | Q9H4A5 | GOLPH3L |  |
| 2690 | 7805-52 | GOS-28 | Golgi SNAP receptor complex member 1 | O95249 | GOSR1 |  |
| 2691 | 10426-21 | GOSR2 | Golgi SNAP receptor complex member 2 | O14653 | GOSR2 | X |
| 2692 | 15522-2 | GAPR1 | Golgi-associated plant pathogenesis-related protein 1 | Q9H4G4 | GLIPR2 |  |
| 2693 | 19174-141 | GOGA7 | Golgin subfamily A member 7 | Q7Z5G4 | GOLGA7 |  |
| 2694 | 21441-20 | gp75 | gp75 | P17643 | TYRP1 |  |  | X | X |
| 2695 | 16599-38 | GPN1 | GPN-loop GTPase 1 | Q9HCN4 | GPN1 |  |  |  |  |
| 2696 | 13540-1 | GPR26 | G-protein coupled receptor 26 | Q8NDV2 | GPR26 |  |  |  |  |
| 2697 | 10767-52 | GPR64 | G-protein coupled receptor 64 | Q8IZP9 | ADGRG2 |  |  |  |  |
| 2698 | 8336-267 | GRM1C | GRAM domain-containing protein 1C | Q8IYS0 | GRAMD1C |  |  |  |  |
| 2699 | 8842-16 | GRM1C | GRAM domain-containing protein 1C | Q8IYS0 | GRAMD1C |  |  |  |  |
| 2700 | 25086-83 | GRM2B | GRAM domain-containing protein 2B | Q96HH9 | GRAMD2B |  |  |  |  |
| 2701 | 12594-5 | GRAN | Grancalcin | P28676 | GCA |  |  |  |  |
| 2702 | 4992-49 | GRN | Granulins | P28799 | GRN | X |  | X |  |
| 2703 | 4840-73 | G-CSF | Granulocyte colony-stimulating factor | P09919 | CSF3 |  |  |  |  |
| 2704 | 8952-65 | G-CSF | Granulocyte colony-stimulating factor | P09919 | CSF3 | X | X |  | X |
| 2705 | 2719-3 | G-CSF-R | Granulocyte colony-stimulating factor receptor | Q99062 | CSF3R |  | X |  | X |
| 2706 | 4697-59 | GM-CSF | Granulocyte-macrophage colony-stimulating factor | P04141 | CSF2 | X | X |  | X |
| 2707 | 10438-19 | CSF2R | Granulocyte-macrophage colony-stimulating factor receptor subunit alpha | P15509 | CSF2RA |  | X |  |  |
| 2708 | 14102-6 | Granulysin | Granulysin | P22749 | GNLY |  |  |  |  |
| 2709 | 3195-50 | Granulysin | Granulysin | P22749 | GNLY |  |  |  |  |
| 2710 | 13712-104 | granzyme A | Granzyme A | P12544 | GZMA |  | X |  |  |
| 2711 | 3440-7 | granzyme A | Granzyme A | P12544 | GZMA |  |  |  |  |
| 2712 | 14041-13 | Granzyme B | Granzyme B | P10144 | GZMB |  |  |  |  |
| 2713 | 4133-54 | Granzyme B | Granzyme B | P10144 | GZMB |  | X |  | X |
| 2714 | 3373-5 | Granzyme H | Granzyme H | P20718 | GZMH |  |  |  |  |
| 2715 | 9545-156 | Granzyme K | Granzyme K | P49863 | GZMK |  |  |  |  |
| 2716 | 5704-74 | Granzyme M | Granzyme M | P51124 | GZMM |  |  |  |  |
| 2717 | 24268-21 | GAB1 | GRB2-associated-binding protein 1 | Q13480 | GAB1 |  |  |  |  |
| 2718 | 12820-1 | GRAP | GRB2-related adapter protein | Q13588 | GRAP |  |  |  |  |
| 2719 | 16074-12 | GRB2-related adapter protein 2 | GRB2-related adapter protein 2 | O75791 | GRAP2 |  |  |  |  |
| 2720 | 5265-12 | GRB2-related adapter protein 2 | GRB2-related adapter protein 2 | O75791 | GRAP2 |  |  |  |  |
| 2721 | 18878-15 | GREM1 | Gremlin-1 | O60565 | GREM1 |  |  |  | X |
| 2722 | 5598-3 | GREM2 | Gremlin-2 | Q9H772 | GREM2 |  |  |  |  |
| 2723 | 24432-3 | GRAP1 | GRIP1-associated protein 1 | Q4V328 | GRIPAP1 |  |  |  |  |
| 2724 | 22972-26 | Gro-b | Gro-beta | P19875 | CXCL2 |  |  |  |  |
| 2725 | 3148-49 | Gro-b | Gro-beta | P19875 | CXCL2 | X | X |  | X |
| 2726 | 22973-8 | Gro-g | Gro-gamma | P19876 | CXCL3 |  |  |  |  |
| 2727 | 2986-49 | Gro-g | Gro-gamma | P19876 | CXCL3 | X |  |  | X |
| 2728 | 2949-6 | GX | Group 10 secretory phospholipase A2 | O15496 | PLA2G10 |  |  |  |  |
| 2729 | 16298-84 | sPLA(2)-IID | Group IID secretory phospholipase A2 | Q9UNK4 | PLA2G2D |  |  |  |  |
| 2730 | 13670-81 | GIIE | Group IIE secretory phospholipase A2 | Q9NZK7 | PLA2G2E |  |  |  |  |
| 2731 | 2447-7 | GIIE | Group IIE secretory phospholipase A2 | Q9NZK7 | PLA2G2E |  |  |  |  |
| 2732 | 9380-2 | sPLA(2)-XIII | Group XIIB secretory phospholipase A2-like protein | Q9BX93 | PLA2G12B |  |  |  |  |
| 2733 | 9302-90 | G45IP | Growth arrest and DNA damage-inducible proteins-interacting protein 1 | Q8TAE8 | GADD45GIP1 |  |  |  |  |
| 2734 | 5463-22 | GAS1 | Growth arrest-specific protein 1 | P54826 | GAS1 |  |  |  |  |
| 2735 | 23394-125 | GAS2 | Growth arrest-specific protein 2 | O43903 | GAS2 |  |  |  |  |
| 2736 | 15391-114 | GAS-6 | Growth arrest-specific protein 6 | Q14393 | GAS6 | X |  | X |  |
| 2737 | 17721-82 | GAS7 | Growth arrest-specific protein 7 | O60861 | GAS7 |  |  | X |  |
| 2738 | 11358-15 | GRB10 | Growth factor receptor-bound protein 10 | Q13322 | GRB10 |  |  |  |  |
| 2739 | 13628-58 | GRB14 | Growth factor receptor-bound protein 14 | Q14449 | GRB14 |  |  | X |  |
| 2740 | 5464-52 | GRB2 adapter protein | Growth factor receptor-bound protein 2 | P62993 | GRB2 |  |  |  |  |
| 2741 | 11281-6 | GRB7 | Growth factor receptor-bound protein 7 | Q14451 | GRB7 |  |  |  | X |
| 2742 | 2948-58 | Growth hormone receptor | Growth hormone receptor | P10912 | GHR | X |  | X |  |
| 2743 | 10978-39 | SOM2 | Growth hormone variant | P01242 | GH2 |  |  |  |  |
| 2744 | 16749-79 | BMP-3b | Growth/differentiation factor 10 | P55107 | GDF10 |  |  |  |  |
| 2745 | 6517-14 | BMP-3b | Growth/differentiation factor 10 | P55107 | GDF10 |  |  |  | X |
| 2746 | 14587-16 | GDF-11 | Growth/differentiation factor 11 | O95390 | GDF11 |  |  |  |  |
| 2747 | 2765-4 | GDF-11/8 | Growth/differentiation factor 11/8 | O95390|O14793 | GDF11|MSTN | X |  |  |  |
| 2748 | 4374-45 | MIC-1 | Growth/differentiation factor 15 | Q99988 | GDF15 | X |  |  | X |
| 2749 | 4880-21 | GDF2 | Growth/differentiation factor 2 | Q9UK05 | GDF2 | X |  |  |  |
| 2750 | 16755-195 | GDF-3 | Growth/differentiation factor 3 | Q9NR23 | GDF3 |  |  |  | X |
| 2751 | 2752-62 | BMP-14 | Growth/differentiation factor 5 | P43026 | GDF5 |  | X |  |  |
| 2752 | 16756-30 | GDF7 | Growth/differentiation factor 7 | Q7Z4P5 | GDF7 |  |  |  |  |
| 2753 | 22967-15 | GDF7 | Growth/differentiation factor 7 | Q7Z4P5 | GDF7 |  |  |  |  |
| 2754 | 12077-32 | Myostatin | Growth/differentiation factor 8 | O14793 | MSTN | X |  |  |  |
| 2755 | 14583-49 | Myostatin | Growth/differentiation factor 8 | O14793 | MSTN |  |  |  |  |
| 2756 | 3067-67 | GDF-9 | Growth/differentiation factor 9 | O60383 | GDF9 |  |  |  |  |
| 2757 | 2985-35 | Gro-a | Growth-regulated alpha protein | P09341 | CXCL1 | X | X |  |  |
| 2758 | 7113-1 | GRPE1 | GrpE protein homolog 1, mitochondrial | Q9HAV7 | GRPEL1 |  | X |  |  |
| 2759 | 12849-25 | GSKIP | GSK3-beta interaction protein | Q9P0R6 | GSKIP |  |  |  |  |
| 2760 | 11185-145 | GCH1 | GTP cyclohydrolase 1 | P30793 | GCH1 | X |  | X |  |
| 2761 | 21118-48 | GFRP | GTP cyclohydrolase I feedback regulatory protein | P30047 | GCHFR |  |  |  |  |

| # | **Custom Panel (X)** | **SOMAmer SeqID** | **Target Name** | **Human Target or Analyte** | **UniProt ID** | **GeneID** | **Cardiovascular Disease** | **Inflammation and Immune**  **Response** | **Metabolic Disease** | **Oncology** |
| --- | --- | --- | --- | --- | --- | --- | --- | --- | --- | --- |

| 2762 | 18900-37 | H-ras (WT) | GTPase HRas | P01112 | HRAS | X | X |  | X |
| --- | --- | --- | --- | --- | --- | --- | --- | --- | --- |
| 2763 | 24684-7 | GIMA4 | GTPase IMAP family member 4 | Q9NUV9 | GIMAP4 |  |  |  |  |
| 2764 | 19302-7 | GIMA6 | GTPase IMAP family member 6 | Q6P9H5 | GIMAP6 |  |  |  |  |
| 2765 | 24683-11 | GIMA7 | GTPase IMAP family member 7 | Q8NHV1 | GIMAP7 |  |  |  |  |
| 2766 | 23522-1 | GIMD1 | GTPase IMAP family member GIMD1 | P0DJR0 | GIMD1 |  |  |  |  |
| 2767 | 20073-22 | K-ras | GTPase KRas | P01116 | KRAS | X | X | X | X |
| 2768 | 5193-51 | K-ras | GTPase KRas | P01116 | KRAS |  |  |  |  |
| 2769 | 10531-18 | RASN | GTPase NRas | P01111 | NRAS | X | X |  | X |
| 2770 | 4282-3 | RAN | GTP-binding nuclear protein Ran | P62826 | RAN |  | X |  | X |
| 2771 | 24712-6 | GTPBA | GTP-binding protein 10 | A4D1E9 | GTPBP10 |  |  |  |  |
| 2772 | 17793-4 | DIRA1 | GTP-binding protein Di-Ras1 | O95057 | DIRAS1 |  |  |  |  |
| 2773 | 12406-119 | ARHI | GTP-binding protein Di-Ras3 | O95661 | DIRAS3 |  |  |  |  |
| 2774 | 12817-1 | GEM | GTP-binding protein GEM | P55040 | GEM |  |  |  |  |
| 2775 | 23555-11 | RAD | GTP-binding protein RAD | P55042 | RRAD | X |  |  | X |
| 2776 | 24665-48 | REM1 | GTP-binding protein REM 1 | O75628 | REM1 |  |  |  |  |
| 2777 | 17726-3 | SAR1A | GTP-binding protein SAR1a | Q9NR31 | SAR1A |  |  |  |  |
| 2778 | 23024-25 | SAR1A | GTP-binding protein SAR1a | Q9NR31 | SAR1A |  |  |  |  |
| 2779 | 21220-11 | SAR1B | GTP-binding protein SAR1b | Q9Y6B6 | SAR1B |  |  |  |  |
| 2780 | 21660-4 | SAR1B | GTP-binding protein SAR1b | Q9Y6B6 | SAR1B |  |  | X |  |
| 2781 | 12667-2 | GUAD | Guanine deaminase | Q9Y2T3 | GDA |  |  |  | X |
| 2782 | 13934-3 | MCF2L | Guanine nucleotide exchange factor DBS | O15068 | MCF2L |  |  |  |  |
| 2783 | 21375-34 | R3GEF | Guanine nucleotide exchange factor for Rab-3A | Q8TBN0 | RAB3IL1 |  |  |  |  |
| 2784 | 18483-36 | MSS4 | Guanine nucleotide exchange factor MSS4 | P47224 | RABIF |  |  |  |  |
| 2785 | 9830-109 | VAV3 | Guanine nucleotide exchange factor VAV3 | Q9UKW4 | VAV3 | X | X |  |  |
| 2786 | 19271-64 | GNAI1 | Guanine nucleotide-binding protein G(i) subunit alpha-1 | P63096 | GNAI1 |  |  |  |  |
| 2787 | 25211-14 | GBG12 | Guanine nucleotide-binding protein G(I)/G(S)/G(O) gamma-12 subunit | Q9UBI6 | GNG12 |  |  |  |  |
| 2788 | 18282-1 | GBG11 | Guanine nucleotide-binding protein G(I)/G(S)/G(O) subunit gamma-11 | P61952 | GNG11 |  | X |  |  |
| 2789 | 10917-40 | GBGT2 | Guanine nucleotide-binding protein G(I)/G(S)/G(O) subunit gamma-T2 | O14610 | GNGT2 |  |  |  |  |
| 2790 | 12650-43 | GNAI3 | Guanine nucleotide-binding protein G(k) subunit alpha | P08754 | GNAI3 |  |  | X |  |
| 2791 | 23567-37 | GNAQ | Guanine nucleotide-binding protein G(q) subunit alpha | P50148 | GNAQ | X |  |  | X |
| 2792 | 25253-17 | GNAS | Guanine nucleotide-binding protein G(s) subunit alpha isoforms | Q5JWF2|P63092 | GNAS|GNAS | X | X | X | X |
| 2793 | 21857-26 | RACK1 | Guanine nucleotide-binding protein subunit beta-2-like 1 | P63244 | RACK1 |  |  |  |  |
| 2794 | 6223-5 | GUC2B | Guanylate cyclase activator 2B | Q16661 | GUCA2B |  |  |  |  |
| 2795 | 17325-10 | KGUA | Guanylate kinase | Q16774 | GUK1 |  |  |  |  |
| 2796 | 15326-64 | GBP1 | Guanylate-binding protein 1 | P32455 | GBP1 |  | X |  |  |
| 2797 | 18891-98 | GBP2 | Guanylate-binding protein 2 | P32456 | GBP2 | X |  |  |  |
| 2798 | 20211-75 | GBP5 | Guanylate-binding protein 5 | Q96PP8 | GBP5 |  |  | X |  |
| 2799 | 7818-101 | GBP6 | Guanylate-binding protein 6 | Q6ZN66 | GBP6 |  |  |  |  |
| 2800 | 10008-43 | GUC1A | Guanylyl cyclase-activating protein 1 | P43080 | GUCA1A |  |  |  |  |
| 2801 | 22401-26 | GUC1B | Guanylyl cyclase-activating protein 2 | Q9UMX6 | GUCA1B |  |  |  |  |
| 2802 | 20939-113 | NHP2 | H/ACA ribonucleoprotein complex subunit 2 | Q9NX24 | NHP2 |  |  |  |  |
| 2803 | 22374-56 | HEY1 | Hairy/enhancer-of-split related with YRPW motif protein 1 | Q9Y5J3 | HEY1 |  |  |  |  |
| 2804 | 24640-63 | HEY1 | Hairy/enhancer-of-split related with YRPW motif protein 1 | Q9Y5J3 | HEY1 |  |  |  | X |
| 2805 | 13472-35 | HDHD2 | Haloacid dehalogenase-like hydrolase domain-containing protein 2 | Q9H0R4 | HDHD2 |  |  |  |  |
| 2806 | 19482-11 | HDHD3 | Haloacid dehalogenase-like hydrolase domain-containing protein 3 | Q9BSH5 | HDHD3 |  |  |  |  |
| 2807 | 3054-3 | Haptoglobin, Mixed Type | Haptoglobin | P00738 | HP | X | X | X | X |
| 2808 | 7905-30 | HPT | Haptoglobin isoform 2 | P00738 | HP | X | X |  | X |
| 2809 | 17725-37 | USH1C | Harmonin | Q9Y6N9 | USH1C |  |  |  |  |
| 2810 | 21306-7 | HAUS1 | HAUS augmin-like complex subunit 1 | Q96CS2 | HAUS1 |  |  |  |  |
| 2811 | 21206-218 | HAX1 | HCLS1-associated protein X-1 | O00165 | HAX1 |  |  |  |  |
| 2812 | 25267-10 | HS12A | Heat shock 70 kDa protein 12A | O43301 | HSPA12A |  |  |  |  |
| 2813 | 17515-6 | STCH | Heat shock 70 kDa protein 13 | P48723 | HSPA13 |  |  |  |  |
| 2814 | 10721-76 | HSP 70 | Heat shock 70 kDa protein 1A | P0DMV8 | HSPA1A |  |  |  |  |
| 2815 | 10749-18 | HSP 70 | Heat shock 70 kDa protein 1A | P0DMV8 | HSPA1A |  |  |  |  |
| 2816 | 10803-22 | HSP 70 | Heat shock 70 kDa protein 1A | P0DMV8 | HSPA1A |  |  |  |  |
| 2817 | 11157-35 | HSP 70 | Heat shock 70 kDa protein 1A | P0DMV8 | HSPA1A |  |  |  |  |
| 2818 | 14237-1 | HSP 70 | Heat shock 70 kDa protein 1A | P0DMV8 | HSPA1A |  |  |  |  |
| 2819 | 16780-6 | HSP 70 | Heat shock 70 kDa protein 1A | P0DMV8 | HSPA1A |  |  |  |  |
| 2820 | 4124-24 | HSP 70 | Heat shock 70 kDa protein 1A | P0DMV8 | HSPA1A |  |  |  |  |
| 2821 | 6563-78 | HSP 70 | Heat shock 70 kDa protein 1A | P0DMV8 | HSPA1A | X | X |  | X |
| 2822 | 7219-152 | HSP 70 | Heat shock 70 kDa protein 1A | P0DMV8 | HSPA1A |  |  |  |  |
| 2823 | 18901-26 | HS71B | Heat shock 70 kDa protein 1B | P0DMV9 | HSPA1B | X |  |  | X |
| 2824 | 12041-33 | HS71L | Heat shock 70 kDa protein 1-like | P34931 | HSPA1L |  | X |  |  |
| 2825 | 13672-3 | HSP76 | Heat shock 70 kDa protein 6 | P17066 | HSPA6 |  |  |  |  |
| 2826 | 20213-82 | HSP76 | Heat shock 70 kDa protein 6 | P17066 | HSPA6 |  | X |  |  |
| 2827 | 5903-91 | HSP70 protein 8 | Heat shock cognate 71 kDa protein | P11142 | HSPA8 | X |  |  | X |
| 2828 | 19139-3 | HSF2B | Heat shock factor 2-binding protein | O75031 | HSF2BP |  |  |  |  |
| 2829 | 11616-9 | HSF1 | Heat shock factor protein 1 | Q00613 | HSF1 |  |  | X |  |
| 2830 | 17704-74 | HS105 | Heat shock protein 105 kDa | Q92598 | HSPH1 |  | X |  |  |
| 2831 | 11103-24 | HSP 27 | Heat shock protein beta-1 | P04792 | HSPB1 | X | X |  | X |
| 2832 | 23324-6 | HSPB3 | Heat shock protein beta-3 | Q12988 | HSPB3 |  |  |  |  |
| 2833 | 19127-1 | HSPB6 | Heat shock protein beta-6 | O14558 | HSPB6 | X |  |  | X |
| 2834 | 5467-15 | HSP 90b | Heat shock protein HSP 90-beta | P08238 | HSP90AB1 |  | X |  |  |
| 2835 | 22043-174 | HSP70 protein 2 | Heat shock-related 70 kDa protein 2 | P54652 | HSPA2 | X |  |  |  |
| 2836 | 10833-64 | HHIP | Hedgehog-interacting protein | Q96QV1 | HHIP |  |  |  | X |

| # | **Custom Panel (X)** | **SOMAmer SeqID** | **Target Name** | **Human Target or Analyte** | **UniProt ID** | **GeneID** | **Cardiovascular Disease** | **Inflammation and Immune**  **Response** | **Metabolic Disease** | **Oncology** |
| --- | --- | --- | --- | --- | --- | --- | --- | --- | --- | --- |

| 2837 | 19335-2 | HN1 | Hematological and neurological expressed 1 protein | Q9UK76 | JPT1 |  |  |  | |
| --- | --- | --- | --- | --- | --- | --- | --- | --- | --- |
| 2838 | 20110-25 | HN1L | Hematological and neurological expressed 1-like protein | Q9H910 | JPT2 |  |  |
| 2839 | 21439-37 | HCLS1 | Hematopoietic lineage cell-specific protein | P14317 | HCLS1 |  | X |
| 2840 | 11285-8 | CD34 | Hematopoietic progenitor cell antigen CD34 | P28906 | CD34 |  |  |
| 2841 | 12549-33 | PTGD2 | Hematopoietic prostaglandin D synthase | O60760 | HPGDS |  |  |
| 2842 | 23531-79 | HSH2D | Hematopoietic SH2 domain-containing protein | Q96JZ2 | HSH2D |  |  |
| 2843 | 20425-12 | HHEX | Hematopoietically-expressed homeobox protein HHEX | Q03014 | HHEX |  |  | X | X |
| 2844 | 17398-55 | HO-1 | Heme oxygenase 1 | P09601 | HMOX1 | X | X | X | X |
| 2845 | 2622-18 | HO-2 | Heme oxygenase 2 | P30519 | HMOX2 | X |  |  |  |
| 2846 | 18225-13 | HEBP1 | Heme-binding protein 1 | Q9NRV9 | HEBP1 |  |  |  |  |
| 2847 | 11096-57 | HEMK2 | HemK methyltransferase family member 2 | Q9Y5N5 | N6AMT1 |  |  |  |  |
| 2848 | 4915-64 | Hemoglobin | Hemoglobin | P69905|P68871 | HBA1|HBB | X | X | X | X |
| 2849 | 17137-160 | Beta-globin | Hemoglobin subunit beta | P68871 | HBB |  | X |  |  |
| 2850 | 6992-67 | HBD | Hemoglobin subunit delta | P02042 | HBD |  |  |  |  |
| 2851 | 7136-107 | Hemoglobin epsilon chain | Hemoglobin subunit epsilon | P02100 | HBE1 |  |  |  |  |
| 2852 | 19774-8 | HBG2 | Hemoglobin subunit gamma-2 | P69892 | HBG2 |  |  |  |  |
| 2853 | 18198-51 | HBAT | Hemoglobin subunit theta-1 | P09105 | HBQ1 |  |  |  |  |
| 2854 | 7965-25 | HBAT | Hemoglobin subunit theta-1 | P09105 | HBQ1 |  |  |  |  |
| 2855 | 6919-3 | HBAZ | Hemoglobin subunit zeta | P02008 | HBZ |  |  |  |  |
| 2856 | 3332-57 | RGM-C | Hemojuvelin | Q6ZVN8 | HJV |  |  | X |  |
| 2857 | 14073-31 | Hemopexin | Hemopexin | P02790 | HPX |  |  |  |  |
| 2858 | 15347-12 | Hemopexin | Hemopexin | P02790 | HPX |  |  |  |  |
| 2859 | 2768-56 | Hemopexin | Hemopexin | P02790 | HPX | X | X | X |  |
| 2860 | 9116-28 | HECA2:CD | HEPACAM family member 2:Isoform 1, Cytoplasmic domain | A8MVW5 | HEPACAM2 |  |  |  |  |
| 2861 | 6568-18 | HECA2:ECD | HEPACAM family member 2:Isoform 2, Extracellular domain | A8MVW5 | HEPACAM2 |  |  |  |  |
| 2862 | 21736-60 | HS2ST | Heparan sulfate 2-O-sulfotransferase 1 | Q7LGA3 | HS2ST1 |  |  |  |  |
| 2863 | 15497-9 | HS3S1 | Heparan sulfate glucosamine 3-O-sulfotransferase 1 | O14792 | HS3ST1 |  |  |  |  |
| 2864 | 8268-98 | HS3SA | Heparan sulfate glucosamine 3-O-sulfotransferase 3A1 | Q9Y663 | HS3ST3A1 |  |  |  |  |
| 2865 | 6986-17 | HS3SB | Heparan sulfate glucosamine 3-O-sulfotransferase 3B1 | Q9Y662 | HS3ST3B1 |  |  |  |  |
| 2866 | 19370-30 | HS3S4 | Heparan sulfate glucosamine 3-O-sulfotransferase 4 | Q9Y661 | HS3ST4 |  |  |  |  |
| 2867 | 8998-15 | HS3S4 | Heparan sulfate glucosamine 3-O-sulfotransferase 4 | Q9Y661 | HS3ST4 |  |  |  |  |
| 2868 | 10731-10 | HS3S5 | Heparan sulfate glucosamine 3-O-sulfotransferase 5 | Q8IZT8 | HS3ST5 |  |  |  |  |
| 2869 | 5604-30 | HPSE | Heparanase | Q9Y251 | HPSE |  | X |  | X |
| 2870 | 5465-32 | H6ST1 | Heparan-sulfate 6-O-sulfotransferase 1 | O60243 | HS6ST1 |  |  |  |  |
| 2871 | 13524-25 | H6ST2 | Heparan-sulfate 6-O-sulfotransferase 2 | Q96MM7 | HS6ST2 |  |  |  |  |
| 2872 | 18896-23 | H6ST3 | Heparan-sulfate 6-O-sulfotransferase 3 | Q8IZP7 | HS6ST3 |  |  |  |  |
| 2873 | 3316-58 | Heparin cofactor II | Heparin cofactor 2 | P05546 | SERPIND1 | X |  |  |  |
| 2874 | 14094-29 | HB-EGF | Heparin-binding EGF-like growth factor | Q99075 | HBEGF | X | X |  |  |
| 2875 | 21340-38 | HLF | Hepatic leukemia factor | Q16534 | HLF |  | X |  | X |
| 2876 | 9021-1 | TIM-1 | Hepatitis A virus cellular receptor 1 | Q96D42 | HAVCR1 | X |  |  |  |
| 2877 | 11481-25 | TIMD3 | Hepatitis A virus cellular receptor 2 | Q8TDQ0 | HAVCR2 |  | X |  |  |
| 2878 | 5134-52 | TIMD3 | Hepatitis A virus cellular receptor 2 | Q8TDQ0 | HAVCR2 |  |  |  |  |
| 2879 | 7152-5 | TIMD3 | Hepatitis A virus cellular receptor 2 | Q8TDQ0 | HAVCR2 |  |  |  |  |
| 2880 | 8810-26 | HEPACAM | Hepatocyte cell adhesion molecule | Q14CZ8 | HEPACAM |  |  | X |  |
| 2881 | 2681-23 | HGF | Hepatocyte growth factor | P14210 | HGF | X | X | X | X |
| 2882 | 3617-80 | HGFA | Hepatocyte growth factor activator | Q04756 | HGFAC |  |  |  |  |
| 2883 | 8385-248 | HGFA | Hepatocyte growth factor activator | Q04756 | HGFAC |  |  |  |  |
| 2884 | 11814-29 | Met | Hepatocyte growth factor receptor | P08581 | MET | X | X | X | X |
| 2885 | 2837-3 | Met | Hepatocyte growth factor receptor | P08581 | MET |  |  |  |  |
| 2886 | 4407-10 | MSP | Hepatocyte growth factor-like protein | P26927 | MST1 |  |  |  | X |
| 2887 | 13644-30 | HGS | Hepatocyte growth factor-regulated tyrosine kinase substrate | O14964 | HGS |  |  |  |  |
| 2888 | 11193-27 | HNF1A | Hepatocyte nuclear factor 1-alpha | P20823 | HNF1A | X | X | X | X |
| 2889 | 10041-3 | HNF4A | Hepatocyte nuclear factor 4-alpha | P41235 | HNF4A |  |  | X |  |
| 2890 | 16758-96 | HDGF | Hepatoma-derived growth factor | P51858 | HDGF |  |  |  |  |
| 2891 | 8953-47 | HDGF | Hepatoma-derived growth factor | P51858 | HDGF |  | X |  |  |
| 2892 | 18898-36 | HDGL1 | Hepatoma-derived growth factor-like protein 1 | Q5TGJ6 | HDGFL1 |  |  |  |  |
| 2893 | 4553-65 | HDGR2 | Hepatoma-derived growth factor-related protein 2 | Q7Z4V5 | HDGFL2 |  |  |  |  |
| 2894 | 18899-82 | HDGR3 | Hepatoma-derived growth factor-related protein 3 | Q9Y3E1 | HDGFL3 |  |  |  |  |
| 2895 | 3504-58 | LEAP-1 | Hepcidin | P81172 | HAMP | X |  | X |  |
| 2896 | 20127-102 | HEPH | Hephaestin | Q9BQS7 | HEPH |  |  |  |  |
| 2897 | 6354-13 | HPHL1 | Hephaestin-like protein 1 | Q6MZM0 | HEPHL1 |  |  |  |  |
| 2898 | 14132-21 | HHLA2 | HERV-H LTR-associating protein 2 | Q9UM44 | HHLA2 |  |  |  |  |
| 2899 | 22467-94 | HHLA3 | HERV-H LTR-associating protein 3 | Q9XRX5 | HHLA3 |  |  |  |  |
| 2900 | 4450-26 | hnRNP A/B | Heterogeneous nuclear ribonucleoprotein A/B | Q99729 | HNRNPAB |  |  |  |  |
| 2901 | 8894-80 | hnRNP A/B | Heterogeneous nuclear ribonucleoprotein A/B | Q99729 | HNRNPAB |  |  |  |  |
| 2902 | 24697-48 | ROA0 | Heterogeneous nuclear ribonucleoprotein A0 | Q13151 | HNRNPA0 |  |  |  |  |
| 2903 | 12466-7 | ROA1 | Heterogeneous nuclear ribonucleoprotein A1 | P09651 | HNRNPA1 |  |  | X | X |
| 2904 | 20137-49 | ROA1 | Heterogeneous nuclear ribonucleoprotein A1 | P09651 | HNRNPA1 |  |  |  |  |
| 2905 | 18348-89 | HNRPD | Heterogeneous nuclear ribonucleoprotein D0 | Q14103 | HNRNPD |  |  |  |  |
| 2906 | 10852-114 | HNRDL | Heterogeneous nuclear ribonucleoprotein D-like | O14979 | HNRNPDL |  |  |  |  |
| 2907 | 9764-79 | HNRPF | Heterogeneous nuclear ribonucleoprotein F | P52597 | HNRNPF |  |  |  |  |
| 2908 | 14309-8 | HNRH1 | Heterogeneous nuclear ribonucleoprotein H | P31943 | HNRNPH1 |  | X |  |  |
| 2909 | 19333-4 | hnRNP K | Heterogeneous nuclear ribonucleoprotein K | P61978 | HNRNPK |  |  |  | X |
| 2910 | 23657-102 | HNRLL | Heterogeneous nuclear ribonucleoprotein L-like | Q8WVV9 | HNRNPLL |  |  |  |  |
| 2911 | 12783-29 | HNRPM | Heterogeneous nuclear ribonucleoprotein M | P52272 | HNRNPM |  |  |  |  |

| # | **Custom Panel (X)** | **SOMAmer SeqID** | **Target Name** | **Human Target or Analyte** | **UniProt ID** | **GeneID** | **Cardiovascular Disease** | **Inflammation and Immune**  **Response** | **Metabolic Disease** | **Oncology** |
| --- | --- | --- | --- | --- | --- | --- | --- | --- | --- | --- |

| 2912 | 4224-7 | HNRPQ | Heterogeneous nuclear ribonucleoprotein Q | O60506 | SYNCRIP |  | | | |
| --- | --- | --- | --- | --- | --- | --- | --- | --- | --- |
| 2913 | 13504-147 | HNRPR | Heterogeneous nuclear ribonucleoprotein R | O43390 | HNRNPR |
| 2914 | 13512-28 | HNRPR | Heterogeneous nuclear ribonucleoprotein R | O43390 | HNRNPR |  |  |  | X |
| 2915 | 5351-52 | hnRNP A2/B1 | Heterogeneous nuclear ribonucleoproteins A2/B1 | P22626 | HNRNPA2B1 |  | X | X |  |
| 2916 | 11429-80 | hnRNP C1/C2 | Heterogeneous nuclear ribonucleoproteins C1/C2 | P07910 | HNRNPC |  |  |  |  |
| 2917 | 13131-5 | HXK1 | Hexokinase-1 | P19367 | HK1 | X |  | X |  |
| 2918 | 13130-150 | HXK2 | Hexokinase-2 | P52789 | HK2 | X |  | X |  |
| 2919 | 19518-12 | HXK3 | Hexokinase-3 | P52790 | HK3 |  |  |  |  |
| 2920 | 5178-5 | PDE7A | High affinity cAMP-specific 3',5'-cyclic phosphodiesterase 7A | Q13946 | PDE7A |  |  |  |  |
| 2921 | 5201-50 | PDE9A | High affinity cGMP-specific 3',5'-cyclic phosphodiesterase 9A | O76083 | PDE9A |  |  |  |  |
| 2922 | 9568-289 | FCAMR | High affinity immunoglobulin alpha and immunoglobulin mu Fc receptor | Q8WWV6 | FCAMR |  |  |  |  |
| 2923 | 9254-18 | FCERA | High affinity immunoglobulin epsilon receptor subunit alpha | P12319 | FCER1A |  | X |  |  |
| 2924 | 3312-64 | FCGR1 | High affinity immunoglobulin gamma Fc receptor I | P12314 | FCGR1A | X | X | X |  |
| 2925 | 3477-63 | TrkA | High affinity nerve growth factor receptor | P04629 | NTRK1 |  | X |  | X |
| 2926 | 20390-4 | HMGN3 | High mobility group nucleosome-binding domain-containing protein 3 | Q15651 | HMGN3 |  |  |  |  |
| 2927 | 12712-9 | HM20A | High mobility group protein 20A | Q9NP66 | HMG20A |  |  | X |  |
| 2928 | 2524-56 | HMG-1 | High mobility group protein B1 | P09429 | HMGB1 | X | X |  |  |
| 2929 | 22978-13 | HMG-2 | High mobility group protein B2 | P26583 | HMGB2 |  |  | X |  |
| 2930 | 6913-189 | HMG-2 | High mobility group protein B2 | P26583 | HMGB2 |  |  |  |  |
| 2931 | 12775-6 | HMGB3 | High mobility group protein B3 | O15347 | HMGB3 |  |  |  |  |
| 2932 | 16536-3 | HMGA1 | High mobility group protein HMG-I/HMG-Y | P17096 | HMGA1 |  |  | X | X |
| 2933 | 19516-10 | HMGA2 | High mobility group protein HMGI-C | P52926 | HMGA2 |  |  |  | X |
| 2934 | 17791-25 | HPCL1 | Hippocalcin-like protein 1 | P37235 | HPCAL1 |  |  |  |  |
| 2935 | 22981-3 | HPCL1 | Hippocalcin-like protein 1 | P37235 | HPCAL1 |  |  |  |  |
| 2936 | 24921-73 | HIRP3 | HIRA-interacting protein 3 | Q9BW71 | HIRIP3 |  |  |  |  |
| 2937 | 23178-95 | HNMT | Histamine N-methyltransferase | P50135 | HNMT |  | X |  |  |
| 2938 | 10608-9 | HIS1 | Histatin-1 | P15515 | HTN1 |  |  |  |  |
| 2939 | 10603-1 | HIS3 | Histatin-3 | P15516 | HTN3 |  |  |  |  |
| 2940 | 5900-11 | HINT1 | Histidine triad nucleotide-binding protein 1 | P49773 | HINT1 |  |  |  |  |
| 2941 | 5612-16 | HINT2 | Histidine triad nucleotide-binding protein 2, mitochondrial | Q9BX68 | HINT2 |  |  |  |  |
| 2942 | 4996-66 | HRG | Histidine-rich glycoprotein | P04196 | HRG |  |  |  | X |
| 2943 | 17450-51 | Histidyl-tRNA synthetase | Histidine--tRNA ligase, cytoplasmic | P12081 | HARS1 |  |  |  | X |
| 2944 | 9253-52 | BGAT | Histo-blood group ABO system transferase | P16442 | ABO | X |  |  | X |
| 2945 | 19369-17 | GCNL2 | Histone acetyltransferase KAT2A | Q92830 | KAT2A |  |  |  |  |
| 2946 | 19797-4 | GCNL2 | Histone acetyltransferase KAT2A | Q92830 | KAT2A |  |  |  |  |
| 2947 | 12368-18 | PCAF | Histone acetyltransferase KAT2B | Q92831 | KAT2B |  |  |  |  |
| 2948 | 25879-6 | KAT5 | Histone acetyltransferase KAT5 | Q92993 | KAT5 |  |  |  |  |
| 2949 | 19327-31 | Hat1 | Histone acetyltransferase type B catalytic subunit | O14929 | HAT1 |  |  |  |  |
| 2950 | 2858-29 | Hat1 | Histone acetyltransferase type B catalytic subunit | O14929 | HAT1 |  |  |  |  |
| 2951 | 18172-71 | ASF1A | Histone chaperone ASF1A | Q9Y294 | ASF1A |  |  |  |  |
| 2952 | 20057-177 | ASF1B | Histone chaperone ASF1B | Q9NVP2 | ASF1B |  |  |  |  |
| 2953 | 18897-31 | HDAC2 | Histone deacetylase 2 | Q92769 | HDAC2 | X |  |  | X |
| 2954 | 25275-30 | HDAC4 | Histone deacetylase 4 | P56524 | HDAC4 |  |  |  |  |
| 2955 | 25059-18 | HDAC6 | Histone deacetylase 6 | Q9UBN7 | HDAC6 |  |  |  | X |
| 2956 | 2859-69 | HDAC8 | Histone deacetylase 8 | Q9BY41 | HDAC8 |  |  | X |  |
| 2957 | 12830-4 | SAP18 | Histone deacetylase complex subunit SAP18 | O00422 | SAP18 |  |  |  |  |
| 2958 | 12888-18 | SAP30 | Histone deacetylase complex subunit SAP30 | O75446 | SAP30 |  |  |  |  |
| 2959 | 22391-34 | SP30L | Histone deacetylase complex subunit SAP30L | Q9HAJ7 | SAP30L |  |  |  |  |
| 2960 | 2987-37 | Histone H1.2 | Histone H1.2 | P16403 | H1-2 |  |  |  | X |
| 2961 | 12709-63 | H1X | Histone H1x | Q92522 | H1-10 |  |  |  |  |
| 2962 | 11536-9 | MYSM1 | Histone H2A deubiquitinase MYSM1 | Q5VVJ2 | MYSM1 |  | X |  |  |
| 2963 | 22468-54 | Histone H2A type 1 | Histone H2A type 1 | P0C0S8 | H2AC11 |  |  |  |  |
| 2964 | 22402-12 | H2A1A | Histone H2A type 1-A | Q96QV6 | H2AC1 |  |  |  |  |
| 2965 | 14144-3 | H2A3 | Histone H2A type 3 | Q7L7L0 | H2AW |  |  |  |  |
| 2966 | 4163-5 | Histone H2A.z | Histone H2A.z | P0C0S5 | H2AZ1 |  |  |  |  |
| 2967 | 22403-13 | H2B1K | Histone H2B type 1-K | O60814 | H2BC12 |  |  |  | X |
| 2968 | 14143-8 | H2B2E | Histone H2B type 2-E | Q16778 | H2BC21 |  |  |  |  |
| 2969 | 22974-25 | H2B2E | Histone H2B type 2-E | Q16778 | H2BC21 |  |  |  |  |
| 2970 | 18823-52 | H2B3B | Histone H2B type 3-B | Q8N257 | H2BU1 |  |  |  |  |
| 2971 | 14146-92 | H31 | Histone H3.1 | P68431 | H3C1 |  |  |  |  |
| 2972 | 24648-8 | SLBP | Histone RNA hairpin-binding protein | Q14493 | SLBP |  |  |  |  |
| 2973 | 15331-47 | RBBP4 | Histone-binding protein RBBP4 | Q09028 | RBBP4 |  |  |  |  |
| 2974 | 11402-17 | KMT2C | Histone-lysine N-methyltransferase 2C | Q8NEZ4 | KMT2C |  | X |  | X |
| 2975 | 13623-4 | MLL2 | Histone-lysine N-methyltransferase 2D | O14686 | KMT2D |  | X |  | X |
| 2976 | 12622-96 | ASH1L | Histone-lysine N-methyltransferase ASH1L | Q9NR48 | ASH1L |  |  |  |  |
| 2977 | 5843-60 | NG36 | Histone-lysine N-methyltransferase EHMT2 | Q96KQ7 | EHMT2 |  | X |  | X |
| 2978 | 12647-52 | SETD2 | Histone-lysine N-methyltransferase SETD2 | Q9BYW2 | SETD2 |  | X |  | X |
| 2979 | 24260-4 | SETD3 | Histone-lysine N-methyltransferase setd3 | Q86TU7 | SETD3 |  |  |  |  |
| 2980 | 12462-20 | SETMR | Histone-lysine N-methyltransferase SETMAR | Q53H47 | SETMAR |  |  |  |  |
| 2981 | 12452-32 | SV422 | Histone-lysine N-methyltransferase SUV420H2 | Q86Y97 | KMT5C |  |  |  |  |
| 2982 | 12692-56 | DOT1L | Histone-lysine N-methyltransferase, H3 lysine-79 specific | Q8TEK3 | DOT1L |  |  |  |  |
| 2983 | 25918-60 | HLAE | HLA class I histocompatibility antigen, alpha chain E | P13747 | HLA-E |  |  |  | X |
| 2984 | 19636-23 | HLA-G | HLA class I histocompatibility antigen, alpha chain G | P17693 | HLA-G |  | X |  |  |
| 2985 | 6974-6 | HG2A | HLA class II histocompatibility antigen gamma chain | P04233 | CD74 |  |  |  | X |
| 2986 | 8748-45 | HG2A | HLA class II histocompatibility antigen gamma chain | P04233 | CD74 |  |  |  |  |

| # | **Custom Panel (X)** | **SOMAmer SeqID** | **Target Name** | **Human Target or Analyte** | **UniProt ID** | **GeneID** | **Cardiovascular Disease** | **Inflammation and Immune**  **Response** | **Metabolic Disease** | **Oncology** |
| --- | --- | --- | --- | --- | --- | --- | --- | --- | --- | --- |

| 2987 | 7757-5 | DQA2 | HLA class II histocompatibility antigen, DQ alpha 2 chain | P01906 | HLA-DQA2 |  | X |  | |
| --- | --- | --- | --- | --- | --- | --- | --- | --- | --- |
| 2988 | 6962-5 | DRB3 | HLA class II histocompatibility antigen, DR beta 3 chain | P79483 | HLA-DRB3 |  |  |
| 2989 | 21797-4 | HLA-C | HLA-C | P10321 | HLA-C |  | X |  | X |
| 2990 | 5584-21 | Holo-TC II | Holo-Transcobalamin-2 | P20062 | TCN2 | X | X |  | X |
| 2991 | 22104-39 | CDX1 | Homeobox protein CDX-1 | P47902 | CDX1 |  |  |  |  |
| 2992 | 22123-15 | DLX2 | Homeobox protein DLX-2 | Q07687 | DLX2 |  |  |  |  |
| 2993 | 11422-2 | DLX3 | Homeobox protein DLX-3 | O60479 | DLX3 |  |  |  |  |
| 2994 | 11910-27 | DLX4 | Homeobox protein DLX-4 | Q92988 | DLX4 |  |  |  |  |
| 2995 | 22151-48 | GSC2 | Homeobox protein goosecoid-2 | O15499 | GSC2 |  |  |  |  |
| 2996 | 22469-103 | HMX2 | Homeobox protein HMX2 | A2RU54 | HMX2 |  |  |  |  |
| 2997 | 24418-14 | HMX3 | Homeobox protein HMX3 | A6NHT5 | HMX3 |  |  |  |  |
| 2998 | 22375-15 | HXA11 | Homeobox protein Hox-A11 | P31270 | HOXA11 |  |  |  |  |
| 2999 | 22376-95 | HXA5 | Homeobox protein Hox-A5 | P20719 | HOXA5 |  |  |  |  |
| 3000 | 22474-28 | HXC11 | Homeobox protein Hox-C11 | O43248 | HOXC11 |  |  |  |  |
| 3001 | 22476-115 | HXD4 | Homeobox protein Hox-D4 | P09016 | HOXD4 |  | X |  |  |
| 3002 | 23568-41 | MEIS2 | Homeobox protein Meis2 | O14770 | MEIS2 | X |  |  |  |
| 3003 | 22503-24 | MKX | Homeobox protein Mohawk | Q8IYA7 | MKX |  |  |  |  |
| 3004 | 20183-48 | MEOX1 | Homeobox protein MOX-1 | P50221 | MEOX1 |  | X |  |  |
| 3005 | 18259-15 | MEOX2 | Homeobox protein MOX-2 | P50222 | MEOX2 |  |  |  |  |
| 3006 | 23039-56 | MEOX2 | Homeobox protein MOX-2 | P50222 | MEOX2 | X |  |  |  |
| 3007 | 2732-58 | NANOG | Homeobox protein NANOG | Q9H9S0 | NANOG |  |  |  | X |
| 3008 | 22386-11 | OTX1 | Homeobox protein OTX1 | P32242 | OTX1 |  |  |  |  |
| 3009 | 22521-13 | OTX2 | Homeobox protein OTX2 | P32243 | OTX2 |  |  |  | X |
| 3010 | 20987-21 | SIX6 | Homeobox protein SIX6 | O95475 | SIX6 |  |  |  |  |
| 3011 | 9847-21 | TGIF2 | Homeobox protein TGIF2 | Q9GZN2 | TGIF2 |  |  |  |  |
| 3012 | 21164-83 | TF2LX | Homeobox protein TGIF2LX | Q8IUE1 | TGIF2LX |  |  |  |  |
| 3013 | 20447-11 | TF2LY | Homeobox protein TGIF2LY | Q8IUE0 | TGIF2LY |  |  |  |  |
| 3014 | 19329-31 | HIPK3 | Homeodomain-interacting protein kinase 3 | Q9H422 | HIPK3 |  |  |  |  |
| 3015 | 19196-73 | HOP | Homeodomain-only protein | Q9BPY8 | HOPX |  |  |  |  |
| 3016 | 19229-92 | HOME1 | Homer protein homolog 1 | Q86YM7 | HOMER1 |  |  |  |  |
| 3017 | 22980-37 | HOME1 | Homer protein homolog 1 | Q86YM7 | HOMER1 |  |  |  |  |
| 3018 | 12685-57 | HOME2 | Homer protein homolog 2 | Q9NSB8 | HOMER2 |  |  |  |  |
| 3019 | 21799-15 | HOME3 | Homer protein homolog 3 | Q9NSC5 | HOMER3 |  |  |  |  |
| 3020 | 9779-63 | HERP2 | Homocysteine-responsive endoplasmic reticulum-resident ubiquitin-like domain member 2 | Q9BSE4 | HERPUD2 |  | X |  |  |
| 3021 | 9832-33 | HGD | Homogentisate 1,2-dioxygenase | Q93099 | HGD |  |  | X |  |
| 3022 | 23309-11 | HOP2 | Homologous-pairing protein 2 homolog | Q9P2W1 | PSMC3IP |  |  |  |  |
| 3023 | 23405-33 | HORM2 | HORMA domain-containing protein 2 | Q8N7B1 | HORMAD2 |  |  |  |  |
| 3024 | 7822-11 | HRSL2 | HRAS-like suppressor 2 | Q9NWW9 | PLAAT2 |  |  |  |  |
| 3025 | 7865-126 | HRSL3 | HRAS-like suppressor 3 | P53816 | PLAAT3 |  |  |  |  |
| 3026 | 18387-7 | suppression of tumorigenicity 13 | Hsc70-interacting protein | P50502 | ST13 |  |  |  |  |
| 3027 | 21111-49 | HPBP1 | Hsp70-binding protein 1 | Q9NZL4 | HSPBP1 |  |  |  |  |
| 3028 | 3879-50 | CDC37 | Hsp90 co-chaperone Cdc37 | Q16543 | CDC37 |  |  |  |  |
| 3029 | 2625-53 | HSP 90a | Hsp90alpha | P07900 | HSP90AA1 |  | X |  | X |
| 3030 | 4914-10 | HCG | Human Chorionic Gonadotropin | P01215|P0DN86|P0DN87 | CGA|CGB3|CGB7 | X |  |  | X |
| 3031 | 24960-48 | HIP1R | Huntingtin-interacting protein 1-related protein | O75146 | HIP1R | X |  |  |  |
| 3032 | 23617-15 | HYPK | Huntingtin-interacting protein K | Q9NX55 | HYPK |  |  |  |  |
| 3033 | 3196-6 | HPLN1 | Hyaluronan and proteoglycan link protein 1 | P10915 | HAPLN1 |  | X |  |  |
| 3034 | 6455-52 | HPLN4 | Hyaluronan and proteoglycan link protein 4 | Q86UW8 | HAPLN4 |  |  |  | X |
| 3035 | 8309-12 | HYAL1 | Hyaluronidase-1 | Q12794 | HYAL1 |  |  | X |  |
| 3036 | 21718-150 | HYAL4 | Hyaluronidase-4 | Q2M3T9 | HYAL4 |  |  |  |  |
| 3037 | 17768-50 | HAOX1 | Hydroxyacid oxidase 1 | Q9UJM8 | HAO1 | X |  |  |  |
| 3038 | 17739-1 | HCDH | Hydroxyacyl-coenzyme A dehydrogenase, mitochondrial | Q16836 | HADH |  |  | X |  |
| 3039 | 19267-14 | GLO2 | Hydroxyacylglutathione hydrolase, mitochondrial | Q16775 | HAGH |  |  |  |  |
| 3040 | 23546-9 | HAGHL | Hydroxyacylglutathione hydrolase-like protein | Q6PII5 | HAGHL |  |  |  |  |
| 3041 | 13495-48 | HCAR2 | Hydroxycarboxylic acid receptor 2 | Q8TDS4 | HCAR2 |  |  |  |  |
| 3042 | 21157-6 | HYKK | Hydroxylysine kinase | A2RU49 | HYKK |  |  |  |  |
| 3043 | 24211-25 | HMGCL | Hydroxymethylglutaryl-CoA lyase, mitochondrial | P35914 | HMGCL |  |  | X |  |
| 3044 | 13496-19 | HMCS1 | Hydroxymethylglutaryl-CoA synthase, cytoplasmic | Q01581 | HMGCS1 |  |  |  |  |
| 3045 | 13704-5 | HMCS2 | Hydroxymethylglutaryl-CoA synthase, mitochondrial | P54868 | HMGCS2 | X |  | X | X |
| 3046 | 23588-9 | HSDL2 | Hydroxysteroid dehydrogenase-like protein 2 | Q6YN16 | HSDL2 |  |  |  |  |
| 3047 | 19290-5 | HPRT | Hypoxanthine-guanine phosphoribosyltransferase | P00492 | HPRT1 |  |  | X |  |
| 3048 | 21497-32 | HYOU1 | Hypoxia up-regulated protein 1 | Q9Y4L1 | HYOU1 |  | X |  |  |
| 3049 | 13089-6 | HIF-1a | Hypoxia-inducible factor 1-alpha | Q16665 | HIF1A | X |  |  | X |
| 3050 | 17199-43 | HIF1N | Hypoxia-inducible factor 1-alpha inhibitor | Q9NWT6 | HIF1AN |  |  |  |  |
| 3051 | 5061-27 | B7-H2 | ICOS ligand | O75144 | ICOSLG |  | X | X | X |
| 3052 | 9303-9 | B7-H2 | ICOS ligand | O75144 | ICOSLG |  |  |  |  |
| 3053 | 3198-4 | IDS | Iduronate 2-sulfatase | P22304 | IDS |  |  | X |  |
| 3054 | 13230-174 | IgG2, Kappa | Ig gamma-2, Kappa | P01859 | IGHG2 |  | X |  |  |
| 3055 | 13231-90 | IgG4, Kappa | Ig gamma-4, Kappa | P01861 | IGHG4 |  |  |  |  |
| 3056 | 6561-77 | Ig K chain V-I region HK102-like | Ig Kappa chain V-I region HK102- like | P01602 | IGKV1-5 |  |  |  |  |
| 3057 | 7244-16 | TM149 | IGF-like family receptor 1 | Q9H665 | IGFLR1 |  |  |  |  |
| 3058 | 21708-149 | FCGRN | IgG receptor FcRn large subunit p51 | P55899 | FCGRT |  |  |  |  |
| 3059 | 25910-1 | FCGBP | IgGFc-binding protein | Q9Y6R7 | FCGBP |  |  |  |  |
| 3060 | 6478-2 | IGLO5 | IgLON family member 5 | A6NGN9 | IGLON5 |  |  |  |  |
| 3061 | 21976-4 | IKK-gamma | I-kappa-B kinase gamma | Q9Y6K9 | IKBKG | X | X |  | X |

| # | **Custom Panel (X)** | **SOMAmer SeqID** | **Target Name** | **Human Target or Analyte** | **UniProt ID** | **GeneID** | **Cardiovascular Disease** | **Inflammation and Immune**  **Response** | **Metabolic Disease** | **Oncology** |
| --- | --- | --- | --- | --- | --- | --- | --- | --- | --- | --- |

| 3062 | 20071-53 | IKB-epsilon | I-kappa-B-epsilon | O00221 | NFKBIE |  | X |  | |
| --- | --- | --- | --- | --- | --- | --- | --- | --- | --- |
| 3063 | 21897-4 | IL-17/ IL-17F | IL-17/IL-17F | Q16552|Q96PD4 | IL17A|IL17F |  |  |
| 3064 | 21946-79 | IL-6/ IL-6 sRa Complex | IL-6/IL-6 sRa Complex | P05231|P08887 | IL6|IL6R |  |  |
| 3065 | 22377-27 | IER2 | Immediate early response gene 2 protein | Q9BTL4 | IER2 |  |  |
| 3066 | 11089-7 | IgA | Immunoglobulin A | P01876|P01877 | IGHA1|IGHA2 |  |  |
| 3067 | 4987-17 | FCAR | Immunoglobulin alpha Fc receptor | P24071 | FCAR |  |  |
| 3068 | 4916-2 | IgD | Immunoglobulin D | P01880 | IGHD |  |  |
| 3069 | 4135-84 | IgE | Immunoglobulin E | P01854 | IGHE |  |  |
| 3070 | 2744-57 | IgG | Immunoglobulin G | P01857 | IGHG1 |  | X |
| 3071 | 15306-20 | IgJ | Immunoglobulin J chain | P01591 | JCHAIN |  |  |
| 3072 | 6485-59 | IGLL1 | Immunoglobulin lambda-like polypeptide 1 | P15814 | IGLL1 |  | X |
| 3073 | 3069-52 | IgM | Immunoglobulin M | P01871 | IGHM |  | X |
| 3074 | 13124-20 | ISLR2 | Immunoglobulin superfamily containing leucine-rich repeat protein 2 | Q6UXK2 | ISLR2 |  |  |
| 3075 | 8528-74 | ISLR2 | Immunoglobulin superfamily containing leucine-rich repeat protein 2 | Q6UXK2 | ISLR2 |  |  |
| 3076 | 11952-1 | IGDC3:CD | Immunoglobulin superfamily DCC subclass member 3:Cytoplasmic domain | Q8IVU1 | IGDCC3 |  |  |
| 3077 | 7118-24 | IGDC3:ECD | Immunoglobulin superfamily DCC subclass member 3:Extracellular domain | Q8IVU1 | IGDCC3 |  |  |
| 3078 | 9793-145 | IGDC4 | Immunoglobulin superfamily DCC subclass member 4 | Q8TDY8 | IGDCC4 |  |  |
| 3079 | 10700-10 | IGS11:CD | Immunoglobulin superfamily member 11:Cytoplasmic domain | Q5DX21 | IGSF11 |  |  |
| 3080 | 9279-7 | IGS11:ECD | Immunoglobulin superfamily member 11:Extracellular domain | Q5DX21 | IGSF11 |  |  |
| 3081 | 9715-15 | IGSF3 | Immunoglobulin superfamily member 3 | O75054 | IGSF3 |  |  |
| 3082 | 6984-6 | IGSF8 | Immunoglobulin superfamily member 8 | Q969P0 | IGSF8 |  |  |
| 3083 | 12358-6 | IGBP1 | Immunoglobulin-binding protein 1 | P78318 | IGBP1 |  |  |  | X |
| 3084 | 2860-19 | Karyopherin-a2 | Importin subunit alpha-1 | P52292 | KPNA2 |  |  |  |  |
| 3085 | 12698-72 | IMA4 | Importin subunit alpha-3 | O00629 | KPNA4 |  |  |  |  |
| 3086 | 19587-12 | IMA5 | Importin subunit alpha-5 | P52294 | KPNA1 |  | X |  |  |
| 3087 | 21235-11 | IMA6 | Importin subunit alpha-6 | O15131 | KPNA5 |  |  |  |  |
| 3088 | 13969-24 | IMA7 | Importin subunit alpha-7 | O60684 | KPNA6 |  |  |  |  |
| 3089 | 3887-90 | IMB1 | Importin subunit beta-1 | Q14974 | KPNB1 |  | X |  |  |
| 3090 | 7890-68 | DPP10 | Inactive dipeptidyl peptidase 10 | Q8N608 | DPP10 |  | X |  |  |
| 3091 | 6334-9 | GGT2 | Inactive gamma-glutamyltranspeptidase 2 | P36268 | GGT2 |  |  |  |  |
| 3092 | 6627-25 | LIPR1 | Inactive pancreatic lipase-related protein 1 | P54315 | PNLIPRP1 |  |  |  |  |
| 3093 | 12529-32 | FKBP6 | Inactive peptidyl-prolyl cis-trans isomerase FKBP6 | O75344 | FKBP6 | X |  |  |  |
| 3094 | 8081-55 | PLD5 | Inactive phospholipase D5 | Q8N7P1 | PLD5 |  |  |  |  |
| 3095 | 5602-62 | RNS10 | Inactive ribonuclease-like protein 10 | Q5GAN6 | RNASE10 |  |  |  |  |
| 3096 | 9983-97 | PRS35 | Inactive serine protease 35 | Q8N3Z0 | PRSS35 |  |  |  |  |
| 3097 | 9525-1 | PTK7 | Inactive tyrosine-protein kinase 7 | Q13308 | PTK7 |  |  |  |  |
| 3098 | 2590-69 | ROR1 | Inactive tyrosine-protein kinase transmembrane receptor ROR1 | Q01973 | ROR1 |  |  |  | X |
| 3099 | 8474-6 | ROR1 | Inactive tyrosine-protein kinase transmembrane receptor ROR1 | Q01973 | ROR1 |  |  |  |  |
| 3100 | 19606-28 | ihh | Indian hedgehog protein | Q14623 | IHH |  |  |  |  |
| 3101 | 9759-13 | INDO | Indoleamine 2,3-dioxygenase 1 | P14902 | IDO1 |  |  | X | X |
| 3102 | 10396-6 | Mcl-1 | Induced myeloid leukemia cell differentiation protein Mcl-1 | Q07820 | MCL1 | X | X |  | X |
| 3103 | 9819-110 | Mcl-1 | Induced myeloid leukemia cell differentiation protein Mcl-1 | Q07820 | MCL1 |  |  |  |  |
| 3104 | 21899-36 | inhibin A | inhibin A | P05111|P08476 | INHA|INHBA |  |  |  |  |
| 3105 | 25921-3 | Inhibin a subunit | Inhibin a subunit | P05111 | INHA |  |  |  |  |
| 3106 | 13738-8 | Inhibin bA chain | Inhibin beta A chain | P08476 | INHBA |  |  |  |  |
| 3107 | 4383-97 | Activin AB | Inhibin beta A chain:Inhibin beta B chain heterodimer | P08476|P09529 | INHBA|INHBB |  |  |  |  |
| 3108 | 8467-9 | Activin AB | Inhibin beta A chain:Inhibin beta B chain heterodimer | P08476|P09529 | INHBA|INHBB |  |  |  |  |
| 3109 | 18814-21 | Activin AC | Inhibin beta A chain:Inhibin beta C chain heterodimer | P08476|P55103 | INHBA|INHBC |  |  |  |  |
| 3110 | 13676-46 | Inhibin bB chain | Inhibin beta B chain | P09529 | INHBB |  |  |  |  |
| 3111 | 15686-49 | INHBC | Inhibin beta C chain | P55103 | INHBC |  |  |  |  |
| 3112 | 6408-2 | INHBC | Inhibin beta C chain | P55103 | INHBC |  |  |  |  |
| 3113 | 3888-8 | ING1 | Inhibitor of growth protein 1 | Q9UK53 | ING1 |  |  |  | X |
| 3114 | 17671-58 | ING4 | Inhibitor of growth protein 4 | Q9UNL4 | ING4 |  |  |  |  |
| 3115 | 24940-3 | IKK-beta | Inhibitor of nuclear factor kappa B kinase beta subunit | O14920 | IKBKB | X | X | X |  |
| 3116 | 5021-13 | PPase | Inorganic pyrophosphatase | Q15181 | PPA1 |  |  |  |  |
| 3117 | 18307-71 | PPase 2 | Inorganic pyrophosphatase 2, mitochondrial | Q9H2U2 | PPA2 |  |  |  |  |
| 3118 | 18916-25 | Inosine triphosphatase | Inosine triphosphate pyrophosphatase | Q9BY32 | ITPA |  |  | X |  |
| 3119 | 5229-90 | IMDH1 | Inosine-5'-monophosphate dehydrogenase 1 | P20839 | IMPDH1 |  |  |  |  |
| 3120 | 5250-53 | IMDH2 | Inosine-5'-monophosphate dehydrogenase 2 | P12268 | IMPDH2 |  |  |  |  |
| 3121 | 9221-6 | IPIL1 | Inositol 1,4,5-trisphosphate receptor-interacting protein-like 1 | Q6GPH6 | ITPRIPL1 |  |  |  |  |
| 3122 | 22404-4 | IP6K1 | Inositol hexakisphosphate kinase 1 | Q92551 | IP6K1 |  |  |  |  |
| 3123 | 22479-64 | IP6K2 | Inositol hexakisphosphate kinase 2 | Q9UHH9 | IP6K2 |  |  |  |  |
| 3124 | 17796-15 | IMPA1 | Inositol monophosphatase 1 | P29218 | IMPA1 |  |  |  |  |
| 3125 | 12581-39 | IMPA2 | Inositol monophosphatase 2 | O14732 | IMPA2 |  |  |  |  |
| 3126 | 9231-23 | IMPA3 | Inositol monophosphatase 3 | Q9NX62 | BPNT2 |  |  |  |  |
| 3127 | 13498-1 | MIOX | Inositol oxygenase | Q9UGB7 | MIOX |  |  |  |  |
| 3128 | 10011-65 | OCRL | Inositol polyphosphate 5-phosphatase OCRL-1 | Q01968 | OCRL |  |  | X |  |
| 3129 | 21130-82 | ITPK1 | Inositol-tetrakisphosphate 1-kinase | Q13572 | ITPK1 |  |  |  |  |
| 3130 | 13473-55 | IP3KA | Inositol-trisphosphate 3-kinase A | P23677 | ITPKA |  |  |  |  |
| 3131 | 12507-16 | IP3KC | Inositol-trisphosphate 3-kinase C | Q96DU7 | ITPKC | X |  |  |  |
| 3132 | 4883-56 | Insulin | Insulin | P01308 | INS | X | X | X | X |
| 3133 | 11549-6 | ISL1 | Insulin gene enhancer protein ISL-1 | P61371 | ISL1 |  |  |  |  |
| 3134 | 6961-14 | IGFL3 | Insulin growth factor-like family member 3 | Q6UXB1 | IGFL3 |  |  |  |  |
| 3135 | 3448-13 | IR | Insulin receptor | P06213 | INSR |  |  | X |  |
| 3136 | 6352-8 | INSRR | Insulin receptor-related protein | P14616 | INSRR |  |  |  |  |

| # | **Custom Panel (X)** | **SOMAmer SeqID** | **Target Name** | **Human Target or Analyte** | **UniProt ID** | **GeneID** | **Cardiovascular Disease** | **Inflammation and Immune**  **Response** | **Metabolic Disease** | **Oncology** |
| --- | --- | --- | --- | --- | --- | --- | --- | --- | --- | --- |

| 3137 | 3197-70 | IDE | Insulin-degrading enzyme | P14735 | IDE |  | | | |
| --- | --- | --- | --- | --- | --- | --- | --- | --- | --- |
| 3138 | 10663-42 | INSI1 | Insulin-induced gene 1 protein | O15503 | INSIG1 |  |  |  | X |
| 3139 | 5723-4 | INSL3 | Insulin-like 3 | P51460 | INSL3 |  |  |  |  |
| 3140 | 4232-19 | IGF-I sR | Insulin-like growth factor 1 receptor | P08069 | IGF1R | X | X |  | X |
| 3141 | 2952-75 | IGF-I | Insulin-like growth factor I | P05019 | IGF1 |  |  |  |  |
| 3142 | 8406-17 | IGF-I | Insulin-like growth factor I | P05019 | IGF1 | X | X | X | X |
| 3143 | 6973-111 | IGF-II:Pro-form | Insulin-like growth factor II:Isoform 3, Pro-form | P01344 | IGF2 | X |  | X | X |
| 3144 | 15295-81 | IGF-II:Mature | Insulin-like growth factor II:Mature | P01344 | IGF2 | X |  |  | X |
| 3145 | 13741-36 | IGFBP-1 | Insulin-like growth factor-binding protein 1 | P08833 | IGFBP1 | X |  |  |  |
| 3146 | 2771-35 | IGFBP-1 | Insulin-like growth factor-binding protein 1 | P08833 | IGFBP1 |  |  |  |  |
| 3147 | 22985-160 | IGFBP-2 | Insulin-like growth factor-binding protein 2 | P18065 | IGFBP2 |  |  |  |  |
| 3148 | 2570-72 | IGFBP-2 | Insulin-like growth factor-binding protein 2 | P18065 | IGFBP2 | X |  | X |  |
| 3149 | 8469-41 | IGFBP-2 | Insulin-like growth factor-binding protein 2 | P18065 | IGFBP2 |  |  |  |  |
| 3150 | 8819-3 | IGFBP-2 | Insulin-like growth factor-binding protein 2 | P18065 | IGFBP2 |  |  |  |  |
| 3151 | 2571-12 | IGFBP-3 | Insulin-like growth factor-binding protein 3 | P17936 | IGFBP3 | X | X | X | X |
| 3152 | 2950-57 | IGFBP-4 | Insulin-like growth factor-binding protein 4 | P22692 | IGFBP4 |  |  |  |  |
| 3153 | 19581-15 | IGFBP-5 | Insulin-like growth factor-binding protein 5 | P24593 | IGFBP5 |  |  |  |  |
| 3154 | 2685-21 | IGFBP-5 | Insulin-like growth factor-binding protein 5 | P24593 | IGFBP5 |  |  |  | X |
| 3155 | 14088-38 | IGFBP-6 | Insulin-like growth factor-binding protein 6 | P24592 | IGFBP6 |  |  |  |  |
| 3156 | 2686-67 | IGFBP-6 | Insulin-like growth factor-binding protein 6 | P24592 | IGFBP6 | X |  | X |  |
| 3157 | 3320-49 | IGFBP-7 | Insulin-like growth factor-binding protein 7 | Q16270 | IGFBP7 | X |  |  | X |
| 3158 | 6605-17 | IGFALS | Insulin-like growth factor-binding protein complex acid labile subunit | P35858 | IGFALS |  |  | X |  |
| 3159 | 7815-49 | IBPL1 | Insulin-like growth factor-binding protein-like 1 | Q8WX77 | IGFBPL1 |  |  |  |  |
| 3160 | 10462-14 | INSL5 | Insulin-like peptide INSL5 | Q9Y5Q6 | INSL5 |  |  |  |  |
| 3161 | 5754-76 | INSL6 | Insulin-like peptide INSL6 | Q9Y581 | INSL6 |  |  |  |  |
| 3162 | 7765-15 | ITM2A | Integral membrane protein 2A | O43736 | ITM2A |  |  |  |  |
| 3163 | 8086-49 | ITM2B | Integral membrane protein 2B | Q9Y287 | ITM2B | X |  |  |  |
| 3164 | 9523-34 | ITM2C:C-term | Integral membrane protein 2C:C-term | Q9NQX7 | ITM2C |  |  |  |  |
| 3165 | 10560-1 | ITM2C:N-term | Integral membrane protein 2C:N-term | Q9NQX7 | ITM2C |  |  |  |  |
| 3166 | 8055-33 | IDD | Integral membrane protein DGCR2/IDD | P98153 | DGCR2 | X |  |  |  |
| 3167 | 11934-9 | INT3 | Integrator complex subunit 3 | Q68E01 | INTS3 |  |  |  |  |
| 3168 | 21698-11 | Integrin a11b1 | Integrin a11b1 | Q9UKX5|P05556 | ITGA11|ITGB1 |  |  |  |  |
| 3169 | 21901-14 | Integrin a3b1 | Integrin a3b1 | P26006|P05556 | ITGA3|ITGB1 | X |  | X |  |
| 3170 | 21909-10 | Integrin a5b1 | Integrin a5b1 | P08648|P05556 | ITGA5|ITGB1 |  |  |  |  |
| 3171 | 21909-2 | Integrin a5b1 | Integrin a5b1 | P08648|P05556 | ITGA5|ITGB1 | X |  |  | X |
| 3172 | 21903-6 | Integrin aLb2 | Integrin alpha L beta 2 | P20701|P05107 | ITGAL|ITGB2 | X | X | X | X |
| 3173 | 20187-10 | Integrin aVb3 | Integrin alpha V beta 3 | P06756|P05106 | ITGAV|ITGB3 | X | X |  |  |
| 3174 | 20189-28 | Integrin aVb6 | Integrin alpha V beta 6 | P06756|P18564 | ITGAV|ITGB6 |  |  |  |  |
| 3175 | 20189-4 | Integrin aVb6 | Integrin alpha V beta 6 | P06756|P18564 | ITGAV|ITGB6 |  |  |  |  |
| 3176 | 20191-13 | Integrin aVb8 | Integrin alpha V beta 8 | P06756|P26012 | ITGAV|ITGB8 | X |  |  | X |
| 3177 | 20181-17 | ITA11 | Integrin alpha-11 | Q9UKX5 | ITGA11 |  |  |  |  |
| 3178 | 15603-20 | Integrin alpha-2 | Integrin alpha-2 | P17301 | ITGA2 | X |  |  |  |
| 3179 | 21905-10 | Integrin alpha-2/ b1 | Integrin alpha-2/b1 | P17301|P05556 | ITGA2|ITGB1 | X |  |  |  |
| 3180 | 6932-42 | ITA5 | Integrin alpha-5 | P08648 | ITGA5 | X |  |  | X |
| 3181 | 21979-12 | Integrin a6 | Integrin alpha-6 | P23229 | ITGA6 |  | X |  |  |
| 3182 | 21979-8 | Integrin a6 | Integrin alpha-6 | P23229 | ITGA6 |  |  |  |  |
| 3183 | 3503-4 | Integrin a1b1 | Integrin alpha-I: beta-1 complex | P56199|P05556 | ITGA1|ITGB1 |  |  | X |  |
| 3184 | 19574-5 | gpIIbIIIa | Integrin alpha-IIb: beta-3 complex | P08514|P05106 | ITGA2B|ITGB3 |  |  |  |  |
| 3185 | 3739-72 | gpIIbIIIa | Integrin alpha-IIb: beta-3 complex | P08514|P05106 | ITGA2B|ITGB3 | X | X |  |  |
| 3186 | 8488-33 | gpIIbIIIa | Integrin alpha-IIb: beta-3 complex | P08514|P05106 | ITGA2B|ITGB3 |  |  |  |  |
| 3187 | 11617-1 | LFA-1 alpha-L chain | Integrin alpha-L | P20701 | ITGAL | X |  |  |  |
| 3188 | 21981-2 | Integrin alpha-M | Integrin alpha-M | P11215 | ITGAM | X | X | X |  |
| 3189 | 20215-45 | Integrin aVb1 | Integrin alpha-V: beta-1 complex | P06756|P05556 | ITGAV|ITGB1 | X |  |  |  |
| 3190 | 4917-62 | Integrin aVb5 | Integrin alpha-V: beta-5 complex | P06756|P18084 | ITGAV|ITGB5 | X | X |  | X |
| 3191 | 21985-61 | ITBP2 | Integrin beta-1-binding protein 2 | Q9UKP3 | ITGB1BP2 |  |  |  |  |
| 3192 | 12750-9 | LFA-1 beta-2 | Integrin beta-2 | P05107 | ITGB2 |  |  |  | X |
| 3193 | 7755-37 | ITB5 | Integrin beta-5 | P18084 | ITGB5 |  | X |  | X |
| 3194 | 7737-76 | ITB6 | Integrin beta-6 | P18564 | ITGB6 |  | X |  |  |
| 3195 | 11205-10 | Integrin beta-7 | Integrin beta-7 | P26010 | ITGB7 |  |  |  |  |
| 3196 | 21523-71 | ILKAP | Integrin-linked kinase-associated serine/threonine phosphatase 2C | Q9H0C8 | ILKAP |  |  |  |  |
| 3197 | 10053-5 | ILK1 | Integrin-linked protein kinase | Q13418 | ILK | X |  |  |  |
| 3198 | 18830-1 | Omentin | Intelectin-1 | Q8WWA0 | ITLN1 | X |  | X |  |
| 3199 | 7955-195 | ITI heavy chain H1 | Inter-alpha-trypsin inhibitor heavy chain H1 | P19827 | ITIH1 |  |  |  |  |
| 3200 | 9326-33 | ITI heavy chain H2 | Inter-alpha-trypsin inhibitor heavy chain H2 | P19823 | ITIH2 |  |  |  |  |
| 3201 | 7145-1 | ITIH3 | Inter-alpha-trypsin inhibitor heavy chain H3 | Q06033 | ITIH3 |  |  |  |  |
| 3202 | 4811-33 | ITI heavy chain H4 | Inter-alpha-trypsin inhibitor heavy chain H4 | Q14624 | ITIH4 |  |  |  | X |
| 3203 | 8233-2 | ITIH5 | Inter-alpha-trypsin inhibitor heavy chain H5 | Q86UX2 | ITIH5 |  |  |  |  |
| 3204 | 4342-10 | sICAM-1 | Intercellular adhesion molecule 1 | P05362 | ICAM1 | X | X | X | X |
| 3205 | 14756-29 | sICAM-2 | Intercellular adhesion molecule 2 | P13598 | ICAM2 | X |  |  |  |
| 3206 | 5486-73 | sICAM-2 | Intercellular adhesion molecule 2 | P13598 | ICAM2 |  |  |  |  |
| 3207 | 2649-77 | sICAM-3 | Intercellular adhesion molecule 3 | P32942 | ICAM3 | X |  | X |  |
| 3208 | 6550-4 | ICAM4 | Intercellular adhesion molecule 4 | Q14773 | ICAM4 |  |  |  |  |
| 3209 | 9461-2 | ICAM4 | Intercellular adhesion molecule 4 | Q14773 | ICAM4 |  |  |  |  |
| 3210 | 5124-62 | sICAM-5 | Intercellular adhesion molecule 5 | Q9UMF0 | ICAM5 |  |  |  | X |
| 3211 | 5124-69 | sICAM-5 | Intercellular adhesion molecule 5 | Q9UMF0 | ICAM5 |  |  |  |  |

| # | **Custom Panel (X)** | **SOMAmer SeqID** | **Target Name** | **Human Target or Analyte** | **UniProt ID** | **GeneID** | **Cardiovascular Disease** | **Inflammation and Immune**  **Response** | **Metabolic Disease** | **Oncology** |
| --- | --- | --- | --- | --- | --- | --- | --- | --- | --- | --- |

| 3212 | 8245-27 | sICAM-5 | Intercellular adhesion molecule 5 | Q9UMF0 | ICAM5 |  |  |  | |
| --- | --- | --- | --- | --- | --- | --- | --- | --- | --- |
| 3213 | 6055-53 | IFN-a/b R1 | Interferon alpha/beta receptor 1 | P17181 | IFNAR1 |  |  |
| 3214 | 9183-7 | IFN-a/b R1 | Interferon alpha/beta receptor 1 | P17181 | IFNAR1 |  | X |
| 3215 | 18389-11 | IFNA1 | Interferon alpha-1/13 | P01562 | IFNA1 |  | X |  | X |
| 3216 | 14128-121 | IFN10 | Interferon alpha-10 | P01566 | IFNA10 |  |  |  |  |
| 3217 | 5733-61 | IFN10 | Interferon alpha-10 | P01566 | IFNA10 |  |  |  |  |
| 3218 | 7180-114 | IFN14 | Interferon alpha-14 | P01570 | IFNA14 |  |  |  |  |
| 3219 | 6421-52 | IFN16 | Interferon alpha-16 | P05015 | IFNA16 |  |  |  |  |
| 3220 | 3497-13 | IFN-aA | Interferon alpha-2 | P01563 | IFNA2 | X | X | X | X |
| 3221 | 15404-3 | IFN21 | Interferon alpha-21 | P01568 | IFNA21 |  |  |  |  |
| 3222 | 15405-23 | IFNA4 | Interferon alpha-4 | P05014 | IFNA4 |  |  |  |  |
| 3223 | 7268-12 | IFNA4 | Interferon alpha-4 | P05014 | IFNA4 |  |  |  |  |
| 3224 | 6210-100 | IFNA5 | Interferon alpha-5 | P01569 | IFNA5 |  |  |  |  |
| 3225 | 5714-88 | IFNA6 | Interferon alpha-6 | P05013 | IFNA6 |  |  |  |  |
| 3226 | 14129-1 | IFNA7 | Interferon alpha-7 | P01567 | IFNA7 |  |  |  |  |
| 3227 | 6214-84 | IFNA8 | Interferon alpha-8 | P32881 | IFNA8 |  |  |  |  |
| 3228 | 14127-240 | IFN-b | Interferon beta | P01574 | IFNB1 |  | X |  | X |
| 3229 | 7243-8 | IFN-b | Interferon beta | P01574 | IFNB1 |  |  |  |  |
| 3230 | 24027-32 | IFNE1 | Interferon epsilon-1 | Q86WN2 | IFNE |  |  |  |  |
| 3231 | 15346-31 | IFN-g | Interferon gamma | P01579 | IFNG | X | X | X | X |
| 3232 | 2989-17 | IFN-g | Interferon gamma | P01579 | IFNG |  |  |  |  |
| 3233 | 5825-49 | IFN-g R1 | Interferon gamma receptor 1 | P15260 | IFNGR1 | X | X |  | X |
| 3234 | 8818-13 | INGR2:CD | Interferon gamma receptor 2:Cytoplasmic domain | P38484 | IFNGR2 |  |  |  |  |
| 3235 | 9180-6 | INGR2:ECD | Interferon gamma receptor 2:Extracellular domain | P38484 | IFNGR2 |  |  |  |  |
| 3236 | 9305-89 | INGR2:ECD | Interferon gamma receptor 2:Extracellular domain | P38484 | IFNGR2 |  |  |  |  |
| 3237 | 7192-37 | CRF2-12 | Interferon lambda receptor 1 | Q8IU57 | IFNLR1 |  | X |  |  |
| 3238 | 13734-22 | IFN-lambda 1 | Interferon lambda-1 | Q8IU54 | IFNL1 |  |  |  |  |
| 3239 | 4396-54 | IFN-lambda 1 | Interferon lambda-1 | Q8IU54 | IFNL1 |  |  |  |  |
| 3240 | 4397-26 | IFN-lambda 2 | Interferon lambda-2 | Q8IZJ0 | IFNL2 |  |  |  |  |
| 3241 | 5713-9 | IFN-lambda 3 | Interferon lambda-3 | Q8IZI9 | IFNL3 |  | X |  |  |
| 3242 | 21895-36 | IFNL4 | Interferon lambda-4 | K9M1U5 | None |  |  |  |  |
| 3243 | 7196-21 | IFN-w | Interferon omega-1 | P05000 | IFNW1 |  |  |  |  |
| 3244 | 10351-51 | IRF1 | Interferon regulatory factor 1 | P10914 | IRF1 | X |  |  | X |
| 3245 | 17462-19 | IRF1 | Interferon regulatory factor 1 | P10914 | IRF1 |  |  |  |  |
| 3246 | 12801-33 | IRF2 | Interferon regulatory factor 2 | P14316 | IRF2 |  |  |  |  |
| 3247 | 17151-84 | IRF-3 | Interferon regulatory factor 3 | Q14653 | IRF3 |  | X |  |  |
| 3248 | 19564-61 | IRF4 | Interferon regulatory factor 4 | Q15306 | IRF4 | X | X |  | X |
| 3249 | 9857-38 | IRF4 | Interferon regulatory factor 4 | Q15306 | IRF4 |  |  |  |  |
| 3250 | 25460-36 | IRF5 | Interferon regulatory factor 5 | Q13568 | IRF5 | X | X | X |  |
| 3251 | 9999-1 | IRF6 | Interferon regulatory factor 6 | O14896 | IRF6 |  | X |  |  |
| 3252 | 22065-32 | IRF8 | Interferon regulatory factor 8 | Q02556 | IRF8 |  | X |  |  |
| 3253 | 12439-67 | ISGF3 | Interferon regulatory factor 9 | Q00978 | IRF9 |  |  |  |  |
| 3254 | 17460-51 | Mx1 | Interferon-induced GTP-binding protein Mx1 | P20591 | MX1 | X | X | X | X |
| 3255 | 20195-13 | IFIH1 | Interferon-induced helicase C domain-containing protein 1 | Q9BYX4 | IFIH1 | X | X | X |  |
| 3256 | 9853-3 | IFIT2 | Interferon-induced protein with tetratricopeptide repeats 2 | P09913 | IFIT2 |  | X |  |  |
| 3257 | 13642-90 | CIG49 | Interferon-induced protein with tetratricopeptide repeats 3 | O14879 | IFIT3 |  | X |  |  |
| 3258 | 22010-36 | PRKRA | Interferon-inducible double-stranded RNA-dependent protein kinase activator A | O75569 | PRKRA |  |  |  |  |
| 3259 | 25428-103 | IFRD1 | Interferon-related developmental regulator 1 | O00458 | IFRD1 |  |  |  |  |
| 3260 | 12665-16 | ILF2 | Interleukin enhancer-binding factor 2 | Q12905 | ILF2 |  | X |  |  |
| 3261 | 12759-47 | DRBP76 | Interleukin enhancer-binding factor 3 | Q12906 | ILF3 |  |  |  |  |
| 3262 | 4851-25 | IL-1a | Interleukin-1 alpha | P01583 | IL1A | X | X | X | X |
| 3263 | 3037-62 | IL-1b | Interleukin-1 beta | P01584 | IL1B | X | X | X | X |
| 3264 | 17356-34 | IL1FA | Interleukin-1 family member 10 | Q8WWZ1 | IL1F10 |  |  |  |  |
| 3265 | 14048-7 | IL-1 R AcP | Interleukin-1 Receptor accessory protein | Q9NPH3 | IL1RAP |  |  |  |  |
| 3266 | 2630-12 | IL-1 R AcP | Interleukin-1 Receptor accessory protein | Q9NPH3 | IL1RAP |  |  |  |  |
| 3267 | 20522-2 | IRPL1 | Interleukin-1 receptor accessory protein-like 1 | Q9NZN1 | IL1RAPL1 |  |  |  |  |
| 3268 | 5353-89 | IL-1Ra | Interleukin-1 receptor antagonist protein | P18510 | IL1RN | X | X | X | X |
| 3269 | 2991-9 | IL-1 sRI | Interleukin-1 receptor type 1 | P14778 | IL1R1 | X | X |  |  |
| 3270 | 14133-93 | IL-1 sRII | Interleukin-1 receptor type 2 | P27930 | IL1R2 |  |  |  |  |
| 3271 | 5666-64 | IL-1 sRII | Interleukin-1 receptor type 2 | P27930 | IL1R2 | X | X |  |  |
| 3272 | 18162-167 | IRAK4 | Interleukin-1 receptor-associated kinase 4 | Q9NWZ3 | IRAK4 | X | X |  |  |
| 3273 | 4234-8 | IL-1 R4 | Interleukin-1 receptor-like 1 | Q01638 | IL1RL1 | X | X |  |  |
| 3274 | 2994-71 | IL-1Rrp2 | Interleukin-1 receptor-like 2 | Q9HB29 | IL1RL2 |  |  |  |  |
| 3275 | 13723-6 | IL-10 | Interleukin-10 | P22301 | IL10 |  |  |  |  |
| 3276 | 2773-50 | IL-10 | Interleukin-10 | P22301 | IL10 | X | X | X | X |
| 3277 | 10344-334 | IL-10 Ra | Interleukin-10 receptor subunit alpha | Q13651 | IL10RA |  |  |  |  |
| 3278 | 9768-5 | IL-10 Ra:CD | Interleukin-10 receptor subunit alpha:Cytoplasmic domain | Q13651 | IL10RA |  | X |  |  |
| 3279 | 8104-21 | IL-10 Ra:ECD | Interleukin-10 receptor subunit alpha:Extracellular domain | Q13651 | IL10RA |  | X |  |  |
| 3280 | 2631-50 | IL-10 Rb | Interleukin-10 receptor subunit beta | Q08334 | IL10RB | X | X |  |  |
| 3281 | 4493-92 | IL-11 | Interleukin-11 | P20809 | IL11 | X |  |  |  |
| 3282 | 18216-22 | IL-11 RA | Interleukin-11 receptor subunit alpha | Q14626 | IL11RA |  |  |  |  |
| 3283 | 3814-63 | IL-11 RA | Interleukin-11 receptor subunit alpha | Q14626 | IL11RA |  |  |  |  |
| 3284 | 10367-62 | IL-12 | Interleukin-12 | P29459|P29460 | IL12A|IL12B | X | X |  | X |
| 3285 | 13706-12 | IL-12 Rb1 | Interleukin-12 receptor subunit beta-1 | P42701 | IL12RB1 |  |  |  |  |
| 3286 | 2632-5 | IL-12 Rb1 | Interleukin-12 receptor subunit beta-1 | P42701 | IL12RB1 | X | X |  |  |

| # | **Custom Panel (X)** | **SOMAmer SeqID** | **Target Name** | **Human Target or Analyte** | **UniProt ID** | **GeneID** | **Cardiovascular Disease** | **Inflammation and Immune**  **Response** | **Metabolic Disease** | **Oncology** |
| --- | --- | --- | --- | --- | --- | --- | --- | --- | --- | --- |

| 3287 | 3815-14 | IL-12 RB2 | Interleukin-12 receptor subunit beta-2 | Q99665 | IL12RB2 |  | | | |
| --- | --- | --- | --- | --- | --- | --- | --- | --- | --- |
| 3288 | 13733-5 | IL-12 p40 | Interleukin-12 subunit beta | P29460 | IL12B | X | X |  | X |
| 3289 | 14085-28 | IL-13 | Interleukin-13 | P35225 | IL13 |  | X |  | X |
| 3290 | 3072-4 | IL-13 | Interleukin-13 | P35225 | IL13 |  |  |  |  |
| 3291 | 2633-52 | IL-13 Ra1 | Interleukin-13 receptor subunit alpha-1 | P78552 | IL13RA1 |  |  | X |  |
| 3292 | 19568-17 | IL-15 | Interleukin-15 | P40933 | IL15 |  | X | X |  |
| 3293 | 14054-17 | IL-15 Ra | Interleukin-15 receptor subunit alpha | Q13261 | IL15RA |  |  |  |  |
| 3294 | 3445-53 | IL-15 Ra | Interleukin-15 receptor subunit alpha | Q13261 | IL15RA |  |  |  |  |
| 3295 | 2774-10 | IL-16 | Interleukin-16 | Q14005 | IL16 | X | X |  |  |
| 3296 | 2992-59 | IL-17 sR | Interleukin-17 receptor A | Q96F46 | IL17RA | X | X |  |  |
| 3297 | 5084-154 | IL-17B R | Interleukin-17 receptor B | Q9NRM6 | IL17RB |  |  |  |  |
| 3298 | 6262-14 | IL-17B R | Interleukin-17 receptor B | Q9NRM6 | IL17RB |  |  |  |  |
| 3299 | 5468-67 | IL-17 RC | Interleukin-17 receptor C | Q8NAC3 | IL17RC | X | X |  |  |
| 3300 | 3376-49 | IL-17 RD | Interleukin-17 receptor D | Q8NFM7 | IL17RD |  |  |  |  |
| 3301 | 20535-68 | I17RE | Interleukin-17 receptor E | Q8NFR9 | IL17RE |  |  |  |  |
| 3302 | 3498-53 | IL-17 | Interleukin-17A | Q16552 | IL17A | X | X |  |  |
| 3303 | 9170-24 | IL-17 | Interleukin-17A | Q16552 | IL17A |  |  |  |  |
| 3304 | 14022-17 | IL-17B | Interleukin-17B | Q9UHF5 | IL17B |  |  |  |  |
| 3305 | 3499-77 | IL-17B | Interleukin-17B | Q9UHF5 | IL17B |  |  |  |  |
| 3306 | 9255-5 | IL-17C | Interleukin-17C | Q9P0M4 | IL17C |  | X |  |  |
| 3307 | 4136-40 | IL-17D | Interleukin-17D | Q8TAD2 | IL17D |  |  |  |  |
| 3308 | 14026-24 | IL-17F | Interleukin-17F | Q96PD4 | IL17F |  | X |  | X |
| 3309 | 2775-54 | IL-17F | Interleukin-17F | Q96PD4 | IL17F |  |  |  |  |
| 3310 | 5661-15 | IL-18 | Interleukin-18 | Q14116 | IL18 | X | X | X | X |
| 3311 | 14079-14 | IL-18 Ra | Interleukin-18 receptor 1 | Q13478 | IL18R1 |  | X |  |  |
| 3312 | 3446-7 | IL-18 Ra | Interleukin-18 receptor 1 | Q13478 | IL18R1 |  |  |  |  |
| 3313 | 10457-3 | IL-18 Rb | Interleukin-18 receptor accessory protein | O95256 | IL18RAP |  |  |  |  |
| 3314 | 2993-1 | IL-18 Rb | Interleukin-18 receptor accessory protein | O95256 | IL18RAP |  |  |  |  |
| 3315 | 3073-51 | IL-18 BPa | Interleukin-18-binding protein | O95998 | IL18BP | X |  |  |  |
| 3316 | 3035-80 | IL-19 | Interleukin-19 | Q9UHD0 | IL19 |  | X | X |  |
| 3317 | 3070-1 | IL-2 | Interleukin-2 | P60568 | IL2 | X | X |  | X |
| 3318 | 3151-6 | IL-2 sRa | Interleukin-2 receptor subunit alpha | P01589 | IL2RA | X | X | X |  |
| 3319 | 9343-16 | IL-2 sRb | Interleukin-2 receptor subunit beta | P14784 | IL2RB |  | X |  |  |
| 3320 | 4138-25 | IL-20 | Interleukin-20 | Q9NYY1 | IL20 |  | X | X |  |
| 3321 | 5085-18 | IL-20 Ra | Interleukin-20 receptor subunit alpha | Q9UHF4 | IL20RA |  | X |  |  |
| 3322 | 13435-31 | IL-20 Rb | Interleukin-20 receptor subunit beta | Q6UXL0 | IL20RB |  |  |  |  |
| 3323 | 7124-18 | IL-21 | Interleukin-21 | Q9HBE4 | IL21 |  | X | X |  |
| 3324 | 9366-54 | IL-21 sR | Interleukin-21 receptor | Q9HBE5 | IL21R |  | X | X |  |
| 3325 | 13742-66 | IL-22 | Interleukin-22 | Q9GZX6 | IL22 |  |  |  |  |
| 3326 | 2778-10 | IL-22 | Interleukin-22 | Q9GZX6 | IL22 | X | X |  |  |
| 3327 | 3620-67 | IL22RA1 | Interleukin-22 receptor subunit alpha-1 | Q8N6P7 | IL22RA1 |  |  |  |  |
| 3328 | 9603-9 | IL22RA1 | Interleukin-22 receptor subunit alpha-1 | Q8N6P7 | IL22RA1 |  |  |  |  |
| 3329 | 5087-5 | IL-22BP | Interleukin-22 receptor subunit alpha-2 | Q969J5 | IL22RA2 |  |  |  |  |
| 3330 | 9456-34 | IL-22BP | Interleukin-22 receptor subunit alpha-2 | Q969J5 | IL22RA2 |  |  |  |  |
| 3331 | 10365-132 | IL-23 | Interleukin-23 | P29460|Q9NPF7 | IL12B|IL23A | X | X |  | X |
| 3332 | 5088-175 | IL-23 R | Interleukin-23 receptor | Q5VWK5 | IL23R | X | X |  |  |
| 3333 | 3321-2 | IL24 | Interleukin-24 | Q13007 | IL24 |  | X |  | X |
| 3334 | 4137-57 | IL-17E | Interleukin-25 | Q9H293 | IL25 |  |  |  |  |
| 3335 | 16760-2 | IL-26 | Interleukin-26 | Q9NPH9 | IL26 |  |  |  |  |
| 3336 | 2829-19 | IL-27 | Interleukin-27 | Q8NEV9|Q14213 | IL27|EBI3 | X | X | X | X |
| 3337 | 5132-71 | TCCR | Interleukin-27 receptor subunit alpha | Q6UWB1 | IL27RA |  |  |  |  |
| 3338 | 10851-77 | IL27B | Interleukin-27 subunit beta | Q14213 | EBI3 |  |  |  |  |
| 3339 | 4717-55 | IL-3 | Interleukin-3 | P08700 | IL3 |  |  |  |  |
| 3340 | 13744-37 | IL-3 Ra | Interleukin-3 receptor subunit alpha | P26951 | IL3RA |  |  |  |  |
| 3341 | 10455-196 | IL-31 | Interleukin-31 | Q6EBC2 | IL31 |  | X |  |  |
| 3342 | 8273-84 | IL31R | Interleukin-31 receptor subunit alpha | Q8NI17 | IL31RA |  |  | X |  |
| 3343 | 9051-13 | IL32 | Interleukin-32 | P24001 | IL32 |  | X |  | X |
| 3344 | 4556-10 | IL-34 | Interleukin-34 | Q6ZMJ4 | IL34 |  |  |  |  |
| 3345 | 20533-39 | IL-35 | Interleukin-35 | P29459|Q14213 | IL12A|EBI3 | X | X |  | X |
| 3346 | 14150-7 | IL-1F6 | Interleukin-36 alpha | Q9UHA7 | IL36A |  |  |  |  |
| 3347 | 14149-9 | IL-1F8 | Interleukin-36 beta | Q9NZH7 | IL36B |  |  |  |  |
| 3348 | 9117-4 | IL-1F9 | Interleukin-36 gamma | Q9NZH8 | IL36G |  |  |  |  |
| 3349 | 18375-28 | IL-1F5 | Interleukin-36 receptor antagonist protein | Q9UBH0 | IL36RN |  |  |  |  |
| 3350 | 2723-9 | IL-1F7 | Interleukin-37 | Q9NZH6 | IL37 |  |  |  |  |
| 3351 | 13663-2 | IL-4 | Interleukin-4 | P05112 | IL4 |  |  |  |  |
| 3352 | 2906-55 | IL-4 | Interleukin-4 | P05112 | IL4 | X | X |  |  |
| 3353 | 3055-54 | IL-4 sR | Interleukin-4 receptor subunit alpha | P24394 | IL4R | X | X |  | X |
| 3354 | 11071-1 | IL-5 | Interleukin-5 | P05113 | IL5 |  |  |  |  |
| 3355 | 3741-4 | IL-5 | Interleukin-5 | P05113 | IL5 |  | X |  |  |
| 3356 | 13686-2 | IL-5 Ra | Interleukin-5 receptor subunit alpha | Q01344 | IL5RA |  | X |  |  |
| 3357 | 4491-4 | IL-5 Ra | Interleukin-5 receptor subunit alpha | Q01344 | IL5RA |  |  |  |  |
| 3358 | 2573-20 | IL-6 | Interleukin-6 | P05231 | IL6 |  |  |  |  |
| 3359 | 4673-13 | IL-6 | Interleukin-6 | P05231 | IL6 | X | X | X | X |
| 3360 | 15602-43 | IL-6 sRa | Interleukin-6 receptor subunit alpha | P08887 | IL6R |  |  |  |  |
| 3361 | 4139-71 | IL-6 sRa | Interleukin-6 receptor subunit alpha | P08887 | IL6R | X | X | X | X |

| # | **Custom Panel (X)** | **SOMAmer SeqID** | **Target Name** | **Human Target or Analyte** | **UniProt ID** | **GeneID** | **Cardiovascular Disease** | **Inflammation and Immune**  **Response** | **Metabolic Disease** | **Oncology** |
| --- | --- | --- | --- | --- | --- | --- | --- | --- | --- | --- |

| 3362 | 8092-29 | IL-6 sRa | Interleukin-6 receptor subunit alpha | P08887 | IL6R |  | | | |
| --- | --- | --- | --- | --- | --- | --- | --- | --- | --- |
| 3363 | 2620-4 | gp130, soluble | Interleukin-6 receptor subunit beta | P40189 | IL6ST | X | X |  | X |
| 3364 | 14049-17 | IL-7 | Interleukin-7 | P13232 | IL7 |  |  |  |  |
| 3365 | 4140-3 | IL-7 | Interleukin-7 | P13232 | IL7 |  | X |  |  |
| 3366 | 5089-11 | IL-7 Ra | Interleukin-7 receptor subunit alpha | P16871 | IL7R | X | X | X |  |
| 3367 | 3447-64 | IL-8 | Interleukin-8 | P10145 | CXCL8 | X | X | X | X |
| 3368 | 5834-18 | IL-9 | Interleukin-9 | P15248 | IL9 |  | X |  |  |
| 3369 | 14070-56 | ITSN1 | Intersectin-1 | Q15811 | ITSN1 |  |  |  |  |
| 3370 | 4924-32 | MMP-1 | Interstitial collagenase | P03956 | MMP1 | X | X |  | X |
| 3371 | 10463-23 | Alkaline phosphatase, intestine | Intestinal-type alkaline phosphatase | P09923 | ALPI |  |  |  |  |
| 3372 | 17441-4 | Alkaline phosphatase, intestine | Intestinal-type alkaline phosphatase | P09923 | ALPI |  |  |  |  |
| 3373 | 10815-2 | HABP4 | Intracellular hyaluronan-binding protein 4 | Q5JVS0 | HABP4 |  |  |  |  |
| 3374 | 20460-22 | IFT20 | Intraflagellar transport protein 20 homolog | Q8IY31 | IFT20 |  |  |  |  |
| 3375 | 20451-126 | IFT22 | Intraflagellar transport protein 22 homolog | Q9H7X7 | IFT22 |  |  |  |  |
| 3376 | 22481-15 | IQCD | IQ domain-containing protein D | Q96DY2 | IQCD |  |  |  |  |
| 3377 | 7991-54 | IQCF1 | IQ domain-containing protein F1 | Q8N6M8 | IQCF1 |  |  |  |  |
| 3378 | 13439-6 | IQCF3 | IQ domain-containing protein F3 | P0C7M6 | IQCF3 |  |  |  |  |
| 3379 | 8011-96 | PAPL | Iron/zinc purple acid phosphatase-like protein | Q6ZNF0 | ACP7 |  |  |  |  |
| 3380 | 7201-5 | ISCU | Iron-sulfur cluster assembly enzyme ISCU, mitochondrial | Q9H1K1 | ISCU |  |  | X |  |
| 3381 | 19316-2 | HSC20 | Iron-sulfur cluster co-chaperone protein HscB, mitochondrial | Q8IWL3 | HSCB |  |  |  |  |
| 3382 | 19639-53 | IAPP | Islet amyloid polypeptide | P10997 | IAPP |  |  | X |  |
| 3383 | 21975-22 | ICA69 | Islet cell autoantigen 1 | Q05084 | ICA1 |  |  |  |  |
| 3384 | 18313-4 | Asparaginase-like protein 1 | Isoaspartyl peptidase/L-asparaginase | Q7L266 | ASRGL1 |  | X |  |  |
| 3385 | 11406-82 | ACAD8 | Isobutyryl-CoA dehydrogenase, mitochondrial | Q9UKU7 | ACAD8 |  |  | X |  |
| 3386 | 9816-37 | ISOC1 | Isochorismatase domain-containing protein 1 | Q96CN7 | ISOC1 |  |  |  |  |
| 3387 | 19275-68 | IDH3G | Isocitrate dehydrogenase [NAD] subunit gamma, mitochondrial | P51553 | IDH3G |  |  |  |  |
| 3388 | 18338-26 | IDH | Isocitrate dehydrogenase [NADP] cytoplasmic | O75874 | IDH1 |  | X |  | X |
| 3389 | 19437-61 | L-VEGF165 | Isoform L-VEGF165 | P15692 | VEGFA |  |  |  |  |
| 3390 | 12815-9 | SYIC | Isoleucine--tRNA ligase, cytoplasmic | P41252 | IARS1 |  |  |  |  |
| 3391 | 17712-7 | IDI1 | Isopentenyl-diphosphate Delta-isomerase 1 | Q13907 | IDI1 |  |  |  |  |
| 3392 | 17832-12 | IDI2 | Isopentenyl-diphosphate delta-isomerase 2 | Q9BXS1 | IDI2 |  |  |  |  |
| 3393 | 17737-7 | IVD | Isovaleryl-CoA dehydrogenase, mitochondrial | P26440 | IVD |  |  | X |  |
| 3394 | 12434-25 | K0174 | IST1 homolog | P53990 | IST1 |  |  |  |  |
| 3395 | 8355-80 | ISM1 | Isthmin-1 | B1AKI9 | None |  |  |  |  |
| 3396 | 21526-88 | sperm-egg fusion protein 1 | Izumo sperm-egg fusion protein 1 | Q8IYV9 | IZUMO1 |  |  |  |  |
| 3397 | 20549-1 | IZUM4 | Izumo sperm-egg fusion protein 4 | Q1ZYL8 | IZUMO4 |  |  |  |  |
| 3398 | 9068-17 | JKIP3 | Janus kinase and microtubule-interacting protein 3 | Q5VZ66 | JAKMIP3 |  |  |  |  |
| 3399 | 20449-72 | JOS1 | Josephin-1 | Q15040 | JOSD1 |  |  |  |  |
| 3400 | 21311-22 | JDP2 | Jun dimerization protein 2 | Q8WYK2 | JDP2 |  |  |  |  |
| 3401 | 16768-3 | PLAK | Junction plakoglobin | P14923 | JUP | X |  |  |  |
| 3402 | 23007-8 | PLAK | Junction plakoglobin | P14923 | JUP |  |  |  |  |
| 3403 | 20511-3 | JAM-A | Junctional adhesion molecule A | Q9Y624 | F11R |  |  |  |  |
| 3404 | 2997-8 | JAM-B | Junctional adhesion molecule B | P57087 | JAM2 |  |  |  |  |
| 3405 | 2998-53 | JAM-C | Junctional adhesion molecule C | Q9BX67 | JAM3 |  |  |  |  |
| 3406 | 5094-62 | JAML1 | Junctional adhesion molecule-like | Q86YT9 | JAML |  |  |  |  |
| 3407 | 8232-90 | JAML1 | Junctional adhesion molecule-like | Q86YT9 | JAML |  |  |  |  |
| 3408 | 6940-18 | JPH1 | Junctophilin-1 | Q9HDC5 | JPH1 |  |  |  |  |
| 3409 | 9089-77 | JPH3 | Junctophilin-3 | Q8WXH2 | JPH3 |  |  |  |  |
| 3410 | 9111-40 | JPH4 | Junctophilin-4 | Q96JJ6 | JPH4 |  |  |  |  |
| 3411 | 21444-40 | Kallikrein 2 | Kallikrein 2 | P20151 | KLK2 |  |  |  |  |
| 3412 | 6227-1 | kallikrein 10 | Kallikrein-10 | O43240 | KLK10 |  |  |  | X |
| 3413 | 2831-29 | Kallikrein 11 | Kallikrein-11 | Q9UBX7 | KLK11 |  |  |  |  |
| 3414 | 7775-15 | Kallikrein 11 | Kallikrein-11 | Q9UBX7 | KLK11 |  |  |  |  |
| 3415 | 3199-54 | kallikrein 12 | Kallikrein-12 | Q9UKR0 | KLK12 |  |  |  |  |
| 3416 | 11152-46 | kallikrein 13 | Kallikrein-13 | Q9UKR3 | KLK13 |  |  |  |  |
| 3417 | 3200-49 | kallikrein 13 | Kallikrein-13 | Q9UKR3 | KLK13 |  |  |  |  |
| 3418 | 15544-25 | kallikrein 14 | Kallikrein-14 | Q9P0G3 | KLK14 |  |  |  |  |
| 3419 | 3681-87 | kallikrein 14 | Kallikrein-14 | Q9P0G3 | KLK14 |  |  |  |  |
| 3420 | 8620-56 | kallikrein 14 | Kallikrein-14 | Q9P0G3 | KLK14 |  |  |  |  |
| 3421 | 6491-59 | kallikrein 15 | Kallikrein-15 | Q9H2R5 | KLK15 |  |  |  |  |
| 3422 | 2833-20 | Kallikrein 4 | Kallikrein-4 | Q9Y5K2 | KLK4 |  |  |  | X |
| 3423 | 14039-33 | kallikrein 5 | Kallikrein-5 | Q9Y337 | KLK5 |  |  |  |  |
| 3424 | 3201-49 | kallikrein 5 | Kallikrein-5 | Q9Y337 | KLK5 |  |  |  |  |
| 3425 | 3450-4 | Kallikrein 6 | Kallikrein-6 | Q92876 | KLK6 |  |  |  |  |
| 3426 | 3378-49 | Kallikrein 7 | Kallikrein-7 | P49862 | KLK7 |  |  |  |  |
| 3427 | 13708-56 | kallikrein 8 | Kallikrein-8 | O60259 | KLK8 |  |  |  | X |
| 3428 | 2834-54 | kallikrein 8 | Kallikrein-8 | O60259 | KLK8 |  |  |  |  |
| 3429 | 5758-49 | kallikrein 9 | Kallikrein-9 | Q9UKQ9 | KLK9 |  |  |  |  |
| 3430 | 14105-5 | Kallistatin | Kallistatin | P29622 | SERPINA4 |  |  |  |  |
| 3431 | 3449-58 | Kallistatin | Kallistatin | P29622 | SERPINA4 |  |  |  |  |
| 3432 | 24494-67 | KATL1 | Katanin p60 ATPase-containing subunit A-like 1 | Q9BW62 | KATNAL1 |  |  |  |  |
| 3433 | 7144-234 | KAZD1 | Kazal-type serine protease inhibitor domain-containing protein 1 | Q96I82 | KAZALD1 |  |  |  |  |
| 3434 | 6611-8 | KDEL1 | KDEL motif-containing protein 1 | Q6UW63 | POGLUT2 |  |  |  |  |
| 3435 | 8296-117 | KDEL2 | KDEL motif-containing protein 2 | Q7Z4H8 | POGLUT3 |  |  |  |  |
| 3436 | 25285-14 | KBTBB | Kelch repeat and BTB domain-containing protein 11 | O94819 | KBTBD11 |  |  |  |  |

| # | **Custom Panel (X)** | **SOMAmer SeqID** | **Target Name** | **Human Target or Analyte** | **UniProt ID** | **GeneID** | **Cardiovascular Disease** | **Inflammation and Immune**  **Response** | **Metabolic Disease** | **Oncology** |
| --- | --- | --- | --- | --- | --- | --- | --- | --- | --- | --- |

| 3437 | 12568-14 | KEAP1 | Kelch-like ECH-associated protein 1 | Q14145 | KEAP1 |  | | | |
| --- | --- | --- | --- | --- | --- | --- | --- | --- | --- |
| 3438 | 8485-7 | KEAP1 | Kelch-like ECH-associated protein 1 | Q14145 | KEAP1 |  |  |  | X |
| 3439 | 12695-62 | KLH12 | Kelch-like protein 12 | Q53G59 | KLHL12 |  |  |  |  |
| 3440 | 12463-7 | KLH13 | Kelch-like protein 13 | Q9P2N7 | KLHL13 |  |  |  |  |
| 3441 | 24903-7 | KLH14 | Kelch-like protein 14 | Q9P2G3 | KLHL14 |  |  |  |  |
| 3442 | 23649-6 | KLHL2 | Kelch-like protein 2 | O95198 | KLHL2 |  |  |  |  |
| 3443 | 25039-10 | KLHL3 | Kelch-like protein 3 | Q9UH77 | KLHL3 |  |  |  |  |
| 3444 | 24256-7 | KLH40 | Kelch-like protein 40 | Q2TBA0 | KLHL40 |  |  |  |  |
| 3445 | 23660-112 | KLH41 | Kelch-like protein 41 | O60662 | KLHL41 |  |  |  |  |
| 3446 | 12625-138 | KLHL7 | Kelch-like protein 7 | Q8IXQ5 | KLHL7 |  |  |  |  |
| 3447 | 7070-25 | KELL | Kell blood group glycoprotein | P23276 | KEL |  |  |  |  |
| 3448 | 22378-2 | Keratin 34 | Keratin 34 | O76011 | KRT34 |  |  |  |  |
| 3449 | 22482-87 | K1C14 | Keratin, type I cytoskeletal 14 | P02533 | KRT14 |  |  |  | X |
| 3450 | 18905-5 | Keratin-16 | Keratin, type I cytoskeletal 16 | P08779 | KRT16 |  |  |  |  |
| 3451 | 12923-51 | Keratin 17 | Keratin, type I cytoskeletal 17 | Q04695 | KRT17 |  |  |  | X |
| 3452 | 5354-11 | Keratin 18 | Keratin, type I cytoskeletal 18 | P05783 | KRT18 |  |  |  | X |
| 3453 | 15606-19 | Keratin 19 | Keratin, type I cytoskeletal 19 | P08727 | KRT19 |  | X | X |  |
| 3454 | 12975-11 | Keratin 20 | Keratin, type I cytoskeletal 20 | P35900 | KRT20 |  |  |  | X |
| 3455 | 9931-20 | Keratin-1 | Keratin, type II cytoskeletal 1 | P04264 | KRT1 |  |  |  |  |
| 3456 | 11177-16 | K2C5 | Keratin, type II cytoskeletal 5 | P13647 | KRT5 |  |  |  | X |
| 3457 | 22486-58 | Keratin 6A | Keratin, type II cytoskeletal 6A | P02538 | KRT6A |  |  |  |  |
| 3458 | 11383-41 | Keratin 7 | Keratin, type II cytoskeletal 7 | P08729 | KRT7 |  |  | X | X |
| 3459 | 22483-109 | K2C71 | Keratin, type II cytoskeletal 71 | Q3SY84 | KRT71 |  |  |  | X |
| 3460 | 11566-48 | Keratin 72 | Keratin, type II cytoskeletal 72 | Q14CN4 | KRT72 |  |  |  |  |
| 3461 | 14615-46 | KRA24 | Keratin-associated protein 2-4 | Q9BYR9 | KRTAP2-4 |  |  |  |  |
| 3462 | 23550-10 | KDF1 | Keratinocyte differentiation factor 1 | Q8NAX2 | KDF1 |  |  |  |  |
| 3463 | 5739-75 | KTDAP | Keratinocyte differentiation-associated protein | P60985 | KRTDAP |  |  |  |  |
| 3464 | 10758-2 | KERA | Keratocan | O60938 | KERA |  |  |  |  |
| 3465 | 21856-59 | KHK | Ketohexokinase | P50053 | KHK |  |  | X |  |
| 3466 | 23395-5 | KHDR2 | KH domain-containing, RNA-binding, signal transduction-associated protein 2 | Q5VWX1 | KHDRBS2 |  |  |  |  |
| 3467 | 19492-5 | KAAG1 | Kidney-associated antigen 1 | Q9UBP8 | KAAG1 |  |  |  |  |
| 3468 | 12956-40 | KBP | KIF1-binding protein | Q96EK5 | KIFBP |  |  |  |  |
| 3469 | 17200-50 | KI2L1 | Killer cell immunoglobulin-like receptor 2DL1 | P43626 | KIR2DL1 |  |  |  |  |
| 3470 | 7773-20 | KI2L2 | Killer cell immunoglobulin-like receptor 2DL2 | P43627 | KIR2DL2 |  |  |  |  |
| 3471 | 17153-46 | KI2L3 | Killer cell immunoglobulin-like receptor 2DL3 | P43628 | KIR2DL3 |  |  |  |  |
| 3472 | 5095-21 | KI2L4 | Killer cell immunoglobulin-like receptor 2DL4 | Q99706 | KIR2DL4 |  |  |  |  |
| 3473 | 9245-1 | KI2L4 | Killer cell immunoglobulin-like receptor 2DL4 | Q99706 | KIR2DL4 |  |  |  |  |
| 3474 | 7799-3 | KI2LA | Killer cell immunoglobulin-like receptor 2DL5A | Q8N109 | KIR2DL5A |  |  |  |  |
| 3475 | 8000-17 | KI2LA | Killer cell immunoglobulin-like receptor 2DL5A | Q8N109 | KIR2DL5A |  |  |  |  |
| 3476 | 10428-1 | KI2S2 | Killer cell immunoglobulin-like receptor 2DS2 | P43631 | KIR2DS2 |  |  |  |  |
| 3477 | 17152-10 | KI2S4 | Killer cell immunoglobulin-like receptor 2DS4 | P43632 | KIR2DS4 |  |  |  |  |
| 3478 | 18907-97 | KI3L1 | Killer cell immunoglobulin-like receptor 3DL1 | P43629 | KIR3DL1 |  | X |  |  |
| 3479 | 5096-51 | KI3L2 | Killer cell immunoglobulin-like receptor 3DL2 | P43630 | KIR3DL2 |  |  |  |  |
| 3480 | 8045-3 | KI3L3:CD | Killer cell immunoglobulin-like receptor 3DL3:Cytoplasmic domain | Q8N743 | KIR3DL3 |  |  |  |  |
| 3481 | 7944-1 | KI3L3:ECD | Killer cell immunoglobulin-like receptor 3DL3:Extracellular domain | Q8N743 | KIR3DL3 |  |  |  |  |
| 3482 | 5097-14 | KI3S1 | Killer cell immunoglobulin-like receptor 3DS1 | Q14943 | KIR3DS1 |  |  |  |  |
| 3483 | 10809-14 | KLRB1 | Killer cell lectin-like receptor subfamily B member 1 | Q12918 | KLRB1 |  |  |  |  |
| 3484 | 20562-78 | KLRB1 | Killer cell lectin-like receptor subfamily B member 1 | Q12918 | KLRB1 |  |  |  |  |
| 3485 | 5098-79 | KLRF1 | Killer cell lectin-like receptor subfamily F member 1 | Q9NZS2 | KLRF1 |  |  |  |  |
| 3486 | 7756-37 | KLRF1 | Killer cell lectin-like receptor subfamily F member 1 | Q9NZS2 | KLRF1 |  |  |  |  |
| 3487 | 10693-43 | KLRG2:C-term | Killer cell lectin-like receptor subfamily G member 2:C-term | A4D1S0 | KLRG2 |  |  |  |  |
| 3488 | 10762-2 | KLRG2:N-term | Killer cell lectin-like receptor subfamily G member 2:N-term | A4D1S0 | KLRG2 |  |  |  |  |
| 3489 | 22563-4 | KIRR1 | Kin of IRRE-like protein 1 | Q96J84 | KIRREL1 |  |  |  |  |
| 3490 | 16609-106 | KIRR2 | Kin of IRRE-like protein 2 | Q6UWL6 | KIRREL2 |  |  |  |  |
| 3491 | 7958-15 | KIRR2 | Kin of IRRE-like protein 2 | Q6UWL6 | KIRREL2 |  |  |  |  |
| 3492 | 4557-61 | KIRR3 | Kin of IRRE-like protein 3 | Q8IZU9 | KIRREL3 |  |  |  |  |
| 3493 | 12656-1 | KLC1 | Kinesin light chain 1 | Q07866 | KLC1 |  |  |  |  |
| 3494 | 24245-2 | KLC3 | Kinesin light chain 3 | Q6P597 | KLC3 |  |  |  |  |
| 3495 | 11672-17 | KI16B | Kinesin-like protein KIF16B | Q96L93 | KIF16B |  |  |  |  |
| 3496 | 9899-28 | KIF1C | Kinesin-like protein KIF1C | O43896 | KIF1C |  |  |  |  |
| 3497 | 12734-112 | KIF22 | Kinesin-like protein KIF22 | Q14807 | KIF22 |  | X |  |  |
| 3498 | 5228-25 | KIF23 | Kinesin-like protein KIF23 | Q02241 | KIF23 |  |  | X |  |
| 3499 | 13961-18 | KIF3A | Kinesin-like protein KIF3A | Q9Y496 | KIF3A |  | X |  |  |
| 3500 | 24953-27 | KIF3B | Kinesin-like protein KIF3B | O15066 | KIF3B |  |  |  |  |
| 3501 | 24407-31 | KIF3C | Kinesin-like protein KIF3C | O14782 | KIF3C | X |  | X |  |
| 3502 | 12730-3 | KNTC2 | Kinetochore protein NDC80 homolog | O14777 | NDC80 |  |  |  |  |
| 3503 | 22782-80 | SPC25 | Kinetochore protein Spc25 | Q9HBM1 | SPC25 |  |  |  |  |
| 3504 | 22518-54 | NSL1 | Kinetochore-associated protein NSL1 homolog | Q96IY1 | NSL1 |  |  |  |  |
| 3505 | 15343-337 | Kininogen, HMW, Two Chain | Kininogen, HMW, Two Chain | P01042 | KNG1 | X | X |  |  |
| 3506 | 4918-21 | Kininogen, HMW | Kininogen-1 | P01042 | KNG1 |  |  |  |  |
| 3507 | 7784-1 | Kininogen, HMW | Kininogen-1 | P01042 | KNG1 | X | X |  |  |
| 3508 | 19631-13 | Kininostatin | Kininostatin | P01042 | KNG1 |  |  |  |  |
| 3509 | 9377-25 | SCF | Kit ligand | P21583 | KITLG | X | X |  | X |
| 3510 | 23579-3 | KKLC1 | Kita-kyushu lung cancer antigen 1 | Q5H943 | CT83 |  |  |  |  |
| 3511 | 15384-15 | KLOTHO | Klotho | Q9UEF7 | KL | X |  | X |  |

| # | **Custom Panel (X)** | **SOMAmer SeqID** | **Target Name** | **Human Target or Analyte** | **UniProt ID** | **GeneID** | **Cardiovascular Disease** | **Inflammation and Immune**  **Response** | **Metabolic Disease** | **Oncology** |
| --- | --- | --- | --- | --- | --- | --- | --- | --- | --- | --- |

| 3512 | 17331-138 | KREM1 | Kremen protein 1 | Q96MU8 | KREMEN1 |  |  |  | |
| --- | --- | --- | --- | --- | --- | --- | --- | --- | --- |
| 3513 | 6512-68 | KREM1 | Kremen protein 1 | Q96MU8 | KREMEN1 |  |  |
| 3514 | 3202-28 | KREM2 | Kremen protein 2 | Q8NCW0 | KREMEN2 |  |  |
| 3515 | 17850-42 | KLF4 | Krueppel-like factor 4 | O43474 | KLF4 | X | X |
| 3516 | 22488-17 | KLF9 | Krueppel-like factor 9 | Q13886 | KLF9 | X |  |
| 3517 | 2828-82 | HAI-1 | Kunitz-type protease inhibitor 1 | O43278 | SPINT1 |  |  |
| 3518 | 2843-13 | SPINT2 | Kunitz-type protease inhibitor 2 | O43291 | SPINT2 |  |  | X | X |
| 3519 | 7926-13 | SPIT3 | Kunitz-type protease inhibitor 3 | P49223 | SPINT3 |  |  |  |  |
| 3520 | 13650-11 | KCIP1 | Kv channel-interacting protein 1 | Q9NZI2 | KCNIP1 |  |  |  |  |
| 3521 | 7011-8 | KCIP4 | Kv channel-interacting protein 4 | Q6PIL6 | KCNIP4 |  |  |  |  |
| 3522 | 22050-19 | KXDL1 | KxDL motif-containing protein 1 | Q9BQD3 | KXD1 |  |  |  |  |
| 3523 | 4559-64 | KYNU | Kynureninase | Q16719 | KYNU |  |  | X |  |
| 3524 | 25466-84 | KMO | Kynurenine 3-monooxygenase | O15229 | KMO |  | X |  |  |
| 3525 | 22023-3 | AADAT | Kynurenine/alpha-aminoadipate aminotransferase, mitochondrial | Q8N5Z0 | AADAT |  |  |  |  |
| 3526 | 17365-7 | KAT1 | Kynurenine--oxoglutarate transaminase 1 | Q16773 | KYAT1 |  |  |  | X |
| 3527 | 12682-5 | KAT3 | Kynurenine--oxoglutarate transaminase 3 | Q6YP21 | KYAT3 |  |  |  |  |
| 3528 | 23595-6 | LACC1 | Laccase domain-containing protein 1 | Q8IV20 | LACC1 |  | X |  |  |
| 3529 | 4455-89 | MFGM | Lactadherin | Q08431 | MFGE8 |  |  | X | X |
| 3530 | 10890-135 | LCTL | Lactase-like protein | Q6UWM7 | LCTL |  |  |  |  |
| 3531 | 9017-58 | LPH | Lactase-phlorizin hydrolase | P09848 | LCT |  |  | X |  |
| 3532 | 4801-13 | PERL | Lactoperoxidase | P22079 | LPO |  |  |  |  |
| 3533 | 8759-29 | a1,4-Galactosyltransferase | Lactosylceramide 4-alpha-galactosyltransferase | Q9NPC4 | A4GALT |  | X |  |  |
| 3534 | 10588-39 | SIAT9 | Lactosylceramide alpha-2,3-sialyltransferase | Q9UNP4 | ST3GAL5 |  |  |  |  |
| 3535 | 14755-4 | Lactoferrin | Lactotransferrin | P02788 | LTF |  |  |  |  |
| 3536 | 2780-35 | Lactoferrin | Lactotransferrin | P02788 | LTF | X | X | X | X |
| 3537 | 9883-29 | Glyoxalase I | Lactoylglutathione lyase | Q04760 | GLO1 | X |  |  |  |
| 3538 | 6407-63 | LAD1 | Ladinin-1 | O00515 | LAD1 |  |  |  |  |
| 3539 | 24643-21 | CRYL1 | Lambda-crystallin homolog | Q9Y2S2 | CRYL1 |  |  |  |  |
| 3540 | 8265-225 | LAP2B | Lamina-associated polypeptide 2, isoforms beta/gamma | P42167 | TMPO | X | X |  |  |
| 3541 | 3889-64 | Lamin-B1 | Lamin-B1 | P20700 | LMNB1 |  |  | X |  |
| 3542 | 20217-26 | Lamin-B2 | Lamin-B2 | Q03252 | LMNB2 |  |  | X |  |
| 3543 | 20074-3 | LAMA3 | Laminin subunit alpha-3 | Q16787 | LAMA3 |  |  |  |  |
| 3544 | 6577-64 | LAMA4 | Laminin subunit alpha-4 | Q16363 | LAMA4 | X |  |  |  |
| 3545 | 9580-5 | Laminin gamma-2 | Laminin subunit gamma-2 | Q13753 | LAMC2 |  |  |  |  |
| 3546 | 18347-15 | Laminin-2 | Laminin-2 | P24043|P07942|P11047 | LAMA2|LAMB1|LAMC1 | X |  |  | X |
| 3547 | 17320-19 | ADPPT | L-aminoadipate-semialdehyde dehydrogenase-phosphopantetheinyl transferase | Q9NRN7 | AASDHPPT |  |  |  |  |
| 3548 | 13986-6 | LANC1 | LanC-like protein 1 | O43813 | LANCL1 |  |  |  |  |
| 3549 | 12628-31 | LANC2 | LanC-like protein 2 | Q9NS86 | LANCL2 |  |  |  |  |
| 3550 | 21955-36 | BAT3 | Large proline-rich protein BAT3 | P46379 | BAG6 |  |  |  |  |
| 3551 | 25414-11 | LCE3B | Late cornified envelope protein 3B | Q5TA77 | LCE3B |  |  |  |  |
| 3552 | 23334-21 | LCE3C | Late cornified envelope protein 3C | Q5T5A8 | LCE3C |  |  |  |  |
| 3553 | 13133-73 | LTBP4 | Latent-transforming growth factor beta-binding protein 4 | Q8N2S1 | LTBP4 |  |  |  |  |
| 3554 | 20592-8 | LXN | Latexin | Q9BS40 | LXN |  |  |  |  |
| 3555 | 20578-10 | LPHN3 | Latrophilin-3 | Q9HAR2 | ADGRL3 |  |  |  |  |
| 3556 | 2635-61 | Layilin | Layilin | Q6UX15 | LAYN |  |  |  |  |
| 3557 | 15299-102 | MESD2 | LDLR chaperone MESD | Q14696 | MESD |  |  |  |  |
| 3558 | 5594-87 | MESD2 | LDLR chaperone MESD | Q14696 | MESD |  |  |  |  |
| 3559 | 21390-68 | L-dopachrome tautomerase | L-dopachrome tautomerase | P40126 | DCT |  |  |  |  |
| 3560 | 15503-15 | Lefty-A | Left-right determination factor 2 | O00292 | LEFTY2 |  |  |  |  |
| 3561 | 15503-20 | Lefty-A | Left-right determination factor 2 | O00292 | LEFTY2 | X |  |  |  |
| 3562 | 3622-33 | LGMN | Legumain | Q99538 | LGMN |  |  |  |  |
| 3563 | 7840-64 | LGMN | Legumain | Q99538 | LGMN |  |  |  |  |
| 3564 | 12504-26 | LMOD1 | Leiomodin-1 | P29536 | LMOD1 | X |  |  |  |
| 3565 | 8040-9 | LEMD1 | LEM domain-containing protein 1 | Q68G75 | LEMD1 |  |  |  |  |
| 3566 | 24499-36 | LGSN | Lengsin | Q5TDP6 | LGSN |  |  |  |  |
| 3567 | 2575-5 | Leptin | Leptin | P41159 | LEP |  |  |  |  |
| 3568 | 8484-24 | Leptin | Leptin | P41159 | LEP | X | X | X | X |
| 3569 | 5400-52 | sLeptin R | Leptin receptor, soluble | P48357 | LEPR | X | X | X | X |
| 3570 | 12708-91 | LMBL2 | Lethal(3)malignant brain tumor-like protein 2 | Q969R5 | L3MBTL2 |  |  |  | X |
| 3571 | 5751-14 | HCCR-1 | LETM1 domain-containing protein 1 | Q6P1Q0 | LETMD1 |  |  |  |  |
| 3572 | 4237-70 | LCMT1 | Leucine carboxyl methyltransferase 1 | Q9UIC8 | LCMT1 |  |  |  |  |
| 3573 | 24638-3 | LRA25 | Leucine repeat adapter protein 25 | Q8N5H3 | FAM89B |  |  |  |  |
| 3574 | 20378-110 | LZTL1 | Leucine zipper transcription factor-like protein 1 | Q9NQ48 | LZTFL1 | X |  |  |  |
| 3575 | 11252-30 | LRCH4:CH | Leucine-rich repeat and calponin homology domain-containing protein 4:Calponin Homology | O75427 | LRCH4 |  |  |  |  |
| 3576 | 8984-28 | LRCH4: LRR5 | Leucine-rich repeat and calponin homology domain-containing protein 4:Leucine-rich repeat 5 | O75427 | LRCH4 |  |  |  |  |
| 3577 | 7910-41 | LRFN1 | Leucine-rich repeat and fibronectin type III domain-containing protein 1 | Q9P244 | LRFN1 |  |  |  |  |
| 3578 | 7200-4 | LRFN2 | Leucine-rich repeat and fibronectin type-III domain-containing protein 2 | Q9ULH4 | LRFN2 |  |  |  |  |
| 3579 | 21691-27 | LRFN3 | Leucine-rich repeat and fibronectin type-III domain-containing protein 3 | Q9BTN0 | LRFN3 |  |  |  |  |
| 3580 | 21696-80 | LRFN4 | Leucine-rich repeat and fibronectin type-III domain-containing protein 4 | Q6PJG9 | LRFN4 |  |  |  |  |
| 3581 | 6587-6 | LRFN5 | Leucine-rich repeat and fibronectin type-III domain-containing protein 5 | Q96NI6 | LRFN5 |  |  |  |  |
| 3582 | 6620-82 | LIGO1 | Leucine-rich repeat and immunoglobulin-like domain-containing nogo receptor-interacting | Q96FE5 | LINGO1 |  |  |  |  |
| 3583 | 10827-67 | LIGO3 | Leucine-rich repeat and immunoglobulin-like domain-containing nogo receptor-interacting | P0C6S8 | LINGO3 |  |  |  |  |
| 3584 | 9368-64 | LRTM1 | Leucine-rich repeat and transmembrane domain-containing protein 1 | Q9HBL6 | LRTM1 |  |  |  |  |
| 3585 | 8906-60 | LRTM2 | Leucine-rich repeat and transmembrane domain-containing protein 2 | Q8N967 | LRTM2 |  |  |  |  |
| 3586 | 25479-8 | LRRF2 | Leucine-rich repeat flightless-interacting protein 2 | Q9Y608 | LRRFIP2 |  |  |  |  |

| # | **Custom Panel (X)** | **SOMAmer SeqID** | **Target Name** | **Human Target or Analyte** | **UniProt ID** | **GeneID** | **Cardiovascular Disease** | **Inflammation and Immune**  **Response** | **Metabolic Disease** | **Oncology** |
| --- | --- | --- | --- | --- | --- | --- | --- | --- | --- | --- |

| 3587 | 8003-57 | LGI3 | Leucine-rich repeat LGI family member 3 | Q8N145 | LGI3 |  | | | |
| --- | --- | --- | --- | --- | --- | --- | --- | --- | --- |
| 3588 | 11293-14 | LRRN1:CD | Leucine-rich repeat neuronal protein 1:Cytoplasmic domain | Q6UXK5 | LRRN1 |
| 3589 | 11586-2 | LRRN1:ECD | Leucine-rich repeat neuronal protein 1:Extracellular domain | Q6UXK5 | LRRN1 |
| 3590 | 10990-21 | LRRK2 | Leucine-rich repeat serine/threonine-protein kinase 2 | Q5S007 | LRRK2 |  |  |  | X |
| 3591 | 19360-22 | LRRT1 | Leucine-rich repeat transmembrane neuronal protein 1 | Q86UE6 | LRRTM1 |  |  |  |  |
| 3592 | 4452-9 | LRRT1 | Leucine-rich repeat transmembrane neuronal protein 1 | Q86UE6 | LRRTM1 |  |  |  |  |
| 3593 | 6904-14 | LRRT2 | Leucine-rich repeat transmembrane neuronal protein 2 | O43300 | LRRTM2 |  |  |  |  |
| 3594 | 4453-83 | LRRT3 | Leucine-rich repeat transmembrane neuronal protein 3 | Q86VH5 | LRRTM3 |  |  |  |  |
| 3595 | 8646-61 | LRRT4:CD | Leucine-rich repeat transmembrane neuronal protein 4:Isoform 2, Cytoplasmic domain | Q86VH4 | LRRTM4 |  |  |  |  |
| 3596 | 6572-10 | LRRT4:ECD | Leucine-rich repeat transmembrane neuronal protein 4:Isoform 2, Extracellular domain | Q86VH4 | LRRTM4 |  |  |  |  |
| 3597 | 13739-3 | FLRT1 | Leucine-rich repeat transmembrane protein FLRT1 | Q9NZU1 | FLRT1 |  |  |  |  |
| 3598 | 4547-59 | FLRT1 | Leucine-rich repeat transmembrane protein FLRT1 | Q9NZU1 | FLRT1 |  |  |  |  |
| 3599 | 13122-19 | FLRT2 | Leucine-rich repeat transmembrane protein FLRT2 | O43155 | FLRT2 |  |  |  |  |
| 3600 | 9128-34 | FLRT3:CD | Leucine-rich repeat transmembrane protein FLRT3:Cytoplasmic domain | Q9NZU0 | FLRT3 |  |  |  |  |
| 3601 | 13123-3 | FLRT3:ECD | Leucine-rich repeat transmembrane protein FLRT3:Extracellular domain | Q9NZU0 | FLRT3 |  |  |  |  |
| 3602 | 11716-28 | LRIT2 | Leucine-rich repeat, immunoglobulin-like domain and transmembrane domain-containing | A6NDA9 | LRIT2 |  |  |  |  |
| 3603 | 11534-6 | LRIT3 | Leucine-rich repeat, immunoglobulin-like domain and transmembrane domain-containing | Q3SXY7 | LRIT3 |  |  |  |  |
| 3604 | 16304-6 | LGR4 | Leucine-rich repeat-containing G-protein coupled receptor 4 | Q9BXB1 | LGR4 |  |  |  |  |
| 3605 | 16296-43 | LGR5 | Leucine-rich repeat-containing G-protein coupled receptor 5 | O75473 | LGR5 |  |  |  |  |
| 3606 | 7122-31 | LGR5 | Leucine-rich repeat-containing G-protein coupled receptor 5 | O75473 | LGR5 |  |  |  |  |
| 3607 | 6557-50 | LRC15 | Leucine-rich repeat-containing protein 15 | Q8TF66 | LRRC15 |  |  |  |  |
| 3608 | 23288-28 | LRC20 | Leucine-rich repeat-containing protein 20 | Q8TCA0 | LRRC20 |  |  |  |  |
| 3609 | 9989-12 | LRC24 | Leucine-rich repeat-containing protein 24 | Q50LG9 | LRRC24 |  |  |  |  |
| 3610 | 9987-30 | LRC25:CD | Leucine-rich repeat-containing protein 25:Cytoplasmic domain | Q8N386 | LRRC25 |  |  |  |  |
| 3611 | 9317-4 | LRC25:ECD | Leucine-rich repeat-containing protein 25:Extracellular domain | Q8N386 | LRRC25 |  |  |  |  |
| 3612 | 6917-49 | LRRC3 | Leucine-rich repeat-containing protein 3 | Q9BY71 | LRRC3 |  |  |  |  |
| 3613 | 7551-33 | LRC32 | Leucine-rich repeat-containing protein 32 | Q14392 | LRRC32 |  |  |  |  |
| 3614 | 8897-3 | L37A2 | Leucine-rich repeat-containing protein 37A2 | A6NM11 | LRRC37A2 |  |  |  | X |
| 3615 | 18947-3 | LRC3B | Leucine-rich repeat-containing protein 3B | Q96PB8 | LRRC3B |  |  |  | X |
| 3616 | 21690-31 | LRRC4 | Leucine-rich repeat-containing protein 4 | Q9HBW1 | LRRC4 |  |  |  |  |
| 3617 | 11911-13 | LRC4B:CD | Leucine-rich repeat-containing protein 4B:Cytoplasmic domain | Q9NT99 | LRRC4B |  |  |  |  |
| 3618 | 9916-146 | LRC4B:ECD | Leucine-rich repeat-containing protein 4B:Extracellular domain | Q9NT99 | LRRC4B |  |  |  |  |
| 3619 | 9369-174 | NGL1 | Leucine-rich repeat-containing protein 4C | Q9HCJ2 | LRRC4C |  |  |  |  |
| 3620 | 10785-8 | LRC52 | Leucine-rich repeat-containing protein 52 | Q8N7C0 | LRRC52 |  |  |  |  |
| 3621 | 19163-26 | LRC59 | Leucine-rich repeat-containing protein 59 | Q96AG4 | LRRC59 |  |  |  | X |
| 3622 | 8389-8 | CN16B | Leucine-rich repeat-containing protein 74A | Q0VAA2 | LRRC74A |  |  |  |  |
| 3623 | 24718-8 | LR75A | Leucine-rich repeat-containing protein 75A | Q8NAA5 | LRRC75A |  |  |  |  |
| 3624 | 18831-6 | LRIG1 | Leucine-rich repeats and immunoglobulin-like domains protein 1 | Q96JA1 | LRIG1 | X |  |  |  |
| 3625 | 3322-52 | LRIG3 | Leucine-rich repeats and immunoglobulin-like domains protein 3 | Q6UXM1 | LRIG3 |  |  |  |  |
| 3626 | 7790-21 | LIF | Leukemia inhibitory factor | P15018 | LIF | X | X |  |  |
| 3627 | 5837-49 | LIF sR | Leukemia inhibitory factor receptor | P42702 | LIFR |  | X |  | X |
| 3628 | 18202-22 | CD37 | Leukocyte antigen CD37 | P11049 | CD37 |  |  |  |  |
| 3629 | 9928-125 | LECT1 | Leukocyte cell-derived chemotaxin 1 | O75829 | CNMD |  |  |  |  |
| 3630 | 16763-11 | LECT2 | Leukocyte cell-derived chemotaxin-2 | O14960 | LECT2 |  |  |  | X |
| 3631 | 5970-61 | LECT2 | Leukocyte cell-derived chemotaxin-2 | O14960 | LECT2 |  |  |  |  |
| 3632 | 10737-96 | Serpin B1 | Leukocyte elastase inhibitor | P30740 | SERPINB1 |  |  |  |  |
| 3633 | 21706-29 | LIRA1 | Leukocyte immunoglobulin-like receptor subfamily A member 1 | O75019 | LILRA1 |  |  |  |  |
| 3634 | 15614-168 | LIRA2 | Leukocyte immunoglobulin-like receptor subfamily A member 2 | Q8N149 | LILRA2 |  |  |  |  |
| 3635 | 6391-52 | LIRA3 | Leukocyte immunoglobulin-like receptor subfamily A member 3 | Q8N6C8 | LILRA3 |  |  |  |  |
| 3636 | 8299-66 | LIRA4 | Leukocyte immunoglobulin-like receptor subfamily A member 4 | P59901 | LILRA4 |  | X |  |  |
| 3637 | 7787-25 | LIRA5 | Leukocyte immunoglobulin-like receptor subfamily A member 5 | A6NI73 | LILRA5 |  | X |  |  |
| 3638 | 8766-29 | LIRA5 | Leukocyte immunoglobulin-like receptor subfamily A member 5 | A6NI73 | LILRA5 |  |  |  |  |
| 3639 | 7059-14 | LIRA6 | Leukocyte immunoglobulin-like receptor subfamily A member 6 | Q6PI73 | LILRA6 |  |  |  |  |
| 3640 | 5090-49 | ILT-2 | Leukocyte immunoglobulin-like receptor subfamily B member 1 | Q8NHL6 | LILRB1 | X |  | X |  |
| 3641 | 5091-28 | ILT-4 | Leukocyte immunoglobulin-like receptor subfamily B member 2 | Q8N423 | LILRB2 |  |  |  |  |
| 3642 | 5633-65 | ILT-4 | Leukocyte immunoglobulin-like receptor subfamily B member 2 | Q8N423 | LILRB2 |  |  |  |  |
| 3643 | 11334-7 | LIRB3 | Leukocyte immunoglobulin-like receptor subfamily B member 3 | O75022 | LILRB3 |  |  |  |  |
| 3644 | 15615-8 | LIRB3 | Leukocyte immunoglobulin-like receptor subfamily B member 3 | O75022 | LILRB3 |  |  |  |  |
| 3645 | 6453-70 | LIRB4 | Leukocyte immunoglobulin-like receptor subfamily B member 4 | Q8NHJ6 | LILRB4 |  |  |  |  |
| 3646 | 7015-8 | LIRB5 | Leukocyte immunoglobulin-like receptor subfamily B member 5 | O75023 | LILRB5 | X |  | X |  |
| 3647 | 6653-58 | CD47 | Leukocyte surface antigen CD47 | Q08722 | CD47 |  |  |  |  |
| 3648 | 23181-2 | LTK | Leukocyte tyrosine kinase receptor | P29376 | LTK |  |  |  |  |
| 3649 | 11284-24 | LAIR1 | Leukocyte-associated immunoglobulin-like receptor 1 | Q6GTX8 | LAIR1 |  | X |  |  |
| 3650 | 7130-4 | LAIR2 | Leukocyte-associated immunoglobulin-like receptor 2 | Q6ISS4 | LAIR2 |  |  |  |  |
| 3651 | 9531-24 | LST1 | Leukocyte-specific transcript 1 protein | O00453 | LST1 |  |  |  |  |
| 3652 | 12873-11 | LEUK | Leukosialin | P16150 | SPN | X |  | X |  |
| 3653 | 3204-2 | LKHA4 | Leukotriene A-4 hydrolase | P09960 | LTA4H | X |  |  |  |
| 3654 | 13477-65 | LT4R1 | Leukotriene B4 receptor 1 | Q15722 | LTB4R |  | X |  |  |
| 3655 | 23619-235 | L10K | Leydig cell tumor 10 kDa protein homolog | Q9UNZ5 | C19orf53 |  |  |  |  |
| 3656 | 4304-18 | transcription factor MLR1, isoform CRA_b | Ligand-dependent nuclear receptor corepressor-like protein | Q8N3X6 | LCORL |  |  |  |  |
| 3657 | 21862-145 | LASP-1 | LIM and SH3 domain protein 1 | Q14847 | LASP1 |  |  |  |  |
| 3658 | 11543-84 | LIMA1 | LIM domain and actin-binding protein 1 | Q9UHB6 | LIMA1 |  |  |  |  |
| 3659 | 25308-8 | LIMK1 | LIM domain kinase 1 | P53667 | LIMK1 | X |  |  |  |
| 3660 | 22492-108 | LMO3 | LIM domain only protein 3 | Q8TAP4 | LMO3 |  |  |  |  |
| 3661 | 23284-5 | LMO4 | LIM domain only transcription factor protein 4 | P61968 | LMO4 |  |  |  |  |

| # | **Custom Panel (X)** | **SOMAmer SeqID** | **Target Name** | **Human Target or Analyte** | **UniProt ID** | **GeneID** | **Cardiovascular Disease** | **Inflammation and Immune**  **Response** | **Metabolic Disease** | **Oncology** |
| --- | --- | --- | --- | --- | --- | --- | --- | --- | --- | --- |

| 3662 | 25926-29 | LDB1 | LIM domain-binding protein 1 | Q86U70 | LDB1 |  | | | |
| --- | --- | --- | --- | --- | --- | --- | --- | --- | --- |
| 3663 | 22491-10 | LDB2 | LIM domain-binding protein 2 | O43679 | LDB2 |
| 3664 | 25280-2 | LIMD1 | LIM domain-containing protein 1 | Q9UGP4 | LIMD1 |
| 3665 | 20991-2 | LIMD2 | LIM domain-containing protein 2 | Q9BT23 | LIMD2 |  |  |  | X |
| 3666 | 2999-6 | LSAMP | Limbic system-associated membrane protein | Q13449 | LSAMP |  |  |  |  |
| 3667 | 10551-7 | LAT | Linker for activation of T-cells family member 1 | O43561 | LAT |  |  | X |  |
| 3668 | 5613-75 | NTAL | Linker for activation of T-cells family member 2 | Q9GZY6 | LAT2 |  |  |  |  |
| 3669 | 6413-79 | LIPK | Lipase member K | Q5VXJ0 | LIPK |  |  |  |  |
| 3670 | 8097-77 | LIPN | Lipase member N | Q5VXI9 | LIPN |  |  |  |  |
| 3671 | 23673-9 | ODB2 | Lipoamide acyltransferase component of branched-chain alpha-keto acid dehydrogenase | P11182 | DBT |  |  | X |  |
| 3672 | 11708-2 | LCN1 | Lipocalin-1 | P31025 | LCN1 |  |  |  |  |
| 3673 | 22490-16 | LCNL1 | Lipocalin-like 1 protein | Q6ZST4 | LCNL1 |  |  |  |  |
| 3674 | 3074-6 | LBP | Lipopolysaccharide-binding protein | P18428 | LBP |  | X | X |  |
| 3675 | 21987-76 | LPL | Lipoprotein lipase | P06858 | LPL | X | X | X | X |
| 3676 | 13975-56 | LIPA1 | Liprin-alpha-1 | Q13136 | PPFIA1 |  |  |  |  |
| 3677 | 21655-18 | FOPNL | LisH domain-containing protein FOPNL | Q96NB1 | CEP20 |  |  |  |  |
| 3678 | 13095-51 | PSP | Lithostathine-1-alpha | P05451 | REG1A |  |  |  |  |
| 3679 | 16770-3 | REG1B | Lithostathine-1-beta | P48304 | REG1B |  |  |  |  |
| 3680 | 23018-4 | REG1B | Lithostathine-1-beta | P48304 | REG1B |  |  |  |  |
| 3681 | 5693-6 | REG1B | Lithostathine-1-beta | P48304 | REG1B |  |  |  |  |
| 3682 | 15487-164 | carboxylesterase, liver | Liver carboxylesterase 1 | P23141 | CES1 |  |  |  |  |
| 3683 | 7182-1 | carboxylesterase, liver | Liver carboxylesterase 1 | P23141 | CES1 | X |  | X |  |
| 3684 | 5708-1 | LEAP2 | Liver-expressed antimicrobial peptide 2 | Q969E1 | LEAP2 |  |  |  |  |
| 3685 | 15414-316 | LDHA | L-lactate dehydrogenase A chain | P00338 | LDHA | X |  | X |  |
| 3686 | 9761-89 | LDHA | L-lactate dehydrogenase A chain | P00338 | LDHA |  |  |  |  |
| 3687 | 3890-8 | LDH-H 1 | L-lactate dehydrogenase B chain | P07195 | LDHB | X |  | X | X |
| 3688 | 9828-86 | LDHC | L-lactate dehydrogenase C chain | P07864 | LDHC |  |  |  |  |
| 3689 | 6398-12 | LONM | Lon protease homolog, mitochondrial | P36776 | LONP1 |  |  | X |  |
| 3690 | 17857-6 | ACADL | Long-chain specific acyl-CoA dehydrogenase, mitochondrial | P28330 | ACADL |  |  | X |  |
| 3691 | 3291-30 | CD23 | Low affinity immunoglobulin epsilon Fc receptor | P06734 | FCER2 |  | X |  |  |
| 3692 | 3309-2 | FCG2A | Low affinity immunoglobulin gamma Fc region receptor II-a | P12318 | FCGR2A | X | X | X |  |
| 3693 | 22960-8 | FCG2B | Low affinity immunoglobulin gamma Fc region receptor II-b | P31994 | FCGR2B |  |  |  |  |
| 3694 | 3310-62 | FCG2B | Low affinity immunoglobulin gamma Fc region receptor II-b | P31994 | FCGR2B | X | X |  |  |
| 3695 | 15388-24 | FcRIIIa | Low affinity immunoglobulin gamma Fc region receptor III-A | P08637 | FCGR3A | X | X | X |  |
| 3696 | 3311-27 | FCG3B | Low affinity immunoglobulin gamma Fc region receptor III-B | O75015 | FCGR3B | X | X | X |  |
| 3697 | 18183-3 | ARH | Low density lipoprotein receptor adapter protein 1 | Q5SW96 | LDLRAP1 |  |  | X |  |
| 3698 | 3858-5 | PPAC | Low molecular weight phosphotyrosine protein phosphatase | P24666 | ACP1 |  |  |  |  |
| 3699 | 13129-40 | LDLR | Low-density lipoprotein receptor | P01130 | LDLR | X | X | X |  |
| 3700 | 22564-5 | LRAD3 | Low-density lipoprotein receptor class A domain-containing protein 3 | Q86YD5 | LDLRAD3 |  |  |  |  |
| 3701 | 9004-24 | CR001 | Low-density lipoprotein receptor class A domain-containing protein 4 | O15165 | LDLRAD4 |  |  |  |  |
| 3702 | 8601-167 | sLRP1:CD | Low-density lipoprotein receptor-related protein 1, soluble:Cytoplasmic domain | Q07954 | LRP1 |  |  |  |  |
| 3703 | 9182-3 | sLRP1:ECD | Low-density lipoprotein receptor-related protein 1, soluble:Extracellular domain, LDL receptor | Q07954 | LRP1 |  |  |  |  |
| 3704 | 16610-13 | LRP10 | Low-density lipoprotein receptor-related protein 10 | Q7Z4F1 | LRP10 |  |  |  |  |
| 3705 | 15472-16 | LRP11 | Low-density lipoprotein receptor-related protein 11 | Q86VZ4 | LRP11 |  |  |  |  |
| 3706 | 6713-4 | LRP11 | Low-density lipoprotein receptor-related protein 11 | Q86VZ4 | LRP11 |  |  |  |  |
| 3707 | 8330-1 | LRP11 | Low-density lipoprotein receptor-related protein 11 | Q86VZ4 | LRP11 |  |  |  |  |
| 3708 | 15506-34 | LRP12 | Low-density lipoprotein receptor-related protein 12 | Q9Y561 | LRP12 |  |  |  |  |
| 3709 | 7744-10 | LRP12 | Low-density lipoprotein receptor-related protein 12 | Q9Y561 | LRP12 |  |  |  |  |
| 3710 | 11275-94 | LRP1B | Low-density lipoprotein receptor-related protein 1B | Q9NZR2 | LRP1B |  |  |  | X |
| 3711 | 10618-190 | megalin | Low-density lipoprotein receptor-related protein 2 | P98164 | LRP2 |  |  | X |  |
| 3712 | 19558-10 | LRP4 | Low-density lipoprotein receptor-related protein 4 | O75096 | LRP4 |  |  |  |  |
| 3713 | 25922-7 | LRP5 | Low-density lipoprotein receptor-related protein 5 | O75197 | LRP5 | X |  | X |  |
| 3714 | 22584-2 | LRP6 | Low-density lipoprotein receptor-related protein 6 | O75581 | LRP6 | X |  |  |  |
| 3715 | 3323-37 | LRP8 | Low-density lipoprotein receptor-related protein 8 | Q14114 | LRP8 | X |  |  |  |
| 3716 | 24911-57 | LRP | LRP | Q14764 | MVP |  |  |  |  |
| 3717 | 21181-10 | LR2BP | LRP2-binding protein | Q9P2M1 | LRP2BP |  |  |  |  |
| 3718 | 4831-4 | sL-Selectin | L-Selectin | P14151 | SELL | X |  | X |  |
| 3719 | 13114-50 | Lumican | Lumican | P51884 | LUM |  |  |  |  |
| 3720 | 13625-19 | LA:HTH | Lupus La protein:HTH La-type RNA-binding domain | P05455 | SSB |  |  |  |  |
| 3721 | 13526-5 | LA:RRM | Lupus La protein:RNA recognition motif | P05455 | SSB |  |  |  |  |
| 3722 | 2953-31 | Luteinizing hormone | Luteinizing hormone | P01215|P01229 | CGA|LHB | X |  |  |  |
| 3723 | 8376-25 | LSHB | Lutropin subunit beta | P01229 | LHB | X |  |  |  |
| 3724 | 14634-13 | LSHR | Lutropin-choriogonadotropic hormone receptor | P22888 | LHCGR |  |  |  |  |
| 3725 | 17758-79 | DCXR | L-xylulose reductase | Q7Z4W1 | DCXR |  |  | X |  |
| 3726 | 9327-3 | LYPD1 | Ly6/PLAUR domain-containing protein 1 | Q8N2G4 | LYPD1 |  |  |  |  |
| 3727 | 13107-9 | LYPD3 | Ly6/PLAUR domain-containing protein 3 | O95274 | LYPD3 |  |  |  |  |
| 3728 | 3206-4 | LYVE1 | Lymphatic vessel endothelial hyaluronic acid receptor 1 | Q9Y5Y7 | LYVE1 | X |  | X |  |
| 3729 | 5099-14 | LAG-3 | Lymphocyte activation gene 3 protein | P18627 | LAG3 |  |  |  |  |
| 3730 | 9950-229 | LAG-3 | Lymphocyte activation gene 3 protein | P18627 | LAG3 |  |  |  |  |
| 3731 | 6256-9 | LY66C | Lymphocyte antigen 6 complex locus protein G6c | O95867 | LY6G6C |  |  |  |  |
| 3732 | 6469-62 | LY66D | Lymphocyte antigen 6 complex locus protein G6d | O95868 | LY6G6D |  |  |  |  |
| 3733 | 19784-16 | LY6D | Lymphocyte antigen 6D | Q14210 | LY6D |  |  |  |  |
| 3734 | 16620-26 | LY75 | Lymphocyte antigen 75 | O60449 | LY75 |  |  |  |  |
| 3735 | 3623-84 | LY86 | Lymphocyte antigen 86 | O95711 | LY86 | X |  | X |  |
| 3736 | 10938-13 | sLFA-3 | Lymphocyte function-associated antigen 3 | P19256 | CD58 |  | X |  |  |

| # | **Custom Panel (X)** | **SOMAmer SeqID** | **Target Name** | **Human Target or Analyte** | **UniProt ID** | **GeneID** | **Cardiovascular Disease** | **Inflammation and Immune**  **Response** | **Metabolic Disease** | **Oncology** |
| --- | --- | --- | --- | --- | --- | --- | --- | --- | --- | --- |

| 3737 | 21533-51 | LSP1 | Lymphocyte-specific protein 1 | P33241 | LSP1 | X |  | X | X |
| --- | --- | --- | --- | --- | --- | --- | --- | --- | --- |
| 3738 | 10704-91 | LRMP | Lymphoid-restricted membrane protein | Q12912 | IRAG2 |  |  |  |  |
| 3739 | 9096-80 | LRMP | Lymphoid-restricted membrane protein | Q12912 | IRAG2 |  |  |  |  |
| 3740 | 17475-18 | TOPK | Lymphokine-activated killer T-cell-originated protein kinase | Q96KB5 | PBK |  |  |  |  |
| 3741 | 14078-69 | Lymphotactin | Lymphotactin | P47992 | XCL1 |  |  |  |  |
| 3742 | 4143-74 | Lymphotactin | Lymphotactin | P47992 | XCL1 |  |  |  |  |
| 3743 | 3505-6 | Lymphotoxin a1/b2 | Lymphotoxin alpha1:beta2 | P01374|Q06643 | LTA|LTB | X | X | X |  |
| 3744 | 3506-49 | Lymphotoxin a2/b1 | Lymphotoxin alpha2:beta1 | P01374|Q06643 | LTA|LTB |  |  |  |  |
| 3745 | 4703-87 | TNF-b | Lymphotoxin-alpha | P01374 | LTA | X | X |  |  |
| 3746 | 22496-21 | LYRM1 | LYR motif-containing protein 1 | O43325 | LYRM1 |  |  |  |  |
| 3747 | 25298-53 | KDM4C | Lysine-specific demethylase 4C | Q9H3R0 | KDM4C |  | X |  | X |
| 3748 | 24307-22 | KDM8 | Lysine-specific demethylase 8 | Q8N371 | KDM8 |  |  |  |  |
| 3749 | 22429-13 | KDM1A | Lysine-specific histone demethylase 1A | O60341 | KDM1A |  | X |  | X |
| 3750 | 19249-18 | SYK | Lysine--tRNA ligase | Q15046 | KARS1 |  |  |  |  |
| 3751 | 10563-13 | LYSM3 | LysM and putative peptidoglycan-binding domain-containing protein 3 | Q7Z3D4 | LYSMD3 |  |  |  |  |
| 3752 | 9106-87 | LYSM4 | LysM and putative peptidoglycan-binding domain-containing protein 4 | Q5XG99 | LYSMD4 |  |  |  |  |
| 3753 | 5742-14 | PPA6 | Lysophosphatidic acid phosphatase type 6 | Q9NPH0 | ACP6 |  |  |  |  |
| 3754 | 11175-45 | PCAT2 | Lysophosphatidylcholine acyltransferase 2 | Q7L5N7 | LPCAT2 |  |  |  |  |
| 3755 | 24428-76 | GDPD1 | Lysophospholipase D GDPD1 | Q8N9F7 | GDPD1 |  |  |  |  |
| 3756 | 12428-2 | LYPL1 | Lysophospholipase-like protein 1 | Q5VWZ2 | LYPLAL1 |  |  |  |  |
| 3757 | 9237-54 | Lysosomal acid phosphatase | Lysosomal acid phosphatase | P11117 | ACP2 |  |  | X |  |
| 3758 | 9385-4 | GAA | Lysosomal alpha-glucosidase | P10253 | GAA | X | X | X |  |
| 3759 | 3179-51 | Cathepsin A | Lysosomal protective protein | P10619 | CTSA |  |  | X |  |
| 3760 | 5722-78 | Prolylcarboxypeptidase | Lysosomal Pro-X carboxypeptidase | P42785 | PRCP |  |  |  |  |
| 3761 | 5100-53 | LIMP II | Lysosome membrane protein 2 | Q14108 | SCARB2 |  |  | X |  |
| 3762 | 16902-17 | LAMP1 | Lysosome-associated membrane glycoprotein 1 | P11279 | LAMP1 |  |  |  |  |
| 3763 | 4920-10 | Lysozyme | Lysozyme C | P61626 | LYZ |  |  | X |  |
| 3764 | 9243-10 | LYG1 | Lysozyme g-like protein 1 | Q8N1E2 | LYG1 |  |  |  |  |
| 3765 | 15416-54 | LYG2 | Lysozyme g-like protein 2 | Q86SG7 | LYG2 |  |  |  |  |
| 3766 | 6377-54 | LYZL2 | Lysozyme-like protein 2 | Q7Z4W2 | LYZL2 |  |  |  |  |
| 3767 | 6504-65 | Lysyl oxidase-like protein 2 | Lysyl oxidase homolog 2 | Q9Y4K0 | LOXL2 |  | X | X | X |
| 3768 | 15427-35 | LOXL3 | Lysyl oxidase homolog 3 | P58215 | LOXL3 |  |  |  |  |
| 3769 | 12367-52 | MACOI | Macoilin | Q8N5G2 | MACO1 | X |  | X |  |
| 3770 | 10741-22 | CSF-1 | Macrophage colony-stimulating factor 1 | P09603 | CSF1 |  |  |  |  |
| 3771 | 3738-54 | CSF-1 | Macrophage colony-stimulating factor 1 | P09603 | CSF1 |  | X |  | X |
| 3772 | 13682-47 | M-CSF R | Macrophage colony-stimulating factor 1 receptor | P07333 | CSF1R |  |  |  | X |
| 3773 | 2638-12 | M-CSF R | Macrophage colony-stimulating factor 1 receptor | P07333 | CSF1R |  |  |  |  |
| 3774 | 21477-105 | MAEA | Macrophage erythroblast attacher | Q7L5Y9 | MAEA |  |  | X |  |
| 3775 | 2637-77 | Macrophage mannose receptor | Macrophage mannose receptor 1 | P22897 | MRC1 |  | X | X |  |
| 3776 | 4496-60 | MMP-12 | Macrophage metalloelastase | P39900 | MMP12 | X | X | X |  |
| 3777 | 5356-2 | MIF | Macrophage migration inhibitory factor | P14174 | MIF |  |  |  |  |
| 3778 | 8221-19 | MIF | Macrophage migration inhibitory factor | P14174 | MIF |  | X |  | X |
| 3779 | 20134-27 | MARCO | Macrophage receptor MARCO | Q9UEW3 | MARCO | X |  |  |  |
| 3780 | 9003-99 | MARCO | Macrophage receptor MARCO | Q9UEW3 | MARCO |  |  |  |  |
| 3781 | 11207-3 | Macrophage scavenger receptor:CD | Macrophage scavenger receptor types I and II:Cytoplasmic domain | P21757 | MSR1 |  |  |  | X |
| 3782 | 11282-16 | Macrophage scavenger receptor:ECD | Macrophage scavenger receptor types I and II:Extracellular domain | P21757 | MSR1 |  |  |  | X |
| 3783 | 15533-97 | Macrophage scavenger receptor:ECD | Macrophage scavenger receptor types I and II:Extracellular domain | P21757 | MSR1 |  |  |  |  |
| 3784 | 3684-78 | Macrophage scavenger receptor:ECD | Macrophage scavenger receptor types I and II:Extracellular domain | P21757 | MSR1 |  |  |  |  |
| 3785 | 4968-50 | CAPG | Macrophage-capping protein | P40121 | CAPG |  |  |  | X |
| 3786 | 2640-3 | MSP R | Macrophage-stimulating protein receptor | Q04912 | MST1R |  |  |  |  |
| 3787 | 18922-27 | CD68 | Macrosialin | P34810 | CD68 |  |  |  |  |
| 3788 | 20528-23 | CD68 | Macrosialin | P34810 | CD68 |  |  | X |  |
| 3789 | 21129-95 | MD2BP | MAD2L1-binding protein | Q15013 | MAD2L1BP |  |  |  |  |
| 3790 | 12864-9 | NIPA4 | Magnesium transporter NIPA4 | Q0D2K0 | NIPAL4 |  |  |  |  |
| 3791 | 20923-10 | MGDP1 | Magnesium-dependent phosphatase 1 | Q86V88 | MDP1 |  |  |  |  |
| 3792 | 24265-6 | MPP2 | MAGUK p55 subfamily member 2 | Q14168 | MPP2 |  |  |  |  |
| 3793 | 24982-33 | MPP5 | MAGUK p55 subfamily member 5 | Q8N3R9 | MPP5 |  |  |  |  |
| 3794 | 13490-1 | MPP6 | MAGUK p55 subfamily member 6 | Q9NZW5 | MPP6 |  |  |  |  |
| 3795 | 12732-13 | MPP7 | MAGUK p55 subfamily member 7 | Q5T2T1 | MPP7 |  |  |  |  |
| 3796 | 6545-58 | PRIO | Major prion protein | P04156 | PRNP |  | X | X | X |
| 3797 | 3853-56 | MDHC | Malate dehydrogenase, cytoplasmic | P40925 | MDH1 | X |  |  |  |
| 3798 | 12649-80 | Malate dehydrogenase 2 | Malate dehydrogenase, mitochondrial | P40926 | MDH2 |  |  |  |  |
| 3799 | 15534-26 | Malate dehydrogenase 2 | Malate dehydrogenase, mitochondrial | P40926 | MDH2 |  |  |  | X |
| 3800 | 6285-71 | K0152 | Malectin | Q14165 | MLEC |  |  |  |  |
| 3801 | 16872-248 | MAAI | Maleylacetoacetate isomerase | O43708 | GSTZ1 |  |  |  |  |
| 3802 | 12488-9 | MCTS1 | Malignant T-cell-amplified sequence 1 | Q9ULC4 | MCTS1 |  |  |  |  |
| 3803 | 11538-216 | DCMC | Malonyl-CoA decarboxylase, mitochondrial | O95822 | MLYCD |  |  | X |  |
| 3804 | 16900-29 | MDGA1 | MAM domain-containing glycosylphosphatidylinositol anchor protein 1 | Q8NFP4 | MDGA1 |  |  |  |  |
| 3805 | 19372-7 | MDGA2 | MAM domain-containing glycosylphosphatidylinositol anchor protein 2 | Q7Z553 | MDGA2 |  |  |  |  |
| 3806 | 6119-14 | MAMC2 | MAM domain-containing protein 2 | Q7Z304 | MAMDC2 |  |  |  |  |
| 3807 | 5001-6 | Mammaglobin 2 | Mammaglobin-B | O75556 | SCGB2A1 |  |  |  |  |
| 3808 | 9086-95 | Mammaglobin 2 | Mammaglobin-B | O75556 | SCGB2A1 |  |  |  |  |
| 3809 | 9040-144 | EPDR1 | Mammalian ependymin-related protein 1 | Q9UM22 | EPDR1 |  |  |  |  |
| 3810 | 13659-36 | AT131 | Manganese-transporting ATPase 13A1 | Q9HD20 | ATP13A1 |  |  |  |  |
| 3811 | 8091-16 | MASP3:Heavy | Mannan-binding lectin serine protease 1:Mannan-binding lectin serine protease 1 heavy chain | P48740 | MASP1 | X | X |  |  |

| # | **Custom Panel (X)** | **SOMAmer SeqID** | **Target Name** | **Human Target or Analyte** | **UniProt ID** | **GeneID** | **Cardiovascular Disease** | **Inflammation and Immune**  **Response** | **Metabolic Disease** | **Oncology** |
| --- | --- | --- | --- | --- | --- | --- | --- | --- | --- | --- |

| 3812 | 3605-77 | MASP3:Light | Mannan-binding lectin serine protease 1:Mannan-binding lectin serine protease 1 light chain | P48740 | MASP1 | X | X |  |  |
| --- | --- | --- | --- | --- | --- | --- | --- | --- | --- |
| 3813 | 9125-23 | MASP3:Sushi 1 and Sushi 2 | Mannan-binding lectin serine protease 1:Sushi 1 and Sushi 2 | P48740 | MASP1 | X | X |  |
| 3814 | 24271-9 | GMPPA | Mannose-1-phosphate guanyltransferase alpha | Q96IJ6 | GMPPA |  |  |  |
| 3815 | 25233-2 | MPI | Mannose-6-phosphate isomerase | P34949 | MPI |  |  | X |
| 3816 | 3000-66 | MBL | Mannose-binding protein C | P11226 | MBL2 | X | X | X |
| 3817 | 9077-10 | MA1A2 | Mannosyl-oligosaccharide 1,2-alpha-mannosidase IB | O60476 | MAN1A2 |  |  |  |
| 3818 | 13427-66 | MA1C1 | Mannosyl-oligosaccharide 1,2-alpha-mannosidase IC | Q9NR34 | MAN1C1 |  |  |  |
| 3819 | 9557-5 | MANS1 | MANSC domain-containing protein 1 | Q9H8J5 | MANSC1 |  |  |  |
| 3820 | 9578-263 | MANS4 | MANSC domain-containing protein 4 | A6NHS7 | MANSC4 |  |  |  |
| 3821 | 3820-68 | MAPK2 | MAP kinase-activated protein kinase 2 | P49137 | MAPKAPK2 |  |  |  |
| 3822 | 3822-54 | MAPKAPK3 | MAP kinase-activated protein kinase 3 | Q16644 | MAPKAPK3 |  |  |  |
| 3823 | 3821-28 | MAPK5 | MAP kinase-activated protein kinase 5 | Q8IW41 | MAPKAPK5 |  |  |  |
| 3824 | 8382-47 | MAPK5 | MAP kinase-activated protein kinase 5 | Q8IW41 | MAPKAPK5 |  |  |  |
| 3825 | 23693-4 | MKNK1 | MAP kinase-interacting serine/threonine-protein kinase 1 | Q9BUB5 | MKNK1 |  |  |  |
| 3826 | 9747-48 | MARK3 | MAP/microtubule affinity-regulating kinase 3 | P27448 | MARK3 |  |  |  |
| 3827 | 24661-4 | MA6D1 | MAP6 domain-containing protein 1 | Q9H9H5 | MAP6D1 |  |  |  |
| 3828 | 19175-18 | MARCKSL1 | MARCKS-related protein | P49006 | MARCKSL1 |  |  |  |
| 3829 | 16322-10 | PACAP | Marginal zone B- and B1-cell-specific protein | Q8WU39 | MZB1 |  |  |  |
| 3830 | 7780-34 | PACAP | Marginal zone B- and B1-cell-specific protein | Q8WU39 | MZB1 |  |  |  |
| 3831 | 21810-50 | MART-1 | MART-1 | Q16655 | MLANA |  |  |  |
| 3832 | 23611-16 | MALD2 | MARVEL domain-containing protein 2 | Q8N4S9 | MARVELD2 |  |  |  |
| 3833 | 11122-97 | SPG21 | Maspardin | Q9NZD8 | SPG21 |  |  |  |
| 3834 | 6283-60 | MCEM1 | Mast cell-expressed membrane protein 1 | Q8IX19 | MCEMP1 |  |  |  |
| 3835 | 2475-1 | SCF sR | Mast/stem cell growth factor receptor Kit | P10721 | KIT | X | X | X | X |
| 3836 | 3325-2 | MATN2 | Matrilin-2 | O00339 | MATN2 |  |  |  |  |
| 3837 | 19361-78 | MATN3 | Matrilin-3 | O15232 | MATN3 |  |  |  |  |
| 3838 | 3208-2 | MATN3 | Matrilin-3 | O15232 | MATN3 |  |  |  |  |
| 3839 | 7083-74 | MATN4 | Matrilin-4 | O95460 | MATN4 |  |  |  |  |
| 3840 | 2789-26 | MMP-7 | Matrilysin | P09237 | MMP7 | X | X |  | X |
| 3841 | 8475-15 | MMP-7 | Matrilysin | P09237 | MMP7 |  |  |  |  |
| 3842 | 3209-69 | MEPE | Matrix extracellular phosphoglycoprotein | Q9NQ76 | MEPE | X |  | X |  |
| 3843 | 6520-87 | MGP | Matrix Gla protein | P08493 | MGP | X |  | X | X |
| 3844 | 5002-76 | MMP-14 | Matrix metalloproteinase-14 | P50281 | MMP14 | X |  | X | X |
| 3845 | 5268-49 | MMP-16 | Matrix metalloproteinase-16 | P51512 | MMP16 |  |  |  |  |
| 3846 | 9719-145 | MMP-16 | Matrix metalloproteinase-16 | P51512 | MMP16 |  |  |  |  |
| 3847 | 2838-53 | MMP-17 | Matrix metalloproteinase-17 | Q9ULZ9 | MMP17 |  |  |  |  |
| 3848 | 6425-87 | MMP19 | Matrix metalloproteinase-19 | Q99542 | MMP19 |  |  |  |  |
| 3849 | 15419-15 | MMP20 | Matrix metalloproteinase-20 | O60882 | MMP20 |  |  |  |  |
| 3850 | 6374-7 | MMP20 | Matrix metalloproteinase-20 | O60882 | MMP20 |  |  |  |  |
| 3851 | 2579-17 | MMP-9 | Matrix metalloproteinase-9 | P14780 | MMP9 | X | X | X | X |
| 3852 | 8005-1 | MXRA7 | Matrix-remodeling-associated protein 7 | P84157 | MXRA7 |  |  |  |  |
| 3853 | 7002-1 | MXRA8:CD | Matrix-remodeling-associated protein 8:Cytoplasmic domain | Q9BRK3 | MXRA8 |  |  |  |  |
| 3854 | 10521-10 | MXRA8:ECD | Matrix-remodeling-associated protein 8:Extracellular domain | Q9BRK3 | MXRA8 |  |  |  |  |
| 3855 | 11587-5 | MGAP | MAX gene-associated protein | Q8IWI9 | MGA |  | X |  | X |
| 3856 | 9035-2 | MXI1 | MAX-interacting protein 1 | P50539 | MXI1 |  |  |  |  |
| 3857 | 23294-19 | MLX | Max-like protein X | Q9UH92 | MLX | X |  |  |  |
| 3858 | 23680-1 | MCPIP | MCP-1 Induced Protein | Q5D1E8 | ZC3H12A | X |  |  |  |
| 3859 | 3892-21 | MED-1 | Mediator of RNA polymerase II transcription subunit 1 | Q15648 | MED1 |  |  |  |  |
| 3860 | 23264-42 | MED10 | Mediator of RNA polymerase II transcription subunit 10 | Q9BTT4 | MED10 |  |  |  |  |
| 3861 | 22500-9 | MED11 | Mediator of RNA polymerase II transcription subunit 11 | Q9P086 | MED11 |  |  |  |  |
| 3862 | 20427-18 | MED20 | Mediator of RNA polymerase II transcription subunit 20 | Q9H944 | MED20 |  |  |  |  |
| 3863 | 22499-15 | MED28 | Mediator of RNA polymerase II transcription subunit 28 | Q9H204 | MED28 |  |  |  | X |
| 3864 | 14021-81 | MED4 | Mediator of RNA polymerase II transcription subunit 4 | Q9NPJ6 | MED4 |  |  |  |  |
| 3865 | 17333-20 | ACADM | Medium-chain specific acyl-CoA dehydrogenase, mitochondrial | P11310 | ACADM |  |  | X |  |
| 3866 | 3823-9 | MATK | Megakaryocyte-associated tyrosine-protein kinase | P42679 | MATK |  |  |  |  |
| 3867 | 23685-132 | M1AP | Meiosis 1 arrest protein | Q8TC57 | M1AP |  |  |  |  |
| 3868 | 24286-48 | MEIG1 | Meiosis expressed gene 1 protein homolog | Q5JSS6 | MEIG1 |  |  |  |  |
| 3869 | 23341-16 | DMC1 | Meiotic recombination protein DMC1/LIM15 homolog | Q14565 | DMC1 |  |  |  |  |
| 3870 | 7895-108 | MRAP | Melanocortin-2 receptor accessory protein | Q8TCY5 | MRAP |  |  | X |  |
| 3871 | 10889-2 | MRAP2 | Melanocortin-2 receptor accessory protein 2 | Q96G30 | MRAP2 |  |  | X |  |
| 3872 | 6472-40 | GP100 | Melanocyte protein PMEL | P40967 | PMEL |  |  |  |  |
| 3873 | 13610-9 | MAGE-10 | Melanoma-associated antigen 10 | P43363 | MAGEA10 |  |  |  |  |
| 3874 | 12576-21 | MAGE-3 | Melanoma-associated antigen 3 | P43357 | MAGEA3 |  |  |  |  |
| 3875 | 20133-1 | MAGE-3 | Melanoma-associated antigen 3 | P43357 | MAGEA3 |  |  |  |  |
| 3876 | 10485-56 | MAGE-4 | Melanoma-associated antigen 4 | P43358 | MAGEA4 |  |  |  |  |
| 3877 | 20075-130 | MAGE-4 | Melanoma-associated antigen 4 | P43358 | MAGEA4 |  |  |  |  |
| 3878 | 18192-69 | MAGE-5 | Melanoma-associated antigen 5 | P43359 | MAGEA5 |  |  |  |  |
| 3879 | 18205-123 | MAGE-6 | Melanoma-associated antigen 6 | P43360 | MAGEA6 |  |  |  |  |
| 3880 | 22991-9 | MAGE-6 | Melanoma-associated antigen 6 | P43360 | MAGEA6 |  |  |  |  |
| 3881 | 18326-50 | MAGA8 | Melanoma-associated antigen 8 | P43361 | MAGEA8 |  |  |  |  |
| 3882 | 11456-2 | MAGBA | Melanoma-associated antigen B10 | Q96LZ2 | MAGEB10 |  |  |  |  |
| 3883 | 21392-15 | MAGD1 | Melanoma-associated antigen D1 | Q9Y5V3 | MAGED1 |  |  |  |  |
| 3884 | 20512-2 | MUC18 | Melanoma-associated antigen MUC18 | P43121 | MCAM |  | X |  |  |
| 3885 | 2687-2 | MIA | Melanoma-derived growth regulatory protein | Q16674 | MIA |  |  |  | X |
| 3886 | 19145-4 | MREG | Melanoregulin | Q8N565 | MREG |  |  |  |  |

| # | **Custom Panel (X)** | **SOMAmer SeqID** | **Target Name** | **Human Target or Analyte** | **UniProt ID** | **GeneID** | **Cardiovascular Disease** | **Inflammation and Immune**  **Response** | **Metabolic Disease** | **Oncology** |
| --- | --- | --- | --- | --- | --- | --- | --- | --- | --- | --- |

| 3887 | 17682-1 | CD46 | Membrane cofactor protein | P15529 | CD46 | X | X | X |  |
| --- | --- | --- | --- | --- | --- | --- | --- | --- | --- |
| 3888 | 3685-53 | MFRP | Membrane frizzled-related protein | Q9BY79 | MFRP |  |  |  |
| 3889 | 7225-51 | MMGT1 | Membrane magnesium transporter 1 | Q8N4V1 | MMGT1 |  |  |  |
| 3890 | 3627-71 | MMEL2 | Membrane metallo-endopeptidase-like 1 | Q495T6 | MMEL1 |  | X |  |
| 3891 | 13431-74 | F159A | Membrane protein FAM159A | Q6UWV7 | SHISAL2A |  |  |  |
| 3892 | 6597-24 | TM157 | Membrane protein FAM174A | Q8TBP5 | FAM174A |  |  |  |
| 3893 | 14066-49 | MAGI2 | Membrane-associated guanylate kinase, WW and PDZ domain-containing protein 2 | Q86UL8 | MAGI2 |  |  | X |
| 3894 | 25058-23 | PITM3 | Membrane-associated phosphatidylinositol transfer protein 3 | Q9BZ71 | PITPNM3 |  |  |  |
| 3895 | 7863-50 | PGRC1 | Membrane-associated progesterone receptor component 1 | O00264 | PGRMC1 |  |  |  |
| 3896 | 10506-53 | PGRC2 | Membrane-associated progesterone receptor component 2 | O15173 | PGRMC2 |  |  |  |
| 3897 | 10631-9 | PGRC2 | Membrane-associated progesterone receptor component 2 | O15173 | PGRMC2 |  |  |  |
| 3898 | 8681-93 | PGRC2:N-term | Membrane-associated progesterone receptor component 2:N-term, Isoform 2 | O15173 | PGRMC2 |  |  |  |
| 3899 | 7240-2 | MBTP1 | Membrane-bound transcription factor site-1 protease | Q14703 | MBTPS1 |  |  |  |
| 3900 | 24896-5 | MEN1 | Menin | O00255 | MEN1 |  |  |  | X |
| 3901 | 19786-26 | MEP1A | Meprin A subunit alpha | Q16819 | MEP1A |  |  |  |  |
| 3902 | 21387-64 | MEP1A | Meprin A subunit alpha | Q16819 | MEP1A |  |  |  |  |
| 3903 | 9223-11 | ARMET | Mesencephalic astrocyte-derived neurotrophic factor | P55145 | MANF |  |  |  |  |
| 3904 | 21172-11 | MESD1 | Mesoderm development candidate 1 | Q9H1K6 | TLNRD1 |  |  |  |  |
| 3905 | 3893-64 | Mesothelin | Mesothelin | Q13421 | MSLN |  |  |  | X |
| 3906 | 24013-6 | GRM4 | Metabotropic glutamate receptor 4 | Q14833 | GRM4 |  |  |  |  |
| 3907 | 19153-53 | MPPD2 | Metallophosphoesterase MPPED2 | Q15777 | MPPED2 |  |  |  |  |
| 3908 | 9401-57 | CB089 | Metalloprotease TIKI1 | Q86V40 | TRABD2A |  |  |  |  |
| 3909 | 2211-9 | TIMP-1 | Metalloproteinase inhibitor 1 | P01033 | TIMP1 |  |  |  |  |
| 3910 | 23173-3 | TIMP-1 | Metalloproteinase inhibitor 1 | P01033 | TIMP1 | X | X | X |  |
| 3911 | 25967-34 | TIMP-1 | Metalloproteinase inhibitor 1 | P01033 | TIMP1 |  |  |  |  |
| 3912 | 2278-61 | TIMP-2 | Metalloproteinase inhibitor 2 | P16035 | TIMP2 | X |  | X |  |
| 3913 | 2480-58 | TIMP-3 | Metalloproteinase inhibitor 3 | P35625 | TIMP3 |  | X |  |  |
| 3914 | 6462-12 | TIMP-4 | Metalloproteinase inhibitor 4 | Q99727 | TIMP4 | X |  |  |  |
| 3915 | 6606-61 | KISS1 | Metastasis-suppressor KiSS-1 | Q15726 | KISS1 |  |  |  | X |
| 3916 | 8839-4 | Metaxin-2 | Metaxin-2 | O75431 | MTX2 |  |  |  |  |
| 3917 | 21705-33 | METRL | Meteorin-like protein | Q641Q3 | METRNL |  |  | X |  |
| 3918 | 19129-15 | MTHFSD | Methenyltetrahydrofolate synthase domain-containing protein | Q2M296 | MTHFSD |  |  |  |  |
| 3919 | 18392-19 | MAT2B | Methionine adenosyltransferase 2 subunit beta | Q9NZL9 | MAT2B |  |  |  |  |
| 3920 | 3210-1 | METAP1 | Methionine aminopeptidase 1 | P53582 | METAP1 |  |  |  |  |
| 3921 | 19752-197 | AMP1D | Methionine aminopeptidase 1D, mitochondrial | Q6UB28 | METAP1D |  |  |  |  |
| 3922 | 3170-6 | AMPM2 | Methionine aminopeptidase 2 | P50579 | METAP2 |  |  |  |  |
| 3923 | 20971-1 | MSRB1 | Methionine-R-sulfoxide reductase B1 | Q9NZV6 | MSRB1 |  |  |  |  |
| 3924 | 16877-19 | MSRB2 | Methionine-R-sulfoxide reductase B2, mitochondrial | Q9Y3D2 | MSRB2 |  |  |  |  |
| 3925 | 7824-88 | MSRB3 | Methionine-R-sulfoxide reductase B3 | Q8IXL7 | MSRB3 |  |  |  |  |
| 3926 | 24425-8 | SYMC | Methionyl-tRNA synthetase, cytoplasmic | P56192 | MARS1 |  |  |  |  |
| 3927 | 21437-77 | MGMT | Methylated-DNA--protein-cysteine methyltransferase | P16455 | MGMT |  | X |  | X |
| 3928 | 14294-61 | MBD1 | Methyl-CpG-binding domain protein 1 | Q9UIS9 | MBD1 |  |  |  |  |
| 3929 | 3891-56 | MBD4 | Methyl-CpG-binding domain protein 4 | O95243 | MBD4 |  |  |  |  |
| 3930 | 19108-50 | MECP2 | Methyl-CpG-binding protein 2 | P51608 | MECP2 |  | X |  |  |
| 3931 | 20931-156 | AUHM | Methylglutaconyl-CoA hydratase, mitochondrial | Q13825 | AUH |  |  | X |  |
| 3932 | 19136-22 | MMSA | Methylmalonate-semialdehyde dehydrogenase [acylating], mitochondrial | Q02252 | ALDH6A1 | X |  | X |  |
| 3933 | 20953-34 | MMAC | Methylmalonic aciduria and homocystinuria type C protein | Q9Y4U1 | MMACHC | X |  | X |  |
| 3934 | 21814-13 | MMAD | Methylmalonic aciduria and homocystinuria type D protein, mitochondrial | Q9H3L0 | MMADHC |  |  | X |  |
| 3935 | 9388-18 | MCEE | Methylmalonyl-CoA epimerase, mitochondrial | Q96PE7 | MCEE |  |  | X |  |
| 3936 | 23340-37 | MTNA | Methylthioribose-1-phosphate isomerase | Q9BV20 | MRI1 |  |  |  |  |
| 3937 | 25236-11 | MTL26 | Methyltransferase-like 26 | Q96S19 | METTL26 |  |  |  |  |
| 3938 | 9470-15 | MET24 | Methyltransferase-like protein 24 | Q5JXM2 | METTL24 |  |  |  |  |
| 3939 | 23558-33 | MET2B | Methyltransferase-like protein 2B | Q6P1Q9 | METTL2B |  |  |  |  |
| 3940 | 11391-69 | MVK | Mevalonate kinase | Q03426 | MVK |  | X | X |  |
| 3941 | 2730-58 | MICA | MHC class I polypeptide-related sequence A | Q29983 | MICA | X | X |  | X |
| 3942 | 5102-55 | MICB | MHC class I polypeptide-related sequence B | Q29980 | MICB |  | X |  |  |
| 3943 | 9328-55 | MICB | MHC class I polypeptide-related sequence B | Q29980 | MICB |  |  |  |  |
| 3944 | 24973-11 | MILK1 | MICAL-like protein 1 | Q8N3F8 | MICALL1 |  |  |  |  |
| 3945 | 12891-1 | MILK2 | MICAL-like protein 2 | Q8IY33 | MICALL2 |  |  |  |  |
| 3946 | 7956-11 | MOS1 | MICOS complex subunit MIC10 | Q5TGZ0 | MICOS10 |  |  |  |  |
| 3947 | 20545-17 | MFAP3 | Microfibril-associated glycoprotein 3 | P55082 | MFAP3 |  |  |  |  |
| 3948 | 5636-10 | MFAP4 | Microfibril-associated glycoprotein 4 | P55083 | MFAP4 |  |  |  |  |
| 3949 | 5606-24 | MFAP1 | Microfibrillar-associated protein 1 | P55081 | MFAP1 |  |  |  |  |
| 3950 | 10569-28 | MFAP2 | Microfibrillar-associated protein 2 | P55001 | MFAP2 |  |  |  |  |
| 3951 | 9294-45 | MFAP2 | Microfibrillar-associated protein 2 | P55001 | MFAP2 |  |  |  |  |
| 3952 | 8837-8 | MFA3L | Microfibrillar-associated protein 3-like | O75121 | MFAP3L |  |  |  |  |
| 3953 | 6440-31 | MFAP5 | Microfibrillar-associated protein 5 | Q13361 | MFAP5 | X |  | X |  |
| 3954 | 12469-19 | MARE1 | Microtubule-associated protein RP/EB family member 1 | Q15691 | MAPRE1 | X |  |  |  |
| 3955 | 19371-18 | MARE1 | Microtubule-associated protein RP/EB family member 1 | Q15691 | MAPRE1 |  |  |  |  |
| 3956 | 16856-79 | MARE2 | Microtubule-associated protein RP/EB family member 2 | Q15555 | MAPRE2 |  |  |  |  |
| 3957 | 16885-49 | MARE3 | Microtubule-associated protein RP/EB family member 3 | Q9UPY8 | MAPRE3 |  |  |  |  |
| 3958 | 22992-6 | MARE3 | Microtubule-associated protein RP/EB family member 3 | Q9UPY8 | MAPRE3 |  |  |  |  |
| 3959 | 5854-60 | tau | Microtubule-associated protein tau | P10636 | MAPT | X |  | X |  |
| 3960 | 20965-18 | MP3B2 | Microtubule-associated proteins 1A/1B light chain 3 beta 2 | A6NCE7 | MAP1LC3B2 |  |  |  |  |
| 3961 | 17781-191 | MLP3A | Microtubule-associated proteins 1A/1B light chain 3A | Q9H492 | MAP1LC3A |  |  |  |  |

| # | **Custom Panel (X)** | **SOMAmer SeqID** | **Target Name** | **Human Target or Analyte** | **UniProt ID** | **GeneID** | **Cardiovascular Disease** | **Inflammation and Immune**  **Response** | **Metabolic Disease** | **Oncology** |
| --- | --- | --- | --- | --- | --- | --- | --- | --- | --- | --- |

| 3962 | 11608-5 | MLP3B | Microtubule-associated proteins 1A/1B light chain 3B | Q9GZQ8 | MAP1LC3B |  | | | |
| --- | --- | --- | --- | --- | --- | --- | --- | --- | --- |
| 3963 | 24675-2 | MAST4 | Microtubule-associated serine/threonine-protein kinase 4 | O15021 | MAST4 |
| 3964 | 24710-1 | MTUS2 | Microtubule-associated tumor suppressor candidate 2 | Q5JR59 | MTUS2 |
| 3965 | 2911-27 | Midkine | Midkine | P21741 | MDK |  |  |  | X |
| 3966 | 17224-12 | MIME | Mimecan | P20774 | OGN |  |  |  |  |
| 3967 | 21120-3 | MIMIT | Mimitin, mitochondrial | Q8N183 | NDUFAF2 |  |  | X |  |
| 3968 | 12931-16 | MCR | Mineralocorticoid receptor | P08235 | NR3C2 | X |  |  |  |
| 3969 | 11312-40 | PMS2 | Mismatch repair endonuclease PMS2 | P54278 | PMS2 |  |  | X | X |
| 3970 | 23266-62 | MITD1 | MIT domain-containing protein 1 | Q8WV92 | MITD1 |  |  |  |  |
| 3971 | 22383-21 | MITF | MITF | O75030 | MITF |  |  | X | X |
| 3972 | 8032-23 | MCCD1 | Mitochondrial coiled-coil domain protein 1 | P59942 | MCCD1 |  |  |  |  |
| 3973 | 23668-281 | MID51 | Mitochondrial dynamics protein MID51 | Q9NQG6 | MIEF1 |  |  |  |  |
| 3974 | 17746-77 | FIS1 | Mitochondrial fission 1 protein | Q9Y3D6 | FIS1 |  |  | X |  |
| 3975 | 9095-5 | MTFR1 | Mitochondrial fission regulator 1 | Q15390 | MTFR1 |  |  |  |  |
| 3976 | 5280-68 | GHC2 | Mitochondrial glutamate carrier 2 | Q9H1K4 | SLC25A18 |  |  |  |  |
| 3977 | 25215-1 | TIM10 | Mitochondrial import inner membrane translocase subunit Tim10 | P62072 | TIMM10 |  |  |  |  |
| 3978 | 23241-27 | T10B | Mitochondrial import inner membrane translocase subunit Tim10 B | Q9Y5J6 | TIMM10B |  |  |  |  |
| 3979 | 23621-326 | TIM13 | Mitochondrial import inner membrane translocase subunit Tim13 | Q9Y5L4 | TIMM13 |  |  |  |  |
| 3980 | 4545-53 | DnaJ homolog | Mitochondrial import inner membrane translocase subunit TIM14 | Q96DA6 | DNAJC19 | X |  | X |  |
| 3981 | 9058-1 | TIM21 | Mitochondrial import inner membrane translocase subunit Tim21 | Q9BVV7 | TIMM21 |  |  |  |  |
| 3982 | 22809-32 | TIM23 | Mitochondrial import inner membrane translocase subunit Tim23 | O14925 | TIMM23 |  |  |  |  |
| 3983 | 7743-5 | TIM50 | Mitochondrial import inner membrane translocase subunit TIM50 | Q3ZCQ8 | TIMM50 |  |  |  |  |
| 3984 | 19148-58 | TIM8A | Mitochondrial import inner membrane translocase subunit Tim8 A | O60220 | TIMM8A |  |  |  |  |
| 3985 | 20408-11 | TOM20 | Mitochondrial import receptor subunit TOM20 homolog | Q15388 | TOMM20 |  |  |  |  |
| 3986 | 22077-46 | ATP23 | Mitochondrial inner membrane protease ATP23 homolog | Q9Y6H3 | ATP23 |  |  |  |  |
| 3987 | 20389-36 | IMP2L | Mitochondrial inner membrane protease subunit 2 | Q96T52 | IMMP2L |  | X |  |  |
| 3988 | 7137-8 | MSRA | Mitochondrial peptide methionine sulfoxide reductase | Q9UJ68 | MSRA |  |  |  |  |
| 3989 | 23164-8 | MPC1 | Mitochondrial pyruvate carrier 1 | Q9Y5U8 | MPC1 |  |  |  |  |
| 3990 | 21752-10 | MIRO1 | Mitochondrial Rho GTPase 1 | Q8IXI2 | RHOT1 |  |  |  |  |
| 3991 | 11159-14 | MUL1:CD | Mitochondrial ubiquitin ligase activator of NFKB 1:Cytoplasmic domain | Q969V5 | MUL1 |  |  |  |  |
| 3992 | 9315-16 | MUL1:MIMD | Mitochondrial ubiquitin ligase activator of NFKB 1:Mitochindrial intermembrane domain | Q969V5 | MUL1 |  |  |  |  |
| 3993 | 24907-3 | MPPA | Mitochondrial-processing peptidase subunit alpha | Q10713 | PMPCA |  |  |  |  |
| 3994 | 11364-18 | MFN1 | Mitofusin-1 | Q8IWA4 | MFN1 |  |  | X |  |
| 3995 | 3115-64 | MK01 | Mitogen-activated protein kinase 1 | P28482 | MAPK1 | X | X | X | X |
| 3996 | 15418-25 | MK10 | Mitogen-activated protein kinase 10 | P53779 | MAPK10 |  |  |  |  |
| 3997 | 5004-69 | MK11 | Mitogen-activated protein kinase 11 | Q15759 | MAPK11 | X |  |  |  |
| 3998 | 5005-4 | MK12 | Mitogen-activated protein kinase 12 | P53778 | MAPK12 |  |  |  |  |
| 3999 | 5006-71 | MK13 | Mitogen-activated protein kinase 13 | O15264 | MAPK13 |  |  |  |  |
| 4000 | 5007-1 | MAPK14 | Mitogen-activated protein kinase 14 | Q16539 | MAPK14 | X |  |  | X |
| 4001 | 2855-49 | ERK-1 | Mitogen-activated protein kinase 3 | P27361 | MAPK3 | X | X | X | X |
| 4002 | 22858-3 | MK06 | Mitogen-activated protein kinase 6 | Q16659 | MAPK6 |  |  |  |  |
| 4003 | 3825-18 | MK08 | Mitogen-activated protein kinase 8 | P45983 | MAPK8 | X | X |  | X |
| 4004 | 15604-18 | JNK2 | Mitogen-activated protein kinase 9 | P45984 | MAPK9 | X |  |  | X |
| 4005 | 22987-69 | JNK2 | Mitogen-activated protein kinase 9 | P45984 | MAPK9 |  |  |  |  |
| 4006 | 9760-13 | JNK2 | Mitogen-activated protein kinase 9 | P45984 | MAPK9 |  |  |  |  |
| 4007 | 23162-36 | M3K10 | Mitogen-activated protein kinase kinase kinase 10 | Q02779 | MAP3K10 |  |  |  |  |
| 4008 | 21406-25 | M3K11 | Mitogen-activated protein kinase kinase kinase 11 | Q16584 | MAP3K11 |  |  |  |  |
| 4009 | 12990-39 | MAP3K3 | Mitogen-activated protein kinase kinase kinase 3 | Q99759 | MAP3K3 |  |  |  |  |
| 4010 | 21415-212 | MAP3K3 | Mitogen-activated protein kinase kinase kinase 3 | Q99759 | MAP3K3 |  |  |  |  |
| 4011 | 5259-2 | TAK1-TAB1 | Mitogen-activated protein kinase kinase kinase 7:TGF-beta-activated kinase 1 and MAP3K7- | O43318|Q15750 | MAP3K7|TAB1 | X |  |  |  |
| 4012 | 8954-30 | M4K1 | Mitogen-activated protein kinase kinase kinase kinase 1 | Q92918 | MAP4K1 |  |  |  |  |
| 4013 | 8978-30 | M4K3 | Mitogen-activated protein kinase kinase kinase kinase 3 | Q8IVH8 | MAP4K3 |  |  |  |  |
| 4014 | 21989-12 | M4K5 | Mitogen-activated protein kinase kinase kinase kinase 5 | Q9Y4K4 | MAP4K5 |  |  |  |  |
| 4015 | 12033-3 | BUB1 | Mitotic checkpoint serine/threonine-protein kinase BUB1 | O43683 | BUB1 |  |  |  |  |
| 4016 | 13618-15 | MD1L1 | Mitotic spindle assembly checkpoint protein MAD1 | Q9Y6D9 | MAD1L1 |  | X |  | X |
| 4017 | 15313-28 | MD2L1 | Mitotic spindle assembly checkpoint protein MAD2A | Q13257 | MAD2L1 |  |  |  |  |
| 4018 | 23404-16 | MD2L2 | Mitotic spindle assembly checkpoint protein MAD2B | Q9UI95 | MAD2L2 |  |  | X |  |
| 4019 | 24928-20 | MZT2A | Mitotic-spindle organizing protein 2A | Q6P582 | MZT2A |  |  |  |  |
| 4020 | 12426-19 | MOL1B | MOB kinase activator 1A | Q9H8S9 | MOB1A |  |  |  |  |
| 4021 | 19177-7 | MOL1A | MOB kinase activator 1B | Q7L9L4 | MOB1B |  |  |  |  |
| 4022 | 21815-7 | MOB3B | MOB kinase activator 3B | Q86TA1 | MOB3B |  |  |  |  |
| 4023 | 19332-1 | MOBL3 | MOB-like protein phocein | Q9Y3A3 | MOB4 |  |  |  |  |
| 4024 | 21327-12 | MRI | Modulator of retrovirus infection homolog | Q9BWK5 | CYREN |  |  |  |  |
| 4025 | 5009-11 | Moesin | Moesin | P26038 | MSN | X | X | X |  |
| 4026 | 24419-3 | MOCOS | Molybdenum cofactor sulfurase | Q96EN8 | MOCOS | X |  | X |  |
| 4027 | 7881-244 | PAR16 | Mono [ADP-ribose] polymerase PARP16 | Q8N5Y8 | PARP16 |  |  |  |  |
| 4028 | 7825-7 | ABD12 | Monoacylglycerol lipase ABHD12 | Q8N2K0 | ABHD12 |  | X |  |  |
| 4029 | 9049-2 | MOT4 | Monocarboxylate transporter 4 | O15427 | SLC16A3 |  |  |  | X |
| 4030 | 8969-49 | CD14 | Monocyte differentiation antigen CD14 | P08571 | CD14 |  | X |  |  |
| 4031 | 16914-104 | sCD14 | Monocyte differentiation antigen CD14, soluble | P08571 | CD14 |  |  | X |  |
| 4032 | 21203-14 | MR1L1 | MORF4 family-associated protein 1-like 1 | Q96HT8 | MRFAP1L1 |  |  |  |  |
| 4033 | 18193-165 | MO4L1 | Mortality factor 4-like protein 1 | Q9UBU8 | MORF4L1 |  |  |  |  |
| 4034 | 24443-8 | MO4L2 | Mortality factor 4-like protein 2 | Q15014 | MORF4L2 |  |  |  |  |
| 4035 | 9838-4 | SMAD1 | Mothers against decapentaplegic homolog 1 | Q15797 | SMAD1 | X |  |  |  |
| 4036 | 10364-6 | SMAD2 | Mothers against decapentaplegic homolog 2 | Q15796 | SMAD2 | X |  | X |  |

| # | **Custom Panel (X)** | **SOMAmer SeqID** | **Target Name** | **Human Target or Analyte** | **UniProt ID** | **GeneID** | **Cardiovascular Disease** | **Inflammation and Immune**  **Response** | **Metabolic Disease** | **Oncology** |
| --- | --- | --- | --- | --- | --- | --- | --- | --- | --- | --- |

| 4037 | 11353-143 | SMAD2 | Mothers against decapentaplegic homolog 2 | Q15796 | SMAD2 |  | | | |
| --- | --- | --- | --- | --- | --- | --- | --- | --- | --- |
| 4038 | 10363-13 | SMAD3 | Mothers against decapentaplegic homolog 3 | P84022 | SMAD3 | X | X |  | X |
| 4039 | 12022-12 | SMAD4 | Mothers against decapentaplegic homolog 4 | Q13485 | SMAD4 | X | X |  | X |
| 4040 | 20382-8 | MSPD1 | Motile sperm domain-containing protein 1 | Q9UJG1 | MOSPD1 |  |  |  |  |
| 4041 | 9576-58 | MNX1 | Motor neuron and pancreas homeobox protein 1 | P50219 | MNX1 |  |  | X |  |
| 4042 | 11548-84 | MPIP1 | M-phase inducer phosphatase 1 | P30304 | CDC25A |  |  |  |  |
| 4043 | 12427-8 | MPIP2 | M-phase inducer phosphatase 2 | P30305 | CDC25B | X |  |  |  |
| 4044 | 21310-37 | MRT4 | mRNA turnover protein 4 homolog | Q9UKD2 | MRTO4 |  |  |  |  |
| 4045 | 12847-27 | MCE1 | mRNA-capping enzyme | O60942 | RNGTT |  |  |  |  |
| 4046 | 20135-85 | MCE1 | mRNA-capping enzyme | O60942 | RNGTT |  |  |  |  |
| 4047 | 14008-22 | DCP1A | mRNA-decapping enzyme 1A | Q9NPI6 | DCP1A |  |  |  |  |
| 4048 | 12715-30 | DCP1B | mRNA-decapping enzyme 1B | Q8IZD4 | DCP1B |  |  |  |  |
| 4049 | 23301-2 | MSX-2 | MSX-2 | P35548 | MSX2 |  |  |  |  |
| 4050 | 24216-30 | AAMDC | Mth938 domain-containing protein | Q9H7C9 | AAMDC |  |  |  |  |
| 4051 | 25252-29 | MTSSL | MTSS1-like protein | Q765P7 | MTSS2 |  |  |  |  |
| 4052 | 10623-19 | MUC1:region 1 | Mucin-1:region 1 | P15941 | MUC1 | X | X |  | X |
| 4053 | 9176-3 | MUC1:region 2 | Mucin-1:region 2 | P15941 | MUC1 | X | X |  | X |
| 4054 | 10976-44 | MUC1:region 3 | Mucin-1:region 3 | P15941 | MUC1 | X | X |  | X |
| 4055 | 15565-102 | CA125 | Mucin-16 | Q8WXI7 | MUC16 |  | X |  | X |
| 4056 | 10975-59 | MUCL1 | Mucin-like protein 1 | Q96DR8 | MUCL1 |  |  |  |  |
| 4057 | 25300-39 | MALT1 | Mucosa-associated lymphoid tissue lymphoma translocation protein 1 | Q9UDY8 | MALT1 |  | X | X |  |
| 4058 | 11258-41 | MAdCAM-1 | Mucosal addressin cell adhesion molecule 1 | Q13477 | MADCAM1 |  |  |  |  |
| 4059 | 4923-79 | MIS | Muellerian-inhibiting factor | P03971 | AMH |  |  |  |  |
| 4060 | 8935-22 | MRP6 | Multidrug resistance-associated protein 6 | O95255 | ABCC6 | X |  | X |  |
| 4061 | 9841-197 | Multifunctional protein ADE2 | Multifunctional protein ADE2 | P22234 | PAICS |  |  |  |  |
| 4062 | 9723-105 | EMILIN-3 | Multimerin-2 | Q9H8L6 | MMRN2 |  |  |  |  |
| 4063 | 11168-3 | MEG10 | Multiple epidermal growth factor-like domains protein 10 | Q96KG7 | MEGF10 |  |  |  |  |
| 4064 | 5586-66 | MINP1 | Multiple inositol polyphosphate phosphatase 1 | Q9UNW1 | MINPP1 |  |  |  |  |
| 4065 | 14036-116 | MPDZ | Multiple PDZ domain protein | O75970 | MPDZ |  |  |  |  |
[truncated: 487,023 more chars]
